# Riluzole‐Derived Benzo[d]thiazoles as CK1*δ* Inhibitors for Amyotrophic Lateral Sclerosis‐Related Neurodegeneration

**DOI:** 10.1002/cmdc.70539

**Published:** 2026-10-22

**Authors:** Simone Adorinni, Ilenia Grieco, Stefano Morasso, Michelle Fornasier, Gianluca Novello, Veronica Salmaso, Loreto Martínez‐González, Eleonora Cescon, Davide Bassani, Giovanni Adriano Bassan, Teresa Gianferrara, Filippo Prencipe, Veronika Kost, Tatiana Da Ros, Paola Storici, Ana Martinez, Giampiero Spalluto, Stefano Moro, Stephanie Federico

**Affiliations:** ^1^ Department of Chemical and Pharmaceutical Sciences University of Trieste Trieste Italy; ^2^ Protein Targets for Drug Discovery Lab Structural Biology Elettra Sincrotrone Trieste S.C.p.A Basovizza Italy; ^3^ Molecular Modelling Section (MMS) Department of Pharmaceutical and Pharmacological Sciences University of Padua Padova Italy; ^4^ Centro de Investigaciones Biológicas, CSIC Madrid Spain

**Keywords:** benzo[d]thiazoles, Casein kinase 1*δ*, lateral sclerosis, metabolism‐based drug design, riluzole

## Abstract

Riluzole‐derived benzo[d]thiazoles were investigated as metabolite‐inspired CK1*δ* inhibitors, a kinase involved in TDP‐43 phosphorylation and therefore relevant to amyotrophic lateral sclerosis (ALS). Starting from the known CK1*δ* activity of riluzole, we examined whether its major hydroxylamine metabolite could preserve target recognition and provide a rational entry point for scaffold optimisation. The metabolite retained CK1*δ* inhibition, but its limited chemical stability and toxicity in neuronal cells identified the hydroxylamine group as a critical liability. Replacement of this functionality with a hydrazine converted the fragile metabolite‐derived structure into a more potent and tractable CK1*δ* inhibitor series. Structure–activity relationship studies revealed that the hydrazine moiety accommodates broader substitution than the hydroxylamine counterparts, with branched alkyl and benzyl groups giving the most balanced profiles. Docking and molecular dynamics suggested that the improvement could be attributed to an additional hydrogen‐bond with Gly86 in the hinge region. The best derivative combined submicromolar CK1*δ* inhibition with passive blood–brain barrier permeability, short‐term cellular tolerance, ATP competitive inhibition, direct target engagement, and preliminary selectivity over GSK3β. Although this compound did not result neuroprotective in an oxidative stress model, it paves the way for a focused benzo[d]thiazole platform for probing CK1*δ* biology in ALS.

## Graphical abstract

Riluzole metabolism guided the design of benzo[d]thiazole inhibitors of casein kinase 1 delta (CK1δ), a kinase linked to amyotrophic lateral sclerosis (ALS). Docking supported ATP site recognition by the hydroxylamine metabolite and hydrazine analogues, leading to compounds showing improved potency and retained predicted blood brain barrier permeability.

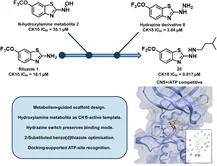

## 1 Introduction

Amyotrophic lateral sclerosis (ALS) is a fatal neurodegenerative disease characterised by the progressive loss of upper and lower motor neurons in the brain and spinal cord, leading to muscle atrophy, paralysis, and death, typically within 3–5 years of symptom onset [1, 2]. Although ALS is commonly classified as either familial (fALS) or sporadic (sALS), accounting for approximately 10% and 90% of cases, respectively, disease‐associated genetic variants have been identified in both forms. fALS is most often associated with mutations in genes such as SOD1, C9orf72, TARDBP, and FUS, whereas sALS is clinically defined by the absence of an apparent family history, although genetic risk factors and pathogenic variants can also contribute to a subset of these cases [1, 3]. Both forms converge on shared pathological features and remain associated with very limited therapeutic options. In this restricted therapeutic landscape, riluzole, a 2‐aminobenzo[d]thiazole derivative, remains a key clinical reference, having been the first disease‐modifying drug approved for ALS by the FDA in 1995. However, its clinical benefit is modest, with a survival extension of approximately 2–3 months and no concomitant improvement in motor function [4, 5, 6, 7]. Despite the subsequent approval of edaravone in 2017, AMX0035 (combination treatment of sodium phenylbutyrate and taurursodiol), subsequently withdrawn, and the antisense oligonucleotide (ASO) tofersen in 2023 for SOD1‐mutant ALS, no curative therapy is currently available, and the therapeutic impact of existing treatments remains limited (Figure 1) [8, 9, 10, 11]. This therapeutic gap continues to drive the search for molecular targets that are more directly connected to ALS pathobiology and are amenable to pharmacological modulation.

**FIGURE 1:**
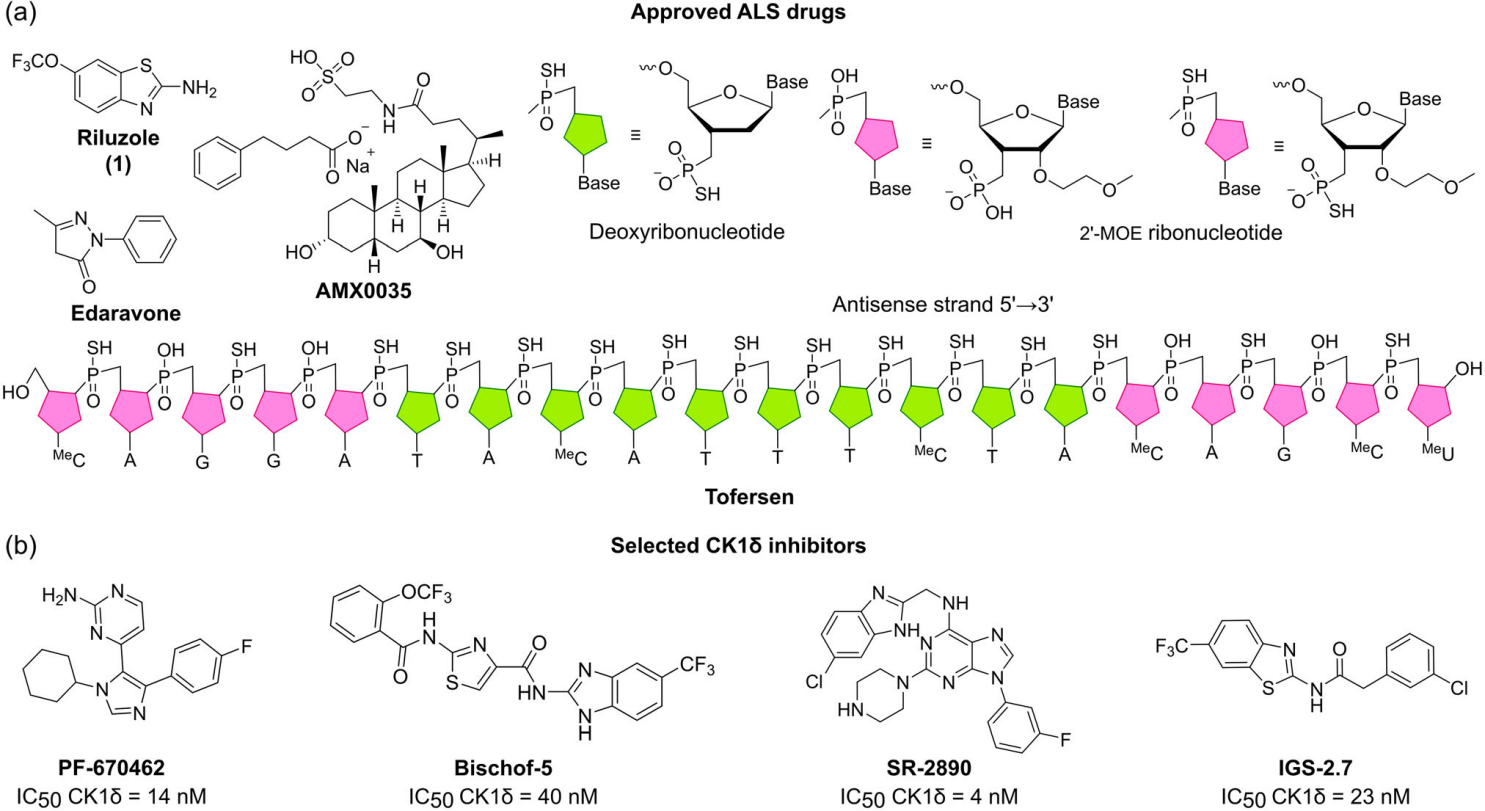
Approved ALS drugs and selected CK1δ inhibitors. (a) Chemical structures of approved ALS drugs discussed in the introduction. (b) Representative potent CK1*δ* inhibitors illustrating the structural diversity of ATP‐competitive chemotypes developed for this kinase. IGS‐2.7 is highlighted as the CK1*δ*inhibitor with the most direct validation in TDP‐43‐related ALS models.

Among the pharmacologically tractable targets emerging from these efforts, Casein Kinase 1 isoform *δ* (CK1*δ*) has attracted increasing interest. CK1*δ* is a constitutively active Ser/Thr kinase involved in the regulation of multiple cellular processes [12]. Its connection with ALS has been investigated primarily through transactive response DNA‐binding protein 43 kDa (TDP‐43), a major component of the ubiquitinated cytoplasmic inclusions observed in most ALS cases [3, 13]. CK1*δ* phosphorylates multiple residues within the C‐terminal region of TDP‐43 [14], while expression of a truncated, constitutively active form of CK1*δ* was shown to promote TDP‐43 phosphorylation, cytoplasmic mislocalisation, and accumulation in cellular models [15]. Increased CK1*δ* expression has also been reported in spinal cord and frontal cortex tissue from sALS patients [2, 16]. More recently, genetic reduction or pharmacological inhibition of CK1*δ*/ε was shown to decrease TDP‐43 phosphorylation in inducible cellular models [17]. Collectively, these observations have supported the use of CK1*δ* inhibitors as pharmacological tools for investigating kinase‐dependent processes in ALS‐related experimental systems. CK1*δ* has been targeted by several chemically diverse classes of ATP‐competitive inhibitors [18, 19, 20, 21, 22, 23, 24], including PF‐670462 (Figure 1), an imidazole‐based inhibitor whose crystallographic complex with CK1*δ* defined a hinge interaction with Leu85 and the occupation of a hydrophobic pocket within the ATP‐binding site [18], thiazole/benzimidazole derivatives such as Bischof‐5 (Figure 1), which showed nanomolar CK1*δ* inhibition and high kinase selectivity [20], and purine‐based inhibitors such as SR‐2890 (Figure 1) [23, 24].

Among the CK1*δ* inhibitor series advanced beyond biochemical characterisation, the N‐(benzothiazolyl)‐2‐phenylacetamides reported by Martínez and Pérez represent a relevant precedent [1]. Selected derivatives combined nanomolar CK1*δ* inhibition and kinase selectivity with predicted BBB permeability, reduction of TDP‐43 phosphorylation in cellular systems, and activity in a Drosophila model of TDP‐43 proteinopathy [1]. Within this series, IGS‐2.7 (Figure 1) was subsequently shown to modulate TDP‐43 phosphorylation, mislocalisation, and to limit the propagation of TDP‐43 pathology in patient‐derived cellular models [25, 26], and to reduce TDP‐43 phosphorylation and preserve motor neurons in vivo [2]. These studies illustrate the progression of potent CK1*δ* inhibitors from biochemical characterisation to evaluation in disease‐relevant experimental systems. Riluzole provides a distinct bridge between this CK1*δ* inhibitor landscape and ALS pharmacotherapy, as it is both an established ALS drug and a 2‐aminobenzo[d]thiazole derivative. Although the drug is conventionally described as a modulator of glutamatergic neurotransmission and a preferential inhibitor of persistent sodium currents, no clinically relevant direct targeting of glutamate receptors has been conclusively established, and several antiglutamatergic compounds have failed in ALS models [5, 6]. In 2018, our group reported that riluzole inhibits CK1*δ* with micromolar potency, with an IC_50_ of 16.1 µM [27]. Molecular docking and supervised molecular dynamics simulations supported an ATP‐site binding mode in which riluzole engages the CK1*δ* hinge region through interactions with Leu85, while additional hydrophobic contacts with Ile23, Tyr56, and Ile148 stabilise its accommodation within the catalytic pocket [27]. Although the biological contribution of this interaction to the clinical pharmacology of riluzole remains to be established, these findings identified riluzole as a structurally defined CK1*δ* inhibitor and its 2‐aminobenzo[d]thiazole core as a starting point for inhibitor development.

On this basis, we examined the metabolic fate of riluzole as a source of metabolite‐inspired CK1*δ* chemotypes (Figure 2). Riluzole is almost completely absorbed after oral administration but displays moderate absolute bioavailability, likely due in part to presystemic and first‐pass metabolism. Its oxidative metabolism is mediated mainly by hepatic CYP1A2, while extrahepatic CYP1A1 may contribute to the formation of additional metabolites and to systemic clearance [28, 29, 30]. The main phase I pathways involve hydroxylation of the benzo[d]thiazole ring, affording metabolites **5‐7**, and N‐hydroxylation at the 2‐amino position, generating the major metabolite RPR 112512 (**2**). This N‐hydroxylamine is rapidly subjected to O‐glucuronidation by UDP‐glucuronosyltransferases, yielding conjugate **3** and favouring its elimination [5, 29]. Minor oxidative pathways produce the dealkylated derivative **4** and additional unidentified metabolites [28, 30]. The preservation of the 6‐(trifluoromethoxy)benzo[d]thiazole core in metabolite **2** raised the question of whether N‐hydroxylation also preserves CK1*δ* recognition, while the newly introduced hydroxylamine functionality offered a chemically accessible site for further diversification.

**FIGURE 2:**
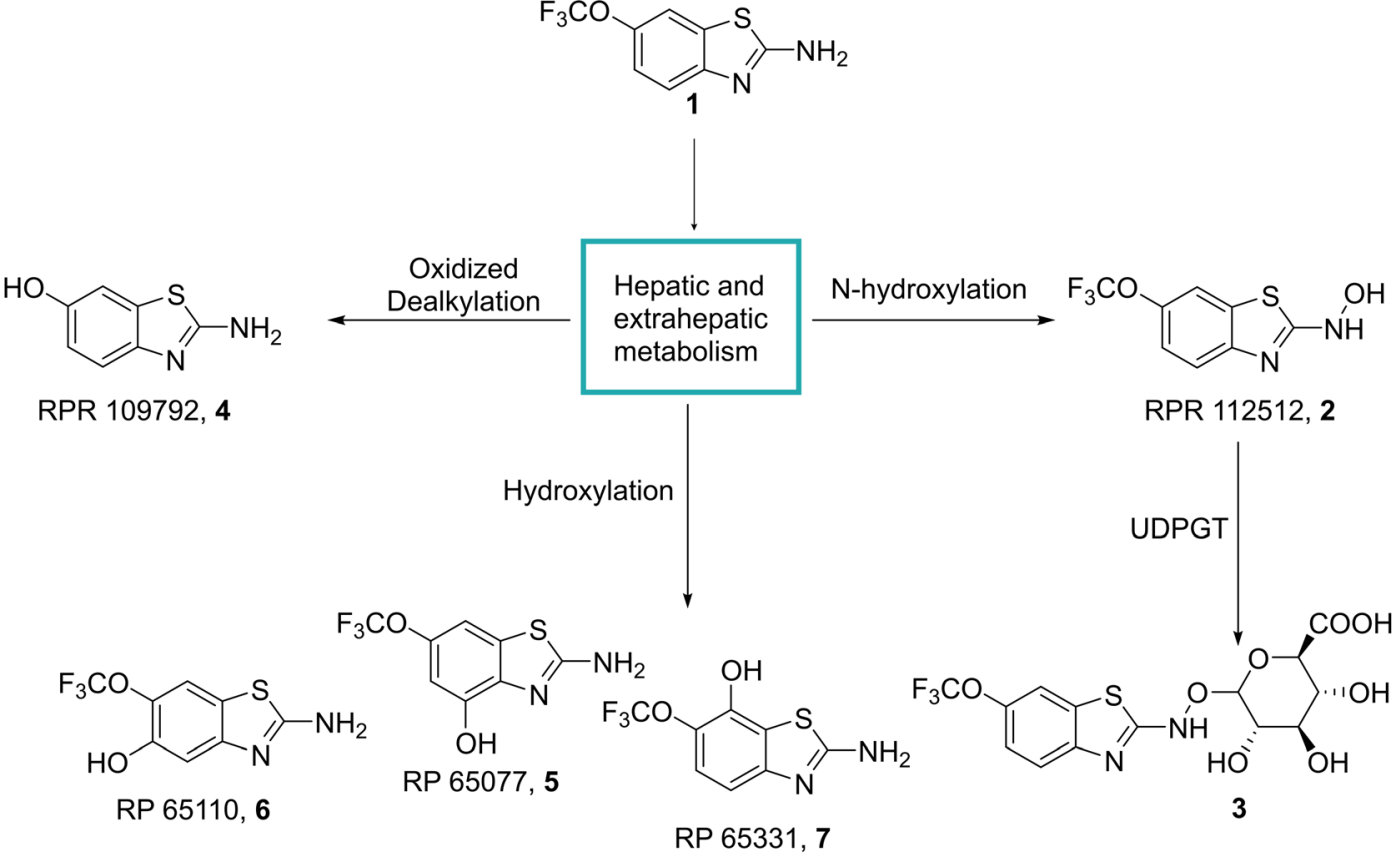
Metabolic pathway of riluzole (**1**). **1** undergoes phase I metabolism mainly through hydroxylation of the benzo[d]thiazole ring to give metabolites **5**–**7**, and through N‐hydroxylation of the 2‐amino group to afford RPR 112512 (**2**), the major oxidative metabolite. The N‐hydroxylamine **2** is rapidly converted by UDP‐glucuronosyltransferases into the corresponding O‐glucuronide conjugate **3**, which favours elimination. Minor oxidative pathways include formation of the dealkylated derivative **4** and additional unidentified metabolites.

In this study, we exploited riluzole metabolism as a medicinal chemistry entry point for the development of 2‐substituted 6‐(trifluoromethoxy)benzo[d]thiazole derivatives. We initially synthesised the N‐hydroxylamine metabolite **2** to determine whether N‐hydroxylation preserves CK1*δ* recognition. We then extended the investigation to O‐alkylhydroxylamines and the corresponding hydrazine derivatives, conceived as metabolite‐inspired analogues enabling broader structure–activity relationship exploration at the 2‐position of the scaffold. The resulting compounds were evaluated for CK1*δ* inhibitory activity, predicted binding mode, ATP‐competitive behaviour, direct target engagement, chemical stability, physicochemical profile, passive BBB permeability, and preliminary selectivity against GSK3β, CK1ε and p38α.

## 2 Results and Discussion

### 2.1 Design Rationale: From Riluzole Metabolism to CK1*δ*‐Targeted Benzo[d]thiazoles

The major N‐hydroxylamine metabolite **2** served as the starting point for assessing whether N‐hydroxylation preserves CK1*δ* recognition and introduces a suitable handle for structural diversification. Docking studies suggested that **2** could be accommodated within the CK1*δ* ATP‐binding site, with the benzo[d]thiazole core positioned in the adenine‐binding region, establishing interactions with Leu85 and the NH of the hydroxylamine engaging the same residue through a hydrogen bond (Figure 3a). These observations provided a structural rationale for considering this metabolite as a relevant CK1*δ*‐directed chemotype. At the same time, its N‐hydroxylamine functionality offered a clear point for chemical intervention since O‐glucuronidation of this group represents a major metabolic route for riluzole derivative **2** and is expected to favour its rapid elimination.

**FIGURE 3:**
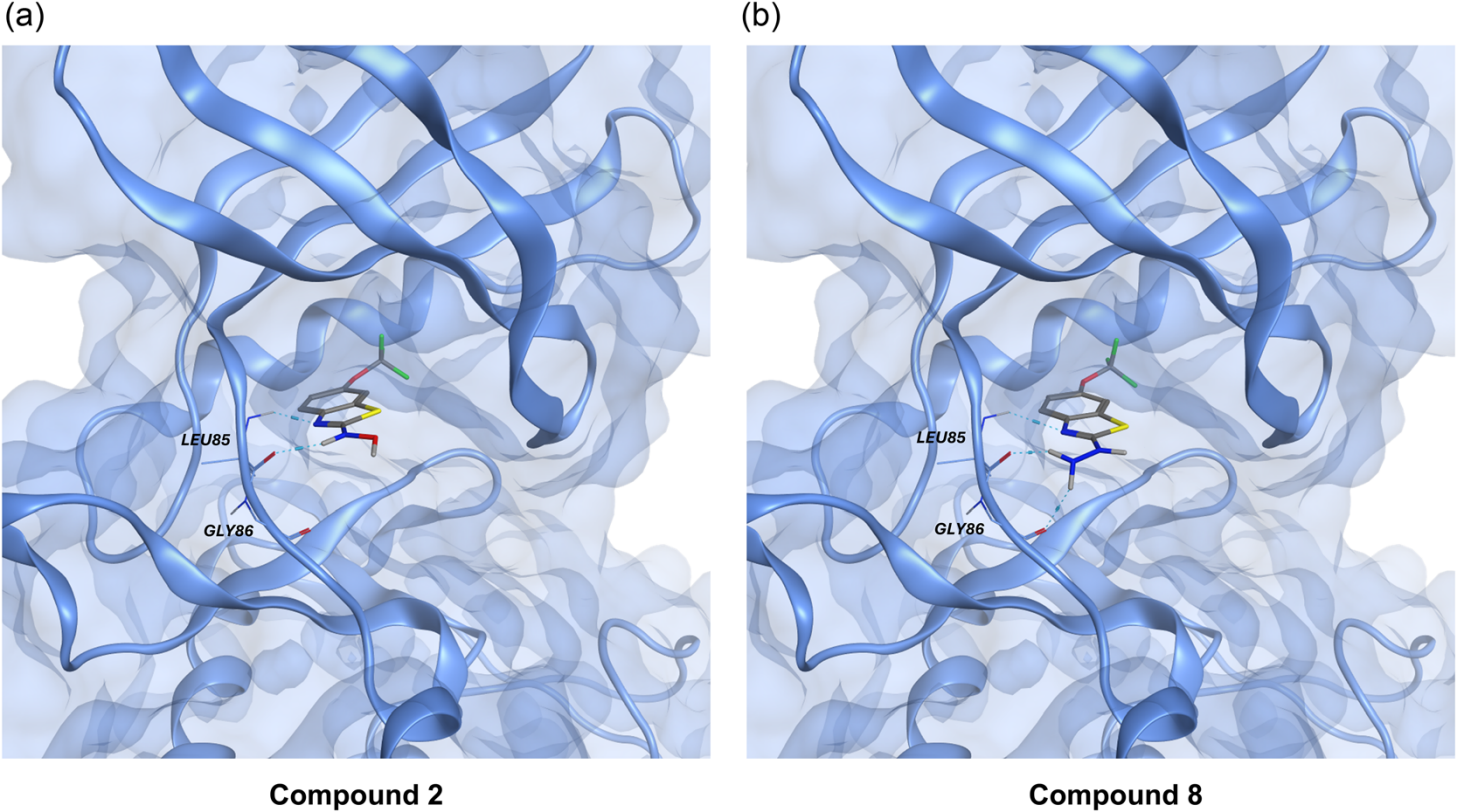
Predicted binding poses of metabolite **2** and hydrazine analogue **8** within the ATP binding site of CK1δ. (a) Docking pose of the N‐hydroxylamine metabolite RPR 112512, **2**, showing accommodation of the benzo[d]thiazole scaffold in the adenine binding region and predicted interactions with Leu85. (b) Docking pose of 2‐hydrazineyl‐6‐(trifluoromethoxy)benzo[d]thiazole, **8**, showing a closely related binding mode and preservation of the predicted hinge region interaction pattern. Docking was performed using the CK1δ crystal structure PDB ID 5OKT.

This consideration led us to explore two complementary strategies at the 2‐position of the benzo[d]thiazole scaffold (Figure 4). O‐alkylation of the hydroxylamine was envisaged as a straightforward means to block the major site of conjugation, while replacement of the hydroxylamine with a hydrazine moiety was explored as a bioisosteric alternative that removes the hydroxylamine O‐glucuronidation site and enables broader structural diversification. Docking of 2‐hydrazineyl‐6‐(trifluoromethoxy)benzo[d]thiazole (**8**) predicted a binding pose virtually superimposable with that of **2**, preserving the hydrogen‐bonding network with Leu85 in the hinge region (Figure 3b), with an additional interaction with Gly86, thus supporting this replacement as a viable design strategy. Compound **8** was synthesised and evaluated on CK1*δ*, affording an IC_50_ of 3.64 μM, a nearly tenfold improvement over metabolite **2**, which identified the hydrazine‐containing analogue as a more potent starting point for SAR exploration while leaving the toxicological implications of this structural alert unresolved. We therefore designed and synthesised a series of 2‐substituted 6‐(trifluoromethoxy)benzo[d]thiazole derivatives in both the hydroxylamine and hydrazine series to explore their structure–activity relationships toward CK1*δ* inhibition.

**FIGURE 4:**
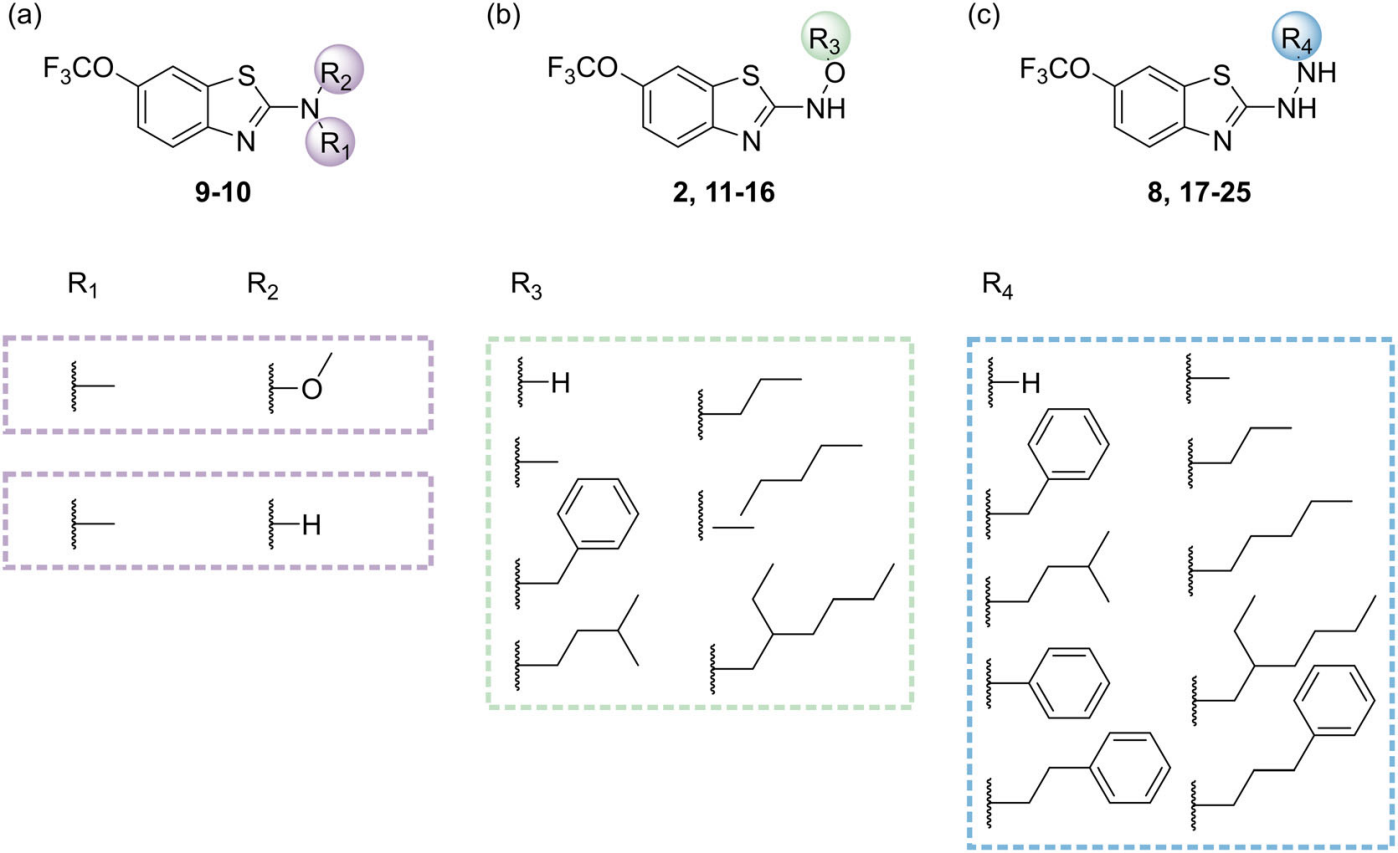
Overview of the synthetic diversification performed on the 6‐(trifluoromethoxy)benzo[d]thiazole scaffold. The library was divided into three sub‐series according to the linker introduced at C‐2: directly substituted N‐functionalised derivatives, **9**, and **10**, O‐alkyl‐N‐(6‐(trifluoromethoxy)benzo[d]thiazol‐2‐yl)hydroxylamine hydrochlorides, **2**, **11–16** and 2‐(2‐alkylhydrazineyl)‐6‐(trifluoromethoxy)benzo[d]thiazole hydrochlorides, **8, 17–25**. The positions diversified across the series are indicated by R_1_ to R_4_.

### 2.2 Chemistry

A focused library of nineteen 2‐amino‐6‐(trifluoromethoxy)benzo[d]thiazole derivatives bearing hydroxylamine, hydrazine, or amino substituents at C‐2 was prepared (Figure 4). The compounds were organised into three sub‐series according to the nature of the heteroatom linker attached to the benzo[d]thiazole core: (i) N‐methylated derivatives (**9–10)**; (ii) hydroxylamino derivatives (**2**, **11–16**); and (iii) hydrazino derivatives (**8**, **17–25**). In all three subseries, 2‐chloro‐6‐(trifluoromethoxy)benzo[d]thiazole (**26**), a commercially available electrophile, served as the common building block. Direct nucleophilic substitution of **26** was used for the simpler derivatives, as shown in Scheme 1. For the systematic preparation of the O‐alkylhydroxylamine and N‐alkylhydrazine subseries, the commercially available or synthesised nucleophilic partners were converted into Boc‐protected intermediates before palladium‐catalysed Buchwald–Hartwig amination. In the hydroxylamine series, Boc protection was introduced to control chemoselectivity and limit over‐arylation, whereas the di‐Boc strategy adopted for the hydrazine series enabled selective N‐alkylation, followed by coupling with **26**.

**SCHEME 1:**
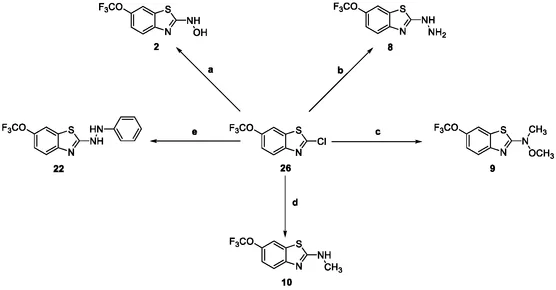
Synthesis of *N*‐substituted‐(6‐(trifluoromethoxy)benzo[d]thiazol‐2‐amine derivatives **2**, **8–10** and **22**. Reagents and conditions. a: NH_2_OH·HCl, K_2_CO_3_, MeOH, 65 °C, 4 h, Ar; b: NH_2_NH_2_·H_2_O, MeOH, rfx, 2 h; c: NH(CH_3_)OCH_3_·HCl, Et_3_N, BuOH, rfx, 24 h; d: NH_2_CH_3_·HCl, Et_3_N, BuOH, MW, 150 °C, 1 h; e: NH_2_NH_2_Ph, BuOH, rfx, 3 h.

#### 2.2.1 *N*‐Substituted 6‐(Trifluoromethoxy)benzo[d]thiazol‐2‐amine Derivatives (2, 9–10, 22)

*N*‐Substituted 6‐(trifluoromethoxy)benzo[d]thiazol‐2‐amine derivatives **2**, **9–10**, and **22** were prepared by nucleophilic aromatic substitution of the C‐2 chloride in compound **26** using commercially available hydroxylamine, hydrazine, or alkylamino nucleophiles in refluxing alcohols, with addition of base where required (Scheme 1) [31]. Treatment of compound **26** with hydroxylamine hydrochloride in methanol in the presence of potassium carbonate at 65 °C under argon for 4 h furnished the riluzole‐derived hydroxylamine **2** in 39% yield. This compound proved markedly unstable, undergoing rapid discolouration on exposure to air and gradual degradation during handling. Accordingly, the reaction was performed under an inert atmosphere throughout, and the product was immediately converted into its hydrochloride salt by treatment with hydrochloric acid in 1,4‐dioxane. Despite these precautions, compound **2** remained the most challenging member of the series to isolate and store in pure form, a feature that became particularly relevant during the subsequent stability and biological studies.

Hydrazine monohydrate reacted cleanly with **26** in refluxing methanol to afford the parent hydrazine **8** in a 42% yield. The N,O‐dimethylhydroxylamine derivative **9** was obtained in 58% yield from *N*‐methyl methoxyamine hydrochloride in refluxing butanol in the presence of triethylamine for 24 h. Access to the known N‐methyl analogue **10** was achieved by reacting **26** with methylamine hydrochloride and triethylamine under microwave irradiation in butanol at 150 °C for 1 h, affording the product in 33% yield. This derivative had previously been investigated for its antiglutamatergic activity and for inhibitory activity against the bacterial histidine kinase HK853, resulting in both cases being inactive [32, 33]. In the present study, compound **10** was included to assess the effect of N‐methylation on CK1*δ* inhibition. Finally, treatment of **26** with phenylhydrazine in refluxing butanol for 3 h afforded arylhydrazine **22** in a 10% yield. The low yield was attributed to the competing decomposition of phenylhydrazine under the reaction conditions. Notably, unlike the *O*‐alkylhydroxylamine and *N*‐alkylhydrazine sub‐series discussed below, arylhydrazine **22** was accessible by direct nucleophilic substitution and did not require palladium catalysis.

#### 2.2.2 O‐Alkyl‐N‐(6‐(trifluoromethoxy)benzo[d]thiazol‐2‐yl)hydroxylamine Hydrochlorides (11–16)

The introduction of non‐commercial O‐alkylhydroxylamino chains demanded a convergent four‐step sequence (Scheme 2). In the first stage, the required O‐alkylhydroxylamines were assembled through a Gabriel‐type approach. N‐hydroxyphthalimide (**27**), a commercially available reagent, was O‐alkylated with the appropriate alkyl bromide (propyl, pentyl, isopentyl, or 2‐ethylhexyl) using potassium carbonate as the base in dry DMF under argon at room temperature for 72 h, providing 2‐alkyloxyisoindoline‐1,3‐dione intermediates **28–31** in 60%–94% yield. The phthalimide ring was then cleaved by hydrazinolysis following the Ing–Manske procedure (hydrazine monohydrate, THF, rt, 12 h), liberating the free O‐alkylhydroxylamines [34]. These intermediates were not isolated as free bases owing to their volatility; instead, they were directly converted to their hydrochloride salts (**33–35, 37**) by treatment with hydrochloric acid in 1,4‐dioxane (yields 46%–67% for the two‐step Gabriel/hydrazinolysis sequence).

**SCHEME 2:**
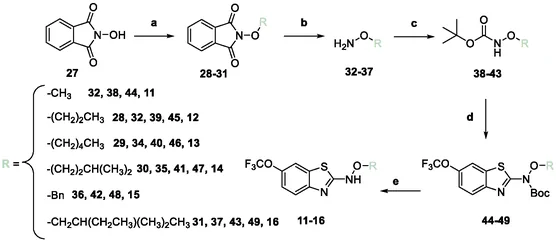
Synthesis of O‐substituted‐N‐(6‐(trifluoromethoxy)benzo[d]thiazol‐2‐yl)hydroxylamine hydrochloride derivatives **11–16**. Reagents and conditions. a: RBr, K_2_CO_3_, dry DMF, Ar, rt, 72 h; b: NH_2_NH_2_·H_2_O, THF, rt, 12 h; HCl in 1,4‐dioxane, rt, 5 min; c: (i) Et_3_N, CH_3_CN, rt, 90 min; (ii) Boc_2_O, Et_3_N, CH_3_CN, rt, 12 h; d: 2‐chloro‐6‐(trifluoromethoxy)benzo[d]thiazole (**26**), Pd_2_(dba)_3_, DPPF, Cs_2_CO_3_, dry CH_3_CN, 40 °C, 12 h; e: AcCl, MeOH, EtOAc, 0 °C to rt, 12 h (compounds **11**, **13–16**) or HCl in 1,4‐dioxane, rt, 1 h (compound **12**).

A critical design consideration arose at the next stage. Preliminary experiments indicated that direct coupling of the free O‐alkylhydroxylamines with chlorobenzothiazole **26** could result in an overreaction, leading to O‐alkyl‐N,N‐bis(6‐(trifluoromethoxy)benzo[d]thiazol‐2‐yl)hydroxylamine by‐products. To circumvent this problem, the amino group of each O‐alkylhydroxylamine, both of commercially available hydrochorides of methoxyamine **32** and O‐benzylhydroxylamine **36** and the synthetised **33–35, 37**, was protected with a *tert*‐butyloxycarbonyl (Boc) group prior to the palladium‐catalysed coupling step. Treatment with di‐*tert*‐butyl dicarbonate and triethylamine in acetonitrile at room temperature afforded *tert*‐butyl alkyloxycarbamates **38–43** in 34%–95% yield [35].

The pivotal C─N bond formation between Boc‐protected O‐alkylhydroxylamines **38–43** and chlorobenzothiazole **26** was accomplished via Buchwald–Hartwig amination [36, 37]. The catalytic system consisted of Pd_2_(dba)_3_ (0.15 equiv) and 1,1′‐bis(diphenylphosphino)ferrocene (DPPF, 0.20 equiv) as ligand, with cesium carbonate (1.6 equiv) as the base in anhydrous acetonitrile at 40 °C for 12 h under argon. This relatively mild temperature was selected for the hydroxylamine sub‐series to minimise Boc cleavage and preserve the integrity of the N─O bond. Under these conditions, Boc‐protected intermediates **44–49** were obtained in a 3%–40% yield. The generally low to moderate yields for this transformation reflect the inherent challenges of coupling sterically encumbered nitrogen nucleophiles with a heteroaromatic chloride. Notably, the propyloxy derivative **45** was obtained as the free hydroxylamine directly from the coupling step, likely due to concomitant Boc cleavage under the reaction conditions; this compound was immediately carried forward to the deprotection step without additional purification as its Boc‐protected form.

Boc removal was accomplished by two complementary methods. Method A involved in situ generation of hydrochloric acid from acetyl chloride added dropwise to methanol at 0 °C, followed by the addition of the Boc‐protected substrate in ethyl acetate and stirring at room temperature overnight. This approach was applied to compounds **11**, **13–16**. Method B used hydrochloric acid in 1,4‐dioxane at room temperature for 1 h and was employed for compound **12**. Both protocols furnished the target hydrochloride salts as white solids in a 34%–90% yield for this final step. The benzyloxy derivative **15** and the pentyloxy analogue **13** gave the highest yields (90% and 60%, respectively) in the deprotection step, whereas the methoxy derivative **12** was obtained in a 34% yield.

#### 2.2.3 2‐(2‐Alkylhydrazineyl)‐6‐(trifluoromethoxy)benzo[d]thiazole Hydrochlorides (17–21, 23–25)

The parallel hydrazine sub‐series followed a conceptually analogous Boc‐protection/Buchwald–Hartwig/deprotection strategy, although specific reaction parameters differed significantly from the hydroxylamine route (Scheme 3). *Tert*‐butyl carbazate (**50**) was treated with di‐*tert*‐butyl dicarbonate under neat conditions at room temperature for 10 min to provide di‐*tert*‐butyl hydrazine‐1,2‐dicarboxylate (**51**) in quantitative yield. Subsequent N‐alkylation of **51** with the appropriate alkyl or benzyl halide (cesium carbonate, dry DMF, rt, 12 h, Ar) furnished the mono‐N‐alkylated Boc‐protected hydrazines **52–59** [38]. Yields for this step ranged from 22% (phenethyl, **58**) to 62% (benzyl, **57**), with linear alkyl chains typically affording intermediate values (e.g., methyl, **52**, 67%; propyl, **53**, 62%; pentyl, **54**, 29%). The branched 2‐ethylhexyl derivative **56** was obtained in a 39% yield.

**SCHEME 3:**
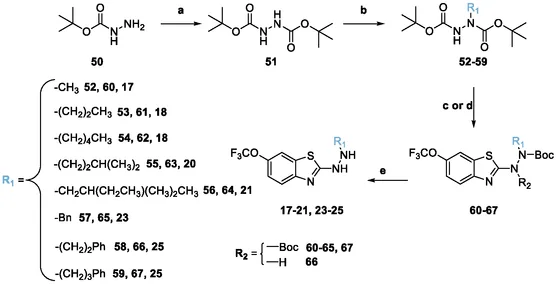
Synthesis of 2‐(2‐alkylhydrazineyl)‐6‐(trifluoromethoxy)benzo[d]thiazole hydrochloride derivatives **17–21, 23–25**. Reagents and conditions. a: Boc_2_O, neat, rt, 10 min; b: R_1_X, Cs_2_CO_3_, dry DMF, rt, 12 h, Ar; c: 2‐chloro‐6‐(trifluoromethoxy)benzo[d]thiazole (**26)**, Pd_2_(dba)_3_, DPPF, Cs_2_CO_3_, dry CH_3_Ph, 110 °C, 12 h, Ar, for compounds **52–55**; d: 2‐chloro‐6‐(trifluoromethoxy)benzo[d]thiazole (**26**), Pd_2_(dba)_3_, DPPF, Cs_2_CO_3_, dry CH_3_CN, 60 °C, 12 h, Ar, for compounds **56–59**; e: AcCl, MeOH, EtOAc, 0 °C to rt, 12 h or HCl in Et_2_O, rt, 1 h.

The Buchwald–Hartwig coupling of Boc‐protected hydrazines **52–59** with chlorobenzothiazole **26** employed the same Pd_2_(dba)_3_/DPPF catalytic system and cesium carbonate as the base [37], with two alternative procedures: toluene at 110 °C were the conditions used for linear alkyl substrates (**60–63**), while acetonitrile at 60 °C was used for sterically demanding or benzylic partners (**64–67**). Yields for the coupling step ranged widely, from 65% for the methyl derivative **60** to 3% for the sterically encumbered 2‐ethylhexyl compound **64**. The benzyl analogue **65** was obtained in a 36% yield, whereas the phenethyl (**66**, 8%) and 3‐phenylpropyl (**67**, 6%) derivatives were significantly less efficient. In the case of compound **66**, only the mono‐Boc intermediate was isolated, suggesting the partial cleavage of one protecting group under the reaction conditions. Finally, Boc deprotection performed using classical methods like acetyl chloride in methanol and hydrochloric acid in diethyl ether led to the final compounds **17–21, 23–25.**

### 2.3 Structure–Activity Relationship Studies

The SAR of the 2‐substituted 6‐(trifluoromethoxy)benzo[d]thiazole series was dominated by changes introduced at the 2‐position. Comparison of the first active derivatives, **2** and **8**, showed that even a minimal modification in this region could strongly affect CK1*δ* inhibition, and this finding guided the subsequent exploration of hydroxylamine and hydrazine analogues bearing linear, branched, or arylalkyl substituents.

Compounds **2**, **8–25**, and the Boc‐protected derivatives **48–49** and **63–65** were screened against truncated recombinant CK1*δ* using a luminescence‐based kinase assay at a fixed concentration of 40 µM, and IC_50_ values were determined for derivatives that produced at least 50% inhibition at the screening concentration. Within this framework, the N‐hydroxylamine metabolite **2** displayed an IC_50_ of 35.1 µM. This value places the metabolite in the same general activity range as riluzole itself and therefore supports the hypothesis that the major phase I metabolite retains the ability to engage CK1*δ*. Although no gain in potency was observed at this stage, the result was still relevant from a mechanistic perspective. These data indicate that effective CK1*δ* inhibition in this scaffold depends on preserving an accessible donor pattern at the 2‐position, with the proximal NH appearing to play a particularly relevant role. This trend is consistent with the preliminary docking model, which placed this region close to the hinge interaction network.

A first key structural change was then introduced by replacing the hydroxyl group of **2** with an amino group, thus generating the hydrazineyl derivative **8**. This modification proved highly productive since **8** showed an IC_50_ of 3.64 µM, corresponding to an approximately tenfold improvement relative to **2** (Table 1). This result clearly identified the hydrazine motif as a more favourable linker than the corresponding hydroxylamine in this benzo[d]thiazole series. On the basis of the docking model, this gain may arise from a more favourable donor arrangement at the hinge region together with a different conformational profile of the 2‐substituent (Figure 3b). Compound **8** established the hydrazine series as a more promising direction for further optimisation, converting a weakly active metabolite‐derived scaffold into a low micromolar hit.

**TABLE 1:** IC_50_s on CK1δ of compounds 2, 8–10.

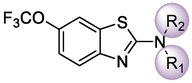				
Compounds	R_1_	R_2_	IC_50_ μM^a^ (% activity at 40 μM)^b^	BBB perm.^c^
**2**	─H	−hydroxy	35.1 ± 4.0	CNS+
**8**	─H	−amino	3.64 ± 0.58	CNS+
**9**	−methyl	−methoxy	n.d. (102% ± 9)	n.d.
**10**	−methyl	─H	n.d. (52.3% ± 0.9)	n.d.

Abbreviation: n.d., not determined.

a Data represent the mean ± SE of three independent experiments performed in technical duplicate.

b Data represent the % of activity at 40 μM concentration expressed as a mean ± SD of two independent experiments performed in technical duplicate.

c BBB behaviour was classified based on BBB‐PAMPA *P*_app_ thresholds, categorising compounds as permeable (CNS+), non‐permeable (CNS−), or intermediate permeability (CNS+/−) (see Supporting Information for details).

To examine the importance of hydrogen‐bond donor functionality at the 2‐position, a small group of methylated analogues was considered. Methylation of the proximal amino nitrogen, as in **10**, led to a loss of activity at 40 µM, while the doubly capped and O‐methylated **9** was likewise weakly active (Table 1). These data show that masking the donor region around the 2‐position is poorly tolerated. More specifically, they indicate that hydrogen‐bond donation in this region is important for CK1*δ* inhibition, with the proximal NH appearing to play a particularly relevant role. Productive binding therefore seems to depend on maintaining an accessible donor arrangement at the 2‐position, while progressive methylation disrupts this interaction pattern and strongly reduces activity.

Having established **8** as the more promising intermediate, the hydroxylamine and hydrazine series were next expanded through the installation of linear, branched, and arylalkyl substituents. The O‐alkyl hydroxylamine subseries showed a rather narrow SAR (Table 2). O‐methyl derivative **11** and O‐propyl derivative **12** were inactive under the assay conditions, and also the bulkier O‐(2‐ethylhexyl) analogue **16** failed to provide meaningful inhibition. The O‐benzyl derivative **15** retained the modest activity of metabolite **2**, with an IC_50_ of 22.6 µM, and therefore offered little real improvement over the parent hydroxylamine motif. Within this entire subset, the only clearly productive modification was the introduction of a linear pentyl chain, since derivative **13** reached an IC_50_ of 4.40 µM and was the sole hydroxylamine analogue to enter the low micromolar range. By contrast, the isopentyl derivative **14** did not show the same improvement observed for the linear pentyl analogue **13** (Table 2). This comparison indicates that the activity of **13** is not simply due to higher lipophilicity or larger substituent size, but depends on the linear arrangement of the pentyl chain. Thus, within the hydroxylamine series, effective CK1*δ* inhibition was achieved only with a specific linear alkyl substituent, whereas branching or further steric expansion was not favourable.

**TABLE 2:** IC_50_s on CK1δ of hydroxylamino compounds 11–16 and 48–49.

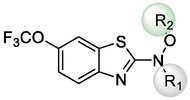				
Compounds	R_1_	R_2_	IC_50_ μM^a^ (% activity at 40 μM)^b^	BBB perm.^c^
**11**	─H	−methyl	n.d. (52.6% ± 2.8)	n.d.
**12**	─H	−propyl	n.d. (95.9% ± 9.5)	n.d.
**13**	─H	−pentyl	4.40 ± 0.21	CNS+
**14**	─H	−isopentyl	n.d. (96.9% ± 3.2)	n.d.
**15**	─H	−benzyl	22.6 ± 12.4	n.d.
**16**	─H	−ethylhexyl	n.d. (60.8% ± 10.4)	n.d.
**48**	−Boc	−benzyl	22.8 ± 5.3	CNS+
**49**	−Boc	−ethylhexyl	n.d. (95.9% ± 5.2)	n.d.

Abbreviation: n.d., not determined.

a Data represent the mean ± SE of three independent experiments performed in technical duplicate.

b Data represent the % of activity at 40 μM concentration expressed as a mean ± SD of two independent experiments performed in technical duplicate.

c BBB behaviour was classified based on BBB‐PAMPA *P*_app_ thresholds, categorising compounds as permeable (CNS+), non‐permeable (CNS−), or intermediate permeability (CNS+/−) (see Supporting Information for details).

The comparison between **15** and its Boc‐protected precursor **48** is also instructive. These two compounds showed closely similar IC_50_ values, 22.6 and 22.8 µM, respectively (Table 2). This similarity indicates that the introduction of the benzyl group on the hydroxylamine oxygen did not improve potency in this series. This residual activity is therefore likely associated with preservation of the benzo[d]thiazole recognition motif, while substitution or protection of the hydroxylamine region does not provide a favourable interaction pattern.

A markedly different picture emerged in the N‐alkyl hydrazine series (Table 3). In this case, substitution was much better tolerated, and the resulting activity profile was clearly stronger. Methyl, propyl, and pentyl derivatives **17**, **18**, and **19** all retained low micromolar activity, with IC_50_ values of 6.77, 9.89, and 5.81 µM, respectively. Thus, even relatively simple alkyl substitution on the hydrazine nitrogen preserved a meaningful degree of potency. More importantly, branching proved to be especially advantageous in this subseries. The isopentyl derivative **20** reached an IC_50_ of 0.917 µM and was the most potent compound of the whole benzo[d]thiazole set, entering the submicromolar range. The benzyl derivative **23** was also highly active, with an IC_50_ of 1.62 µM. Taken together, these data identify the hydrazine series as clearly superior to the corresponding hydroxylamine analogues, both in terms of overall potency and in terms of tolerance toward substituent variation (Table 3).

**TABLE 3:** IC_50_s on CK1δ of hydrazino compounds 17–25, 63, 65.

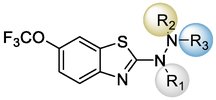					
Compounds	R_1_	R_2_	R_3_	IC_50_ μM^a^ (% activity at 40 μM)^b^	BBB perm.^c^
**17**	─H	─H	−methyl	6.77 ± 1.21	CNS+
**18**	─H	─H	−propyl	9.89 ± 2.83	CNS+
**19**	─H	─H	−pentyl	5.81 ± 0.74	n.d.
**20**	─H	─H	−isopentyl	0.917 ± 0.086	CNS+
**21**	─H	─H	−ethylhexyl	n.d. (85.5% ± 6.2)	n.d.
**22**	─H	─H	−phenyl	2.67 ± 1.10	CNS+
**23**	─H	─H	−benzyl	1.62 ± 0.29	CNS+
**24**	─H	─H	−phenethyl	2.52 ± 0.59	CNS+
**25**	─H	─H	−phenylpropyl	2.30 ± 0.32	n.d.
**63**	−Boc	−Boc	−pentyl	n.d. (85.4% ± 4.7)	n.d.
**65**	−Boc	−Boc	−benzyl	22.7 ± 5.4	n.d.

Abbreviation: n.d., not determined.

a Data represent the mean ± SE of three independent experiments performed in technical duplicate.

b Data represent the % of activity at 40 μM concentration expressed as a mean ± SD of two independent experiments performed in technical duplicate.

c BBB behaviour was classified based on BBB‐PAMPA *P*_app_ thresholds, categorising compounds as permeable (CNS+), non‐permeable (CNS−), or intermediate permeability (CNS+/−) (see Supporting Information for details).

The only clearly unsuccessful hydrazine analogue was the bulky 2‐ethylhexyl derivative **21**, which was inactive at the screening concentration, probably indicating a steric limit within the region explored by the distal substituent. The pocket appears to accommodate medium‐sized alkyl and arylalkyl groups, while tolerance decreases for larger, more highly branched substituents. These data again suggest that the benzo[d]thiazole core provides a basal level of recognition, but that meaningful potency depends on preserving the hydrazine donor pattern together with an appropriate substituent size and topology.

The role of aromatic substitution in the hydrazine series was then examined in more detail through comparison of derivatives differing in the spacer length between the hydrazine nitrogen and the phenyl ring. Phenylhydrazine **22** displayed an IC_50_ of 2.67 µM, benzyl derivative **23** gave 1.62 µM, while the phenethyl and phenylpropyl analogues **24** and **25** showed IC_50_ values of 2.52 and 2.30 µM, respectively (Table 3). The spread of activity across this subgroup is therefore relatively modest. The main conclusion is that aromatic substitution is broadly tolerated in this part of the series, whereas the spacer length exerts only a limited influence. Notably, within this set, the benzyl analogue was the most active member.

A matched comparison between the hydroxylamine and hydrazine subseries best captures the central SAR trend of the study. For the same or closely related substituents, the hydrazine analogues were consistently superior or at least comparable to the corresponding hydroxylamine derivatives. This is apparent not only in the parent pair **8** versus **2** but also in comparisons such as **23** versus **15** and, qualitatively, in the broader contrast between the permissive hydrazine profile and the far narrower hydroxylamine profile. The most important structural lesson from the series is therefore that replacement of the *β*‐oxygen of the hydroxylamine by a *β*‐nitrogen represents the most productive modification identified within this benzo[d]thiazole scaffold. A plausible explanation is that the hydrazine linker combines a more favourable donor arrangement with greater conformational adaptability, in agreement with the docking analysis.

### 2.4 Computational Studies

In the molecular modelling studies conducted on the synthesised compounds, a computational structure‐based approach was carried out, starting from the available crystallographic complexes in the Protein Data Bank [39]. A CK1*δ* 3D structure was selected on the basis of the closest scaffold similarity between the co‐crystallized inhibitor and the series under investigation. The selection was made in favour of the CK1*δ* structure coded as 5OKT, in complex with a 2‐aminobenzothiazole‐like inhibitor, previously employed as a reference structure in a published computational study on the CK1*δ* inhibition by Riluzole [27]. The docking reliability was validated by redocking the co‐crystallized ligand to the protein ATP‐binding site (low heavy‐atom RMSD value of 2.1 Å, 0.86 Å focusing on the 2‐aminobenzothiazole scaffold), confirming the suitability of the selected structure for the subsequent docking campaign.

The selection of the representative binding poses was carried out through a combined approach based on the analysis of electrostatic and hydrophobic interaction energies between the ligand and its recognition site, and subsequently by filtering the poses through a pharmacophore model built on the strongest interactions of the co‐crystallized inhibitor with CK1*δ* in structure 5OKT. In particular, the pharmacophore filtering step retained only the docking poses capable of preserving a bidentate hydrogen bond with Leu85, as well as the positions of the aromatic groups of the benzothiazole ring. An overview of the selected poses, together with the per‐residue interaction analysis for the entire series presented as heat maps of electrostatic and hydrophobic contributions, is provided in Video S1. The predominant electrostatic contributions are mediated by Glu83, Leu84, and Leu85 within the hinge region, together with Asp91 located at the outer edge of the binding site, while interactions with Ile23 and Leu135 represent the most prominent hydrophobic contributions across the series. Leu85 is involved in the typical bidentate hydrogen bond with the thiazole endocyclic nitrogen atom and the exocyclic amino group at position 3. Furthermore, analysis of the docking poses shown in Video S1 suggests that substitution at the amino group in position 2 of the thiazole ring plays a key role in defining the binding mode within the receptor. The presence of a disubstituted amine, as in compounds **9**, **48**, **49**, **62** and **65**, results in a complete loss of inhibitory activity or IC_50_ value above 20 µM, confirmed by the lack of the hydrogen bond with Leu85, underlining the importance of a hydrogen bond donor at this position.

All structural modifications within the series are confined to a side chain positioned outside the binding pocket and exposed to the solvent, as clearly observed in the docking pose analysis. Among monosubstituted analogues (compounds **8**, **17**–**25**), the introduction of a hydrazine moiety at position 2 leads to an improvement of the IC_50_ compared to compounds bearing a hydrogen bond acceptor such as oxygen at the same position. Interactions mediated by solvent‐exposed portions of a ligand are inherently less stable and less reliably assessed by semi‐flexible docking methods, limiting the ability of docking alone to fully rationalise the activity differences arising from substitutions at position 2. To better understand the binding process of this class of inhibitors, molecular dynamics simulations were performed using SuMD (Supervised Molecular Dynamics), a method designed for studying ligand‐target association processes at the nanosecond timescale, integrating classical molecular dynamics simulations with a tabu‐like algorithm to increase the probability of observing a binding event [28]. Compounds **20** and **14** were selected as a representative pair to interpret, at a molecular level, the role of the hydrogen bond donor capability at position 2 of the thiazole ring, differing solely by the substitution of a nitrogen atom with an oxygen at this position.

For both ligands, SuMD successfully captured a binding event, yielding a final bound pose in agreement with the docking predictions. However, significant differences emerged between the two compounds in both the RMSD profiles and the interaction energy analysis (Figure 5 and Video S2). Compound **20** (IC_50_ = 0.917 µM) displayed a shorter binding trajectory with few transient intermediate states outside the binding site, followed by a well‐defined accommodation phase within the binding cleft. In contrast, **14** (residual kinase activity at 40 µM = 70.71%) required a longer simulation time and exhibited multiple intermediate interaction states, suggesting a more complex and less directed binding process.

**FIGURE 5:**
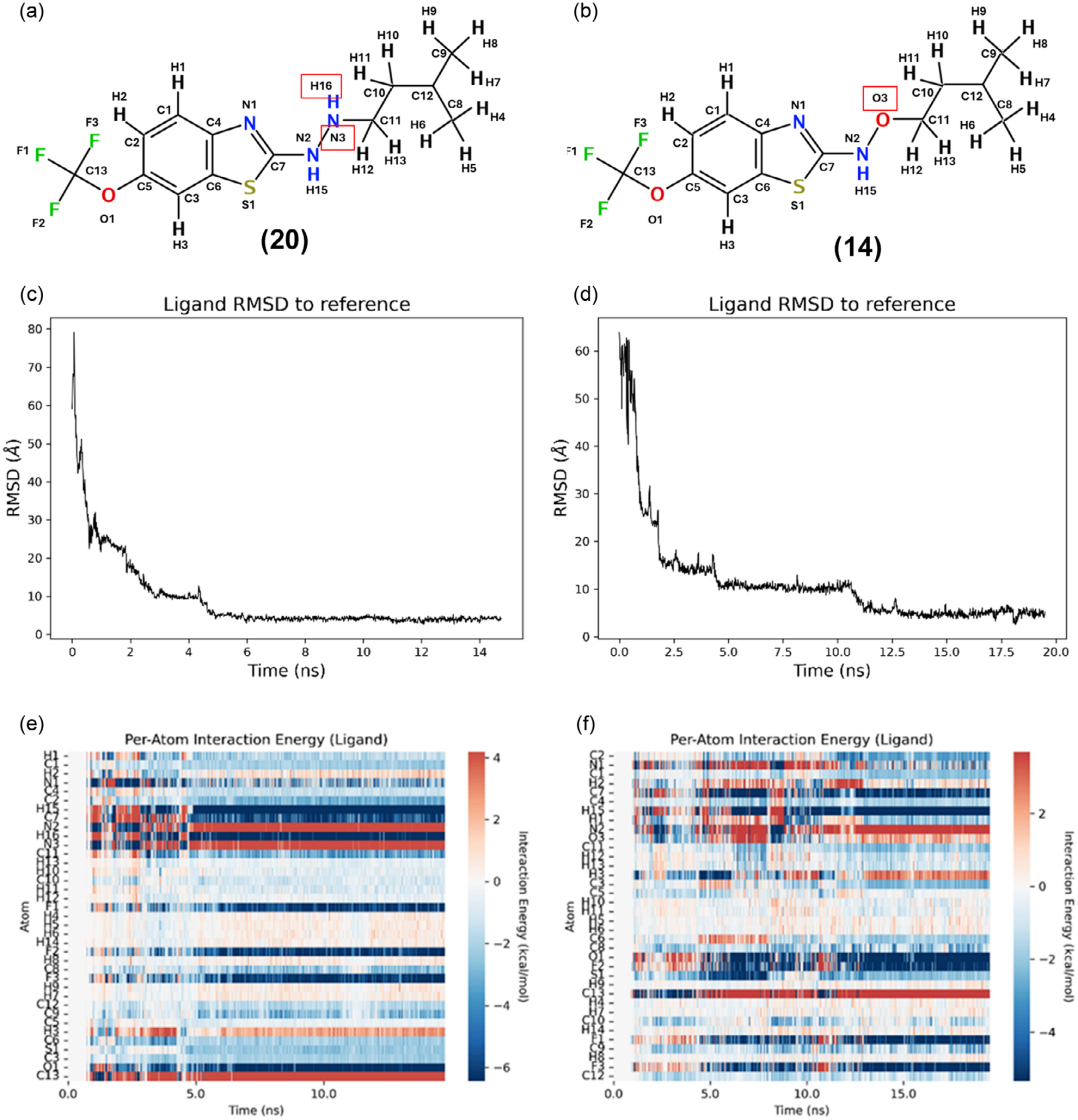
(a,b) Compound **20** and Compound **14**, with atom labels; atoms discussed in the text are highlighted by red squares. (c,d) Ligand RMSD with respect to the reference docking pose as a function of simulation time. (e,f) Per‐atom interaction energy heat maps, reporting the electrostatic interaction energy (kcal/mol) between each ligand atom and CK1*δ* throughout the simulation; favourable (negative) and unfavourable (positive) contributions are shown in blue and red, respectively.

The per‐atom electrostatic interaction energy analysis reveals a key distinction between the two ligands at atom position 3 (Figure 5e,f): in compound **20**, this corresponds to a secondary nitrogen carrying a hydrogen bond donor (NH, numbered N3 and H16), whereas in compound **14** it is an oxygen (atom name O3) acting as a hydrogen bond acceptor. The nitrogen atom (N3), together with the directly bonded hydrogen (H16), contributes a strongly favourable electrostatic energy component, suggesting that this hydrogen facilitates an accessory interaction outside the core binding pocket. In contrast, the oxygen atom (O3) at the equivalent position in compound **14** displays an overall unfavourable electrostatic energy contribution throughout the simulation, indicating that the replacement of nitrogen with oxygen not only abolishes the stabilising interaction observed in compound **20**, but actively introduces an energetically disfavoured contact with the surrounding environment.

The favourable energetic contribution associated with N3 and H16 in compound **20** points to an accessory interaction with a residue outside the core binding pocket. Inspection of the final SuMD frame reveals an additional hydrogen bond between the NH group at position 2 of the thiazole ring and the carbonyl group of Gly86 (Figure 6a), alongside the expected bidentate hydrogen bond with Leu85, a contact that is precluded in compound **14** because of the presence of the oxygen atom at position 3 instead of the NH moiety (Figure 6b). To assess the stability of this interaction over time, a 100 ns classical molecular dynamics simulation was performed in triplicate for both compounds, starting from the respective SuMD final states. The mean per‐residue hydrogen bond frequency, averaged across the three replicates, is reported in Figure 6c. Compound **20** maintains a high hydrogen bond frequency on both Leu85 and Gly86 throughout the simulations, confirming the persistence of this additional contact. In contrast, compound **14** retains the interaction with Leu85 but displays negligible hydrogen bonds with Gly86, consistent with the inability of the oxygen atom at position 3 to act as a hydrogen bond donor.

**FIGURE 6:**
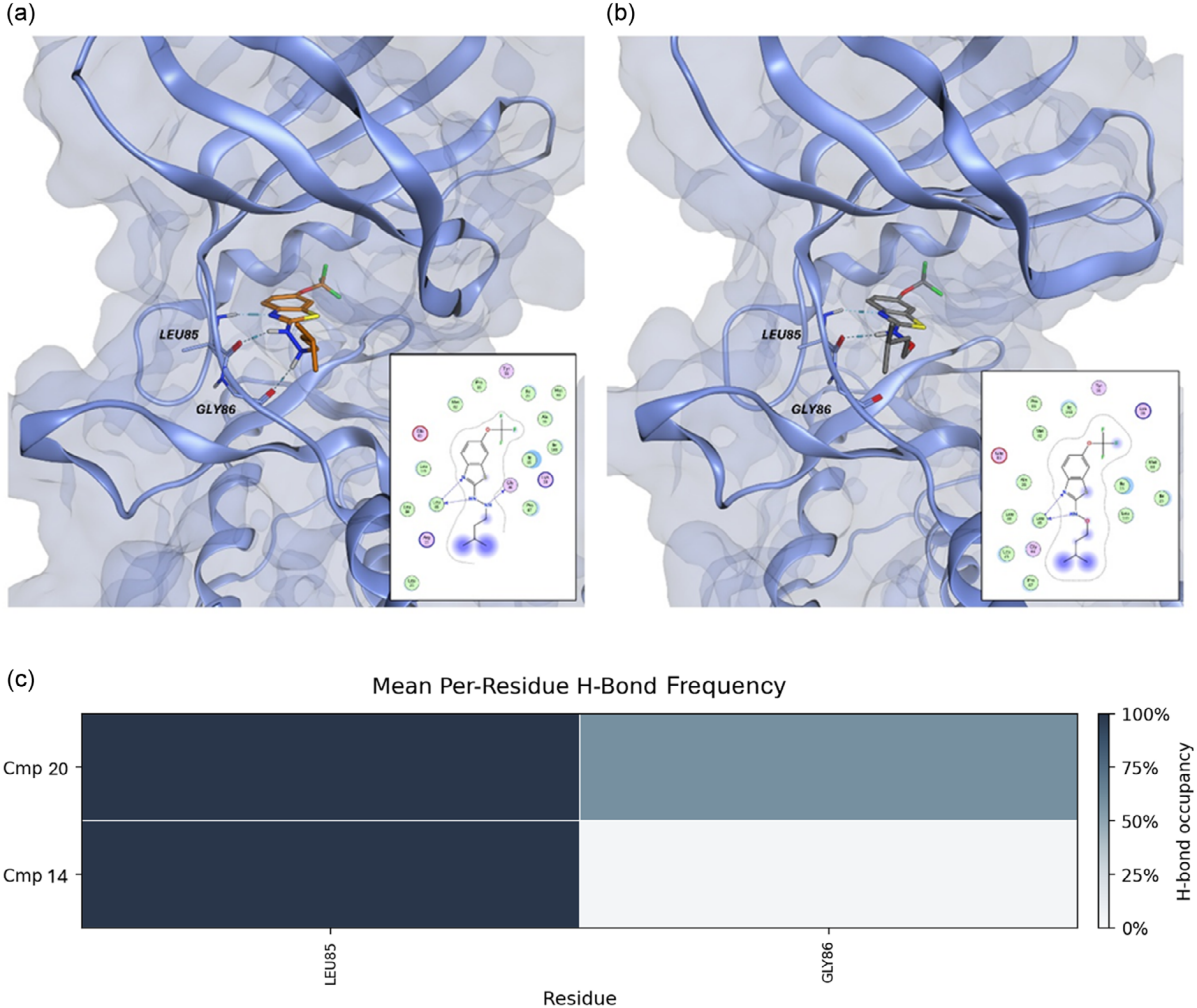
(a, b) Binding mode of compound **20** (a) and **14** (b); protein residues Leu85 and Gly86 are labelled, and hydrogen bond interactions are shown as dashed lines; the 2D interaction diagram is reported in the inset. (c) Mean per‐residue hydrogen bond frequency on Leu85 and Gly86, averaged across three independent 100 ns classical molecular dynamics replicates for both compounds.

The NH group of the hydrazine at position 2 of the thiazole ring therefore plays a key role in stabilising the binding mode by enabling a persistent additional hydrogen bond with Gly86 of the hinge region, a contribution not captured by docking alone.

### 2.5 Physicochemical and Developability Profiling of the Benzo[d]thiazole Series

The metabolism‐based design strategy led from the riluzole hydroxylamine metabolite **2** to the corresponding hydrazine series, which ultimately delivered the most active compounds of the benzo[d]thiazole set. Within this progression, compound **20** emerged as the best derivative, with submicromolar CK1*δ* inhibitory activity, whereas compound **23** represented a second highly promising analogue. The marked gain in potency observed on moving from the hydroxylamine metabolite **2** to the hydrazine derivative **20** raised the question of whether this improvement was accompanied by a more favourable overall profile. For this reason, the developability assessment was centred on the comparison between **2**, as the metabolite‐inspired starting point, and **20**, as the most active compound of the series, while the broader set was used to place this progression in the context of lipophilicity and passive BBB permeability. This comparison was first examined at the level of solution stability. Compound **2**, which already proved challenging during handling, showed clear instability when stored in DMSO at room temperature and monitored by HPLC‐MS over 28 days (Figures S2 and S3). Its purity decreased from 95.7% at the initial analysis (Figure S2) to 81.7% after 1 month (Figure S3), with the concomitant appearance of additional chromatographic peaks and a visible colour change of the solution over time. Under the same conditions, compound **20** proved substantially more robust, with purity decreasing only from 100.0% to 93.8% over the same period (Figures S4 and S5). This result is fully consistent with the progression of the series because the move from the hydroxylamine metabolite **2** to the hydrazine derivative **20** was not associated only with a marked gain in CK1*δ* inhibition but also with a clear improvement in chemical robustness under assay‐relevant conditions. This improved solution stability facilitates the handling and storage of compound **20** relative to hydroxylamine **2**. However, this experiment assesses chemical stability in DMSO and does not provide information regarding metabolic stability, reactive‐metabolite formation, or toxicological safety.

The relationship between CK1*δ* potency and lipophilicity was analysed using lipophilic efficiency, LipE, calculated as pIC_50_ minus clogP (Figure S6), in order to place the SAR outcomes in a developability context. LipE is widely used in medicinal chemistry and kinase inhibitor optimisation because it helps distinguish genuine gains in compound efficiency from apparent potency improvements obtained mainly through increased hydrophobicity [29, 30]. In the present series, the unsubstituted precursors **2** and **8** already occupied a relatively lipophilic region, with clogP values of 2.6 and 2.4, respectively. Functionalisation of the hydrazine nitrogen further shifted the active derivatives into higher lipophilic space, with most analogues showing clogP values between 2.8 and 4.1 and compound **25** reaching 4.4.

The LipE plot showed that the most potent compounds, **20** and **23**, also provided the most useful balance within the active hydrazine subset (Figure 7). This distinction is important because the highest LipE value in the series was not necessarily associated with the highest enzymatic potency. Rather, **20** and **23** emerged as the most convincing compounds because they combined the strongest CK1δ inhibition with an acceptable, although still lipophilic, efficiency profile (Figure 7). Comparison within the arylalkyl subset further showed that increasing hydrophobic extension did not translate into proportional potency gains. Compounds **23**, **24**, and **25** displayed broadly similar CK1*δ* inhibition despite progressively increasing clogP values, suggesting that additional hydrophobic interactions from added methylene units do not enhance binding to the target. Consistently, the more lipophilic and branched 2‐ethylhexyl derivative **21**, as well as the corresponding hydroxylamine **16**, did not retain meaningful activity. This behaviour is consistent with the general principle that continued hydrophobic growth often produces diminishing returns in potency while increasing the risk of poorer developability, a point repeatedly emphasised in LipE‐guided optimisation strategies [40].

**FIGURE 7:**
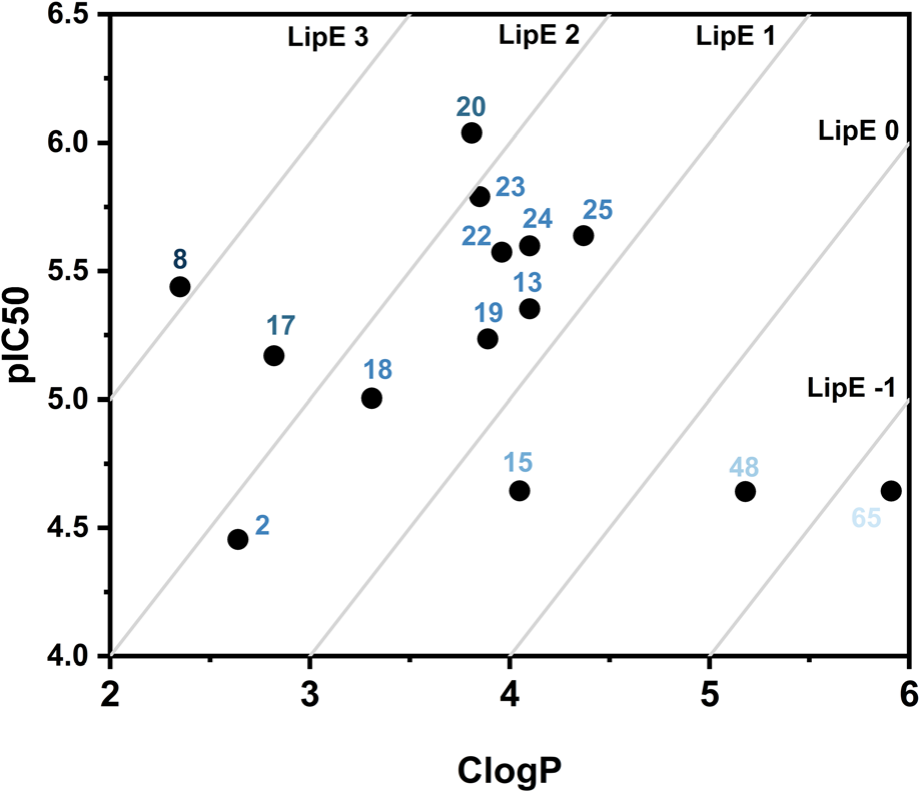
LipE analysis of riluzole‐derived CK1δ inhibitors. Plot of pIC_50_ versus clogP for the active compounds, with diagonal lines indicating constant LipE values. Compounds **20** and **23** showed the most favourable balance between CK1δ potency and lipophilicity, while further hydrophobic extension within the arylalkyl subset did not provide proportional gains in activity.

Overall, LipE analysis supports **20** and **23** as the most balanced compounds of the series while also defining a clear direction for future optimisation. Further improvement should not rely on additional hydrophobic growth but should instead aim to retain the benzo[d]thiazole hydrazine framework while reducing the lipophilic burden, for example, through more polar terminal substituents or small heteroaromatic groups capable of providing specific interactions within the ATP‐binding region.

Passive BBB permeability was evaluated using BBB‐PAMPA to assess whether the compounds retained CNS‐directed permeability after optimisation of the hydrazine series. Under the experimental classification used, all tested riluzole‐derived compounds were classified as CNS+, including the metabolite‐inspired precursors and the most active hydrazine analogues (Figure S8 and Table S1). These data indicate that the potency improvement achieved within the series was not accompanied by a loss of passive BBB permeability.

### 2.6 Biochemical and Cellular Evaluation of the Benzo[d]thiazole Series

The biochemical, biophysical, and cellular evaluation of the most promising benzo[d]thiazole derivatives was carried out to define their mode of inhibition, verify direct interaction with CK1*δ*, obtain a first indication of kinase selectivity, and assess whether the improved enzymatic potency translated into a relevant cellular response.

Compound **20**, the most potent derivative of the series, was selected as an example to clarify the mechanism of inhibition. CK1*δ* activity was measured at increasing ATP concentrations (1, 2, 10, and 50 µM) in the absence and presence of **20** at 1.0 and 2.0 µM, corresponding approximately to onefold and twofold its IC_50_. Lineweaver–Burk analysis showed that all lines converged at a common y‐intercept, with 1/Vmax unchanged at 0.07, whereas the apparent Km increased as the inhibitor concentration increased (Figure 8a). This pattern is consistent with ATP‐competitive inhibition and supports the docking results discussed above, which placed the benzo[d]thiazole scaffold within the adenine‐binding region of CK1*δ*. The behaviour of **20** is therefore consistent with that previously reported for related benzothiazole‐based CK1*δ* inhibitors [1].

**FIGURE 8:**
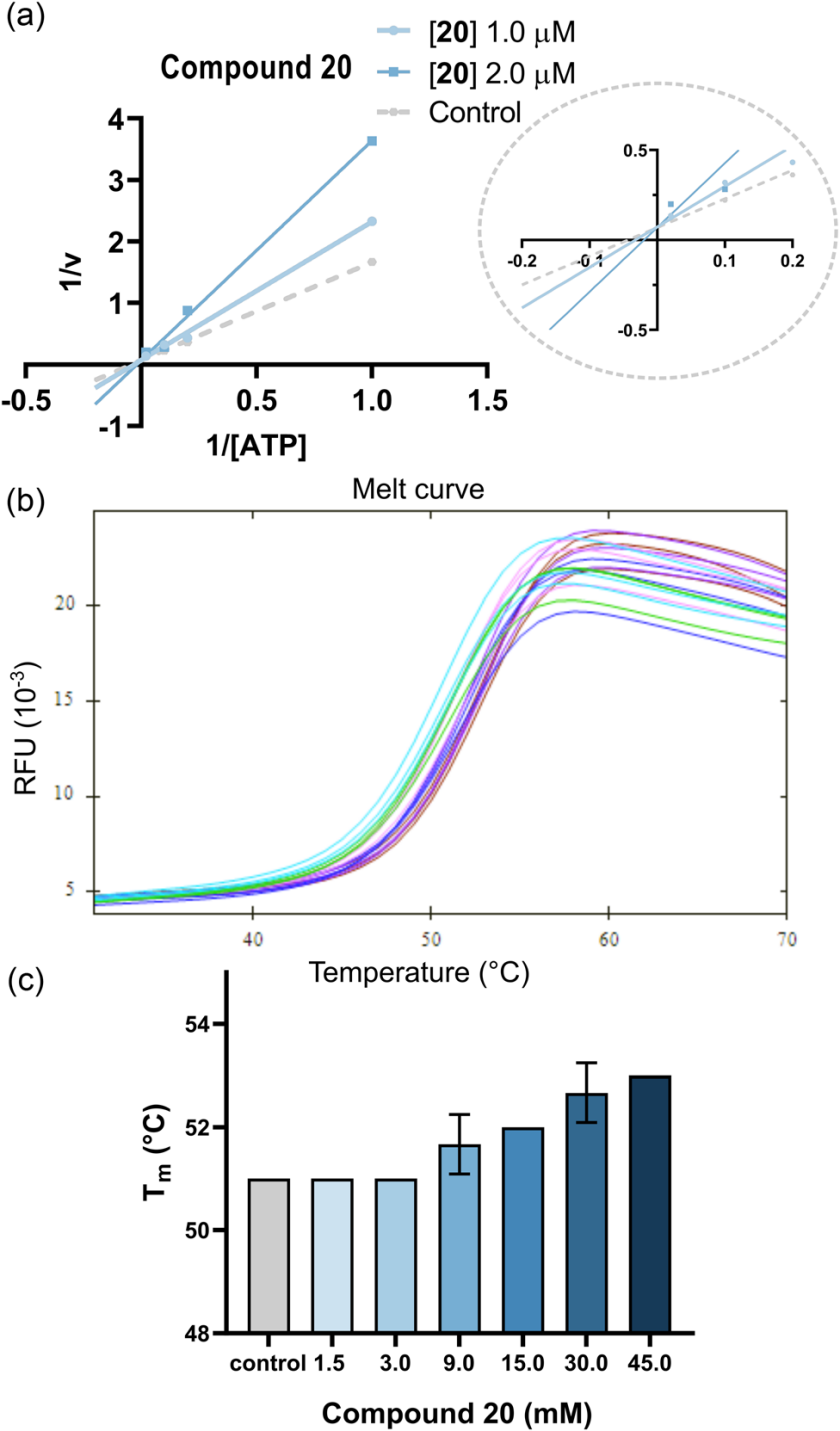
Mode of inhibition and target engagement of compound **20** against CK1δ. (a) Lineweaver–Burk plot obtained by measuring CK1δ activity at increasing ATP concentrations in the absence and presence of **20** at 1.0 and 2.0 µM. The convergence of the lines at a common y intercept is consistent with ATP competitive inhibition. The inset shows a magnification of the intercept region. (b) Thermal shift assay melt curves of recombinant CK1δ incubated with increasing concentrations of **20** and monitored by SYPRO Orange fluorescence. (c) Dose‐dependent *T*_m_ shifts induced by compounds. Values are expressed as the mean of triplicates; error bars indicate standard deviation (SD).

Direct interaction with the target was then evaluated by the thermal shift assay. Recombinant CK1*δ*, residues 1–294, was incubated with increasing concentrations of **20**, from 0.5‐fold to 15‐fold excess relative to the protein concentration of 3 µM, and thermal unfolding was monitored by SYPRO Orange fluorescence (Figure 8b). A concentration‐dependent increase in melting temperature was observed, reaching a maximum *δ* *T*_m_ of +2.0 °C at the highest ligand excess (Figure 8c and Table S2). Although the shift was moderate, this result provides direct biophysical evidence of binding, indicating that compound **20** not only efficiently inhibits CK1*δ* but also stabilises the protein in a dose‐dependent manner.

A preliminary selectivity evaluation for hydroxylamine metabolite **2** and the two most potent hydrazine derivatives, **20** and **23,** was performed against GSK3β, a serine threonine kinase involved in neurodegeneration and belonging to the broader CMGC group. Compound **20** was inactive at 10 µM, with 100% residual activity, whereas compound **23** inhibited GSK3β with an IC_50_ of 16.4 µM, corresponding to an approximately tenfold selectivity window relative to its CK1*δ* IC_50_ of 1.62 µM (Table S3 and Figure S9). By contrast, metabolite **2** showed a GSK3β IC_50_ of 23.1 µM, only slightly higher than its CK1*δ* IC_50_, indicating limited selectivity between the two kinases (Table S3 and Figure S9).

The preliminary selectivity assessment was extended to CK1ε, the closest paralog of CK1*δ*, and p38α, the other two kinases implicated in TDP‐43 phosphorylation. Compounds **20** and **23** inhibited CK1ε with IC_50_ values of 6.20 and 8.53 µM, respectively, compared with their CK1*δ* IC_50_ values of 0.917 and 1.62 µM. These results correspond to approximately 6.8‐fold and 5.3‐fold preferences for CK1*δ*, demonstrating a measurable, although moderate, differentiation between the two closely related isoforms (Table S4 and Figure S10). Conversely, they do not inhibit p38α activity by more than 50% when tested at a concentration of 50 µM (Table S4).

Taken together, these results indicate that substitution of the terminal hydrazine nitrogen improved the preliminary kinase discrimination of the series, with compound **20** showing the most favourable profile among the derivatives examined. The cellular profile of riluzole, metabolite **2**, and the most promising hydrazine derivatives was assessed in SH SY5Y human neuroblastoma cells. Cell viability was first measured by the MTT assay after 24 h of exposure at 5 and 10 µM (Figure S11). Riluzole, **20**, and **23** were all well tolerated at 5 µM, with viability values of 94.9%, 93.2%, and 103.5%, respectively. At 10 µM, compound **20** caused a modest but significant reduction in viability to 77.3%, whereas riluzole and **23** remained better tolerated, with viability values of 87.9% and 92.0%, respectively. A markedly different profile was observed for metabolite **2**, which was clearly cytotoxic already at 5 µM, reducing cell viability to 61.0% and even more strongly at 10 µM, where viability dropped to 47.0% (Figure S11). This result is particularly relevant because **2** is the major phase I metabolite of riluzole. The improved viability observed for **20** and **23** indicates that replacement of the hydroxylamine with a hydrazine improved cellular tolerance under the conditions tested. Nevertheless, hydrazines are recognised structural alerts, and related 1‐aryl‐2‐monoalkylhydrazines have been identified as mutagenicity‐associated toxicophores because of their potential metabolic activation to reactive intermediates. Importantly, the presence of a structural alert does not by itself demonstrate that a compound is toxic, as its relevance depends on the complete molecular structure, metabolic fate, and level of exposure [41, 42]. Recent evidence that substituted hydrazines can interact covalently with cofactor‐dependent enzymes nevertheless supports further investigation of this motif [43]. Thus, the present results identify **20** and **23** as promising CK1*δ* inhibitors with passive BBB permeability, while additional metabolic and toxicological profiling will be required to establish their suitability for CNS applications.

The compounds that were sufficiently well tolerated were then evaluated in a glutathione‐depletion model of oxidative stress. SH‐SY5Y cells were pretreated with the test compounds for 1 h before exposure to ethacrynic acid (45 µM), a thiol‐reactive agent that depletes intracellular glutathione and induces pathological changes associated with TDP‐43 proteinopathy. Under these conditions, neither riluzole at 10 µM nor compounds **20** and **23**, both tested at 5 µM, produced a statistically significant rescue of cell viability relative to the ethacrynic acid‐treated control (Figure 9). Compound **20** showed a higher mean cell viability than compound **23**, but this difference did not reach statistical significance across independent experiments. Overall, none of the tested compounds demonstrated significant neuroprotective activity under the conditions examined.

**FIGURE 9:**
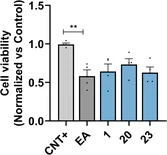
Assessment of neuroprotective activity of riluzole (**1**), compound **20**, and compound **23** in an ethacrynic acid‐induced oxidative stress model. SH SY5Y cells were pretreated with the test compounds for 1 h before exposure to ethacrynic acid (EA), 45 µM. **1** was tested at 10 µM, whereas compounds **20** and **23** were tested at 5 µM. Cell viability is reported as relative intensity. Data are expressed as mean ± SEM from three independent experiments, each performed using the mean of eight wells per condition. **p < 0.01 versus ethacrynic acid treated cells.

Overall, the data identify the 2‐hydrazineyl‐6‐(trifluoromethoxy)benzo[d]thiazole derivatives as genuine ATP‐competitive CK1*δ* inhibitors with direct target engagement, a measurable preference over CK1ε and favourable preliminary discrimination against GSK3β. Among them, compound **20** emerged as the most active analogue, because it combined submicromolar CK1*δ* inhibition, measurable stabilisation of the target protein, no detectable inhibition of GSK3β at 10 µM, and better short‐term cellular tolerance than hydroxylamine **2** at the concentrations tested, although its hydrazine‐associated liabilities remain unresolved. The absence of neuroprotection in the ethacrynic acid assay does not diminish the value of this series. Rather, these results reinforce the meaning of this series as a set of CK1*δ* inhibitors and support their use as a starting point for developing more potent compounds to serve as chemical tools to examine the specific contribution of CK1*δ*, alongside the broader pharmacology of riluzole.

## 3 Conclusion

This work establishes riluzole metabolism as a productive entry point for the design of CK1*δ* inhibitors based on the 6‐(trifluoromethoxy)benzo[d]thiazole scaffold. The major N‐hydroxylamine metabolite of riluzole (**2**) retained micromolar activity against CK1*δ*, supporting the view that this metabolic transformation preserves the recognition of a kinase directly connected to TDP‐43 pathology. At the same time, its limited chemical stability and marked toxicity in SH‐SY5Y cells identified the hydroxylamine group as a liability rather than a suitable motif for further development.

Replacement of the hydroxylamine functionality with a hydrazine linker converted this metabolite‐inspired starting point into a more active and chemically robust CK1*δ*‐directed chemotype. The parent hydrazine already produced an approximately tenfold improvement in potency, and subsequent N‐substitution defined a clear SAR in which medium‐sized branched alkyl and benzyl substituents were preferred. Within this series, compound **20** emerged as the most effective analogue, combining submicromolar CK1*δ* inhibition with improved stability, maintained passive BBB permeability, ATP‐competitive behaviour, direct target engagement by the thermal shift assay, an approximately sevenfold preference over CK1ε, and no detectable inhibition of GSK3β and p38α at the screening concentration. Docking and SuMD simulations highlighted how a hydrogen bond donor at position 2 of the thiazole ring, exemplified by hydrazinic compound **20**, enables an additional stabilising interaction with Gly86, rationalising its improved potency and revealing a dynamic contribution not captured by docking alone.

The cellular data further clarified the value and the limits of this scaffold. The hydrazine derivatives were better tolerated than the hydroxylamine metabolite in the 24 h SH‐SY5Y assay. This result demonstrates improved short‐term cellular tolerance under the conditions tested but does not resolve the metabolic, genotoxic, or off‐target liabilities associated with the hydrazine structural alert. However, compounds **20** and **23** did not rescue the neurotoxic effect in the ethacrynic acid cell model, possibly indicating that they are not yet sufficiently potent CK1*δ* inhibitors to confer cellular protection under these conditions. This result defines the role of the series more precisely. These compounds are not direct functional mimics of riluzole but CK1*δ*‐focused benzo[d]thiazole tools derived from its metabolic profile.

In conclusion, this study connects riluzole metabolism, CK1*δ* inhibition, and benzo[d]thiazole medicinal chemistry into a single design framework. The identification of compound **20** shows that metabolite‐inspired modification can deliver more potent and selective CK1*δ* inhibitors while preserving CNS‐compatible passive permeability. Future work will focus on reducing the lipophilic burden of this scaffold while retaining the hydrazine‐based recognition pattern, with the aim of developing more potent and better‐balanced CK1*δ* chemical tools for investigating CK1*δ* role in ALS.

## 4 Experimental Part

### 4.1 General Chemistry

Reagents were obtained from commercial suppliers and used without further purification. Commercially available racemic 1‐bromo‐2‐ethylhexane was used; consequently, all 2‐ethylhexyl derivatives were obtained and tested as racemic mixtures. Reactions were monitored by TLC on precoated silica gel plates (Macherey‐Nagel, 60FUV254). Final compounds and intermediates were purified by flash chromatography using silica gel as the stationary phase (Macherey‐Nagel, silica 60, 240–400 mesh) or preparative TLC (stationary phase silica gel, Mechery‐Nagel, silica 60, 240–400 mesh, 0.25 mm). When used, light petroleum ether refers to the fractions boiling at 40–60 °C. Melting points were determined with a Stuart SMP10 melting point apparatus, and they were not corrected. The ^1^H NMR, ^13^C NMR, and gHSQCAD bidimensional spectra were determined in CDCl_3_ or DMSO‐d_6_ and recorded on Varian 400 MHz or Varian 500 MHz spectrometers; chemical shifts (*δ* scale) are reported in parts per million (ppm) and referenced to the residual solvent peak, with splitting patterns abbreviated to: s (singlet), d (doublet), dd (doublet of doublets), dt (doublet of triplets), t (triplet), m (multiplet) and bs (broad signal). Coupling constants (J) are given in Hz. Electrospray ionization mass spectra (ESI‐MS) were collected using a ESI Bruker 4000 Esquire spectrometer. High‐resolution mass spectra (HRMS) for accurate mass determination were recorded on a Bruker microTOF‐Q – instrument. Compound purities were determined by HPLC using the instrument SHIMADZU CBM‐20A, column Gemini 5 μm NX‐C18 (Phenomenex). Elution gradient was performed for 30 min at the flow of 1 mL/min from water‐methanol 55:45 to 15:85. The detector was set to 254 nm. Stability tests were conducted by HPLC‐MS using the instrument Bruker micrOTOF‐Q coupled with HPLC Agilent 1260 Infinity II (procedures are described in the dedicated following section).

#### 4.1.1 Synthesis of N‐Substituted‐6‐(trifluoromethoxy)benzo[*d*]thiazol‐2‐amine Derivatives (2, 8–10, 22)

##### 4.1.1.1 Synthesis of N‐(6‐(trifluoromethoxy)benzo[*d*]thiazol‐2‐yl)hydroxylamine Hydrochloride (2)

A solution of 85 mg (1.22 mmol) of hydroxylamine hydrochloride, commercially available, and 71 mg (0.512 mmol) of K_2_CO_3_ was refluxed under argon atmosphere for 30 min. Once completed, 50 mg (0.197 mmol) of 2‐chloro‐6‐(trifluoromethoxy)benzo[d]thiazole (**26**), commercially available, was added, and the mixture was stirred at 65 °C for 3 h. The mixture was then filtered to eliminate the base, and the solvent was removed under reduced pressure. The crude product was purified with preparative TLC (light petroleum 60% – EtOAc 40%), washed with EtOAc 90% – MeOH 10%, and stirred with hydrogen chloride in 1,4‐dioxane for 30 min. The solvent was removed under reduced pressure, and the crude was filtered with EtOAc to give 22.0 mg of white solid. Yield 39%; mp 176 °C d. ^1^H NMR (499 MHz, DMSO‐d_6_) *δ* 10.72 (s, 1H), 9.89 (d, *J* = 28.2 Hz, 1H), 7.71 (s, 1H), 7.26–7.07 (m, 1H), 6.95 (d, *J* = 7.2 Hz, 2H). ^13^C NMR (101 MHz, DMSO‐d_6_) *δ* 173.86 (d, *J* = 4.2 Hz), 158.42, 154.42 (d, *J* = 1.4 Hz), 137.02, 135.89, 120.28 (s, *J* = 42.4, 33.0 Hz), 116.13, 109.56 (d, *J* = 1.7 Hz). ES‐MS (methanol) *m*/*z*: 250.9 [M + H]^+^, 249.2 [M − H]^+^. HRMS (ESI‐TOF) *m*/*z*: C_8_H_5_F_3_N_2_O_2_S, C_8_H_5_F_3_N_2_O_2_S experimental 251.0095 [M + H]^+^, theoretical 251.0097 [M + H]^+^, Δ = 0.0002. Purity HPLC: 99.2%.

##### 4.1.1.2 Synthesis of 2‐Hydrazineyl‐6‐(trifluoromethoxy)benzo[*d*]thiazole (8)

To a solution of 0.500 mL (10.0 mmol) of hydrazine monohydrate in 3.00 mL of methanol, 100 mg (0.394 mmol) of 2‐chloro‐6‐(trifluoromethoxy)benzo[d]thiazole (**26**), commercially available, was added. The mixture was refluxed for 2 h. The solvent was then removed under reduced pressure, and the residue was purified with flash chromatography (light petroleum 70% – EtOAc 30%) to give 42.0 mg of white solid. Yield 42%; mp 202 °C. ^1^H NMR (400 MHz, DMSO‐d_6_) *δ* 9.20 (s, 1H), 7.78 (d, *J* = 2.4 Hz, 1H), 7.34 (d, *J* = 8.7 Hz, 1H), 7.27–7.09 (m, 1H), 5.11 (s, 2H). ^13^C NMR (101 MHz, DMSO‐d_6_) *δ* 175.15, 152.72, 141.61 (q, *J* = 1.8 Hz), 131.43, 120.30 (q, *J* = 255.1 Hz), 118.83 (CH), 118.10 (CH), 114.46 (CH). ES‐MS (methanol) *m/z*: 250.0 [M + H]^+^. HRMS (ESI‐TOF) *m*/*z*: C_8_H_6_F_3_N_3_OS experimental 250.0258 [M + H]^+^, theoretical 250.0256 [M + H]^+^, Δ = 0.0002. Purity HPLC: 98.4%.

##### 4.1.1.3 Synthesis of N,O‐dimethyl‐N‐(6‐(trifluoromethoxy)benzo[*d*]thiazol‐2‐yl)hydroxylamine (9)

To a solution of 100 mg (0.394 mmol) of 2‐chloro‐6‐(trifluoromethoxy)benzo[d]thiazole (**26**), commercially available, in 10 mL of butanol, 154 mg (1.58 mmol) of N‐methyl methoxylamine and 0.274 mL (1.97 mmol) of triethylamine were added, and the mixture was refluxed overnight. Then, the crude product was concentrated under reduced pressure and purified with flash chromatography (light petroleum 95% – EtOAc 5%) to give 63.0 mg of white solid. Yield 58%; mp 53 °C. ^1^H NMR (400 MHz, DMSO‐d_6_) *δ* 8.02 (dd, *J* = 2.3, 0.8 Hz, 1H), 7.68 (d, *J* = 8.8 Hz, 1H), 7.39–7.27 (m, 1H), 3.82 (s, 3H), 3.35 (s, 3H). ^13^C NMR (101 MHz, DMSO‐d_6_) *δ* 173.30, 151.06, 143.16 (d, J = 1.9 Hz), 132.78, 120.77 (CH), 120.21 (q, *J* = 255.8 Hz), 119.71 (CH), 115.12 (CH), 61.54 (CH_3_), 39.01 (CH_3_). ES‐MS (methanol) *m/z*: 279.0 [M + H]^+^. HRMS (ESI‐TOF) *m/z*: C_10_H_9_F_3_N_2_O_2_S experimental 279.0410 [M + H]^+^, theoretical 279.0410 [M + H]^+^, Δ = 0.0000. Purity HPLC: 99.9%.

##### 4.1.1.4 Synthesis of N‐methyl‐6‐(trifluoromethoxy)benzo[*d*]thiazol‐2‐amine (10)

In a microwave test tube, 100 mg (0.394 mmol) of 2‐chloro‐6‐(trifluoromethoxy)benzo[d]thiazole (**26**), commercially available, 107 mg of methylamine hydrochloride (1.58 mmol), and 0.274 ml of triethylamine (1.97 mmol) were suspended in 2.0 mL of butanol. The mixture was stirred in the microwave reactor at 150 °C for 1 h. Once completed, the solvent was removed under reduced pressure, and the residue was purified by flash chromatography (Light petroleum 60% – EtOAc 40%). The product was then filtered with EtOEt, collecting 32.0 mg of white solid. Yield 33%; mp 112 °C. ^1^H NMR (400 MHz, DMSO‐d_6_) *δ* 8.09 (d, *J* = 4.7 Hz, 1H), 7.78 (d, *J* = 2.0 Hz, 1H), 7.42 (d, *J* = 8.7 Hz, 1H), 7.19 (dd, *J* = 8.7, 2.0 Hz, 1H), 2.94 (d, *J* = 4.7 Hz, 3H). ^13^C NMR (101 MHz, DMSO‐d_6_) *δ* 167.96, 151.85, 142.08, 131.41, 120.27 (q, *J* = 255.2 Hz), 119.06 (CH), 118.19 (CH), 114.50 (CH), 30.52 (CH_3_). ES‐MS (methanol) *m/z*: 249.0 [M + H]^+^. HRMS (ESI‐TOF) *m/z*: C_9_H_7_F_3_N_2_OS experimental 249.0305 [M + H]^+^, theoretical 249.0304 [M + H]^+^, Δ = 0.0001. Purity HPLC: 99.3%.

##### 4.1.1.5 Synthesis of 2‐(2‐phenylhydrazineyl)‐6‐(trifluoromethoxy)benzo[*d*]thiazole Hydrochloride (22)

To a solution of 150 mg (0.590 mmol) of 2‐chloro‐6‐(trifluoromethoxy)benzo[d]thiazole (**26**), commercially available, in 7.5 mL of butanol, 145 μL (132 mg, 1.211 mmol) of phenylhydrazine was added. The reaction was refluxed under an argon atmosphere for 3 h; once completed, the solvent was removed under reduced pressure, and the crude product was purified with flash chromatography (light petroleum 90% – EtOAc 10%). The hydrochloride salt of the compound was then formed by adding hydrochloride acid in 1,4‐dioxane at room temperature for 30 min. The solvent was then removed under vacuum, and the crude product was then filtered using EtOAc to provide 21.0 mg of white compound. Yield 10%; mp 134 °C. ^1^H NMR (400 MHz, DMSO‐d_6_) *δ* 9.78 (s, 1H), 7.83 (d, *J* = 1.3 Hz, 1H), 7.81–7.75 (m, 1H), 7.45 (dd, *J* = 5.1, 2.1 Hz, 1H), 7.21–7.11 (m, 5H), 3.99 (s, 1H). ^13^C NMR (101 MHz, DMSO‐d_6_) *δ* 186.13, 169.80, 158.27, 143.26, 128.84, 128.72 (d, *J* = 1.5 Hz), 126.56, 126.41, 119.41, 118.78, 111.40, 110.00 (d, *J* = 1.3 Hz), 102.66. ES‐MS (methanol) *m/z*: 326.0 [M + H]^+^. HRMS (ESI‐TOF) *m/z*: C_14_H_10_F_3_N_3_OS experimental 326.0568 [M + H]^+^, theoretical 326.0569 [M + H]^+^, Δ = 0.0001. Purity HPLC: 93.5%.

#### 4.1.2 Synthesis of O‐Substituted‐N‐(6‐(trifluoromethoxy)benzo[*d*]thiazol‐2‐yl)hydroxylamine Hydrochloride Derivatives (11–16)

##### 4.1.2.1 General Procedure for the Synthesis of 2‐(alkyloxy)isoindoline‐1,3‐dione (28–31)

To a solution of 4.00 g (24.5 mmol) of N‐hydroxy phthalimide (**27**), commercially available, and 1.5 equivalents of the desired 1‐bromoalcane in 100 mL of anhydrous DMF, 8.10 g (58.8 mmol) of K_2_CO_3_ was added under an argon atmosphere. The mixture was stirred at room temperature for 48 h. Once completed, the reaction was quenched dropwise at 0 °C using 60.0 mL of saturated solution of Na_2_CO_3,_ and the mixture was extracted with DCM (90.0 mL × 3). The organic layer was then washed with 90.0 mL of NaHCO_3_, 90.0 mL of H_2_O, and 90.0 mL of brine and finally dried over anhydrous sodium sulphate.

###### 4.1.2.1.1 2‐Propoxyisoindoline‐1,3‐dione (28)

Yield 60% (3.00 g). White solid; mp 76 °C. ^1^H NMR (499 MHz, DMSO‐d_6_) *δ* 7.86 (s, 4H), 4.09 (t, *J* = 6.6 Hz, 2H), 1.74–1.63 (m, 2H), 0.99 (t, *J* = 7.4 Hz, 3H). ESI‐MS (methanol) *m/z* C_11_H_11_NO_3_: 206.0 [M + H]^+^, 228.0 [M + Na]^+^.

###### 4.1.2.1.2 2‐(Pentyloxy)isoindoline‐1,3‐dione (29)

Yield 94% (2.70 g from 2.00 g of starting material). White solid; mp 40 °C. ^1^H NMR (400 MHz, CDCl_3_) *δ* 7.83 (td, *J* = 5.2, 2.0 Hz, 2H), 7.75 (td, *J* = 5.2, 2.0 Hz, 2H), 4.20 (t, *J* = 6.8 Hz, 2H), 1.90–1.71 (m, 2H), 1.52–1.30 (m, 4H), 0.93 (t, *J* = 7.2 Hz, 3H).

###### 4.1.2.1.3 2‐(Isopentyloxy)isoindoline‐1,3‐dione (30)

Yield 74% (4.00 g). White solid; mp 47 °C. ^1^H NMR (400 MHz, DMSO‐d_6_) *δ* 7.85 (s, 4H), 4.16 (t, *J* = 6.6 Hz, 2H), 1.81 (dt, *J* = 13.5, 6.7 Hz, 1H), 1.57 (q, *J* = 6.7 Hz, 2H), 0.92 (d, *J* = 6.7 Hz, 6H). ESI‐MS (methanol) *m/z* C_13_H_15_NO_3_: 234.0 [M + H]^*+*^.

###### 4.1.2.1.4 2‐((2‐Ethylhexyl)oxy)isoindoline‐1,3‐dione (31)

Yield 70% (1.93 g). Transparent liquid. ^1^H NMR (400 MHz, DMSO‐d_6_) *δ* 7.85 (s, 4H), 4.04 (d, *J* = 5.6 Hz, 2H), 3.55 (dd, *J* = 4.7, 1.4 Hz, 2H), 1.70–1.59 (m, 1H), 1.54–1.39 (m, 4H), 0.89 (dt, *J* = 10.0, 5.3 Hz, 8H).

##### 4.1.2.2 General Procedure for the Synthesis of O‐alkylhydrazine Hydrochloride (33–35, 37)

1 equivalent (10 mmol) of 2‐(alkyloxy)isoindoline‐1,3‐dione (**28–31**) was solved in 10 mL of THF. 3 equivalents (30 mmol) of monohydrate hydrazine were added dropwise to the mixture, and the reaction was covered and stirred at room temperature overnight. Once completed, the mixture was filtered and washed with THF and with 60 mL of NaOH 0.1 M and extracted with 30 mL of EtOAc (2 × 30 mL). The organic layer was dried over anhydrous sodium sulphate, and the resulting mixture was acidified with 25 mL of HCl in 1,4‐dioxane for 5 min. The solvent was removed under reduced pressure, and the resulting crude product was filtered with diethyl ether at 0 °C to collect white solid compounds **33–35**, **37**.

###### 4.1.2.2.1 O‐Propylhydroxylamine Hydrochloride (33)

Yield 66% (1.05 g). White solid; mp 127 °C. ^1^H NMR (400 MHz, DMSO‐d_6_) *δ* 11.03 (s, 3H), 3.96 (t, *J* = 6.5 Hz, 2H), 1.66–1.52 (m, 2H), 0.88 (t, *J* = 7.4 Hz, 3H). ESI‐MS (methanol) *m/z* C_3_H_9_NO: 76.1 [M + H]^+^, 113.9 [M + K]^+^.

###### 4.1.2.2.2 O‐Pentylhydroxylamine Hydrochloride (34)

Yield 46% (741 mg). White solid; mp 139 °C. ^1^H NMR (400 MHz, DMSO‐d_6_) *δ* 11.08 (bs, 2H), 4.00 (t, *J* = 6.5 Hz, 2H), 1.72–1.47 (m, 2H), 1.37–1.23 (m, 4H), 0.86 (t, *J* = 6.9 Hz, 3H).

###### 4.1.2.2.3 O‐Isopentylhydroxylamine Hydrochloride (35)

Yield 67% (1.63 g). White solid; mp 121 °C. ^1^H NMR (400 MHz, DMSO‐d_6_) *δ* 10.82 (s, 3H), 4.00 (t, *J* = 6.6 Hz, 2H), 1.64 (dt, *J* = 13.5, 6.7 Hz, 1H), 1.45 (q, *J* = 6.7 Hz, 2H), 0.88 (d, *J* = 6.6 Hz, 6H). ESI‐MS (methanol) *m/z* C_5_H_13_NO: 104.0 [M + H]^+^.

###### 4.1.2.2.4 O‐(2‐Ethylhexyl)hydroxylamine Hydrochloride (37)

Yield 67% (740 mg). White solid; mp 138 °C. ^1^H NMR (400 MHz, DMSO‐d_6_) *δ* 10.97 (s, 3H), 3.92 (d, *J* = 5.7 Hz, 2H), 1.37–1.17 (m, 9H), 0.92–0.79 (m, 6H). ESI‐MS (methanol) *m*/*z* C_8_H_19_NO: 146.0 [M + H]^+^.

##### 4.1.2.3 General Procedure for the Synthesis of *Tert*‐butyl Alkyloxycarbamate (38–43)

1 equivalent of synthesised O‐alkylhydrazine hydrochloride (**33–35, 37**) or commercially available **32**, **36** was solved in CH_3_CN (0.75 mL/mmol), and 1.1 equivalents of Et_3_N was added to the mixture that was stirred at room temperature for 1.5 h. Once completed, the reaction was filtered and washed with CH_3_CN, and the resulting solution was cooled at 0 °C. To this mixture, a solution of 2 equivalents of Boc_2_O in CH_3_CN (3 mL/mmol) was added dropwise at 0 °C. The reaction was then stirred overnight at room temperature. Once completed, the solvent was removed under reduced pressure, and the crude product was solved in 75 mL of DCM and washed with water. The organic layer was dried over anhydrous sodium sulphate, and the solvent was eliminated under vacuum to collect a white solid.

###### 4.1.2.3.1 *Tert*‐butyl Methoxycarbamate (38)

Yield 51% (1.80 g). White solid; mp 63 °C. ^1^H NMR (400 MHz, CDCl_3_) *δ* 7.18 (s, 1H), 3.71 (s, 3H), 1.48 (s, 9H).

###### 4.1.2.3.2 *Tert*‐butyl Propoxycarbamate (39)

Yield 96% (1.50 g). White solid; mp 49 °C. ^1^H NMR (400 MHz, DMSO‐d_6_) *δ* 9.89 (s, ^1^H), 3.63 (t, *J* = 6.6 Hz, 2H), 1.51 (dd, *J* = 14.2, 6.8 Hz, 2H), 1.40 (s, 9H), 0.88 (t, *J* = 7.4 Hz, 3H). ESI‐MS (methanol) *m/z* C_8_H_17_NO_3_: 176.1 [M + H]^+^.

###### 4.1.2.3.3 *Tert*‐butyl (pentyloxy)carbamate (40)

Yield 58% (612 mg). White solid; mp 56 °C. ^1^H NMR (400 MHz, CDCl_3_) *δ* 7.12 (s, 1H), 3.83 (t, *J* = 6.7 Hz, 2H), 1.66–1.53 (m, 2H), 1.47 (s, 9H), 1.41–1.28 (m, 4H), 0.98–0.81 (m, 3H).

###### 4.1.2.3.4 *Tert*‐butyl (isopentyloxy)carbamate (41)

Yield 95% (1.40 g). White solid; mp 67 °C. ^1^H NMR (499 MHz, DMSO‐d_6_) *δ* 9.88 (s, 1H), 3.70 (t, *J* = 6.6 Hz, 2H), 1.67 (dt, *J* = 13.4, 6.7 Hz, 1H), 1.47 (m, 11H), 0.87 (d, *J* = 6.7 Hz, 6H). ESI‐MS (methanol) *m/z* C_10_H_21_NO_3_: 226.0 [M + Na]^+^.

###### 4.1.2.3.5 *Tert*‐butyl (benzyloxy)carbamate (42)

Yield 34% (788 mg). White solid; mp 48 °C. ^1^H NMR (400 MHz, CDCl_3_) *δ* 7.42–7.32 (m, 5H), 7.07 (s, 1H), 4.86 (s, 2H), 1.48 (s, 9H).

###### 4.1.2.3.6 *Tert*‐butyl ((2‐ethylhexyl)oxy)carbamate (43)

Yield 82% (821 mg). White solid; mp 74 °C. ^1^H NMR (400 MHz, DMSO‐d_6_) *δ* 9.85 (s, 1H), 3.56 (d, J = 5.8 Hz, 2H), 1.46 (s, 2H), 1.45 (s, 9H), 1.28 (ddd, J = 27.8, 16.1, 6.0 Hz, 7H), 0.88–0.80 (m, 6H). ESI‐MS (methanol) *m/z* C_13_H_27_NO_3_: 268.1 [M + Na]^+^, 284.1 [M + K]^+^.

##### 4.1.2.4 General Procedure for Synthesis of *Tert*‐butyl Alkyloxy(6‐(trifluoromethoxy)benzo[d]thiazol‐2‐yl)carbamate (44–49)

Under argon atmosphere, 0.15 equivalents of Pd_2_(dba)_3_ and 0.20 equivalents of DPPF were added to 15 mL of anhydrous CH_3_CN. The mixture was heated at 40 °C. Then, 2.0 equivalents of *tert*‐butyl alkyloxycarbamate (**38–43**) were added to the reaction. After 10 min, 1.0 equivalent of 2‐chloro‐6‐(trifluoromethoxy)benzo[d]thiazole, commercially available, and 1.6 equivalents of Cs_2_CO_3_ were added, and the mixture was stirred at 40 °C overnight. Once completed, the catalyst was removed by filtration through a Celite pad. The filtered solution was then washed with brine (2 × 20 mL) and dried over anhydrous sodium sulphate. The solvent was removed under reduced pressure, and the crude was purified with flash chromatography (light petroleum 95% – EtOAc 5%) to obtain a pale yellow solid.

###### 4.1.2.4.1 *Tert*‐butyl methoxy(6‐(trifluoromethoxy)benzo[*d*]thiazol‐2‐yl)carbamate (44)

Yield 23% (98.3 mg). Pale yellow solid; mp 56 °C. ^1^H NMR (400 MHz, CDCl_3_) *δ* 7.87 (d, *J* = 8.9 Hz, 1H), 7.61 (d, *J* = 1.5 Hz, 1H), 7.30–7.26 (m, 1H), 4.04 (s, 3H), 1.65 (s, 9H).

###### 4.1.2.4.2 O‐Propyl‐N‐(6‐(trifluoromethoxy)benzo[*d*]thiazol‐2‐yl)hydroxylamine (45)

Preparative TLC was performed after flash chromatography (light petroleum – EtOAc 95:5). Yield 4% (15.0 mg). HRMS (ESI‐TOF) *m/z*: C_11_H_11_F_3_N_2_O_2_S experimental 293.0560 [M + H]^+^, theoretical 293.0566 [M + H]^+^, Δ = 0.0006. The compound was treated with HCl in situ without ^1^H and ^13^C characterisation because of its instability.

###### 4.1.2.4.3 *Tert*‐butyl (pentyloxy)(6‐(trifluoromethoxy)benzo[*d*]thiazol‐2‐yl)carbamate (46)

Yield 19% (122 mg). Pale yellow solid; mp 77 °C. ^1^H NMR (400 MHz, DMSO‐*d*_6_) *δ* 7.79 (d, *J* = 8.8 Hz, 1H), 7.60–7.51 (m, 2H), 7.41 (dd, *J* = 8.6, 2.9 Hz, 1H), 4.09 (s, 3H), 1.57 (s, 9H), 1.48–1.27 (m, 7H), 1.21 (s, 1H), 0.95 (t, *J* = 7.4 Hz, 3H), 0.89 (t, *J* = 7.0 Hz, 3H). ESI‐MS (metanolo) *m/z* C_18_H_23_F_3_N_2_O_4_S: 443.0 [M + Na]^+^.

###### 4.1.2.4.4 *Tert*‐butyl (isopentyloxy)(6‐(trifluoromethoxy)benzo[*d*]thiazol‐2‐yl)carbamate (47)

Yield 3% (17.0 mg). Pale yellow solid; mp 68 °C. ^1^H NMR (499 MHz, DMSO‐d_6_) *δ* 8.43 (d, *J* = 1.3 Hz, 1H), 8.33 (d, *J* = 8.9 Hz, 1H), 7.65 (dd, *J* = 8.6, 2.1 Hz, 1H), 3.86 (t, *J* = 6.4 Hz, 2H), 1.71 (dt, *J* = 13.4, 6.7 Hz, 1H), 1.46 (s, 9H), 1.44–1.40 (m, 2H), 0.89 (d, *J* = 6.7 Hz, 6H). ESI‐MS (methanol) *m/z* C_18_H_23_F_3_N_2_O_4_S: 443.2 [M + Na]^+^.

###### 4.1.2.4.5 *Tert*‐butyl (benzyloxy)(6‐(trifluoromethoxy)benzo[*d*]thiazol‐2‐yl)carbamate (48)

Yield 40% (300 mg). Pale yellow solid; mp 64 °C. ^1^H NMR (400 MHz, CDCl_3_) *δ* 7.90 (d, *J* = 8.7 Hz, 1H), 7.63 (d, *J* = 1.3 Hz, 1H), 7.53 (dd, *J* = 6.4, 2.9 Hz, 2H), 7.45–7.37 (m, 3H), 7.30 (d, *J* = 7.5 Hz, 1H), 5.23 (s, 2H), 1.55 (s, 9H). ESI‐MS (methanol) *m/z* C_20_H_19_F_3_N_2_O_4_S: 463.0 [M + Na]^+^. Purity HPLC: 99.0%.

###### 4.1.2.4.6 *Tert*‐butyl ((2‐ethylhexyl)oxy)(6‐(trifluoromethoxy)benzo[*d*]thiazol‐2‐yl)carbamate (49)

Yield 22% (142 mg). Pale yellow solid; mp 82 °C. ^1^H NMR (499 MHz, DMSO‐d_6_) *δ* 7.79 (d, *J* = 8.8 Hz, 1H), 7.60–7.51 (m, 2H), 7.41 (dd, *J* = 8.6, 2.9 Hz, 1H), 4.09 (s, 3H), 1.57 (s, 9H), 1.48–1.27 (m, 7H), 1.21 (s, 1H), 0.95 (t, *J* = 7.4 Hz, 3H), 0.89 (t, *J* = 7.0 Hz, 3H). ESI‐MS (methanol) *m/z*: 486.1 [M + Na]^+^. HRMS (ESI‐TOF) *m/z*: C_21_H_29_F_3_N_2_O_4_S experimental 485.1693 [M + Na]^+^, theoretical 485.1692 [M + Na]^+^, Δ = 0.0001. Purity HPLC: 99.2%.

##### 4.1.2.5 General Procedure for the Synthesis of O‐alkyl‐N‐(6‐(trifluoromethoxy)benzo[*d*]thiazol‐2‐yl)hydroxylamine (11–16)

Method A: 2.5 mmol of acetyl chloride was added dropwise at 0 °C to 10 mL of MeOH. Once completed, a solution of 0.5 mmol of **44**, **46**, and **49** in 3 mL of EtOAc was added dropwise to the first solution. The reaction was stirred at room temperature overnight. Then, the solvent was removed under reduced pressure, and the crude was filtered using EtOAc to collect the white solid.

Method B: 4 mL of HCl in 1,4‐dioxane was added to 1 mmol of compound **45**. The mixture was stirred at room temperature overnight. Once completed, the solvent was removed under reduced pressure, and the crude was filtered using EtOAc to collect the white solid of final products **12**.

###### 4.1.2.5.1 O‐Methyl‐N‐(6‐(trifluoromethoxy)benzo[*d*]thiazol‐2‐yl)hydroxylamine Hydrochloride (11)

Method A. Yield 34% (26.0 mg). White solid; mp 135 °C. ^1^H NMR (400 MHz, DMSO‐d_6_) *δ* 7.65 (d, *J* = 1.7 Hz, 1H), 7.16 (dd, *J* = 8.7, 1.6 Hz, 1H), 7.03 (d, *J* = 8.7 Hz, 1H), 3.72 (s, 3H).^13^C NMR (101 MHz, DMSO‐d_6_) *δ* 142.15, 125.20, 124.44, 121.90, 120.21 (CH), 119.35, 116.31 (CH), 111.78 (CH), 62.25 (CH_3_). ESI‐MS (methanol) *m/z*: 264.9 [M + H]^+^. HRMS (ESI‐TOF) *m/z*: C_9_H_7_F_3_N_2_O_2_S experimental 265.0256 [M + H]^+^, theoretical 265.0253 [M + H]^+^, Δ = 0.0003. Purity HPLC: 96.9%.

###### 4.1.2.5.2 O‐Propyl‐N‐(6‐(trifluoromethoxy)benzo[*d*]thiazol‐2‐yl)hydroxylamine Hydrochloride (12)

Method B. Yield 67% (10.0 mg). White solid; mp 157 °C. ^1^H NMR (400 MHz, DMSO‐d_6_) ^1^H NMR (400 MHz, DMSO‐*d*_6_) *δ* 7.91–7.86 (m, 1H), 7.69 (dd, *J* = 11.3 Hz, 2H), 7.17 (s, 1H), 1.65–1.57 (m, 3H), 0.95–0.88 (m, 3H). HRMS (ESI‐TOF) *m/z*: C_11_H_11_F_3_N_2_O_2_S experimental 293.0560 [M + H]^+^, theoretical 293.0566 [M + H]^+^, Δ = 0.0006. Purity HPLC: 93.8%. Only ^1^H NMR was recorded due to the high instability of the compound.

###### 4.1.2.5.3 O‐Pentyl‐N‐(6‐(trifluoromethoxy)benzo[*d*]thiazol‐2‐yl)hydroxylamine Hydrochloride (13)

Method A. Yield 90% (72.0 mg). White solid; mp 139 °C. ^1^H NMR (400 MHz, DMSO‐d_6_) *δ* 7.65 (s, 1H), 7.16 (d, *J* = 8.6 Hz, 1H), 7.04 (d, *J* = 8.6 Hz, 1H), 3.89 (t, *J* = 6.4 Hz, 2H), 1.65–1.52 (m, 2H), 1.36–1.26 (m, 4H), 0.87 (t, *J* = 6.7 Hz, 3H). ^13^C NMR (101 MHz, DMSO‐d_6_) *δ* 158.72, 141.75, 141.22, 125.12, 120.23 (q, *J* = 255.4 Hz), 119.78 (CH), 115.86 (CH), 111.65 (CH), 73.75 (CH_2_), 28.11 (CH_2_), 27.75 (CH_2_), 22.00 (CH_2_), 13.97 (CH_3_). ESI‐MS (methanol) *m/z*: 321.0 [M + H]^+^, 353.3 [M + Na]^+^. HRMS (ESI‐TOF) *m/z*: C_13_H_15_F_3_N_2_O_2_S experimental, 321.0880 [M + H]^+^, theoretical 321.0879 [M + H]^+^, Δ = 0.0001. Purity HPLC: 99.6%.

###### 4.1.2.5.4 O‐Isopentyl‐N‐(6‐(trifluoromethoxy)benzo[*d*]thiazol‐2‐yl)hydroxylamine Hydrochloride (14)

Method A. Yield 60% (14.5 mg). White solid; mp 141 °C. ^1^H NMR (400 MHz, DMSO‐d_6_) ^1^H NMR (400 MHz, DMSO‐*d*_6_) *δ* 8.42 (s, 1H), 8.31 (d, *J* = 8.8 Hz, 1H), 7.64 (d, *J* = 8.4 Hz, 1H), 1.27–1.19 (m, 5H), 0.93–0.71 (m, 6H). ESI‐MS (methanol) *m/z*: 321.2263 [M + H]^+^, 344.0860 [M + Na]^+^. HRMS (ESI‐TOF) *m/z*: C_13_H_15_F_3_N_2_O_2_S experimental, 321.0874 [M + H]^+^, theoretical 321.0879 [M + H]^+^, Δ = 0.0005. Purity HPLC: 96.1%. Only ^1^H NMR was recorded due to the high instability of the compound.

###### 4.1.2.5.5 O‐Benzyl‐N‐(6‐(trifluoromethoxy)benzo[*d*]thiazol‐2‐yl)hydroxylamine Hydrochloride (15)

Method A. Yield 86% (206 mg). White solid; mp 195 °C. ^1^H NMR (400 MHz, DMSO‐d_6_) *δ* 7.64 (s, 1H), 7.43–7.26 (m, 5H), 7.15 (d, *J* = 9.2 Hz, 1H), 7.00 (d, *J* = 8.6 Hz, 1H), 4.95 (s, 2H). ^13^C NMR (101 MHz, DMSO‐d_6_) *δ* 158.48, 141.76, 140.57, 137.88, 128.30 (CH), 128.07 (CH), 127.80 (CH), 124.71, 119.86 (CH), 115.93 (CH), 111.26 (CH), 75.54 (CH_2_). ESI‐MS (methanol) *m*/*z*: 341.0 [M + H]^+^. HRMS (ESI‐TOF) *m/* *z*: C_15_H_11_F_3_N_2_O_2_S experimental 341.0565 [M + H]^+^, theoretical 341.0566 [M + H]^+^, Δ = 0.0001. Purity HPLC: 98.0%.

###### 4.1.2.5.6 O‐(2‐Ethylhexyl)‐N‐(6‐(trifluoromethoxy)benzo[*d*]thiazol‐2‐yl)hydroxylamine Hydrochloride (16)

Method A. Yield 48% (10.0 mg). White solid; mp 154 °C. ^1^H NMR (499 MHz, DMSO‐d_6_) *δ* 12.11 (s, 1H), 7.87 (d, *J* = 8.8 Hz, 1H), 7.53–7.45 (m, 1H), 7.19 (d, *J* = 8.7 Hz, 1H), 1.62–1.30 (m, 6H), 1.03 (t, *J* = 7.4 Hz, 1H), 0.90 (dt, *J* = 34.8, 7.1 Hz, 6H). ^13^C NMR (101 MHz, DMSO‐d_6_) *δ* 170.30 (d, *J* = 11.4 Hz), 161.38, 149.21 (d, *J* = 34.7 Hz), 134.38 (d, *J* = 257.6 Hz), 124.43 (d, *J* = 97.8 Hz, CH), 119.96 (d, *J* = 15.9 Hz), 117.01–114.74 (CH), 112.51 (CH), 76.57 (CH_2_), 38.83–38.18 (CH), 29.96 (d, *J* = 6.8 Hz, CH_2_), 28.60 (d, *J* = 6.8 Hz, CH_2_), 22.62 (CH_2_), 22.55 (CH_2_), 14.12 (d, *J* = 9.4 Hz, CH_3_), 11.15 (d, *J* = 17.5 Hz, CH_3_). ESI‐MS (methanol) *m/z* C_16_H_21_F_3_N_2_O_2_S: 363.13 [M + H]^+^. Purity HPLC:95.4%. NMR and HPLC analyses provided evidence of the high instability of the compounds; mass spectrometry confirmed their successful synthesis.

#### 4.1.3 Synthesis of 2‐(2‐alkylhydrazineyl)‐6‐(trifluoromethoxy)benzo[*d*]thiazole Hydrochloride Derivatives (17–21, 23–25)

##### 4.1.3.1 Synthesis of di‐*tert*‐butyl hydrazine‐1,2‐dicarboxylate (51)

A mixture of 3.63 g (16.6 mmol) of di‐*tert*‐butyl dicarbonate, commercially available (**50**), and 2.00 g (15.0 mmol) of di‐*tert*‐butyl carbazate was allowed to react at room temperature for 20 min. Once completed, the porous solid was crystallized using chloroform – light petroleum 1:4 and filtered, collecting 3.06 g of white solid. Yield 88%; mp 120–122 °C. ^1^H NMR (400 MHz, DMSO‐d_6_) *δ* 8.56 (s, 2H), 1.37 (s, 18H).

##### 4.1.3.2 General Procedure for the Synthesis of di‐*tert*‐butyl 1‐alkylhydrazine‐1,2‐dicarboxylate (52–59)

A mixture of 1.00 g (4.30 mmol) of di‐*tert*‐butyl decarbonate (**51**) and 2.80 g (8.60 mmol) of cesium carbonate in 20 mL of anhydrous DMF was allowed to react at room temperature for 1 h. Then, 4.73 mmol of the desired alkyl bromide was added. The reaction was stirred at room temperature for 12 h. The mixture was diluted with water and extracted with EtOAc (3 × 20 mL). The organic layers were washed with water and brine and dried over anhydrous sodium sulphate. The solvent was concentrated under reduced pressure, and the crude product was purified by flash chromatography.

###### 4.1.3.2.1 Di‐*tert*‐butyl 1‐methylhydrazine‐1,2‐dicarboxylate (52)

Flash chromatography eluent: light petroleum 93% – EtOAc 7%. Yield 50% (666 mg). White solid; mp 53–58 °C. ^1^H NMR (400 MHz, CDCl_3_) *δ* 6.40 (s, 1H), 3.11 (s, 3H), 1.47 (d, *J* = 5.9 Hz, 18H).

###### 4.1.3.2.2 Di‐*tert*‐butyl 1‐propylhydrazine‐1,2‐dicarboxylate (53)

Flash chromatography eluent: light petroleum 93% – EtOAc 7%. Yield 64% (1.67 g). White solid; mp 55 °C. ^1^H NMR (400 MHz, DMSO‐d_6_) *δ* 9.01 (s, 1H), 3.23 (s, 2H), 1.38 (m, 20H), 0.81 (t, *J* = 7.1 Hz, 3H). ES‐MS (methanol) *m/z* C_13_H_26_N_2_O_4_: 297.1 [M + Na]^+^, 313.1 [M + K]^+^.

###### 4.1.3.2.3 Di‐*tert*‐butyl 1‐pentylhydrazine‐1,2‐dicarboxylate (54)

Flash chromatography eluent: light petroleum 93% – EtOAc 7%. Yield 88% (1.37 g). White solid; mp 54–55 °C. ^1^H NMR (400 MHz, DMSO‐d_6_) *δ* 9.01 (s, 1H), 3.31 (s, 2H), 1.38 (m, 20H), 1.26 (dd, *J* = 13.5, 6.9 Hz, 4H), 0.85 (t, *J* = 6.8 Hz, 3H). ES‐MS (methanol) *m/z* C_15_H_30_N_2_O_4_: 325.1 [M + Na]^+^, 341.1 [M + K]^+^.

###### 4.1.3.2.4 Di‐*tert*‐butyl 1‐isopentylhydrazine‐1,2‐dicarboxylate (55)

Flash chromatography eluent: light petroleum 90% – EtOAc 10%. Yield 62% (1.22 g). White solid; mp 70–74 °C. ^1^H NMR (400 MHz, CDCl_3_) *δ* 6.43–5.91 (m, 1H), 3.44 (s, 2H), 1.60–1.35 (m, 21H), 0.90 (d, *J* = 6.6 Hz, 6H).

###### 4.1.3.2.5 Di‐*tert*‐butyl 1‐benzylhydrazine‐1,2‐dicarboxylate (56)

Flash chromatography eluent: light petroleum 90% – EtOAc 1%. Yield 62% (1.30 g). White solid; mp 108 °C. ^1^H NMR (400 MHz, CDCl_3_) *δ* 7.43–7.17 (m, 5H), 6.20 (s, 1H), 4.63 (s, 2H), 1.46 (s, 18H).

###### 4.1.3.2.6 Di‐*tert*‐butyl 1‐(2‐ethylhexyl)hydrazine‐1,2‐dicarboxylate (57)

Flash chromatography eluent: light petroleum 90% – EtOAc 10%. Yield 39% (1.15 g). Transparent liquid. ^1^H NMR (400 MHz, DMSO‐d_6_) *δ* 9.04 (s, 1H), 3.10 (m, 2H), 1.58–1.10 (m, 27H), 0.82 (dt, *J* = 14.4, 6.8 Hz, 6H). ES‐MS (methanol) *m/z* C_18_H_36_N_2_O_4_: 367.2 [M + Na]^+^.

###### 4.1.3.2.7 Di‐*tert*‐butyl 1‐phenethylhydrazine‐1,2‐dicarboxylate (58)

Flash chromatography eluent: light petroleum 93% – EtOAc 7%. Yield 22% (760 mg). ^1^H NMR (400 MHz, DMSO‐d_6_) *δ* 9.16 (s, 1H), 7.32–7.15 (m, 5H), 3.49 (s, 2H), 2.80–2.68 (m, 2H), 1.53–1.13 (m, 18H). ES‐MS (methanol) *m/z* C_18_H_28_N_2_O_4_: 359.1 [M + Na]^+^.

###### 4.1.3.2.8 Di‐*tert*‐butyl 1‐(3‐phenylpropyl)hydrazine‐1,2‐dicarboxylate (59)

Flash chromatography eluent: light petroleum 93% – EtOAc 7%. Yield 31% (590 mg). ^1^H NMR (400 MHz, DMSO‐d_6_) *δ* 9.08 (s, 1H), 7.26 (t, *J* = 7.4 Hz, 2H), 7.17 (dd, *J* = 18.1, 7.1 Hz, 3H), 3.25 (d, *J* = 38.4 Hz, 2H), 2.56 (t, *J* = 7.0 Hz, 2H), 1.71 (d, *J* = 5.7 Hz, 2H), 1.39 (d, *J* = 16.1 Hz, 18H). ES‐MS (methanol) *m/z* C_19_H_30_N_2_O_4_: 373.1 [M + Na]^+^.

##### 4.1.3.3 General Procedure for the Synthesis of 2‐di‐*tert*‐butyl 1‐alkyl‐2‐(6‐(trifluoromethoxy)benzo[*d*]thiazol‐2‐yl)hydrazine‐1,2‐dicarboxylate (60–67)

To a solution of 1.00 equivalent (2.00 mmol) of 2‐chloro‐6‐(trifluoromethoxy)benzo[d]thiazole (**26**) in 5 mL of toluene or acetonitrile, 3.00 equivalents (6.00 mmol) of di‐*tert*‐butyl 1‐alkylhydrazine‐1,2‐dicarboxylate (**52–55** in toluene and **56–59** in acetonitrile), 2.50 equivalents (5.00 mmol) of cesium carbonate, 0.20 equivalents (0.40 mmol) of DPPF and 0.15 equivalents (0.30 mmol) of Pd_2_(dba)_3_ were added to the mixture under argon atmosphere and the reaction was heated overnight at 110 °C in toluene or 60 °C in acetonitrile. Once completed, the catalyst was removed by filtration through a Celite pad. The filtered solution was then washed with brine (2 × 20 mL) and dried over anhydrous sodium sulphate. The solvent was removed under reduced pressure, and the crude was purified with flash chromatography (light petroleum 95% – EtOAc 5%).

###### 4.1.3.3.1 Di‐*tert*‐butyl 1‐methyl‐2‐(6‐(trifluoromethoxy)benzo[*d*]thiazol‐2‐yl)hydrazine‐1,2‐dicarboxylate (60)

Yield 65% (154 mg). White solid; mp 101 °C. 1H NMR (400 MHz, CDCl_3_) *δ* 7.82 (t, *J* = 9.5 Hz, 1H), 7.61 (dd, *J* = 8.7, 1.4 Hz, 1H), 7.29 (dd, *J* = 2.1, 1.2 Hz, 1H), 1.60 (s, 9H), 1.53 (s, 3H), 1.34 (s, 9H). ESI‐MS (methanol) *m/z* C_19_H_24_F_3_N_3_O_5_S: 486.1 [M + Na]^+^.

###### 4.1.3.3.2 Di‐*tert*‐butyl 1‐propyl‐2‐(6‐(trifluoromethoxy)benzo[*d*]thiazol‐2‐yl)hydrazine‐1,2‐dicarboxylate (61)

Yield 19% (110 mg). Pale yellow solid; mp 193 °C. 1H NMR (400 MHz, DMSO‐d_6_) *δ* 8.11 (s, 1H), 7.90–7.83 (m, 1H), 7.40 (d, *J* = 8.8 Hz, 1H), 3.81–3.34 (m, 2H), 1.82–1.33 (m, 20H), 0.90 (q, *J* = 7.5 Hz, 3H). ESI‐MS (methanol) *m/z* C_21_H_28_F_3_N_3_O_5_S: 514.2 [M + Na]^+^, 530.1 [M + K]^+^, 414.0 [(M‐Boc)+Na]+, 392.1 [(M‐Boc)+H]+, 292.0 [(M‐2Boc) + H]^+^.

###### 4.1.3.3.3 Di‐*tert*‐butyl 1‐pentyl‐2‐(6‐(trifluoromethoxy)benzo[*d*]thiazol‐2‐yl)hydrazine‐1,2‐dicarboxylate (62)

Yield 8% (47.0 mg). White solid; mp 239 °C. ^1^H NMR (499 MHz, DMSO‐d_6_) *δ* 8.15–8.10 (m, 1H), 7.86 (dd, *J* = 13.6, 8.8 Hz, 1H), 7.41 (d, *J* = 8.2 Hz, 1H), 3.87–3.67 (m, 2H), 1.42–1.37 (m, 22H), 1.30 (d, *J* = 6.8 Hz, 4H), 0.87 (t, 3H). ^13^C NMR (101 MHz, DMSO‐d_6_) *δ* 165.63 (s), 155.32 (d, *J* = 33.0 Hz), 154.63 (s), 144.96 (s), 134.82 (s), 132.03 (d, *J* = 4.8 Hz), 121.87 (s), 121.48 (s), 109.99 (s), 80.16 (s), 49.20 (s), 28.98 (d, *J* = 2.9 Hz), 28.76–27.70 (m), 27.31 (d, *J* = 5.3 Hz), 22.61–22.11 (m), 14.28 (d, *J* = 1.2 Hz). ESI‐MS (methanol) *m/z*: 520.21 [M + H]^+^, 542.19 [M + Na]^+^, 558.17 [M + K]^+^, 420.16 [(M‐Boc)+H]^+^, 442.14 [(M‐Boc)+Na]^+^, 458.11 [(M‐Boc)+K]^+^, 320.10 [(M‐2Boc)+H]^+^. HRMS (ESI‐TOF) *m/z*: C_23_H_32_F_3_N_3_O_5_S experimental 542.1910 [M + Na]^+^, theoretical 542.1907 [M + Na]^+^, Δ = 0.0003. Purity HPLC: 99.7%.

###### 4.1.3.3.4 Di‐*tert*‐butyl 1‐isopentyl‐2‐(6‐(trifluoromethoxy)benzo[*d*]thiazol‐2‐yl)hydrazine‐1,2‐dicarboxylate (63)

Yield 15% (161 mg). Yellow oil. ^1^H NMR (400 MHz, DMSO‐d_6_) *δ* 8.13 (s, 1H), 7.87 (dd, *J* = 11.5, 8.9 Hz, 1H), 7.44–7.39 (m, 1H), 3.90–3.38 (m, 2H), 1.66 (dt, *J* = 19.9, 6.6 Hz, 1H), 1.54 (d, *J* = 13.4 Hz, 9H), 1.45–1.37 (m, 2H), 1.24 (s, 9H), 0.88 (td, *J* = 6.6, 3.2 Hz, 6H). ^13^C NMR (101 MHz, DMSO‐d_6_) *δ* 171.31, 167.27 (d, *J* = 1.1 Hz), 153.39, 144.70, 136.19, 122.72 (CH), 120.40 (CH), 115.29 (d, *J* = 1.3 Hz, CH), 109.99, 81.04, 46.77 (d, *J* = 1.6 Hz, CH_2_), 36.30 (CH_2_), 28.05 (dd, *J* = 22.7, 13.7 Hz, CH_3_), 25.44 (d, *J* = 16.6 Hz, CH), 23.07– 22.37 (CH_3_). ESI‐MS (methanol) *m/z* C_23_H_32_F_3_N_3_O_5_S: 542.2 [M + Na]^+^, 442.1 [(M‐Boc) + Na]^+^, 558.1 [M + K]^+^.

###### 4.1.3.3.5 Di‐*tert*‐butyl 1‐(2‐ethylhexyl)‐2‐(6‐(trifluoromethoxy)benzo[*d*]thiazol‐2‐yl)hydrazine‐1,2‐dicarboxylate (64)

Yield 3% (25.0 mg). White solid; mp 211 °C. ^1^H NMR (400 MHz, DMSO‐d_6_) *δ* 8.11 (d, *J* = 13.2 Hz, 1H), 7.90–7.81 (m, 1H), 7.41 (d, *J* = 8.9 Hz, 1H), 3.75–3.61 (m, 2H), 3.37 (ddd, *J* = 22.3, 15.2, 7.5 Hz, 2H), 1.60–1.05 (m, 25H), 0.80 (dt, *J* = 16.1, 6.9 Hz, 6H). HRMS (ESI‐TOF, methanol) *m/z* C_26_H_38_F_3_N_3_O_5_S: 562.2519 [M + H]^+^, 584.2341 [M + Na]^+^, 600.2072 [M + K]^+^, 462.1981 [(M‐Boc) + H]^+^.

###### 4.1.3.3.6 Di‐*tert‐*butyl 1‐benzyl‐2‐(6‐(trifluoromethoxy)benzo[*d*]thiazol‐2‐yl)hydrazine‐1,2‐dicarboxylate (65)

Yield 36% (118 mg). Yellow oil. ^1^H NMR (400 MHz, DMSO‐d_6_) *δ* 8.14 (d, *J* = 1.5 Hz, 1H), 7.93 (dd, *J* = 10.2, 8.9 Hz, 1H), 7.49–7.18 (m, 6H), 4.79 (ddd, *J* = 58.7, 42.6, 14.4 Hz, 2H), 1.43–1.15 (m, 18H). Purity HPLC: 99.7%. ESI‐MS (methanol) *m/z* C_25_H_28_F_3_N_3_O_5_S: 562.0 [M + Na]^+^, 462.0 [(M‐Boc)+Na]^+^. HRMS (ESI‐TOF) *m/z*: C_25_H_28_F_3_N_3_O_5_S experimental 540.1775 [M + H]^+^, theoretical 540.1775 [M + H]^+^, Δ = 0.0000.

###### 4.1.3.3.7 Di*‐tert*‐butyl 2‐phenethyl‐1‐(6‐(trifluoromethoxy)benzo[d]thiazol‐2‐yl)hydrazine‐1‐carboxylate (66)

Obtained the mono‐Boc product. Yield 8% (50.0 mg). White solid; mp 215 °C. ESI‐MS (methanol) *m/z* C_25_H_30_F_3_N_3_O_5_S: 542.2 [M + H]^+^, 564.2 [M + Na]^+^, 422.1 [(M‐Boc)+H]^+^, 464.1 [(M‐Boc) + Na]^+^.

###### 4.1.3.3.8 Di‐*tert*‐butyl 1‐(3‐phenylpropyl)‐2‐(6‐(trifluoromethoxy)benzo[d]thiazol‐2‐yl)hydrazine‐1,2‐dicarboxylate (67)

Yi‐eld 6% (31.0 mg). White solid; mp 231 °C. ESI‐MS (methanol) *m/z* C_27_H_32_F_3_N_3_O_5_S: 590.2 [M + Na]^+^, 468.5 [(M‐Boc) + H]^+^, 490.4 [(M‐Boc) + Na]^+^.

##### 4.1.3.4 General Procedure for the Synthesis of 2‐(2‐alkyl hydrazineyl)‐6‐(trifluoromethoxy)benzo[*d*]thiazole Hydrochloride (17–21, 23–25)

Method A: 2.5 mmol of acetyl chloride was added dropwise at 0 °C to 10 mL of MeOH. Once completed, a solution of 0.5 mmol of **60–62, 64** in 3 mL of EtOAc was added dropwise to the first solution. The reaction was stirred at room temperature overnight. Then, the solvent was removed under reduced pressure, and the crude product was filtered using EtOAc to collect the white solid.

Method B: 4 mL of HCl in diethyl ether was added to 1 mmol of compounds **63, 65–67**. The mixture was stirred at room temperature overnight. Once completed, the reaction was filtered using diethyl ether and DCM to collect white solid of final products **20, 23–25**.

###### 4.1.3.4.1 2‐(2‐Methylhydrazineyl)‐6‐(trifluoromethoxy)benzo[*d*]thiazole Hydrochloride (17)

Method A. Yield 36% (14.0 mg). White solid; mp 138 °C. ^1^H NMR (400 MHz, DMSO‐d_6_) *δ* 7.96 (bs, 1H), 7.55 (d, *J* = 8.6 Hz, 1H), 7.33 (dd, *J* = 8.7, 1.7 Hz, 1H), 2.75 (s, 3H). ^13^C NMR (101 MHz, DMSO‐d_6_) *δ* 143.61, 121.89, 120.35 (d, *J* = 3.4 Hz, CH), 119.35 (CH), 115.99 (CH), 112.89, 109.99, 37.13 (CH_3_). ESI‐MS (methanol) *m/z*: 264.0 [M + H]^+^. HRMS (ESI‐TOF) *m/z*: C_9_H_8_F_3_N_3_OS experimental 264.0417 [M + H]^+^, theoretical 264.0413 [M + H]^+^, Δ = 0.0004. Purity HPLC: 96.3%.

###### 4.1.3.4.2 2‐(2‐Propylhydrazineyl)‐6‐(trifluoromethoxy)benzo[*d*]thiazole Hydrochloride (18)

Method A. Yield 5% (42.4 mg). White solid; mp 180 °C. ^1^H NMR (400 MHz, DMSO‐d_6_) *δ* 10.66 (s, 1H), 7.92 (s, 1H), 7.49 (d, *J* = 8.6 Hz, 1H), 7.30 (d, *J* = 7.8 Hz, 1H), 3.79 (s, 2H), 2.91 (d, *J* = 6.0 Hz, 2H), 1.53 (d, *J* = 7.1 Hz, 2H), 0.91 (t, *J* = 7.4 Hz, 3H). ^13^C NMR (101 MHz, DMSO‐d_6_) *δ* 177.11, 160.57, 143.32 (CH), 121.88 (CH), 120.29 (CH), 115.81 (CH_2_), 52.59 (CH_2_), 11.83 (CH_3_). ESI‐MS (methanol) *m/z*: 292.0 [M + H]^+^. HRMS (ESI‐TOF) *m/z*: C_11_H_12_F_3_N_3_OS experimental 292.0726 [M + H]^+^, theoretical 292.0726 [M + H]^+^, Δ = 0.0000. Purity HPLC: 95.4%.

###### 4.1.3.4.3 2‐(2‐Pentylhydrazineyl)‐6‐(trifluoromethoxy)benzo[*d*]thiazole Hydrochloride (19)

Method A. Yield 72% (9.00 mg). Pale yellow solid; mp 174 °C. ^1^H NMR (400 MHz, DMSO‐d_6_) *δ* 7.91 (s, 1H), 7.47 (d, *J* = 8.4 Hz, 1H), 7.29 (d, *J* = 8.5 Hz, 1H), 3.10–3.00 (m, 2H), 2.95–2.79 (m, 2H), 1.31 (ddt, *J* = 10.2, 7.2, 3.7 Hz, 4H), 1.19 (d, *J* = 7.3 Hz, 2H), 0.89–0.84 (m, 3H). ^13^C NMR (101 MHz, DMSO‐d_6_) *δ* 179.80, 176.58, 157.76, 153.08, 120.08 (CH), 119.38 (CH), 115.72 (CH), 109.99, 45.80 (CH_2_), 29.02 (CH_2_), 22.36 (CH_2_), 14.32 (CH_2_), 8.89 (CH_3_). ESI‐MS (methanol) HRMS (ESI‐TOF) *m/z*: C_13_H_16_F_3_N_3_OS experimental 320.1033 [M + H]^+^, theoretical 320.1039 [M + H]^+^, Δ = 0.0006. Purity HPLC: 96.6%.

###### 4.1.3.4.4 2‐(2‐Isopentylhydrazineyl)‐6‐(trifluoromethoxy)benzo[*d*]thiazole Hydrochloride (20)

Method B. Yield 65% (72.0 mg). White solid; mp 166 °C. ^1^H NMR (400 MHz, DMSO‐d_6_) *δ* 7.99–7.94 (m, 1H), 7.54 (d, J = 8.7 Hz, 1H), 7.34 (dd, J = 8.8, 1.7 Hz, 1H), 3.02 (t, J = 7.8 Hz, 2H), 1.67 (dq, J = 13.4, 6.7 Hz, 1H), 1.45 (q, J = 7.4 Hz, 2H), 0.88 (d, J = 6.6 Hz, 6H).^13^C NMR (101 MHz, DMSO‐d_6_) *δ* 143.80, 124.41, 121.86, 120.61 (CH), 119.91, 119.32 (CH), 116.77, 116.23 (CH), 48.72 (CH_2_), 34.94 (CH_2_), 25.74 (CH), 22.81 (CH_3_). ESI‐MS (methanol) *m/z*: 320.0 [M + H]^+^. HRMS (ESI‐TOF) *m/z*: C_13_H_16_F_3_N_3_OS experimental 320.1036 [M + H]^+^, theoretical 320.1039 [M + H]^+^, Δ = 0.0003. Purity HPLC: 98.4%.

###### 4.1.3.4.5 2‐(2‐(2‐Ethylhexyl)hydrazineyl)‐6‐(trifluoromethoxy)benzo[*d*]thiazole Hydrochloride (21)

Method A. Yield 3% (7.00 mg). Yellow solid; mp 216 °C. ^1^H NMR (400 MHz, DMSO‐d_6_) *δ* 7.24 (s, 1H), 7.11 (s, 1H), 6.98 (s, 1H), 4.16 (dd, *J* = 4.7, 3.2 Hz, 2H), 2.70–2.63 (m, 6H), 1.19–1.10 (m, 4H), 0.91–0.76 (m, 6H). ESI‐MS (methanol) *m/z*: 362.1304 [M + H]^+^. HRMS (ESI‐TOF) *m/z*: C_15_H_12_F_3_N_3_OS experimental 362.1509 [M + H]^+^, theoretical 362.1508 [M + H]^+^, Δ = 0.0001. Purity HPLC: 96.6%. The limited amount of material and poor solubility in deuterated solvents hampered NMR characterisation; therefore, reliable ^13^C NMR data could not be obtained. Identity was supported by HRMS and the available ^1^H NMR spectrum.

###### 4.1.3.4.6 2‐(2‐Benzylhydrazineyl)‐6‐(trifluoromethoxy)benzo[*d*]thiazole Hydrochloride (23)

Method B. Yield 67% (42.0 mg). White solid; mp 184 °C. ^1^H NMR (400 MHz, DMSO‐d_6_) *δ* 7.92 (s, 1H), 7.48 (t, J = 7.4 Hz, 1H), 7.43 (d, J = 7.4 Hz, 2H), 7.32 (dt, J = 19.9, 7.2 Hz, 4H), 4.07 (s, 2H). ^13^C NMR (101 MHz, DMSO‐d_6_) *δ* 143.42, 137.11, 129.51, 128.99 (CH), 128.68 (CH), 127.99 (CH), 124.41, 121.87, 120.42, 119.32 (CH), 117.48 (CH), 116.02 (d, *J* = 2.0 Hz, CH), 54.77 (CH_2_). ESI‐MS (methanol) *m/z*: 340.0 [M + H]^+^. HRMS (ESI‐TOF) *m/z*: C_15_H_12_F_3_N_3_OS experimental 340.0727 [M + H]^+^, theoretical 340.0726 [M + H]^+^, Δ =  0.0001. Purity HPLC: 99.5%.

###### 4.1.3.4.7 2‐(2‐Phenethylhydrazineyl)‐6‐(trifluoromethoxy)benzo[*d*]thiazole Hydrochloride (24)

Method B. Yield 98%. Yellow solid; mp 236 °C. ^1^H NMR (400 MHz, DMSO‐d_6_) *δ* 7.90 (s, 1H), 7.83–7.77 (m, 1H), 7.51–7.44 (m, 1H), 7.32–7.14 (m, 6H), 2.97–2.86 (m, 2H), 2.65 (dt, *J* = 16.1, 7.7 Hz, 3H), 1.89–1.72 (m, 2H).^13^C NMR (101 MHz, DMSO‐d_6_) *δ* 129.11, 128.41, 127.54, 126.10, 125.90, 125.74, 119.65, 115.10 Quaternary carbon resonances were not observed, likely due to low signal intensity resulting from the limited amount of material and poor solubility of the HCl salt. ESI‐MS (methanol) *m/z*: 354.0 [M + H]^+^, 376.0 [M + Na]^+^. HRMS (ESI‐TOF) *m/z*: C_16_H_14_F_3_N_3_OS experimental 354.0822 [M + H]^+^, theoretical 354.0822 [M + H]^+^, Δ = 0.0000. Purity HPLC: 98.4%.

###### 4.1.3.4.8 2‐(2‐(3‐Phenylpropyl)hydrazineyl)‐6‐(trifluoromethoxy)benzo[*d*]thiazole Hydrochloride (25)

Method B. Yield 47%. Yellow solid; mp 242 °C. ^1^H NMR (400 MHz, DMSO‐d_6_) *δ* 7.88 (s, 1H), 7.83–7.76 (m, 1H), 7.48–7.42 (m, 1H), 7.31–7.14 (m, 5H), 2.94–2.85 (m, 2H), 2.65 (dt, *J* = 15.2, 6.9 Hz, 2H), 1.85–1.73 (m, 2H).^13^C NMR (101 MHz, DMSO‐d6) *δ* 180.79, 167.05, 160.36, 153.00, 142.14, 138.03, 129.44 (CH), 128.99 (CH), 128.83 (CH), 128.72 (d, 3.0 Hz, 2CH), 128.78–128.72 (m, 2CH), 126.22 (CH), 109.99 (CH), 47.71 (CH_2_), 32.54 (CH_2_), 29.41 (CH_2_). HRMS (ESI‐TOF) *m/z*: C_17_H_16_F_3_N_3_OS experimental 368.1037 [M + H]^+^, theoretical 368.1039 [M + H]^+^, Δ = 0.0002. Purity HPLC: 95.2%.

### 4.2 Stability Tests

Stability tests have been conducted with micrOTOF (Bruker) instrument coupled with HPLC (Agilent Technologies 1260 Infinity II) using column InfinityLab Poroshell 120 EC‐C18 (4.6 × 100 mm, 2.8 μm). Samples have been prepared starting from a stock solution in DMSO at the concentration of 10 mM diluted with CH_3_CH for the injection. Runs have been performed with an elution gradient of 70% CH_3_CN/ 30% H_2_O to 90% CH_3_CN/10% H_2_O returning to the initial condition in 20 min with a flow of 0.2 mL/min. Chromatograms have been elaborated using OtofControl (Bruker software).

### 4.3 Molecular Modelling Studies

#### 4.3.1 Hardware and Software Overview

All molecular modelling operations were conducted using the MOE software suite [44]. For protein‐ligand docking, the PLANTS (Protein Ligand ANT System) algorithm [45] was employed; the resulting poses were evaluated through interaction energy calculations, pharmacophoric filtering, and visual inspection, all performed within the integrated MOE environment.

Molecular Dynamics (MD) systems were built using AmberTools22 [46], MD simulations were run using ACE theMD engine [47], and the collected simulations were analysed with VMD‐1.9.3 [48].

Structure preparation, simulation setup, and trajectory processing were carried out on a Linux workstation equipped with a 32‐core Intel Core i9‐14900K processor (6.0 GHz). MD simulations were executed on a GPU cluster consisting of 30 NVIDIA graphics processing units, spanning from GTX 980 to RTX 4090 models.

#### 4.3.2 Protein Structure Preparation

The three‐dimensional structure of the selected complex for analysis was retrieved from the Protein Data Bank (PDB ID: 5OKT) [39], and subsequently prepared within the MOE 2024.06 suite [44]. Structure preparation was carried out using the dedicated “Structure Preparation” tool available within the MOE environment, which addressed common structural issues such as the addition of missing atoms, capping of N‐ and C‐termini, and the assignment of tautomeric forms and side‐chain conformations for ambiguous residues. Partial charges were assigned according to the AMBER14EHT force field, which was also employed for the energy minimisation of hydrogen atoms introduced during the preparation workflow. Protonation states were determined at pH 7.4 via the Protonate3D tool, which identifies the most probable ionisation state for each residue by evaluating its hydrogen‐bonding contribution to the overall structural stability. In cases where multiple side‐chain conformations were present, the rotamer exhibiting the highest occupancy was selected.

#### 4.3.3 Molecular Docking Simulation

Ligand preparation was realised through a pipeline of tools from the OpenEye suite [49]: three‐dimensional coordinates were generated with Omega, the most probable tautomeric states were predicted with Tautomers, and protonation states were assigned using Fixpka (or MOE Wash tool when unlikely protonation states were generated by Fixpka), partial charges were assigned through Molcharge.

Protein‐ligand docking was carried out using PLANTS (Protein Ligand ANT System) algorithm [45], using the CHEM‐PLP scoring function. A sphere of radius 12 Å centred on the centre of mass of the crystallographic ligand was used for the docking search. 20 poses were generated for each ligand.

#### 4.3.4 Pharmacophoric Filter

A structure‐based pharmacophore model was derived from the crystallographic complex 5OKT using the MOE suite. This resulted in the features describing the aromatic scaffold of the ligand and a bi‐dentate hydrogen bond between the ligand and Leu85. Donor and acceptor features were positioned on the ligand's 2‐aminobenzothiazole NH and N; and acceptor and donor features were positioned on Leu85 carbonyland amino groups. In all cases, the dimension of the feature sphere was 2 Å.

#### 4.3.5 Docking Video Maker

An in‐house tool, MMsDocking Video Maker, was employed to automatically generate video representations of docking results, integrating docking poses, per‐residue hydrophobic IEhyd and electrostatic IEele interaction energy data, and experimental binding data into a unified visualisation to facilitate the comparative analysis of docking outcomes. Individual frames were assembled into video format using MEncoder [50]. Background heatmaps of per‐residue IEhyd and IEele values, computed within MOE using Amber14EHT and MMFF94 partial charges for protein and ligands, respectively, were rendered using GNUPLOT 5.2.8 [51]. Two‐dimensional ligand representations were generated using the RDKit cheminformatics toolkit [52]. Docking pose visualisation within the binding site was performed using UCSF Chimera [53].

#### 4.3.6 System Setup for MD Simulations and Equilibration Protocol

Ligand‐protein complexes for molecular dynamics (MD) simulations were prepared using Visual Molecular Dynamics (VMD) 1.9.3 and AmberTools22 [48, 54]. The protein was parameterized with the ff14 SB force field [55], while ligands were described using the General Amber Force Field (GAFF) [56]; partial charges were assigned using the AM1‐BCC method. Systems were solvated in an orthorhombic box with a 20 Å buffer between the complex and the box boundaries using the TIP3P water model 15. Sodium (Na^+^) and chloride (Cl^‐^) ions were added to neutralise the system and achieve a physiological ionic strength of 0.154 M. A 500‐step energy minimisation was then performed using the conjugate gradient algorithm to remove steric clashes and unfavourable contacts.

A two‐step equilibration process was used before the MD production phase. During the first equilibration, all protein and ligand atoms were restrained with a harmonic positional constraint of 5 kcal mol^−1^ Å^−2^ over a 1 ns simulation performed in the canonical (NVT) ensemble. In the second equilibration step, the same restraint was exclusively applied to ligand and protein backbones during an equal‐length simulation in the isothermal–isobaric ensemble (NPT). Pressure was maintained at 1 atm using a Monte Carlo barostat [57], while temperature was controlled at 310 K throughout all equilibration phases via a Langevin thermostat [58].

The ACEMD 3.7.6 engine [47], which is based on the open‐source Python package OpenMM [59], was used for all MD simulations. An integration time step of 2 fs was adopted, and bonds involving hydrogen atoms were constrained using the M‐SHAKE method [60]. Long‐range electrostatic interactions were computed using the particle‐mesh Ewald approach [61], with a switching distance of 7.5 Å for Lennard‐Jones interactions and a cutoff of 9.0 Å for Lennard‐Jones and real‐space electrostatic interactions.

#### 4.3.7 Supervised Molecular Dynamic Simulations

Supervised Molecular Dynamics (SuMD) is a computational method that allows for the exploration of the ligand–target molecular recognition pathway on a reduced time scale. This process involves a series of unbiased Molecular Dynamics (MD) simulations, followed by an assessment of the simulation's progress using an algorithm like tabu search. In each of these MD simulations, referred to as “SuMD‐steps,” the system is simulated in the canonical ensemble at a constant temperature of 310 K. A 300‐picosecond (ps) SuMD‐step was employed in this work.

At the end of each SuMD‐step, the distance between the ligand's centre of mass and the binding site is computed along the trajectory, and the distance data are then fitted using a linear function. If the slope of the resulting linear fit is negative, suggesting that the ligand is approaching the binding site, the “SuMD‐step” is considered productive and used to generate the final trajectory. The simulation's final state is used as the starting point for the next stage. Conversely, if the slope is positive, indicating that the ligand is not getting closer to the binding site, the “SuMD‐step” is considered unproductive and thus discarded. In this example, the step is repeated by randomly altering particle velocities with the Langevin thermostat, while the final coordinates from the previous “SuMD‐step” are retained. The supervision algorithm was deactivated when the distance between the two centres of mass falls below a threshold value (in this instance, 9 Å).

#### 4.3.8 SuMD and Classical MD Trajectory Analysis

Ligand–receptor interaction energies were computed throughout each MD trajectory using the NAMD Energy plugin [62], based on the NAMD engine and the AMBER14 force field [54], as the sum of van der Waals and electrostatic contributions. Trajectory processing and data aggregation were performed using an in‐house Python 3 tool built on the MDAnalysis [63], NumPy, and Matplotlib libraries [64]. Ligand approach during the SuMD simulations was monitored by calculating the RMSD of the ligand and binding site centroid distance with respect to the starting docking pose. In parallel, ligand–receptor interactions (as sum of electrostatics and van der Waals) were analysed by decomposing the interaction energies according to the individual atom contributions of the ligand, in order to monitor the evolution of the contact profile throughout the binding process. The resulting ligand per–atom interaction energies were plotted along time over the trajectory. For each compound, protein‐ligand hydrogen‐bond frequency (percentage occurrence, from 0% to 100%) was computed from the final portions of the three independent SuMD replicas using the ProLIF library [65]. ProLIF defines donor and acceptor roles based on the ligand molecular graph through RDKit [52], enabling force‐field‐independent hydrogen‐bond detection. For each residue‐ligand pair, hydrogen‐bond frequency was calculated as the fraction of simulation frames in which at least one hydrogen–bond interaction was detected, irrespective of donor or acceptor role. The resulting frequency values were subsequently averaged across the three independent replicas to obtain a mean per‐residue frequency. Residue numbering was consistently assigned according to the canonical UniProt FASTA sequence of the corresponding receptor (P48730).

### 4.4 Biology

#### 4.4.1 CK1δ Activity Assays

Compounds were tested on commercially available CK1δ (Merck Millipore, recombinant human truncated form, amino acids 1‐294, GST‐tagged N‐terminal) with the KinaseGlo luminescence kit (Promega), according to a previously reported procedure, with minor modifications, and are described here for completeness [22]. Experiments were performed in a 96‐well plate (white, flat bottom) using buffer prepared as follows: 50 mM HEPES (pH 7.5), 1 mM EDTA, 1 mM EGTA, and 15 mM magnesium acetate. Compound PF‐670462 (IC_50_ CK1δ 0.014 μM) was used as a positive control at concentration of 40 μM, while a solution of DMSO/buffer as negative control [18]. First, 10 μL of inhibitor solution was added in the well (10 mM stock solution in DMSO was diluted with buffer), followed by 10 μL of enzyme (16 ng of CK1δ) and 20 μL of casein substrate 0.1% and 2 μM ATP (final concentration) (to perform ATP competition, increasing concentrations were chosen: 1, 2, 10, 50 μM). The final DMSO concentration in the mixture did not exceed 1%–2%. After 60 min of incubation at 30 °C, the enzymatic reaction was stopped with 40 μL of the KinaseGlo reagent (Promega). The Luminescence signal (RLU) was recorded after 10 min at 25 °C using Tecan Infinite M100. The inhibitory activities were calculated in relation to the maximal activity (absence of inhibitor). As preliminary screening, each compound was assayed at concentration of 40 μM, consequently, for derivative that reported an activity percentage less than 50% at concentration, IC_50_s were determined in triplicate and reported as mean ± standard error. Results were elaborated with Excel and GraphPad Prism 8.0.

ATP‐competition assays were performed following the just described procedure by first performing the ATP calibration line to obtain the slope. The two chosen inhibitor's concentrations agree with the IC_50_ of compound (the concentration at its IC_50_ and the double), and the experiment was conducted in the presence of increasing ATP concentrations: 1, 2, 10, 50 μM. The mean RLU value was divided by the slope of the calibration line, and the mean of negative controls was subtracted from the ATP consumed; the reciprocal characterises the *x‐*axis of the Lineweaver–Burk plot. The ATP consumed divided by the volume of reaction (40 μL) provides the *y*‐value. Results were elaborated using Excel and GraphPad Prism 8.0.

#### 4.4.2 GSK3β Activity Assays

The GSK3β (Thermo‐Scientific) activity assay was carried out using a KinaseGlo luminescence (Promega) protocol, adapted from previously reported procedures, and is reported here for completeness [66]. Experiments were performed in a 96‐well plate (white, flat bottom) using buffer prepared as follows: 40 mM Tris pH 7.5, 20 mM magnesium chloride, 0.1% mg/mL BSA, and 50 μM DTT. As a positive control, the reaction mixture without enzyme was used, while the solution with enzyme, ATP, and substrate was used as a negative control. First, 10 μL of inhibitor solution was added in the well (10 mM stock solution in DMSO was diluted with buffer), followed by 10 μL of GS‐2 as substrate (0.2 μg/μL), 10 μL of ATP (starting from a stock solution at 8 mM, it was diluted with buffer to achieve an initial concentration of 4 μM), and 10 μL of enzyme (1 ng/μL). The final DMSO concentration in the mixture did not exceed 1%–2%. After 60 min of incubation at 25 °C, the enzymatic reaction was stopped with 40 μL of the KinaseGlo reagent (Promega). The Luminescence signal (RLU) was recorded after 10 min at 25 °C using Tecan Promega. The inhibitory activities were calculated in relation to the maximal activity (absence of the inhibitor). As preliminary screening, each compound was assayed at the concentration of 10 μM, consequently, for derivative that reported an activity percentage less than 50% at concentration, IC_50_s were performed in triplicate and reported as mean ± standard error. Results were elaborated with Excel and GraphPad Prism 8.0.

#### 4.4.3 CK1ε Activity Assays

Compounds were tested on commercially available CK1ε (full‐length human protein, N‐terminal GST‐tagged, expressed in *Sf9* insect cells via baculovirus, Promega) using the KinaseGlo luminescence kit (Promega). Experiments were performed in a 96‐well plate (white, flat bottom) using buffer prepared as follows: 50 mM HEPES (pH 7.5), 1 mM EDTA, 1 mM EGTA, and 15 mM magnesium chloride. Compound PF‐670 462 (IC_50_ CK1ε: 0.0077 μM) was used as a positive control at the concentration of 100 μM, while a solution of DMSO/buffer with the same final DMSO concentration as that of the positive control (1%) served as a negative control.

More specifically, 10 μL of inhibitor solution (prepared by serial dilutions starting from a 10 mM stock solution in DMSO to evaluate 10 concentrations starting from 100 μM) was added to each well, followed by 10 μL of enzyme (18.7 ng of CK1ε), 20 μL of casein substrate (0.1% final concentration, diluted from a 5% commercial stock), and ATP (2 μM final concentration, diluted from an 8 mM stock solution with buffer).

After 60 min of incubation at 30 °C, the enzymatic reaction was stopped by adding 40 μL of the KinaseGlo reagent. The luminescence signal (RLU) was recorded after an 8 min incubation at 25 °C. Luminescence was measured using a Tecan Infinite M100.

IC_50_ values were calculated in triplicate and determined relative to maximal enzyme activity and expressed as mean ± standard error. Data analysis was performed using Excel.

#### 4.4.4 p38α Activity Assays

Compounds were also tested on commercially available p38α kinase (human recombinant protein, N‐terminal GST‐tagged, expressed in *Sf9* insect cells via baculovirus, Promega) using the ADP‐Glo Kinase Assay (Promega). The kinase assay was performed using the buffer provided with the kit, consisting of 40 mM Tris pH 7.5, 20 mM MgCl_2_, 0.1 mg/mL BSA, and 50 μM DTT. Each compound was tested at a single concentration of 50 μM, prepared by dilution from a 10 mM stock solution in DMSO, resulting in a final DMSO concentration of 1%. Enzyme activity was evaluated by measuring the percentage of residual kinase activity in the presence of each tested compound relative to the positive control, represented by the absence of the inhibitor. The negative control consisted of the reaction mixture without the enzyme, containing the same final concentration of DMSO (1%) used in the inhibitor‐treated samples.

For each compound, 6.25 μL of inhibitor solution was added to the wells, followed by 6.25 μL of p38α enzyme (4ng) and 12.5 μL of a reaction mixture containing ATP Ultra (final concentration 150 microM, diluted from a 10 mM stock) and p38α substrate (final concentration 0.2 μg/μL, diluted from a stock of 1 mg/mL stock). The reaction mixture was incubated for 60 min at room temperature.

The enzymatic reaction was subsequently stopped by adding 25 μL of ADP‐Glo Reagent, followed by incubation for 40 min at room temperature. Then, 50 μL of Kinase Detection Reagent was added to each well, and the plate was incubated for an additional 30 min at room temperature. Luminescence was measured using Tecan Infinite M100.

IC_50_ values were calculated in triplicate and determined relative to maximal enzyme activity and expressed as mean ± standard error. Data analysis was performed using GraphPad Prism 8.0.

#### 4.4.5 CNS Permeation Prediction: BBB‐PAMPA

The ability of the selected compounds to cross the blood‐brain barrier by passive permeation was evaluated using the PAMPA‐BBB assay, following a previously reported procedure with minor modifications [22]. Commercial drugs with known CNS permeability were used as internal standards to define the permeability cut‐off values. Stock solutions of test compounds and reference drugs were prepared in ethanol and diluted with PBS/ethanol 70:30, pH 7.4. Solutions were filtered through PVDF membrane filters before use.

The acceptor plate was filled with 180 μL of PBS/ethanol 70:30, while the donor PVDF membrane plate was coated with 4 μL/well of porcine brain lipid in dodecane. After 5 min, 180 μL of compound solution was added to the donor wells. The donor plate was then placed onto the acceptor plate to form a sandwich system and incubated for 2.5 h at 25 °C. After incubation, the donor plate was removed, and absorbance values were measured at the appropriate wavelengths using a Tecan Infinite M1000 plate reader. The experiments were performed in duplicate.

The permeability coefficient Pe was calculated as previously described. Experimental permeability values obtained for the reference drugs were correlated with literature data and used to classify compounds as CNS+, CNS−, or CNS+/−.

#### 4.4.6 Recombinant CK1δ 1‐294 Production and Thermal Shift Assay

Recombinant CK1d 1‐294, representing the catalytic core domain of the protein, was produced at the Structural Biology Laboratory at the Elettra Synchrotron. The CK1δ 1‐294 gene was cloned into the expression vector *pNIC28‐Bsa4* with an N‐terminal His6‐tag and TEV cleavage site and was kindly gifted by Prof. Stefan Knapp (Goethe‐University Frankfurt). Protein was expressed at 18 °C for 16–18 h in Rosetta2(DE3) strain in TB medium with antibiotics, induced at OD600 ~2.5 with 0.15 mM 1‐thio‐β‐D‐galactopyranoside (IPTG). Cell culture was harvested at 3500 xg at 4 °C; the pellet was resuspended in Lysis buffer (50 mM Tris; 500 mM NaCl; 10 mM Imidazole; 5% Glycerol; 1 mM TCEP; protease inhibitors; pH 7.5), and cells were disrupted by homogenisation (PandaPlus2000, GEA). The clarified supernatant was incubated with Sepharose NiNTA resin (Qiagen) for 1 h at 4 °C, and protein fractions were eluted by gravity with the same buffer at 300 mM Imidazole. Eluted proteins were treated o/n with TEV protease and lambda‐phosphatase (Sigma) in dialysis (20 mM Tris; 10% Glycerol; 5 mM Imidazole; 300 mM NaCl; 2 mM MnCl_2_; 0.5 mM EDTA; 0.5 mM TCEP; pH 7.5); treated sample then underwent a second passage over Ni‐NTA resin for protease and His_6_‐tag removal. CK1δ 1‐294 was further purified in a Superdex 75 Hiload 16/600 (GE) in storage buffer (20 mM Tris; 180 mM NaCl; 0.5 mM TCEP; pH 7.5). The eluted protein was finally concentrated and flash‐frozen in lN_2_ for long‐term storage at −80 °C.

TSA experiments were conducted in technical triplicates. Samples were prepared in a white 96‐multiwell plate (Biorad) with a final volume of 20 μL into each well. Recombinant CK1d 1‐294 was diluted to a final concentration of 3 mM in TSA buffer (25 mM Tris, 150 mM NaCl, 0.5 mM TCEP, pH 7.5). Compound **20** was tested at increasing final concentrations of 1.5, 3, 9, 15, 30, and 45 μM (0.5×, 1×, 3×, 5×, 10×, and 15× molar excess), keeping a final concentration of 2.5% DMSO. After 30 min of incubation at RT, SYPRO Orange dye (Thermo Fisher Scientific) was added at a final concentration of 5× from 5000× stock, and the emission of the dye was detected at 560–580 nm using a real‐time PCR (CFX96, Bio‐Rad) every 30 s, with a temperature gradient of 2 °C/min. Each analysis was executed in comparison to a negative control (buffer, buffer, and compound) and a positive control (protein and protein with 2.5 % DMSO). Output data were analysed with CFX software.

#### 4.4.7 In Vitro Experiments on Neuroblastoma Cell Lines (SH‐SY5Y)

##### 4.4.7.1 MTT Assays

The human neuroblastoma SH‐SY5Y cell line (RRID: CVCL_0019; purchased from the European Collection of Cell Cultures, Health Protection Agency, Salisbury, UK) was propagated in Dulbecco's Modified Eagle Medium (DMEM) containing L‐glutamine (2 mM), 1% non‐essential amino acids, 1% penicillin/streptomycin, and 10% fetal bovine serum (FBS) under humidified 5% CO_2_. Once semiconfluence was achieved, cells were counted using TC10 Automated Cell Counter Bio‐Rad Laboratories (CSIC‐Madrid), and 80,000 cells/well were plated in a 96‐well plate. After 24 h of incubation, compounds were added at concentrations of 5 and 10 μM (the stock solution in DMSO was diluted with DMEM to obtain the right final concentration), and after another 24 h of incubation, DMEM was removed, and MTT reactive (3‐[4,5‐dimethylthiazol‐2‐yl]‐2,5‐diphenyltetrazolium bromide) was added at the concentration of 20 mg/mL. After 2 h, absorbance was recorded, and cell survival was estimated as a percentage of value of the untreated control (mean of six wells). Three independent experiments were carried out.

##### 4.4.7.2 Neuroprotection Assays

The human neuroblastoma SH‐SY5Y cell line was propagated in Dulbecco's Modified Eagle Medium (DMEM) containing L‐glutamine (2 mM), 1% non‐essential amino acids, 1% penicillin/streptomycin, and 10% fetal bovine serum (FBS) under humidified 5% CO_2_. Once semiconfluence was achieved, cells were counted using TC10 Automated Cell Counter Bio‐Rad Laboratories (CSIC‐Madrid), and 60,000 cells/well were plated in 96‐well plate. After 24 h of incubation, compounds were added at concentrations of 5 and 10 μM (the stock solution in DMSO was diluted with DMEM to obtain the right final concentration), and after 1 h, ethacrynic acid (45 μM, starting from stock solution of 100 mM diluted in DMEM) was added. As negative control, a solution of DMSO 1% in DMEM was used, while as positive control, ethacrynic acid without compound. After 24 h of incubation, DMEM was removed, and MTT reactive (3‐[4,5‐dimethylthiazol‐2‐yl]‐2,5‐diphenyltetrazolium bromide) was added at the concentration of 20 mg/mL. After 2 h, absorbance was recorded, and cell survival was estimated as the mean of relative absorbance of six wells/compound. Three independent experiments were conducted, and statistical analysis of the data was conducted using GraphPad Prism 8.0 one‐way ANOVA for each plate and T‐TEST to compare the three experiments.

## Funding

This study was supported by Ministero dell’Istruzione, dell’Università e della Ricerca (PRIN 20227K7YJS).

## Conflicts of Interest

The authors declare no conflicts of interest.

## Supporting information

Supplementary Material

Supplementary Material

Supplementary Material

## Data Availability

The data that supports the findings of this study are available in the Supporting Information of this article.
