# Supplementary material for: Riluzole‐Derived Benzo[d]thiazoles as CK1δ Inhibitors for Amyotrophic Lateral Sclerosis‐Related Neurodegeneration
Source: ChemMedChem. 2026 Oct 22;21(20):e70539. doi: 10.1002/cmdc.70539 (PMC13691385; doi:10.1002/cmdc.70539)
Supplement: Supplementary file 1 [file CMDC-21-e70539-s001.pdf]

Riluzole-Derived Benzo[d]thiazoles as CK1δ Inhibitors for ALS-Related Neurodegeneration

Simone Adorinni,†[a] Ilenia Grieco,†[a] Stefano Morasso,[b] Michelle Fornasier,[a, b] Gianluca Novello,[c] Veronica Salmaso,[c] Loreto Martínez-González,[d] Eleonora Cescon,[a] Davide Bassani,[c] Giovanni Adriano Bassan,[a] Teresa Gianferrara,[a] Filippo Prencipe,[a] Veronika Kost,[a] Tatiana Da Ros,[a] Paola Storici,[b] Ana Martinez,[d] Giampiero Spalluto[a], Stefano Moro,*[c] and Stephanie Federico*[a]

[a] Department of Chemical and Pharmaceutical Sciences
University of Trieste
Via Licio Giorgieri, 1, 34127, Trieste, Italy
E-mail: sfederico@units.it

[b] Protein Targets for Drug Discovery Lab, Structural Biology
Elettra Sincrotrone Trieste S.C.p.A
SS14 – km 163,5 in AREA Science Park, Basovizza, 34149 Trieste, Italy

[c] Molecular Modeling Section (MMS), Department of Pharmaceutical and Pharmacological Sciences
University of Padua
Via Marzolo 5, 35131 Padova, Italy

[d] Centro de Investigaciones Biológicas, CSIC
Avenida Ramiro de Maeztu 9, 28040, Madrid, Spain

Index

1. Supplementary characterization data 2
2. Concentration-inhibition curves for CK1δ 91
3. Stability studies 92
4. LipE profile 94
5. *In vitro* evaluation of BBB-permeability 95
6. Thermal Shift Assay (TSA) 97
7. Kinase selectivity studies 98
8. MTT assay 100

1. Supplementary characterization data

N-(6-(trifluoromethoxy)benzo[d]thiazol-2-yl)hydroxylamine hydrochloride (**2**)

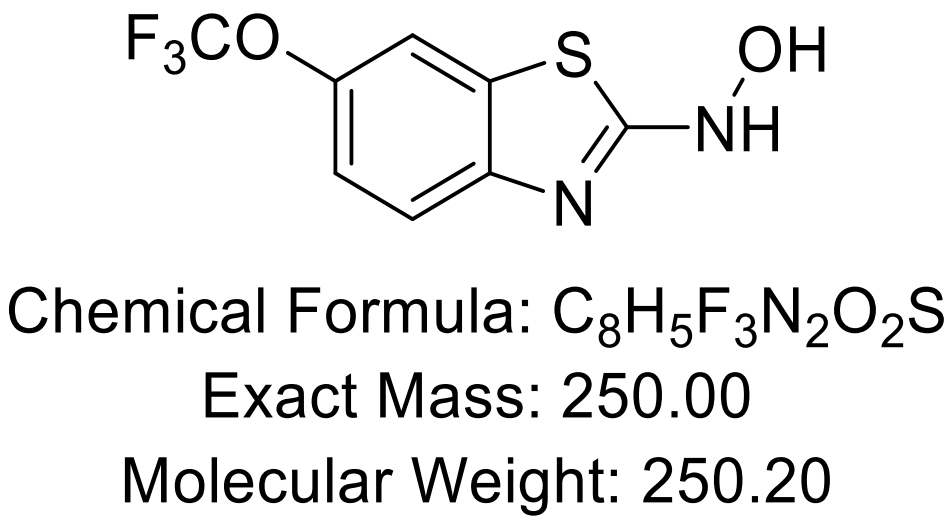

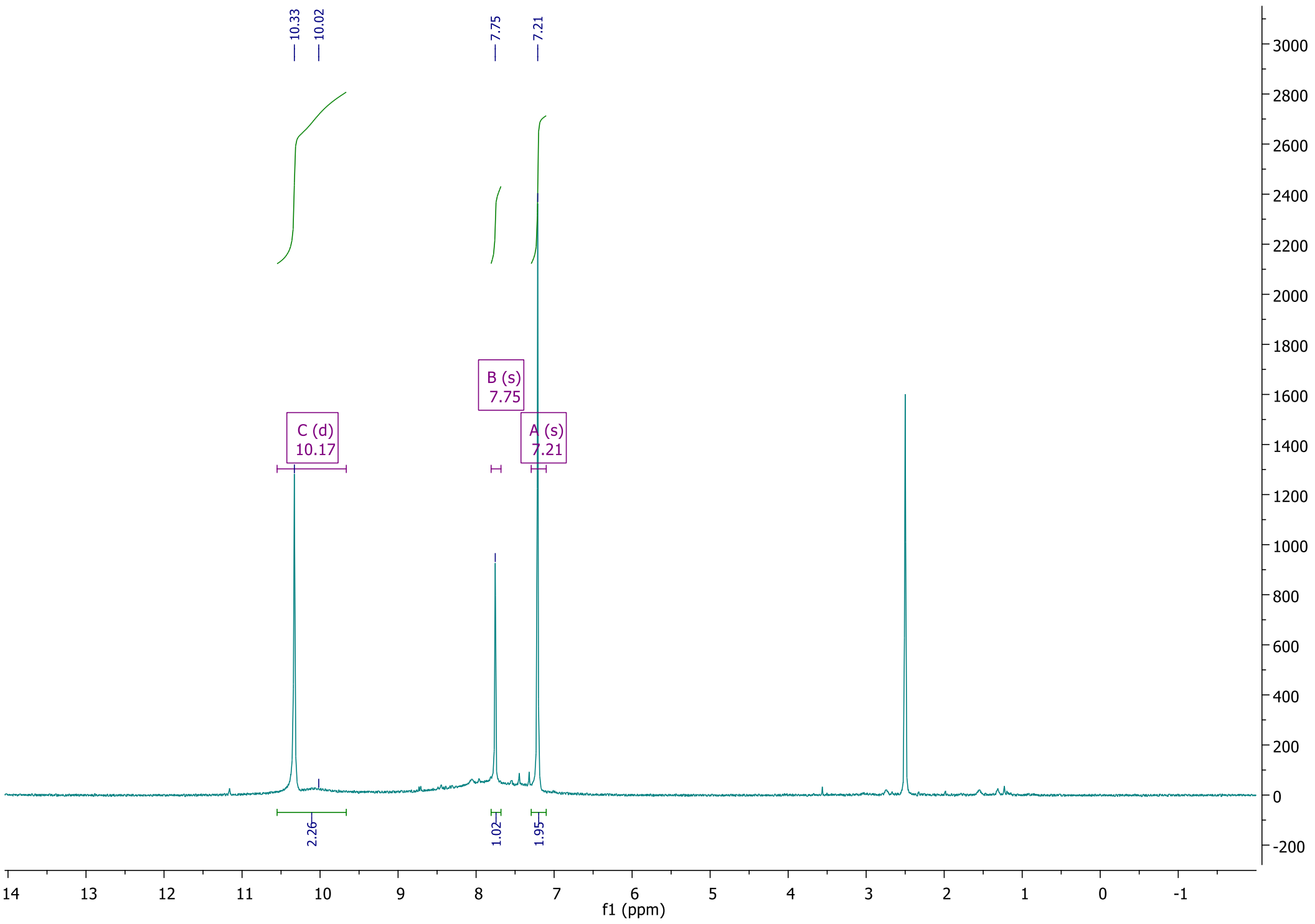

^{1}H-NMR of final compound **2** (DMSO-d_6).

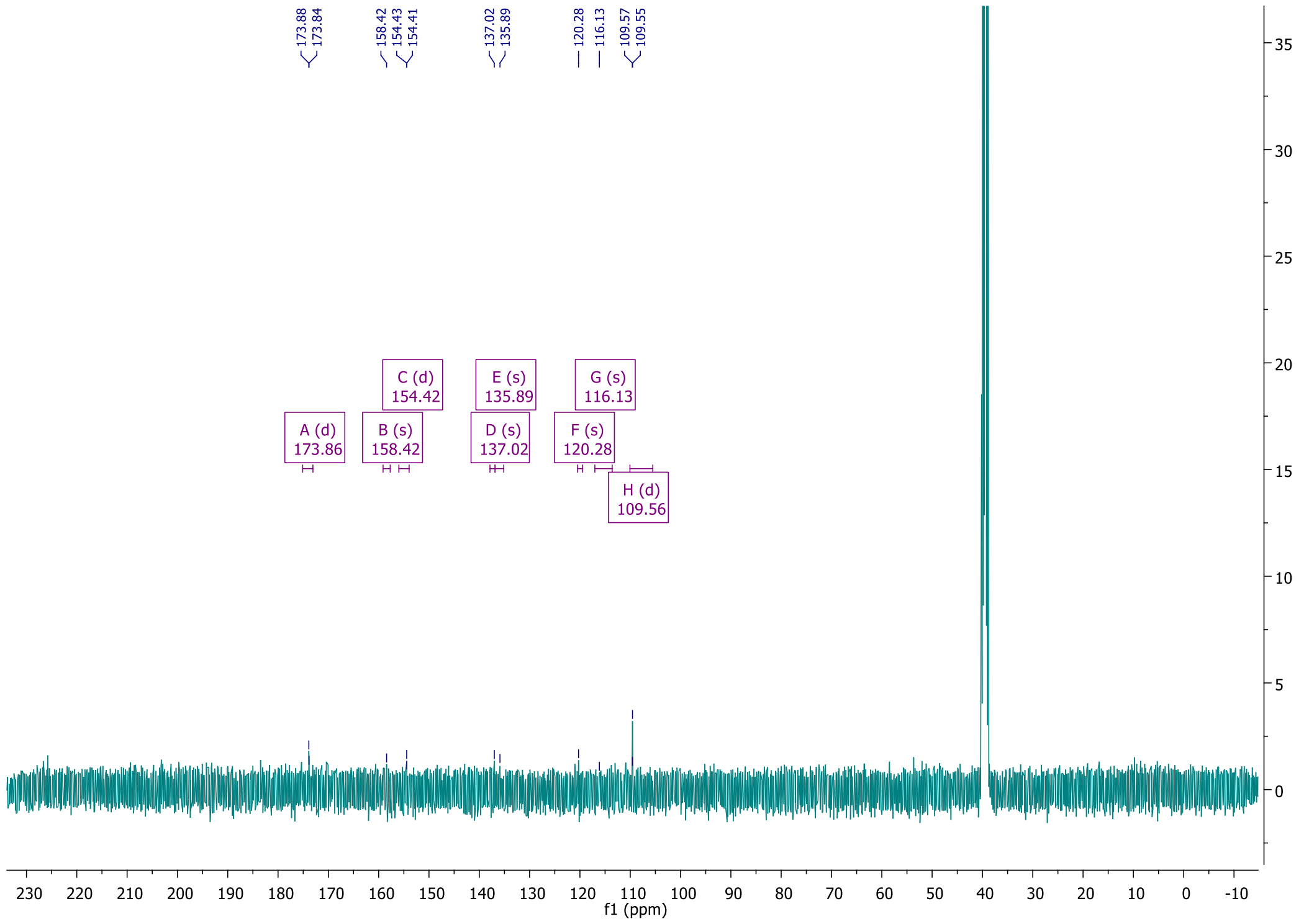

^{13}C-NMR of final compound **2** (DMSO-d_6).

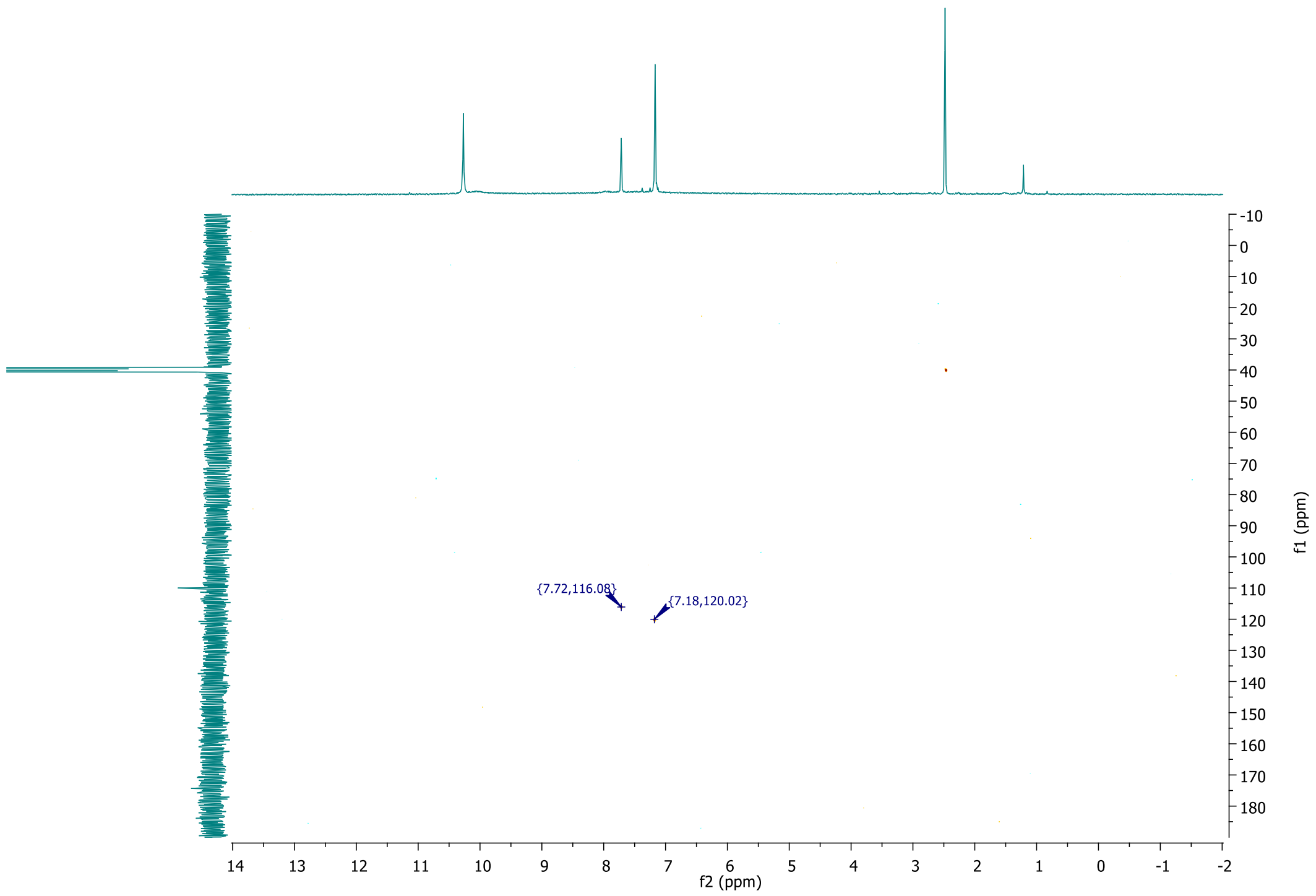

gHSQCAD of final compound **2** (DMSO-d_6).

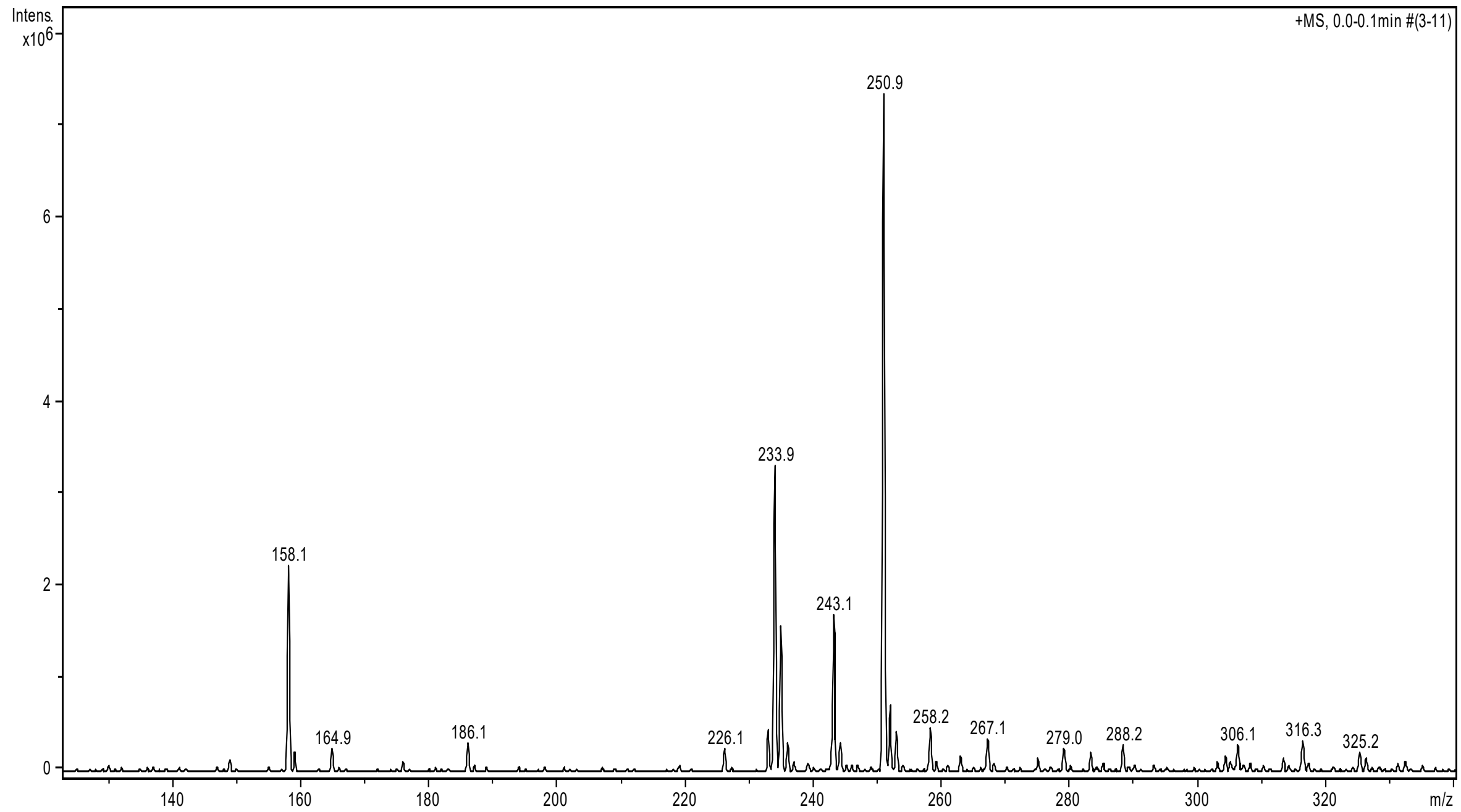

ES-MS of final compound **2** (MeOH).

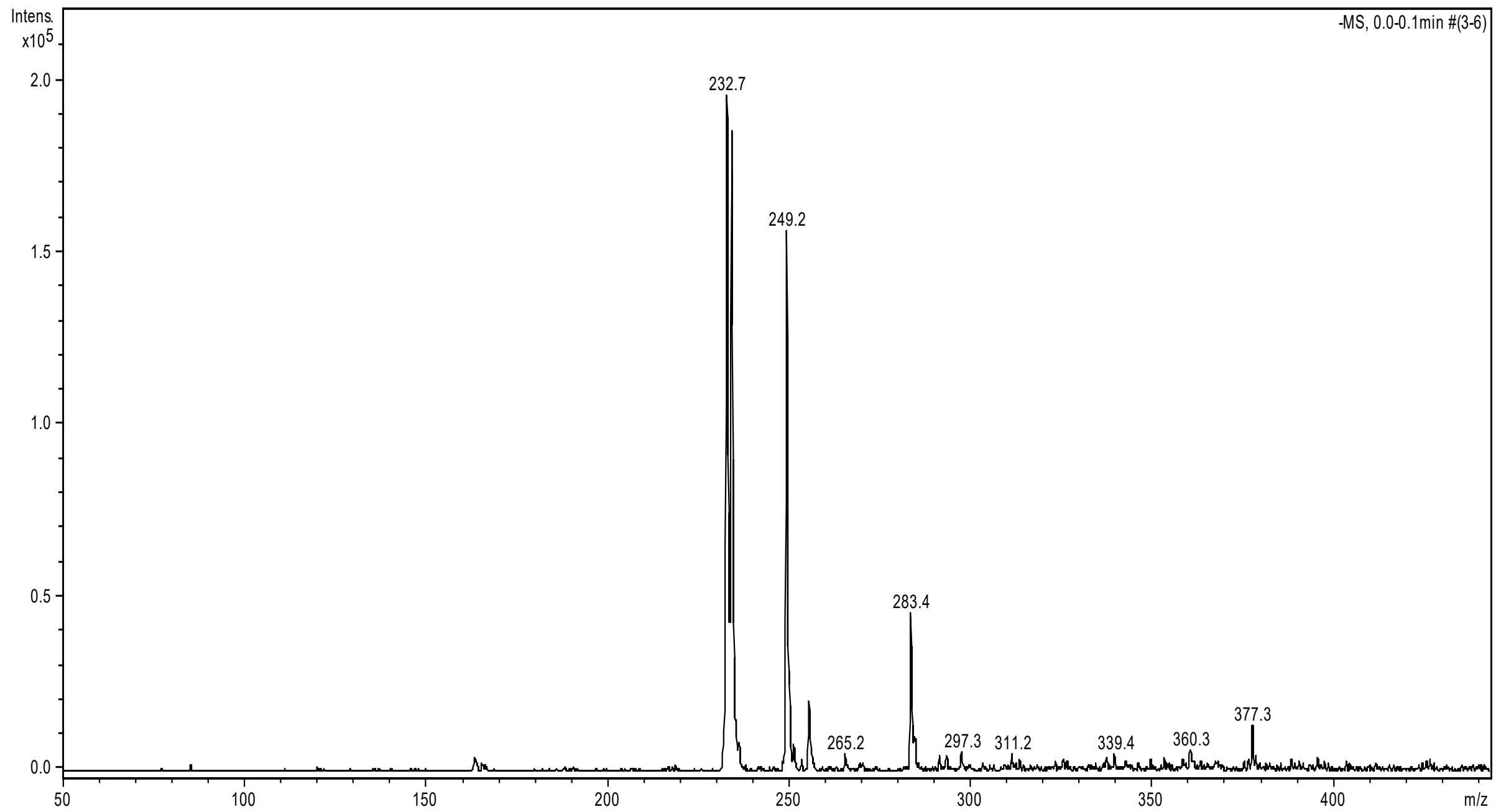

ES-MS negative mode of final compound **2** (MeOH).

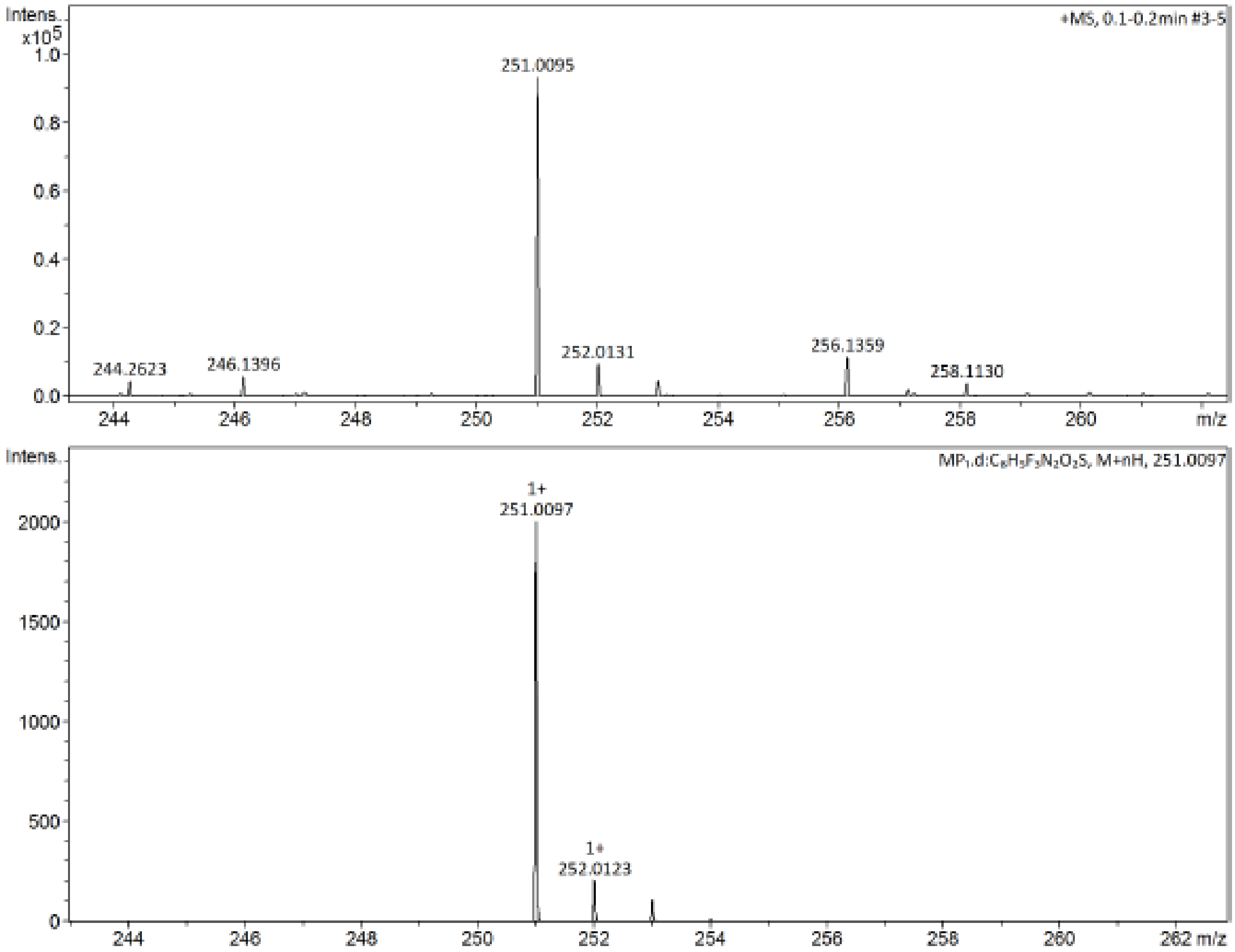

HRMS of final compound **2** (MeOH).

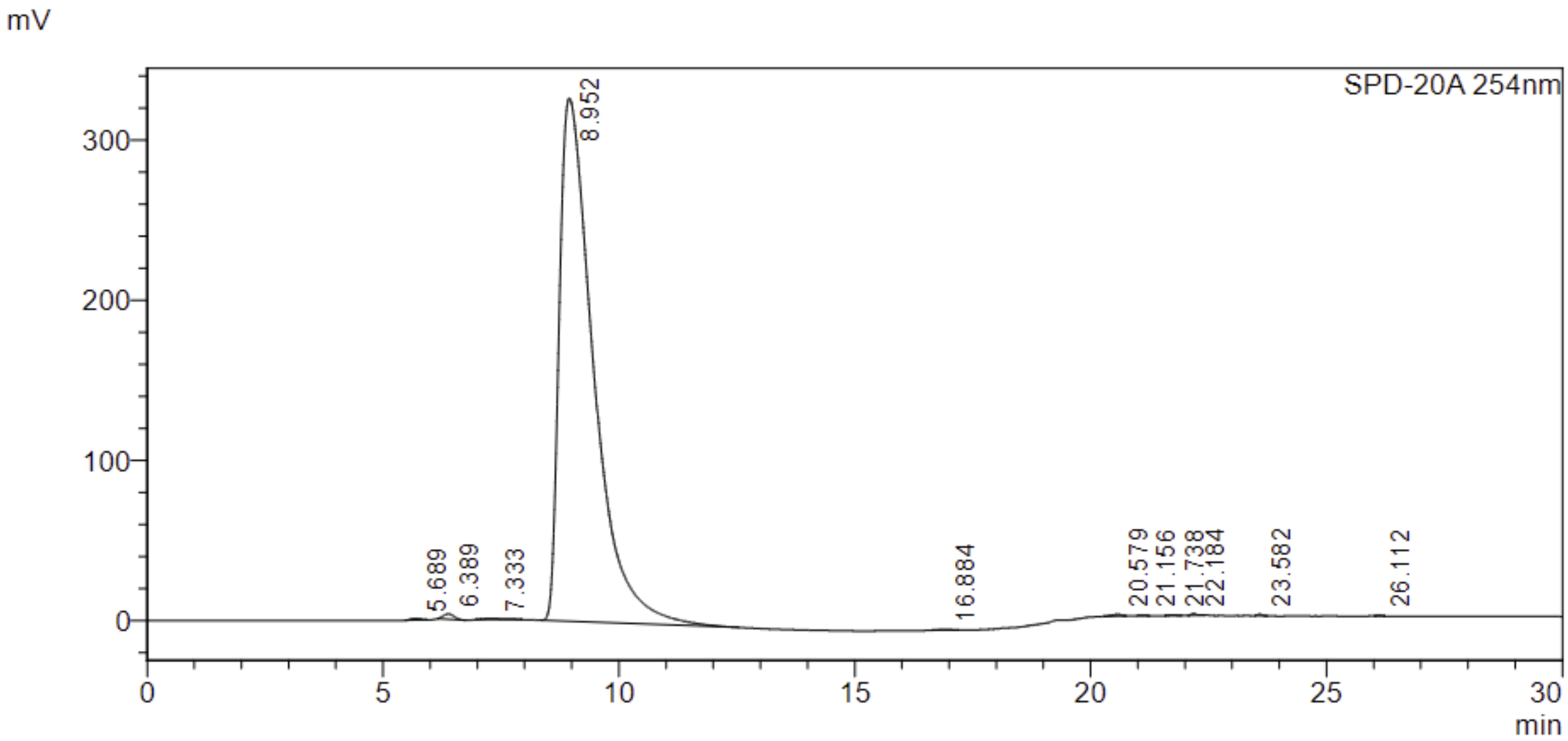

<Peak Table>

SPD-20A 254nm

Peak#	Ret. Time	Area	Height	Conc.	Unit	Mark	Name
1	5.689	12839	939	0.077		M	
2	6.389	52590	3346	0.317		M	
3	7.333	19233	585	0.116		M	
4	8.952	16483854	326784	99.231		M	
5	16.884	4446	210	0.027		M	
6	20.579	13921	1034	0.084		M	
7	21.156	1840	241	0.011		M	
8	21.738	4189	427	0.025		M	
9	22.184	9739	1210	0.059		M	
10	23.582	6021	1000	0.036		M	
11	26.112	2876	442	0.017		M	
Total		16611548	336220				

HPLC of final compound **2** (flow: 1 ml/min, eleunt gradient: from water-methanol 55:45 to 15:85 in 30 min; detector: 254 nm).

2-hydrazineyl-6-(trifluoromethoxy)benzo[d]thiazole (**8**)

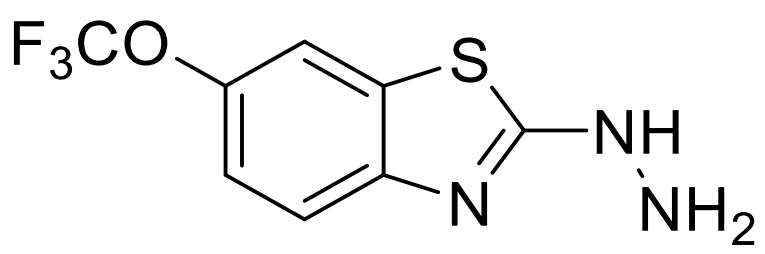

Chemical Formula: $C_8H_6F_3N_3OS$
Exact Mass: 249.02
Molecular Weight: 249.21

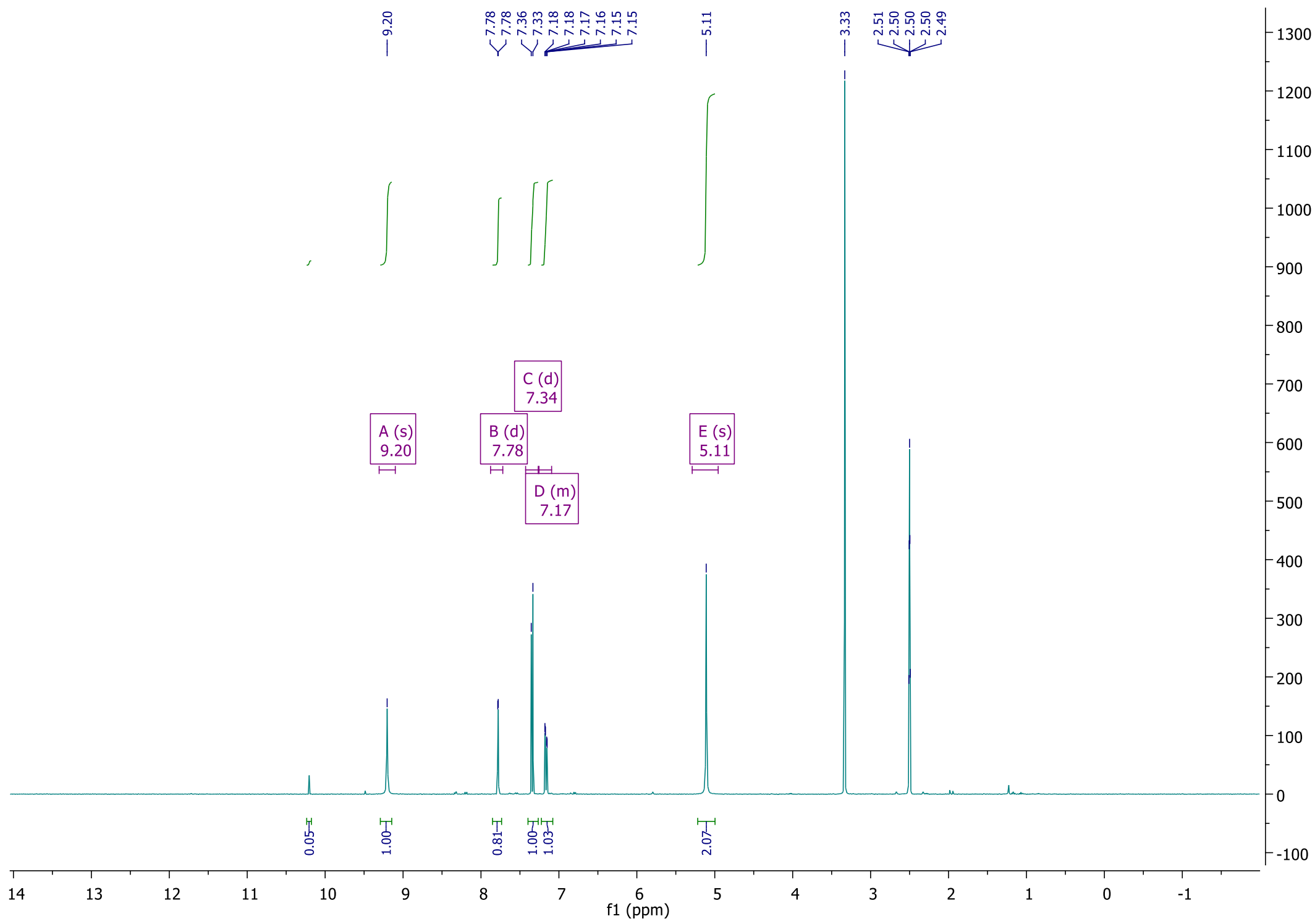

^{1}H-NMR of final compound **8** (DMSO-d_6).

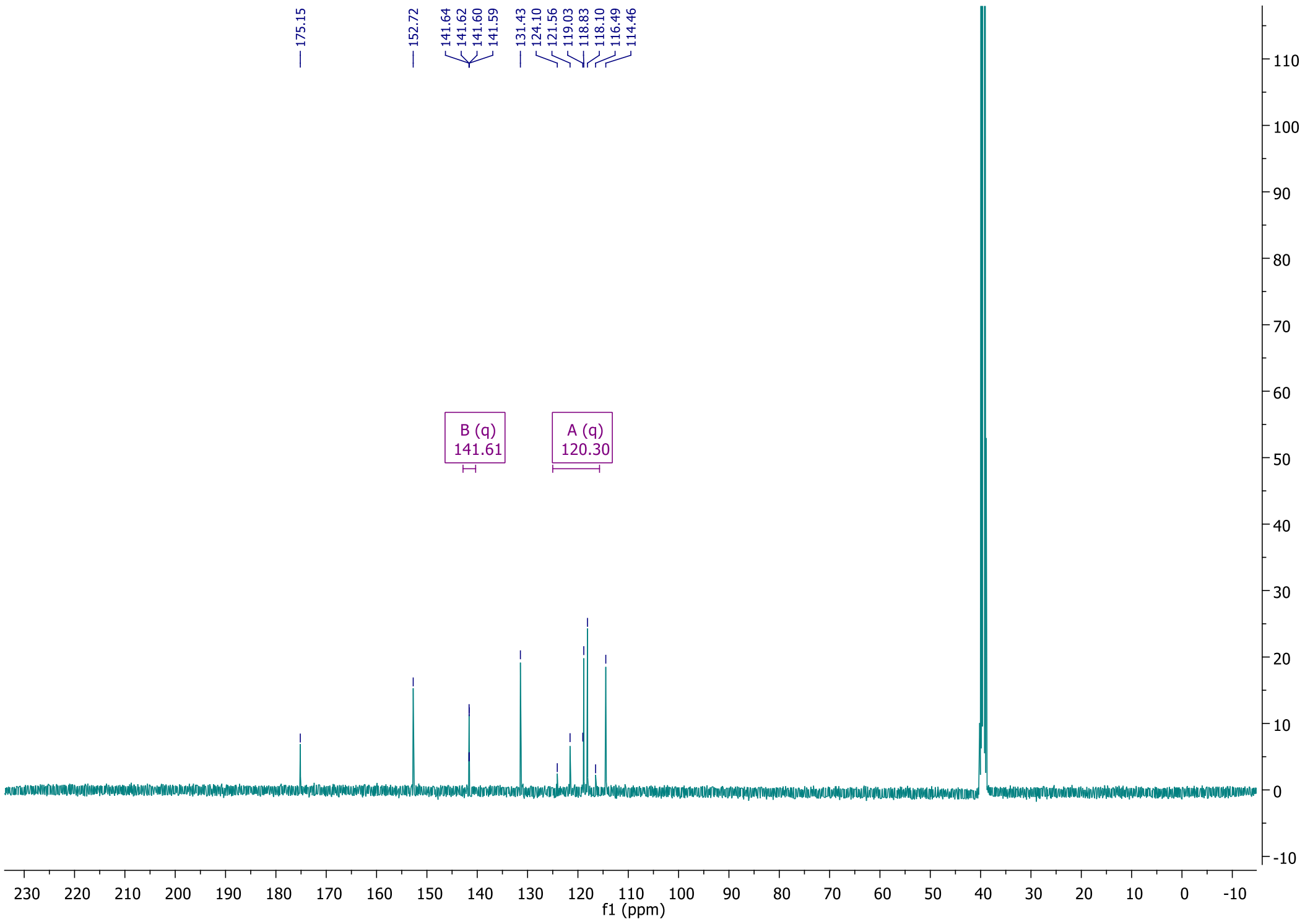

^{13}C-NMR of final compound **8** (DMSO-d_6).

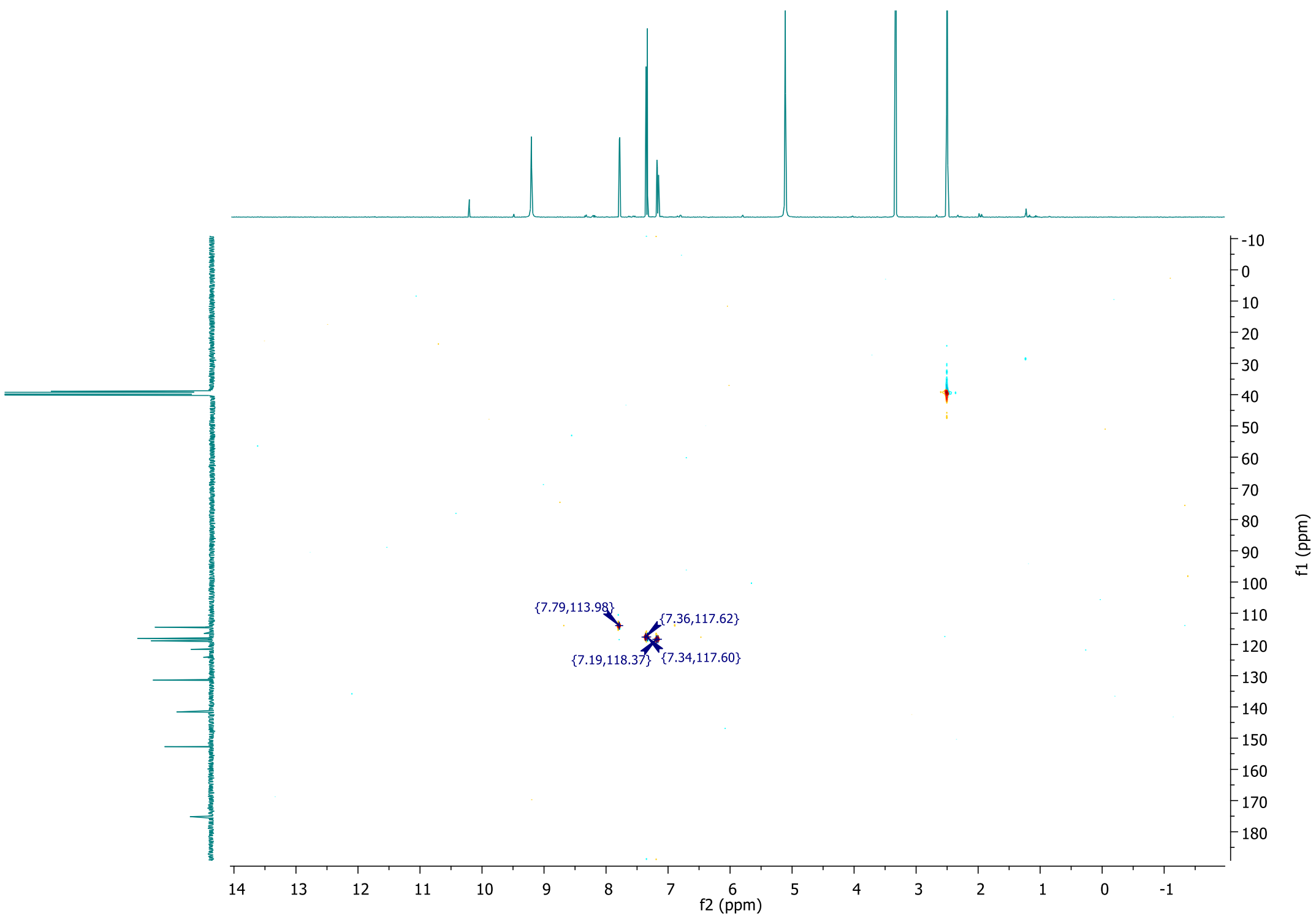

gHSQCAD of final compound **8** (DMSO-d_6).

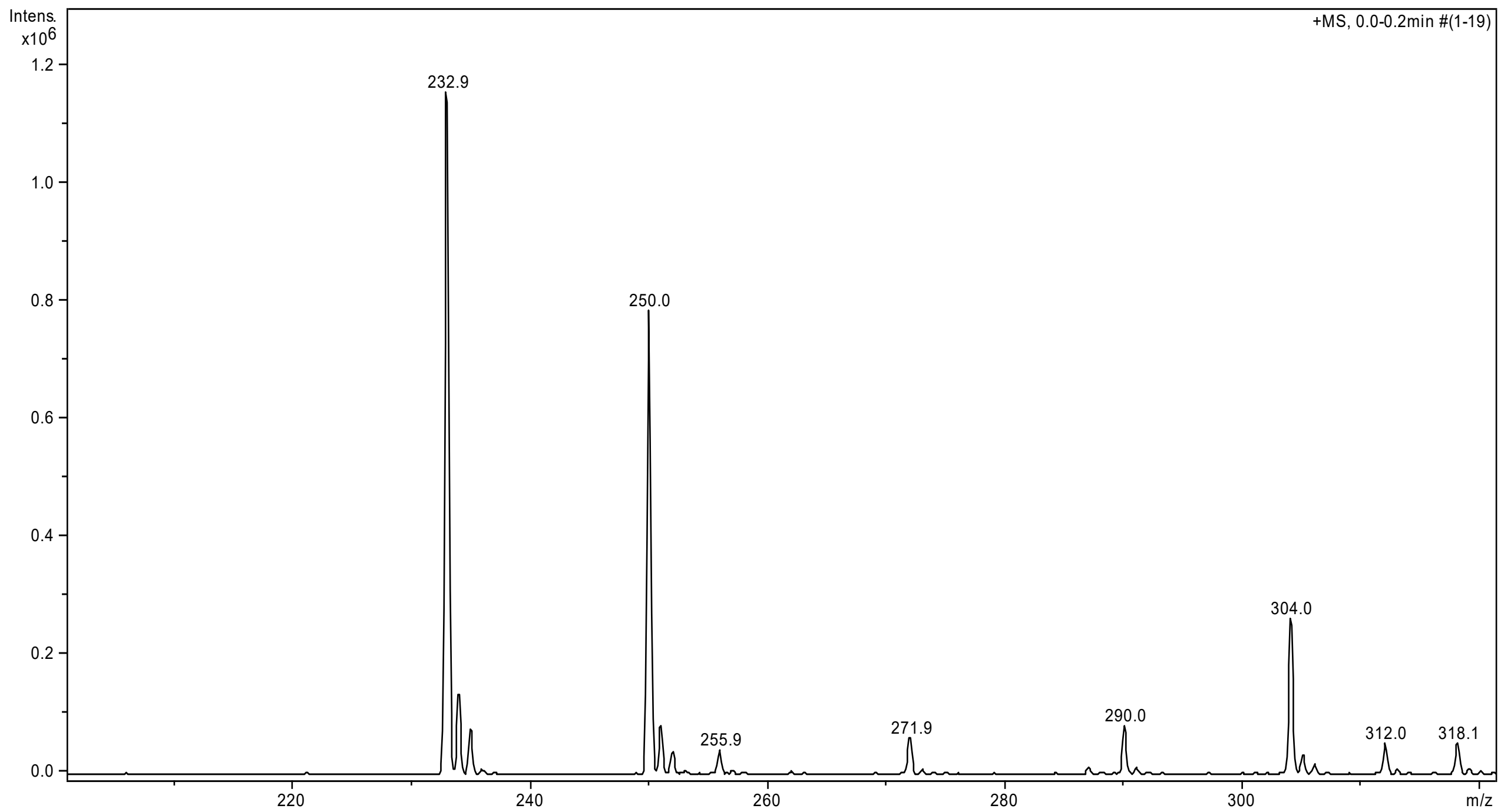

ES-MS of final compound **8** (MeOH).

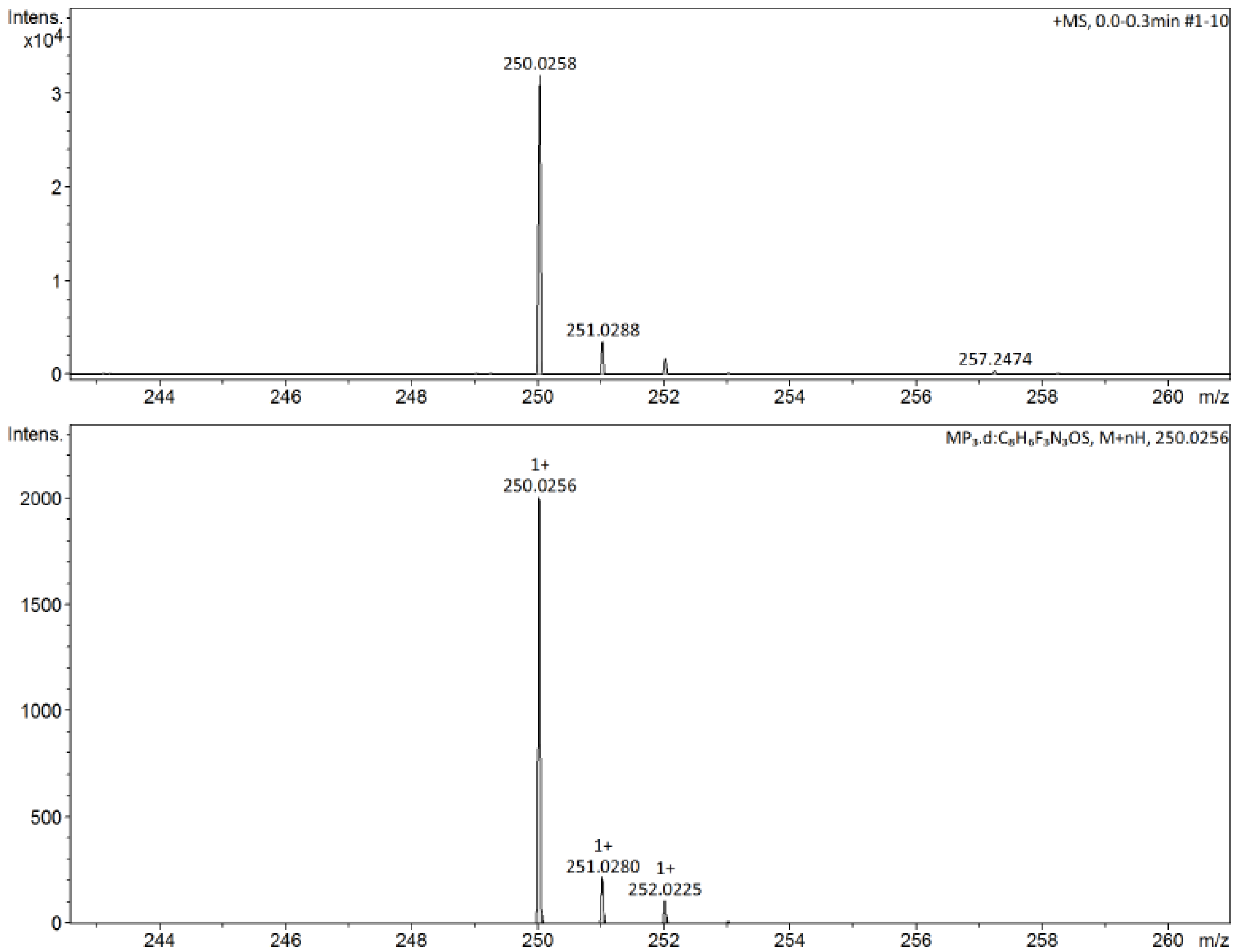

HRMS of final compound **8** (MeOH).

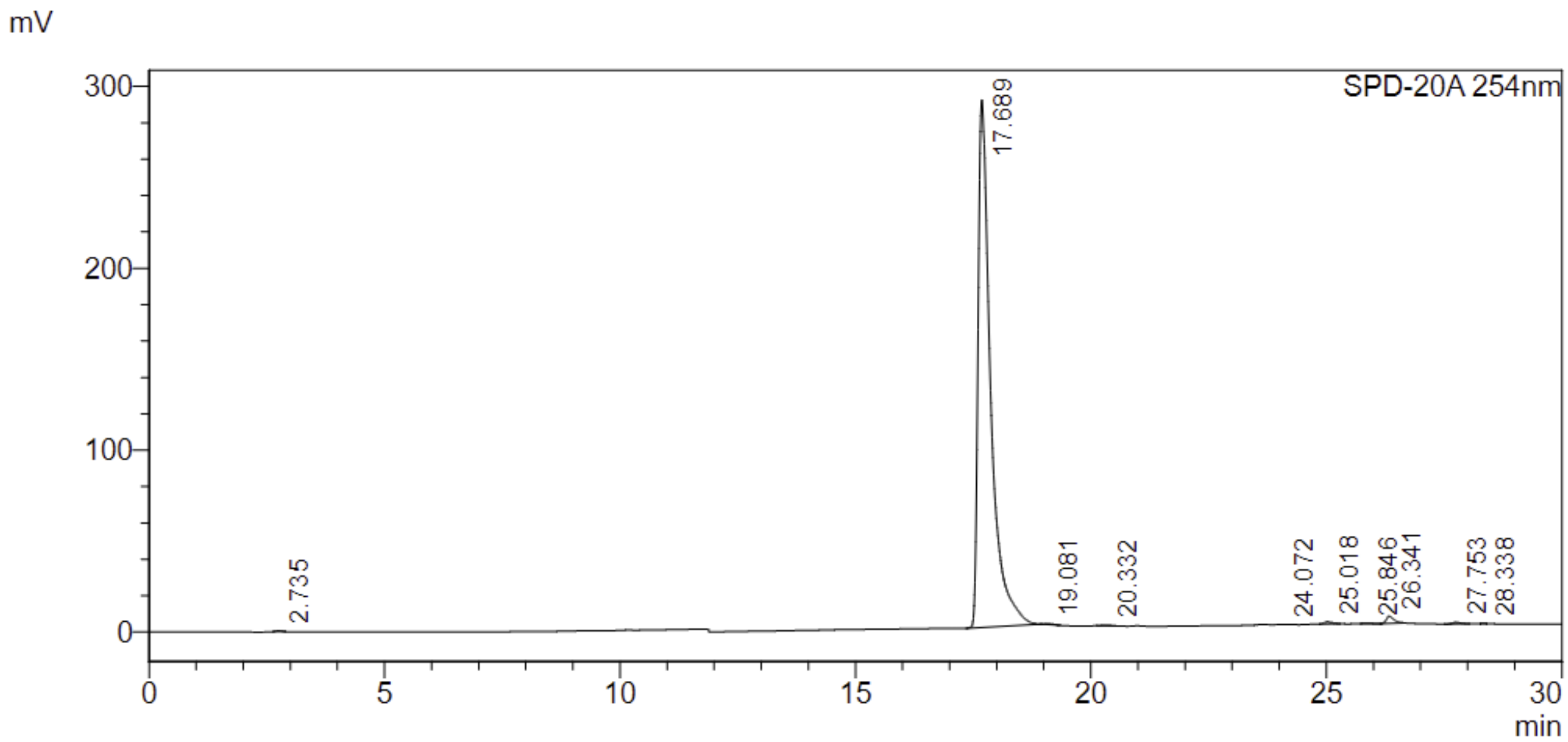

<Peak Table>

SPD-20A 254nm

Peak#	Ret. Time	Area	Height	Conc.	Unit	Mark	Name
1	2.735	4640	591	0.086		M	
2	17.689	5292614	289958	98.395		M	
3	19.081	5689	474	0.106		M	
4	20.332	5106	388	0.095		M	
5	24.072	6	3	0.000		M	
6	25.018	13015	1093	0.242		M	
7	25.846	1771	152	0.033		M	
8	26.341	45953	3951	0.854		M	
9	27.753	9737	823	0.181		M	
10	28.338	424	84	0.008		M	
Total		5378955	297518				

HPLC of final compound **8** (flow: 1 ml/min, eleunt gradient: from water-methanol 55:45 to 15:85 in 30 min; detector: 254 nm).

N,O-dimethyl-N-(6-(trifluoromethoxy)benzo[d]thiazol-2-yl)hydroxylamine (**9**)

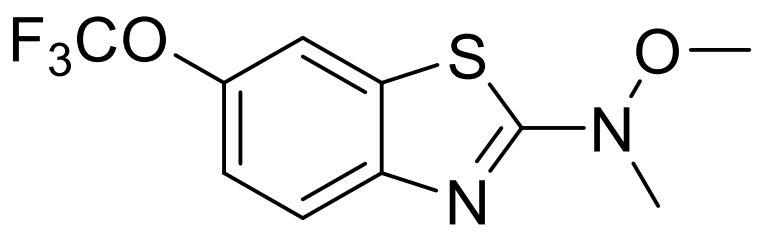

Chemical Formula: $C_{10}H_9F_3N_2O_2S$
Exact Mass: 278.03
Molecular Weight: 278.25

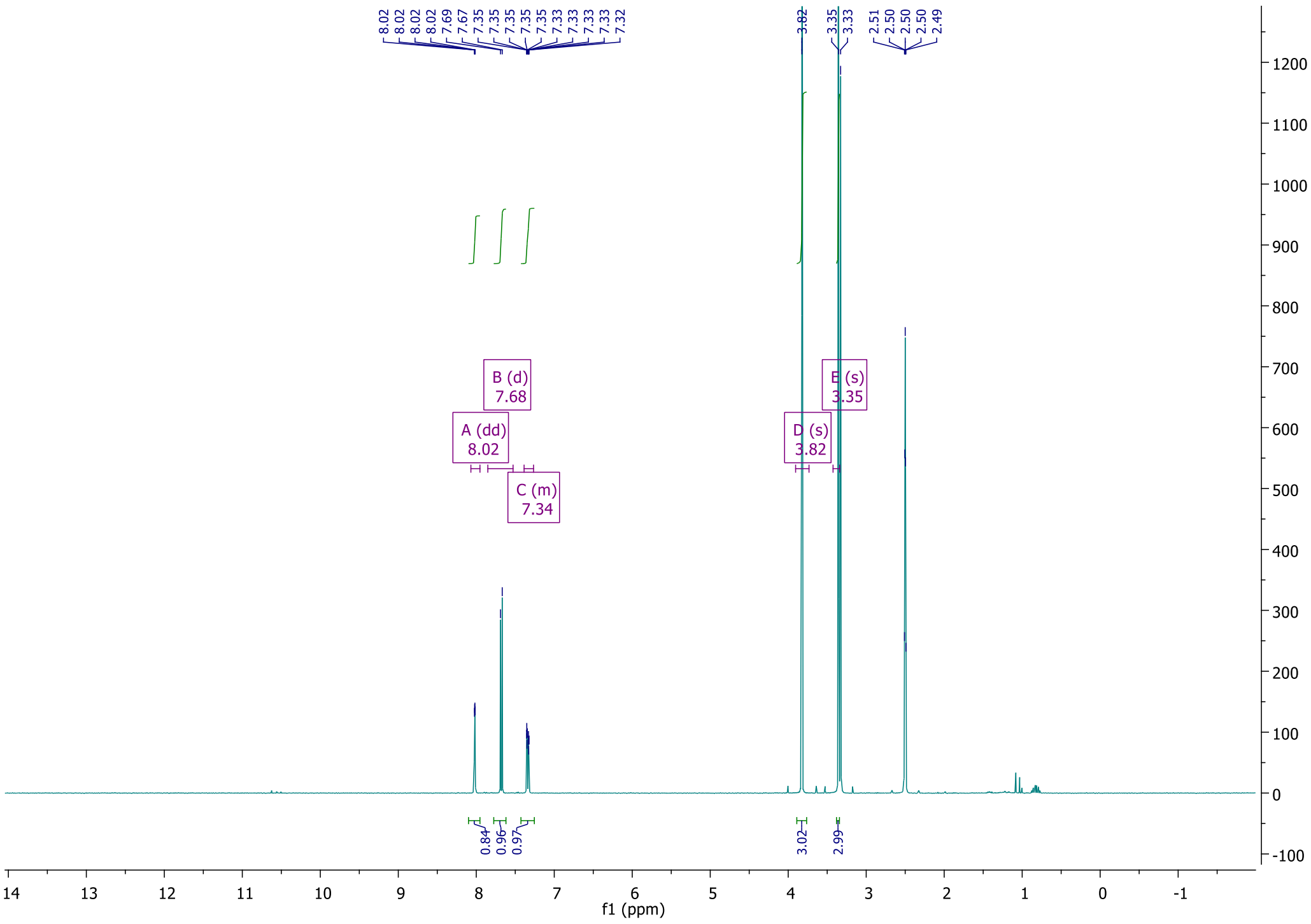

^{1}H-NMR of final compound **9** (DMSO-*d₆*).

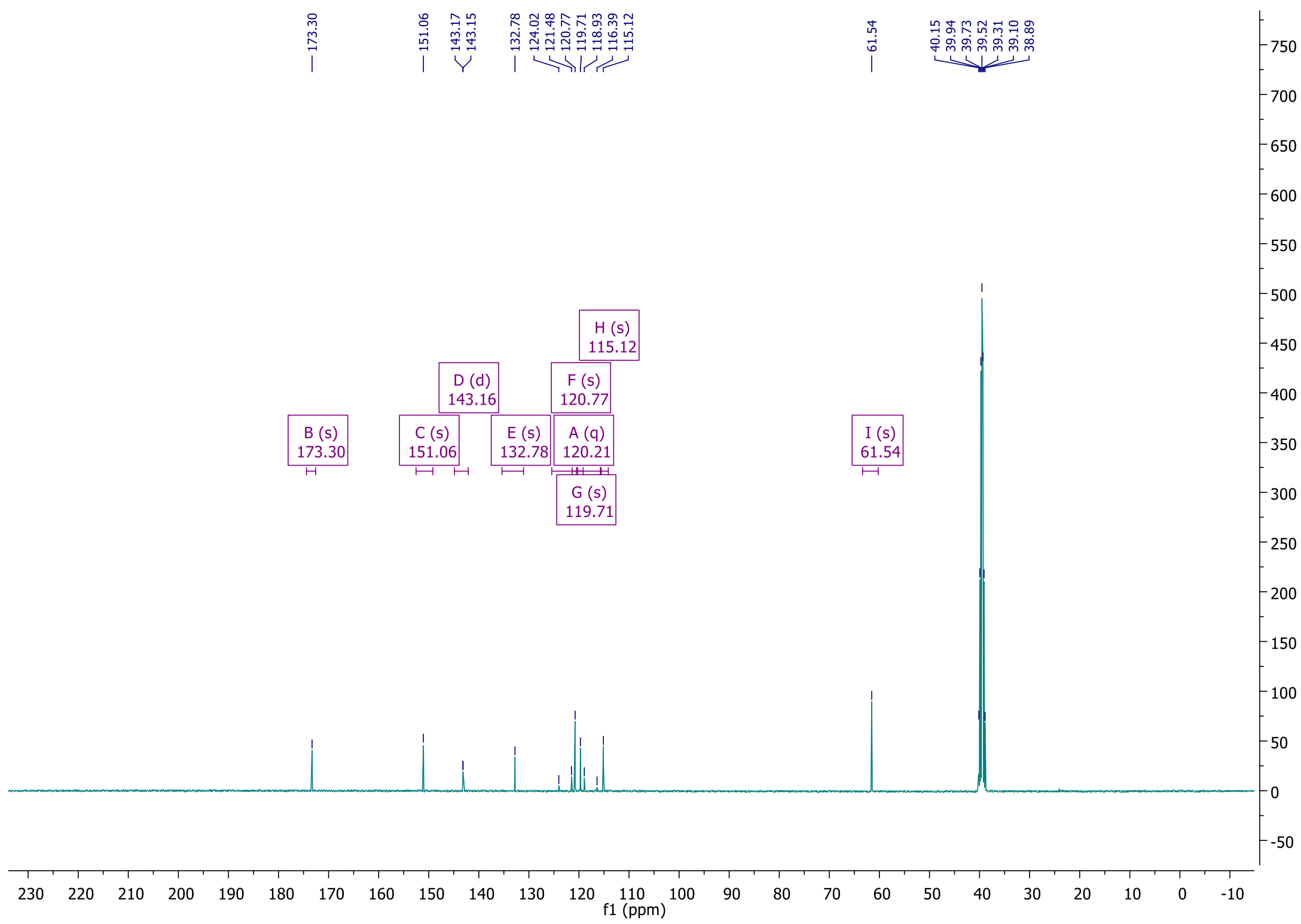

^{13}C-NMR of final compound **9** (DMSO-d_6).

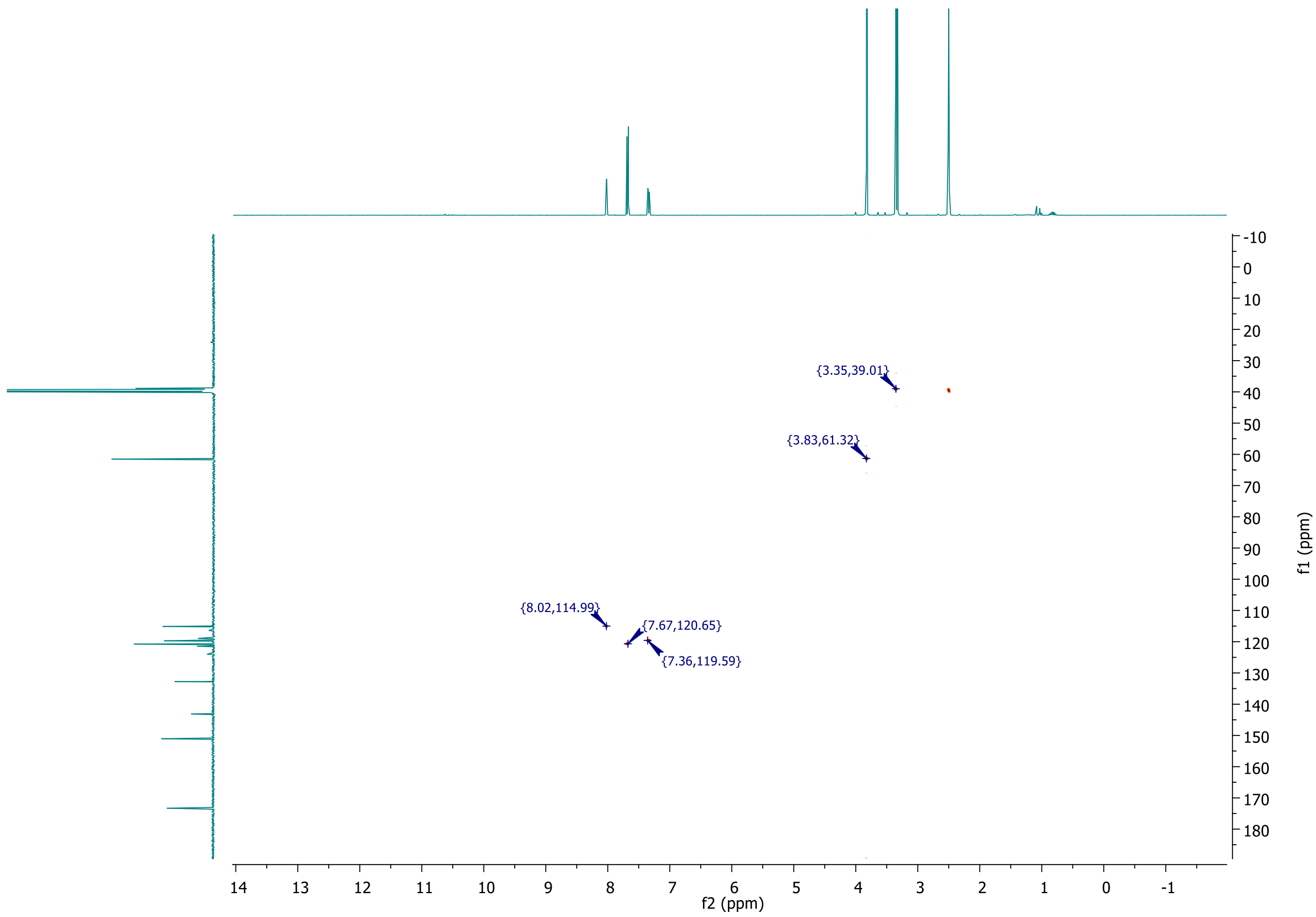

gHSQCAD of final compound **9** (DMSO-d_6).

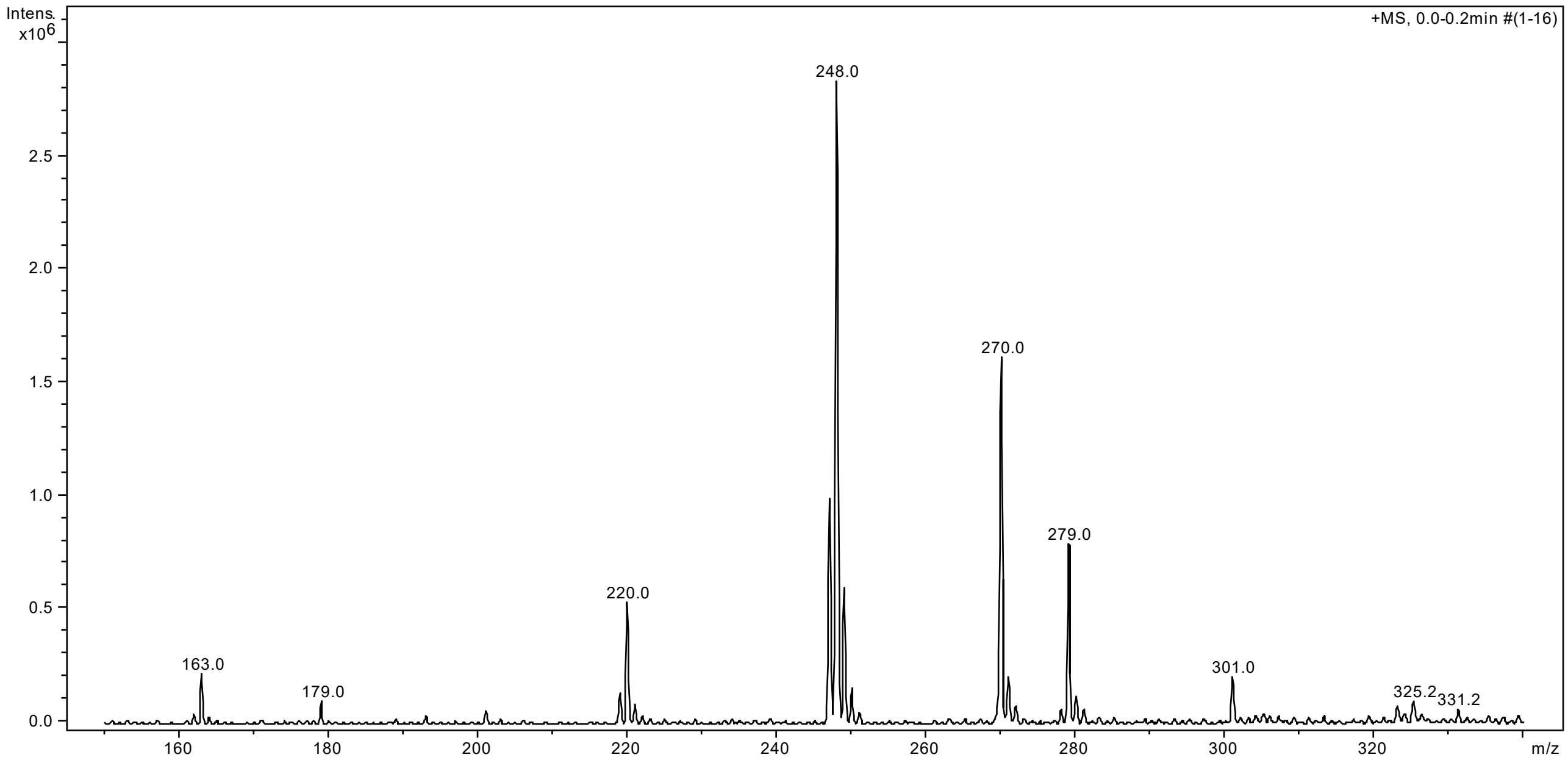

ES-MS of final compound **9** (MeOH).

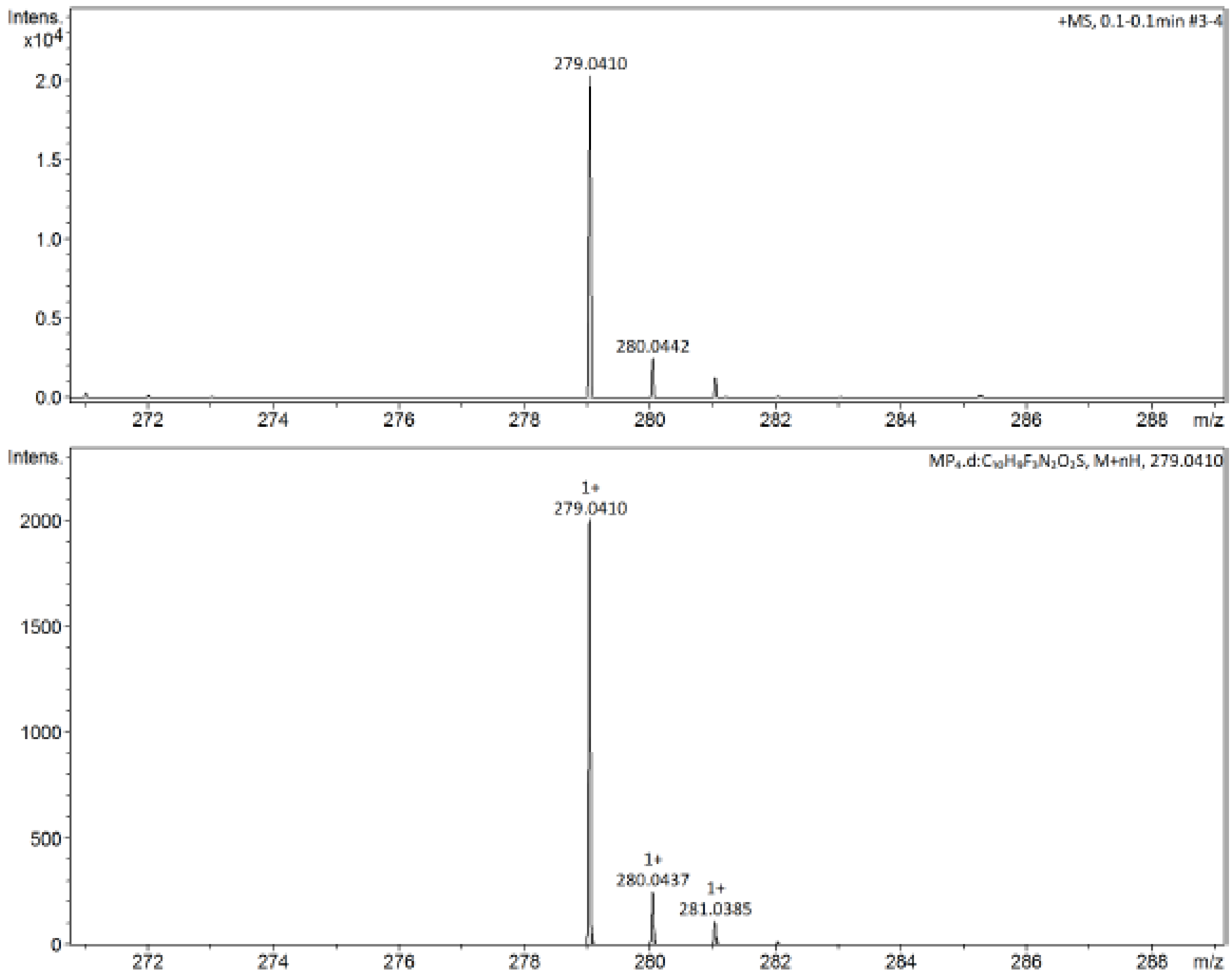

HRMS of final compound **9** (MeOH).

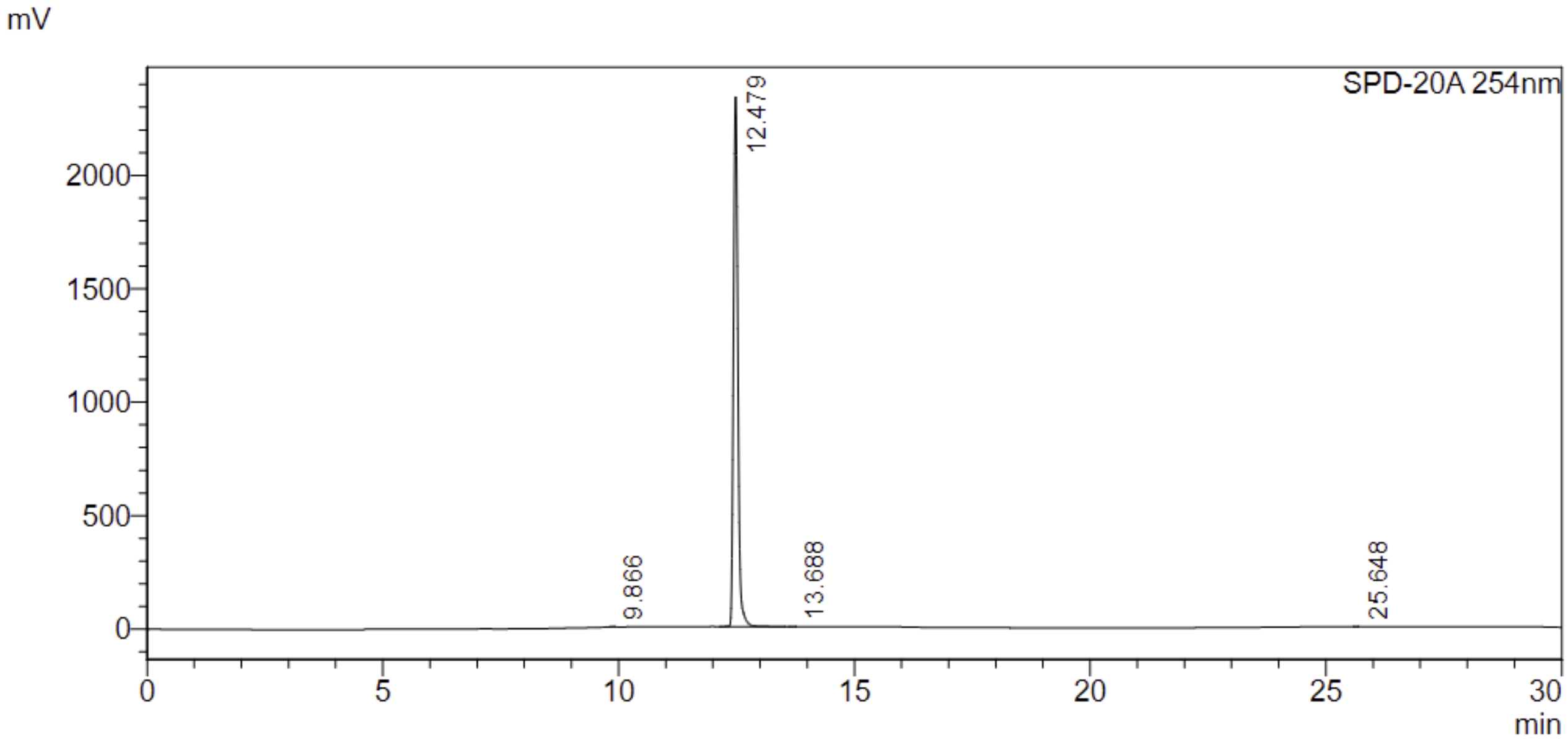

<Peak Table>

SPD-20A 254nm

Peak#	Ret. Time	Area	Height	Conc.	Unit	Mark	Name
1	9.866	9818	1635	0.064		M	
2	12.479	15364286	2334016	99.931		M	
3	13.688	752	148	0.005		M	
4	25.648	85	25	0.001		M	
Total		15374941	2335824				

HPLC of final compound **9** (flow: 1 ml/min, eleunt gradient: from water-methanol 55:45 to 15:85 in 30 min; detector: 254 nm).

N-methyl-6-(trifluoromethoxy)benzo[d]thiazol-2-amine (**10**)

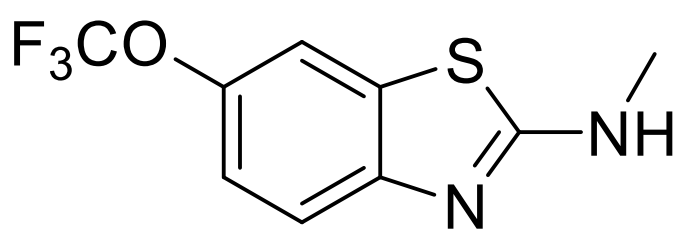

Chemical Formula: $C_9H_7F_3N_2OS$
Exact Mass: 248.02
Molecular Weight: 248.22

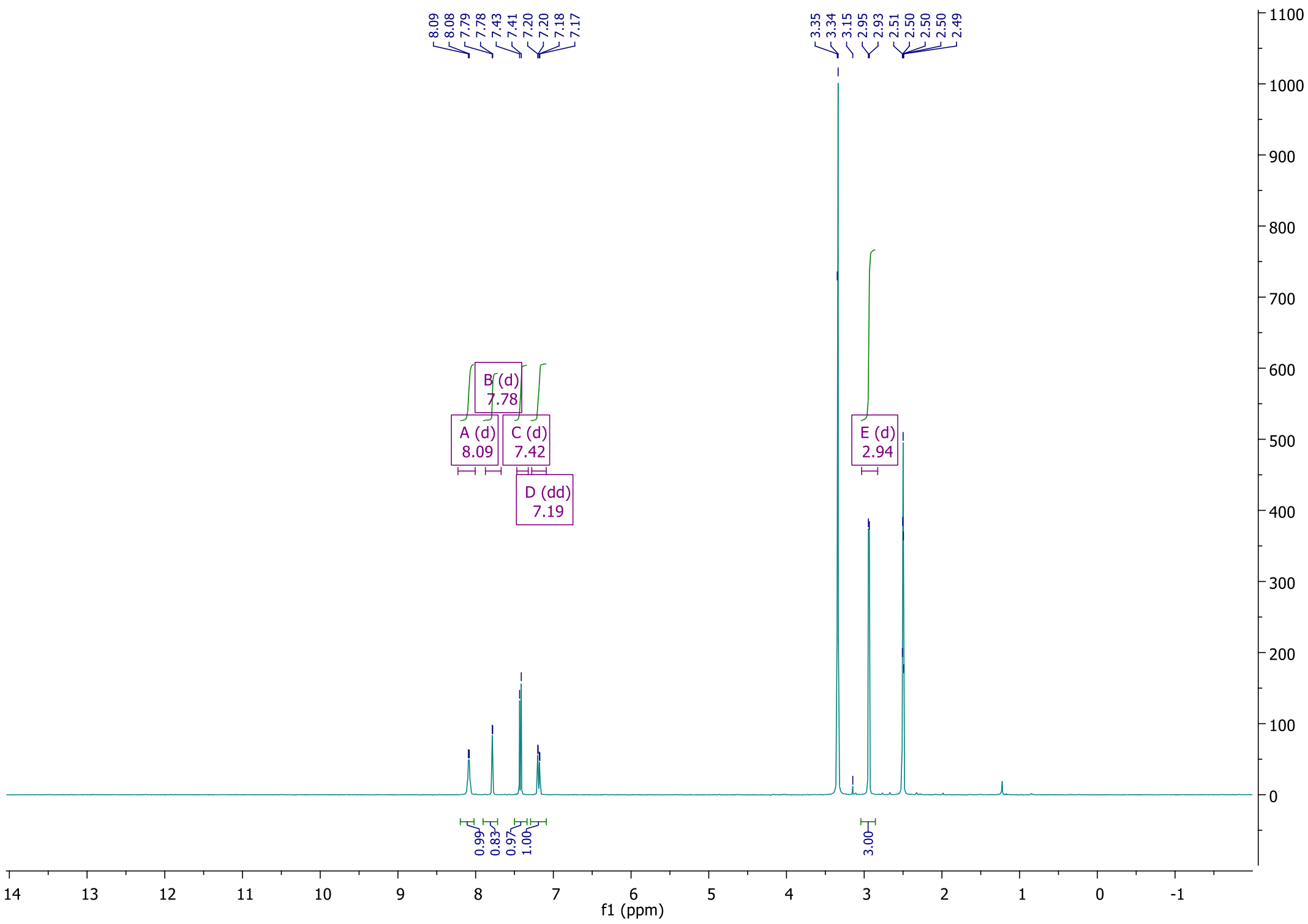

^{1}H-NMR of final compound **10** (DMSO-d_6).

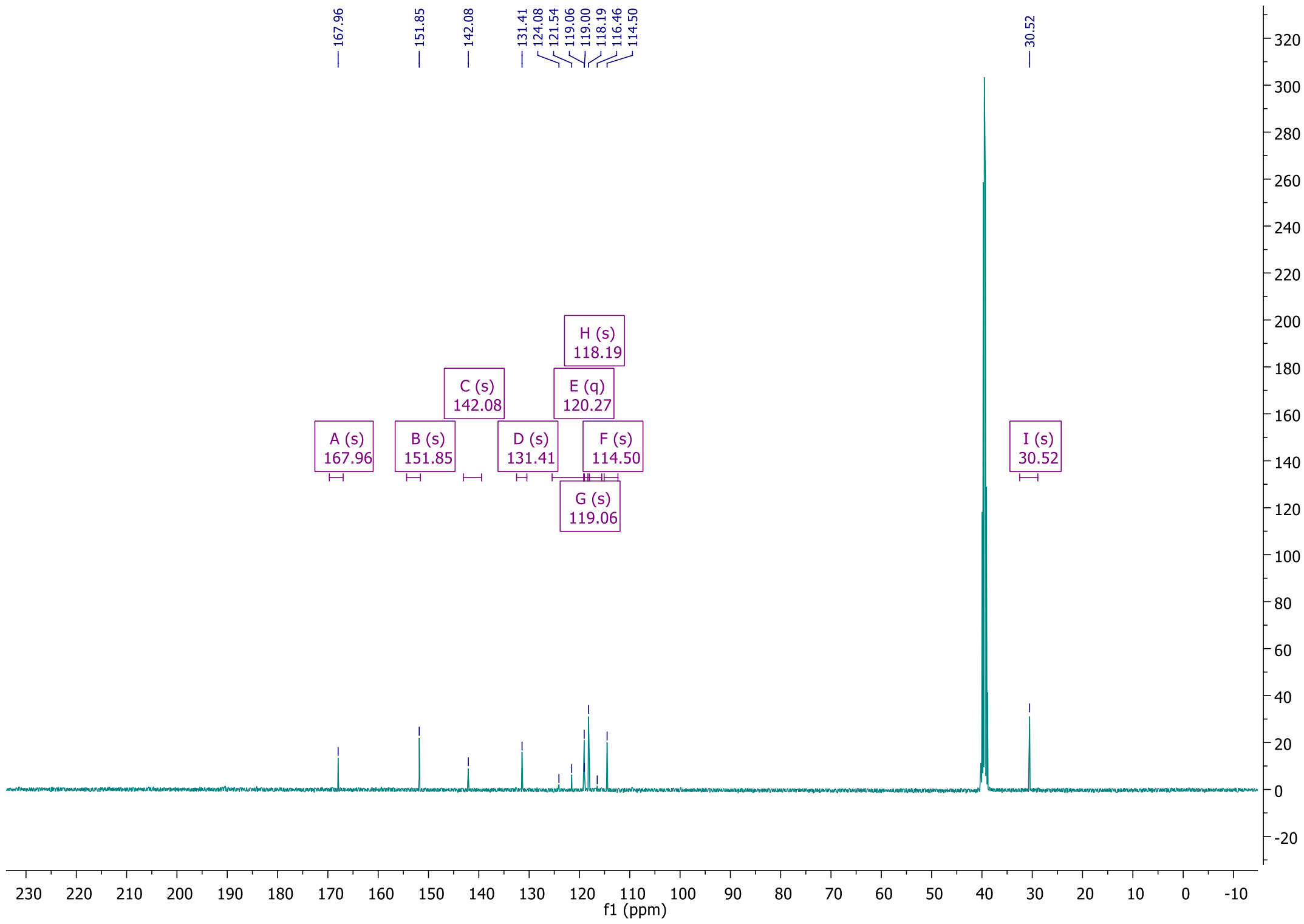

^{13}C-NMR of final compound **10** (DMSO-d_6).

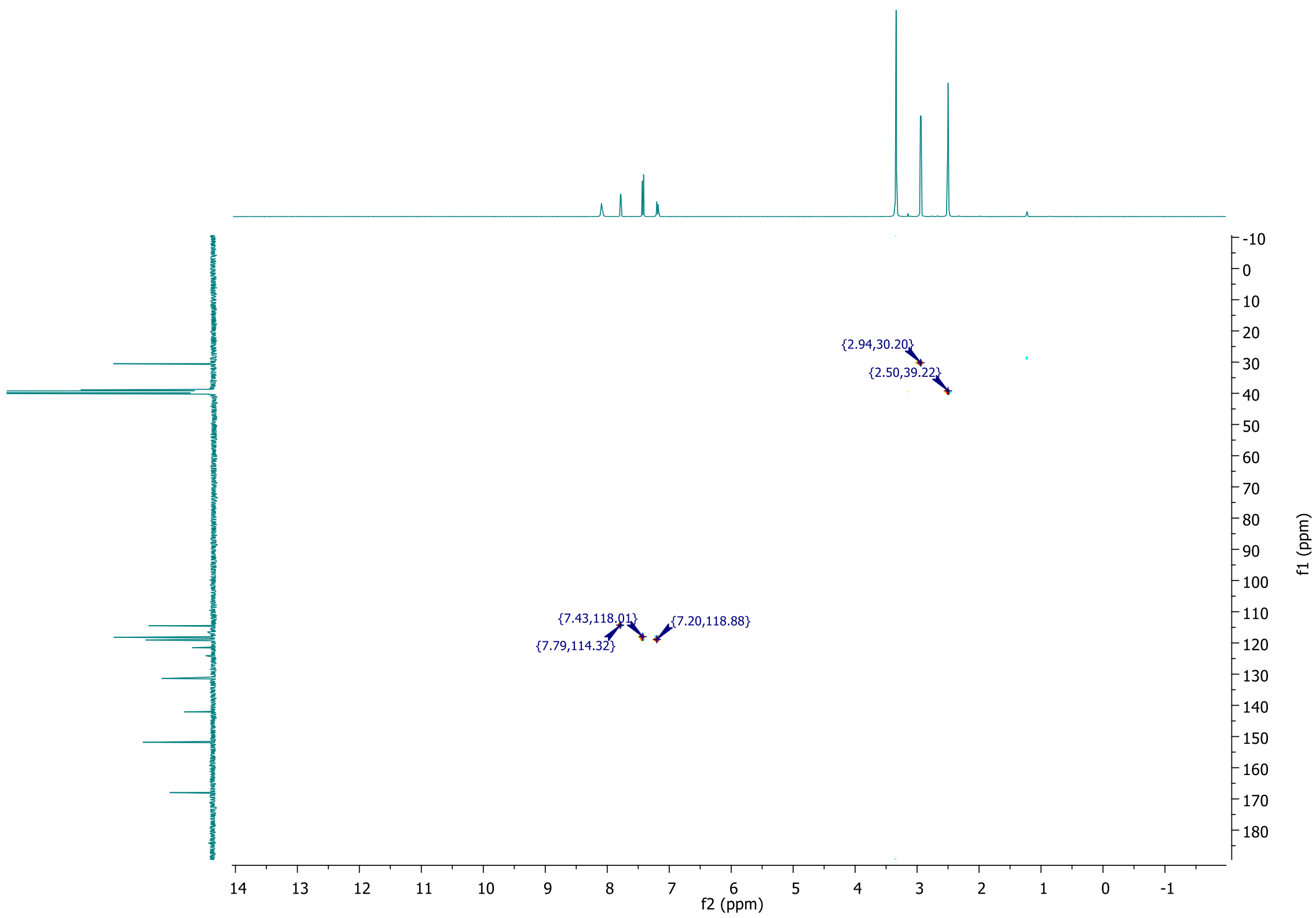

gHSQCAD of final compound **10** (DMSO-d_6).

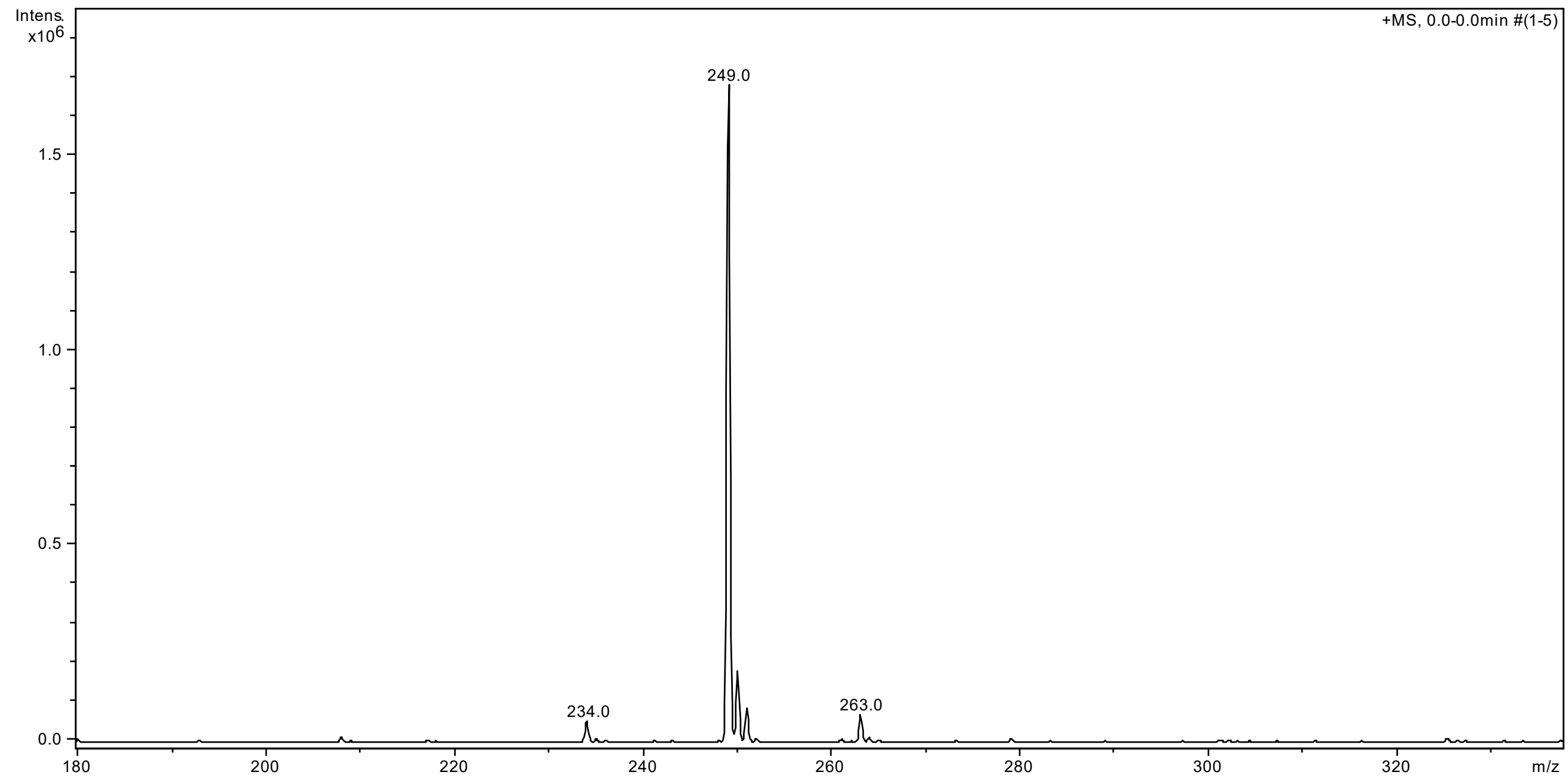

ES-MS of final compound **10** (MeOH).

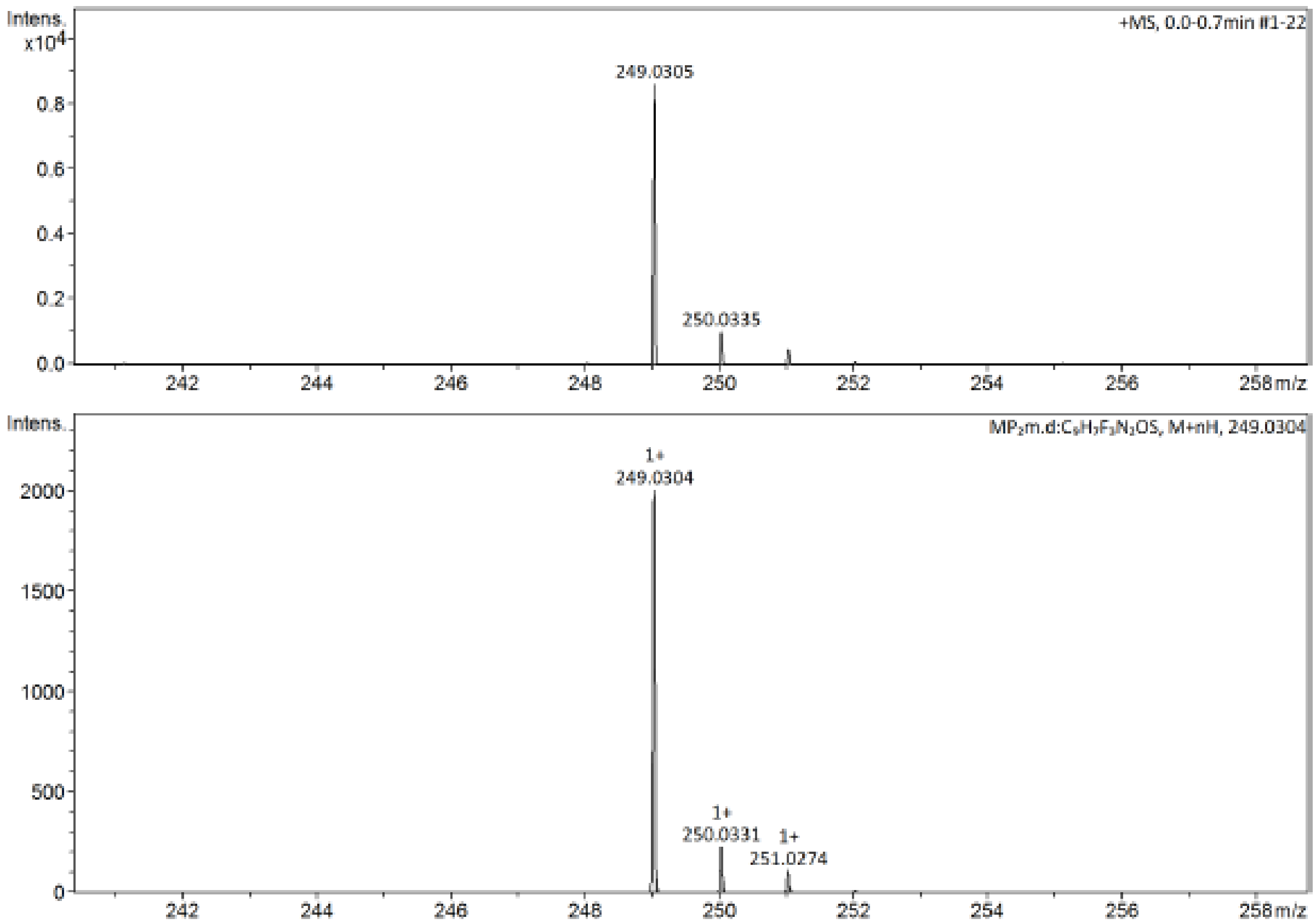

HRMS of final compound **10** (MeOH).

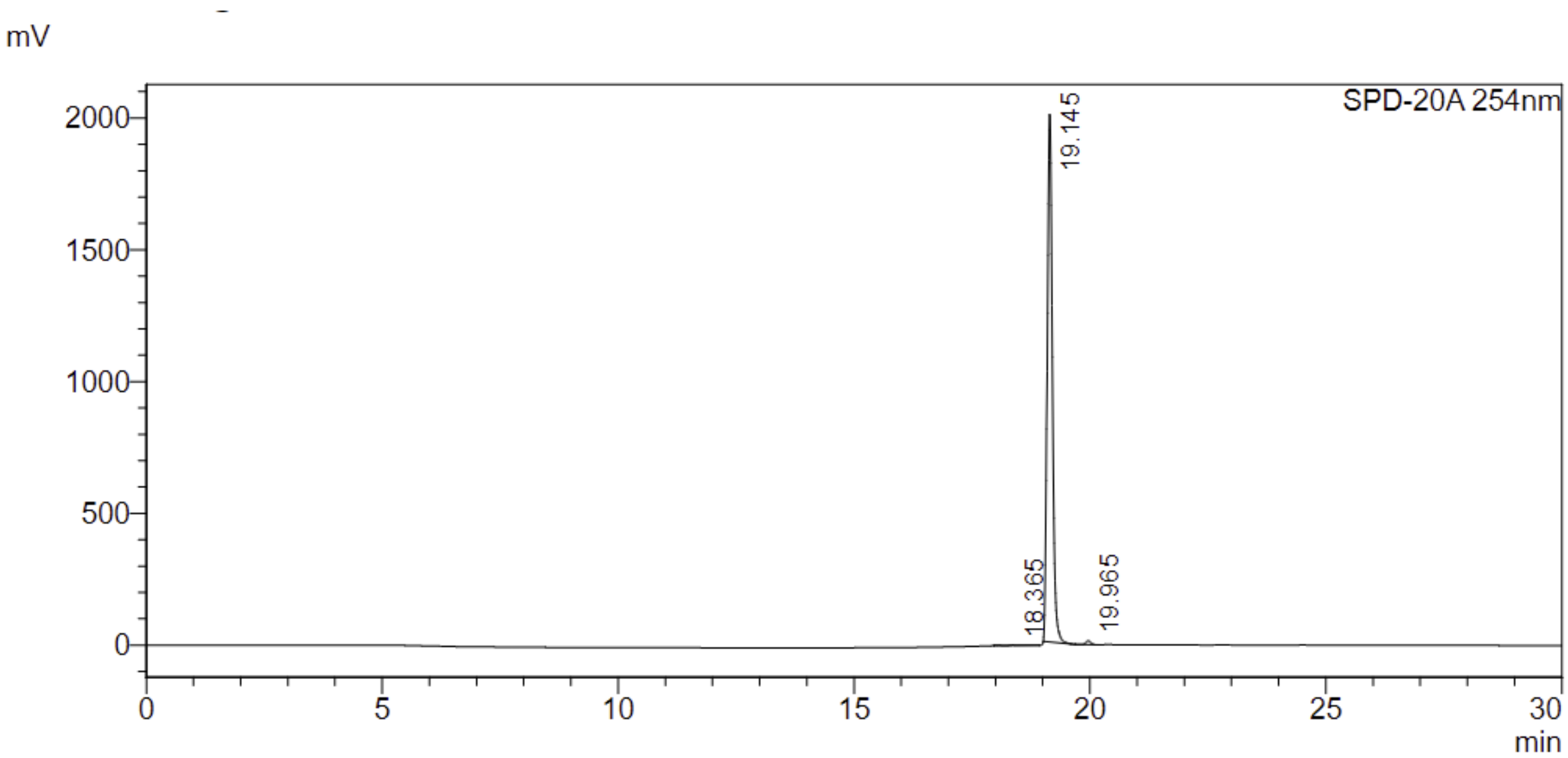

<Peak Table>

SPD-20A 254nm

Peak#	Ret. Time	Area	Height	Conc.	Unit	Mark	Name
1	18.365	7232	1168	0.048		M	
2	19.145	14832338	2001501	99.296		M	
3	19.965	97968	15038	0.656		M	
Total		14937538	2017707				

HPLC of final compound **10** (flow: 1 ml/min, eleunt gradient: from water-methanol 55:45 to 15:85 in 30 min; detector: 254 nm).

O-methyl-N-(6-(trifluoromethoxy)benzo[d]thiazol-2-yl)hydroxylamine (**11**)

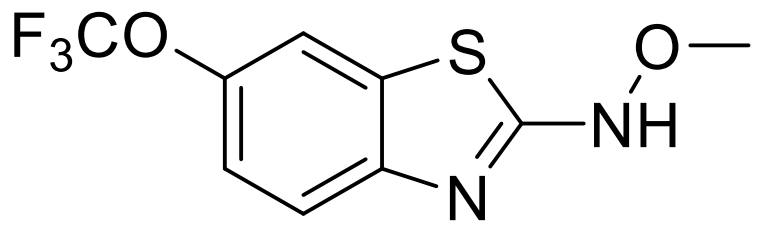

Chemical Formula: $C_9H_7F_3N_2O_2S$
Exact Mass: 264.02
Molecular Weight: 264.22

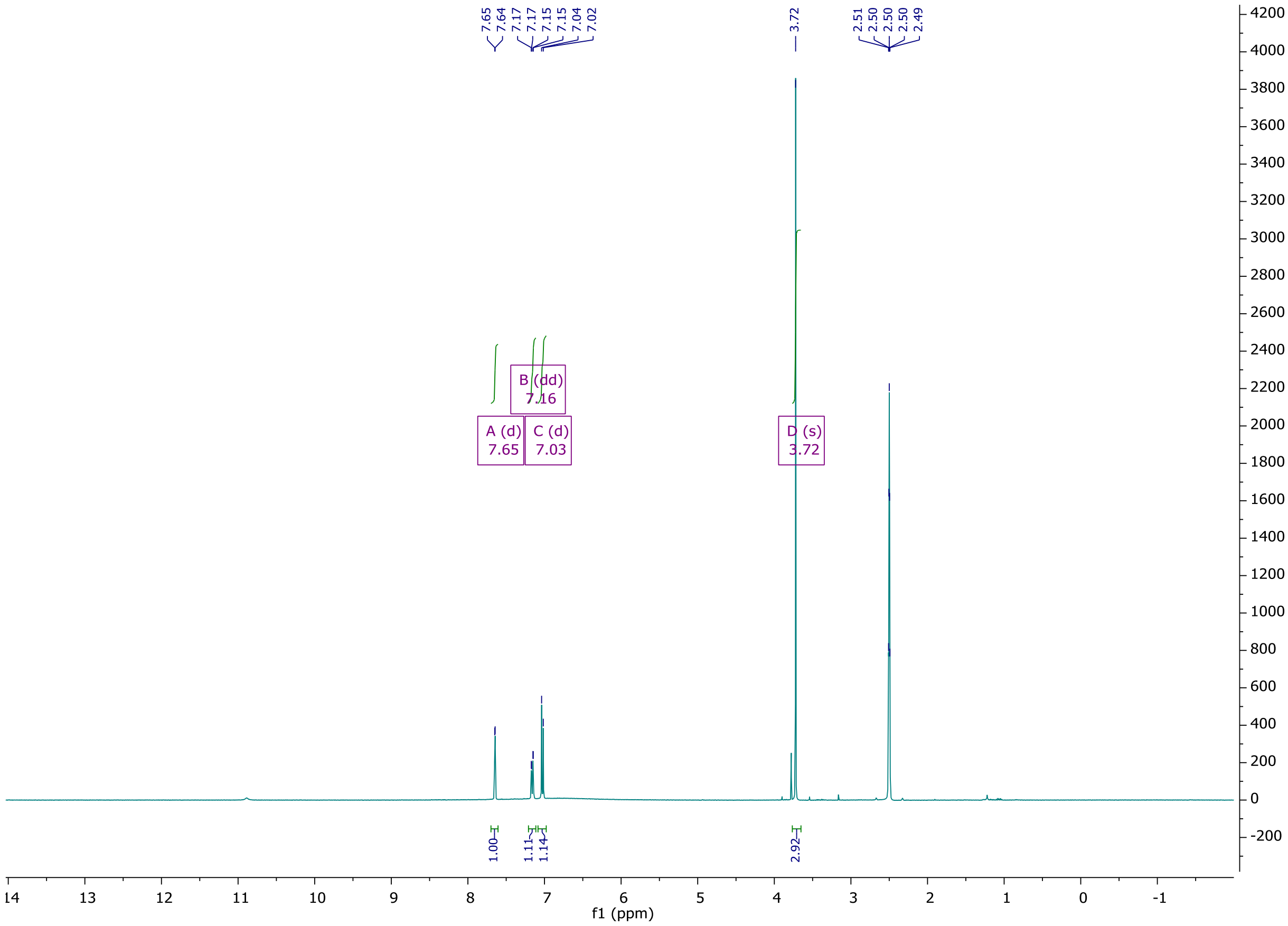

^{1}H-NMR of final compound **11** (DMSO-d_6).

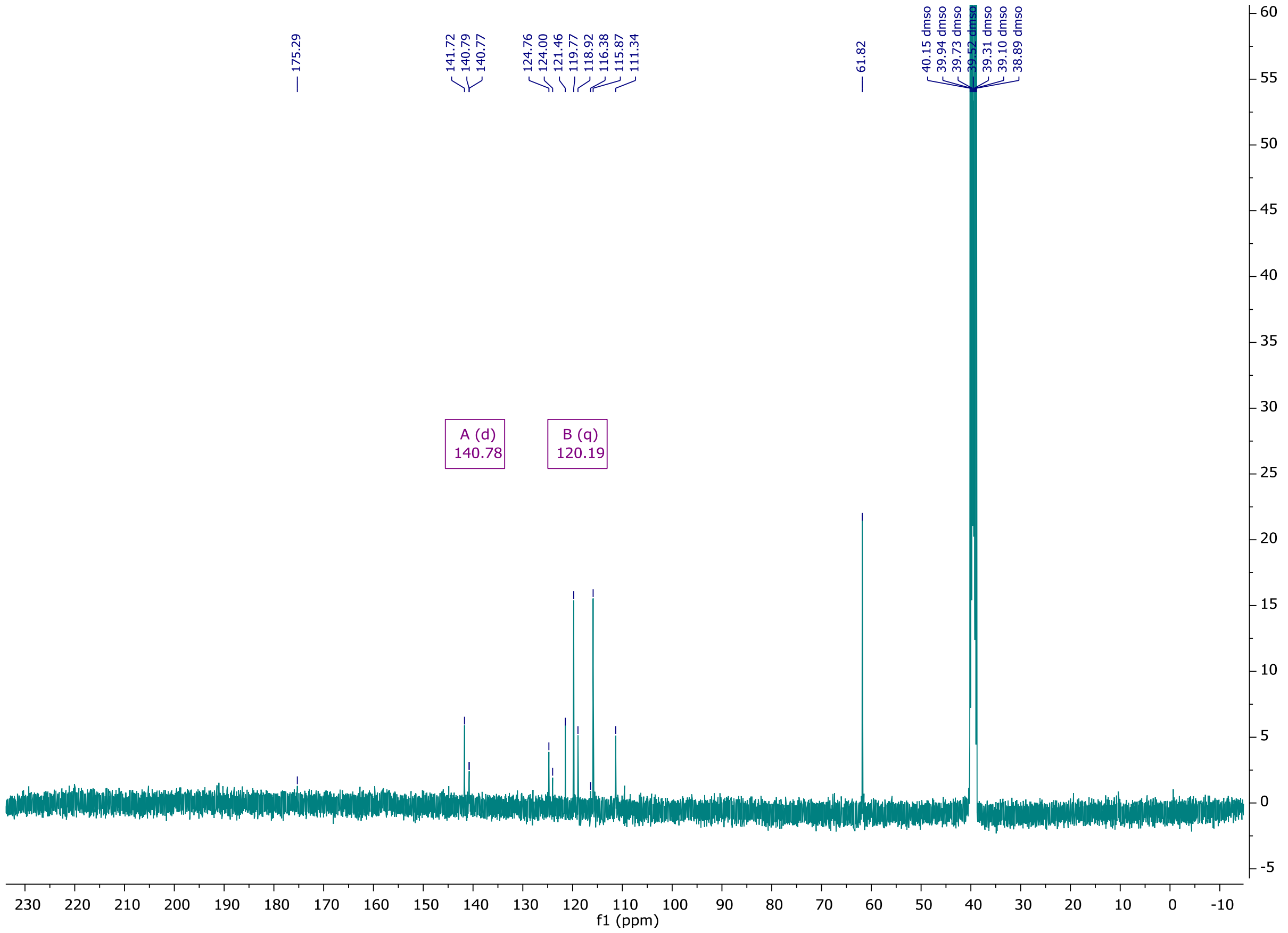

^{13}C-NMR of final compound **11** (DMSO-d_6).

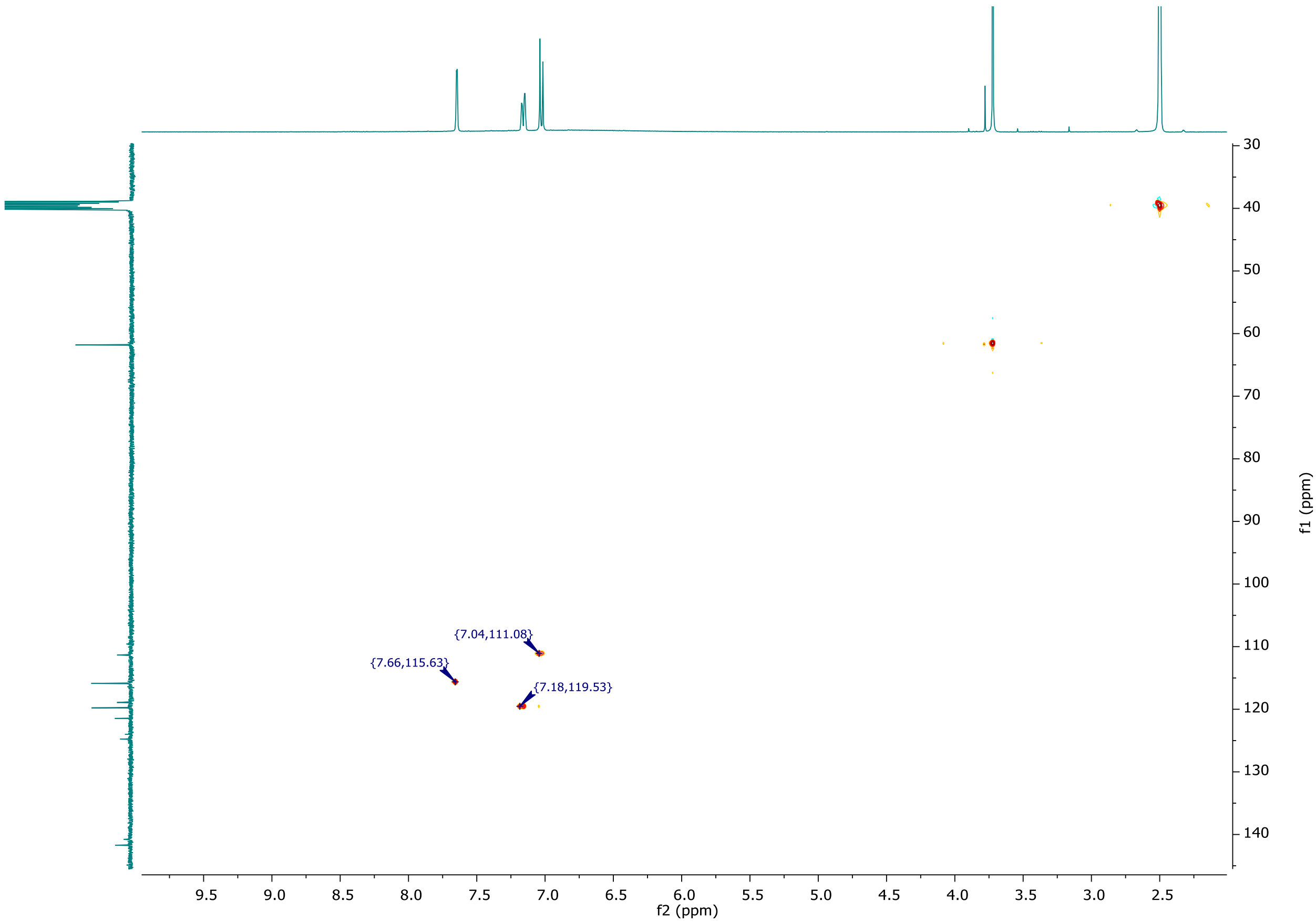

gHSQCAD of final compound **11** (DMSO-d_6).

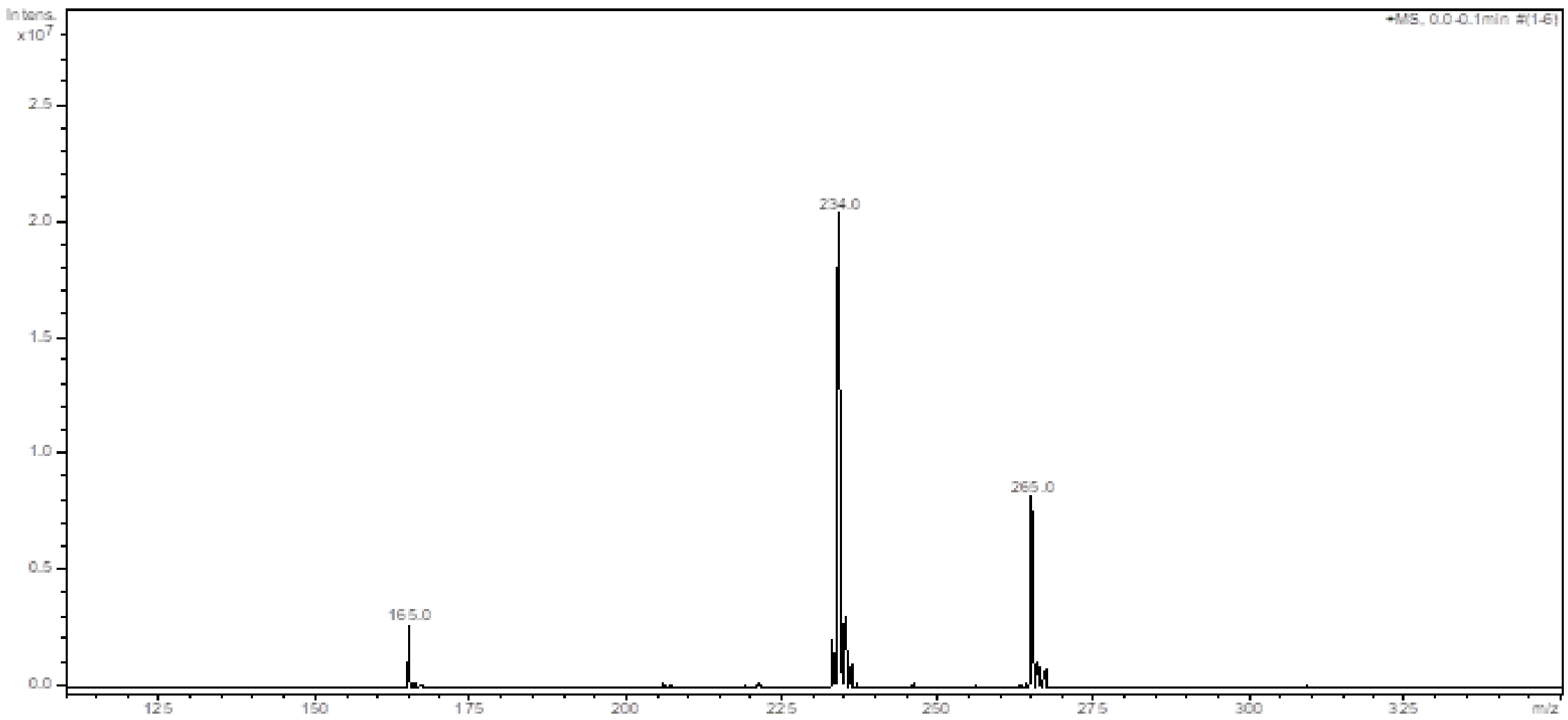

ES-MS of final compound **11** (MeOH).

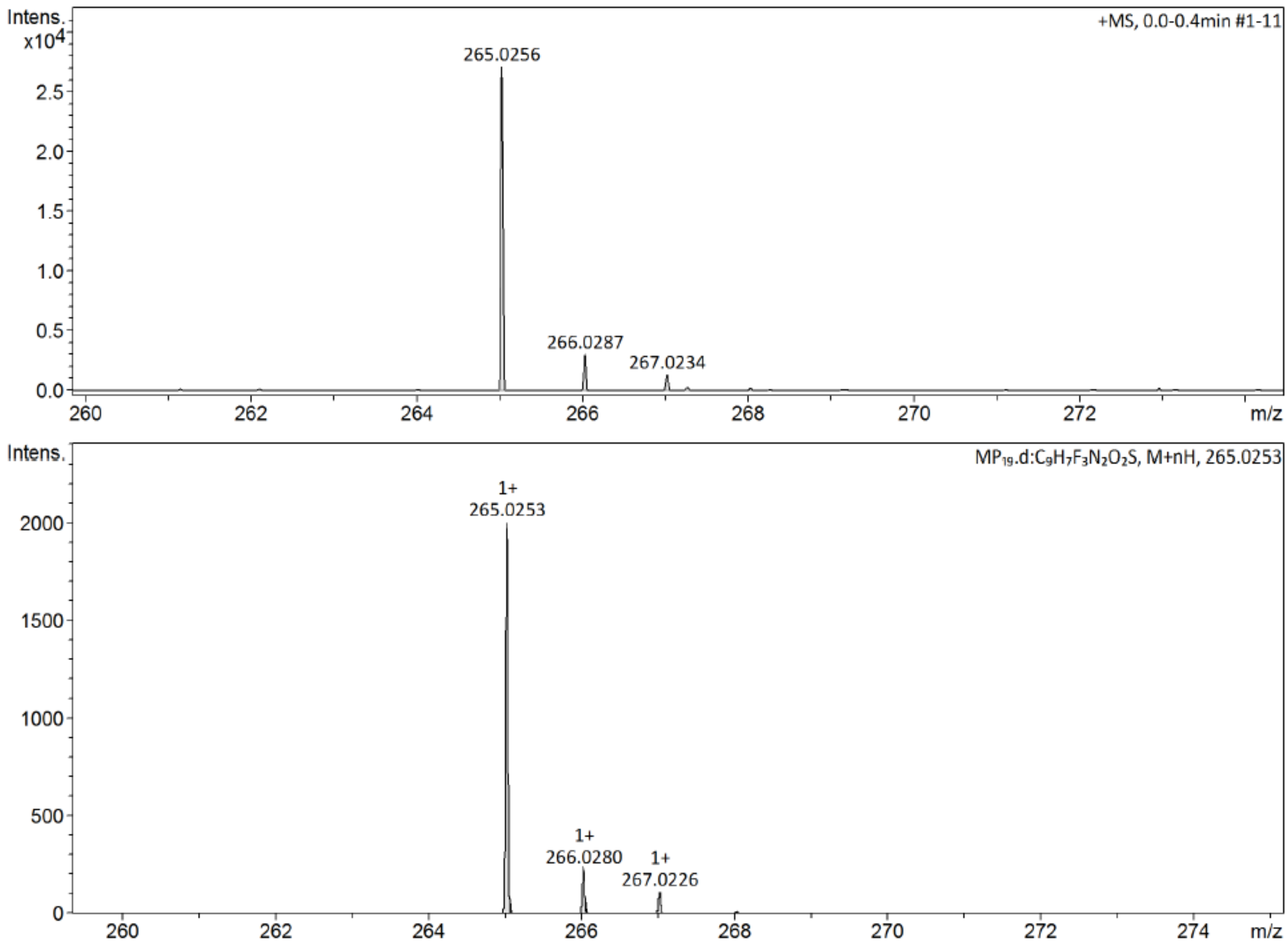

HRMS of final compound **11** (MeOH).

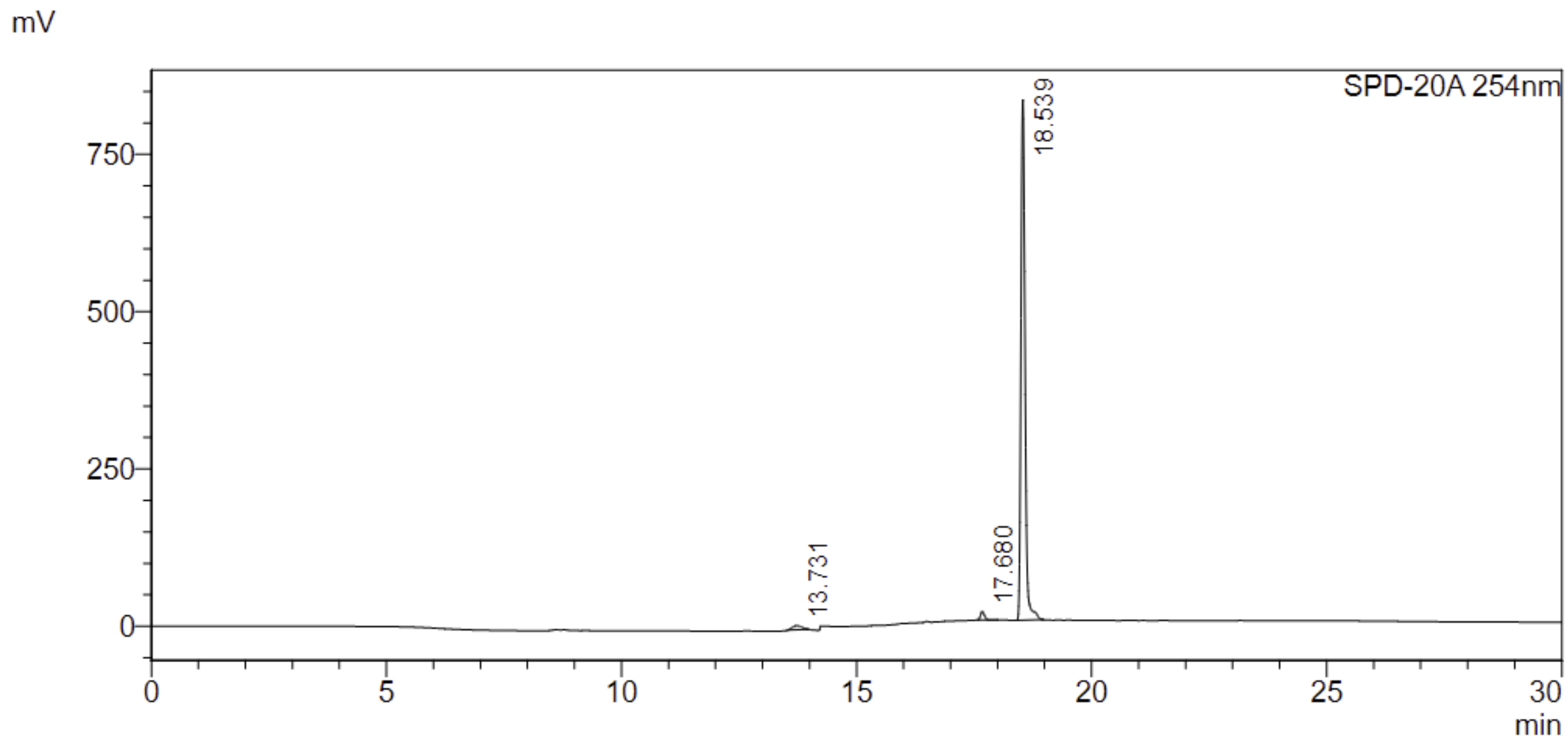

<Peak Table>

SPD-20A 254nm

Peak#	Ret. Time	Area	Height	Conc.	Unit	Mark	Name
1	13.731	89475	6372	1.729		M	
2	17.680	72257	13680	1.396		M	
3	18.539	5013411	826908	96.875		M	
Total		5175143	846960				

HPLC of final compound **11** (flow: 1 ml/min, eleunt gradient: from water-methanol 55:45 to 15:85 in 30 min; detector: 254 nm).

O-propyl-N-(6-(trifluoromethoxy)benzo[d]thiazol-2-yl)hydroxylamine (**12**)

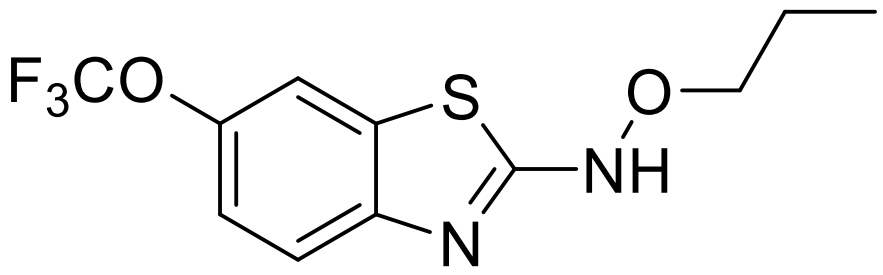

Chemical Formula: $C_{11}H_{11}F_3N_2O_2S$
Exact Mass: 292.05
Molecular Weight: 292.28

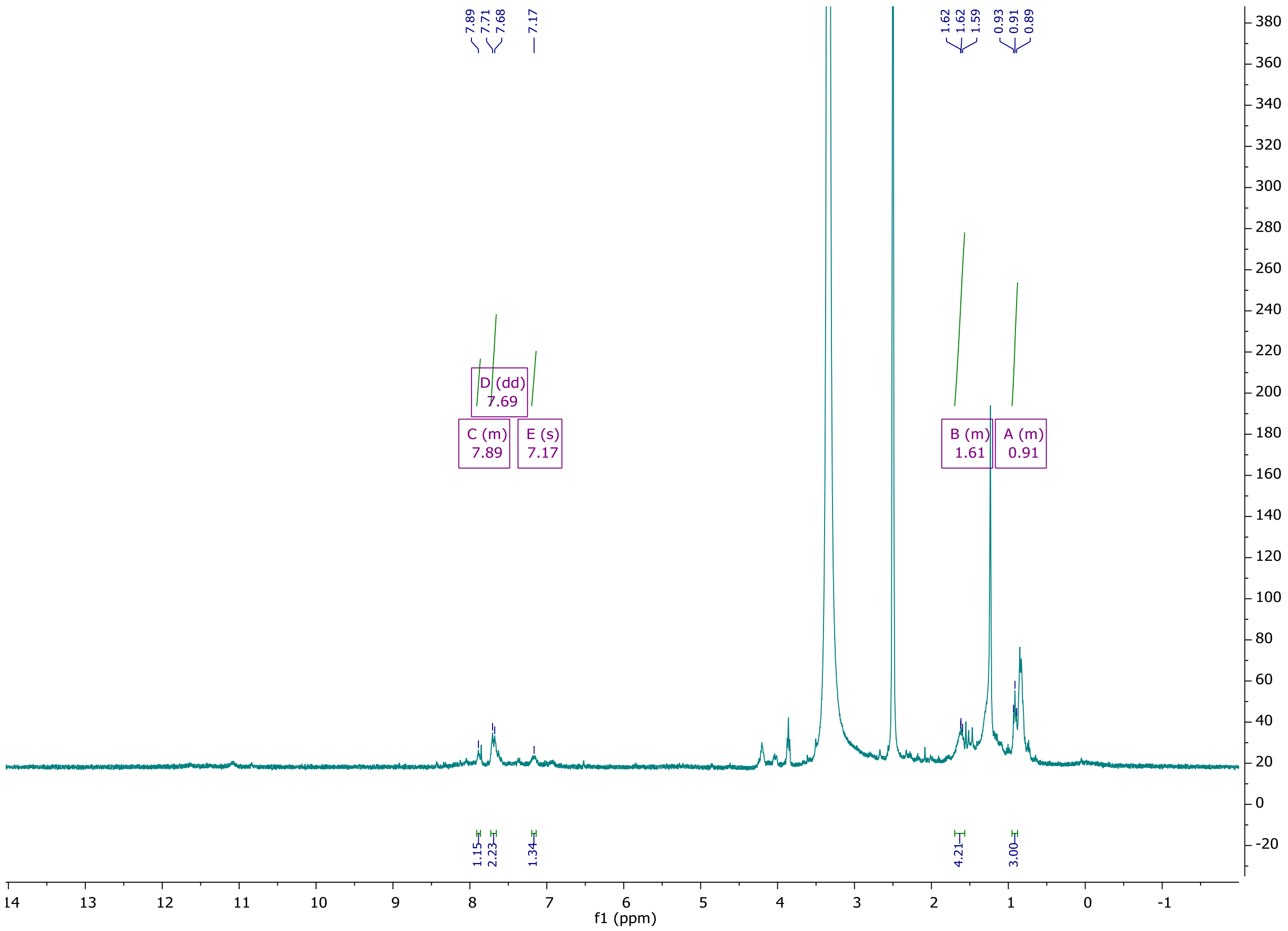

^{1}H-NMR of final compound **12** (DMSO-d_6).

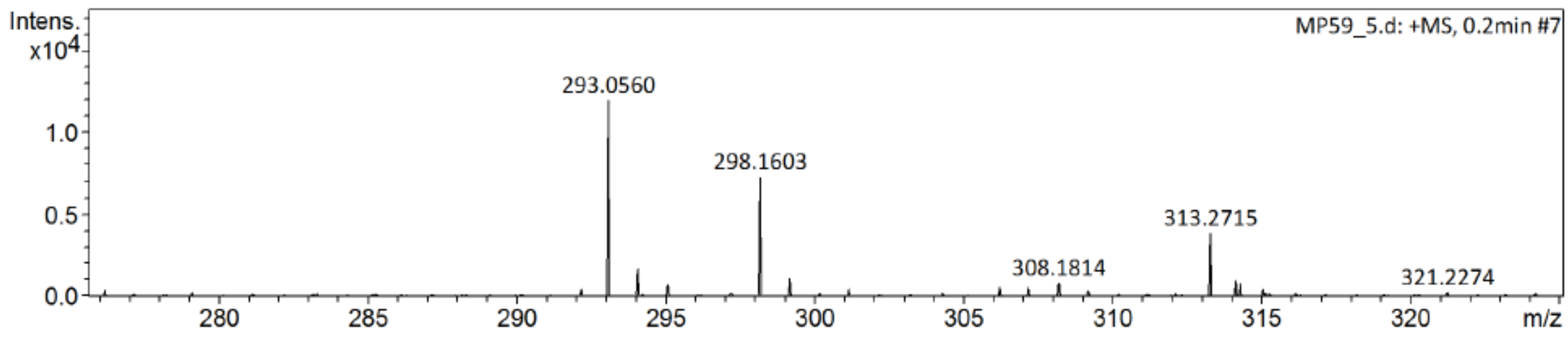

ES-MS of final compound **12** (MeOH).

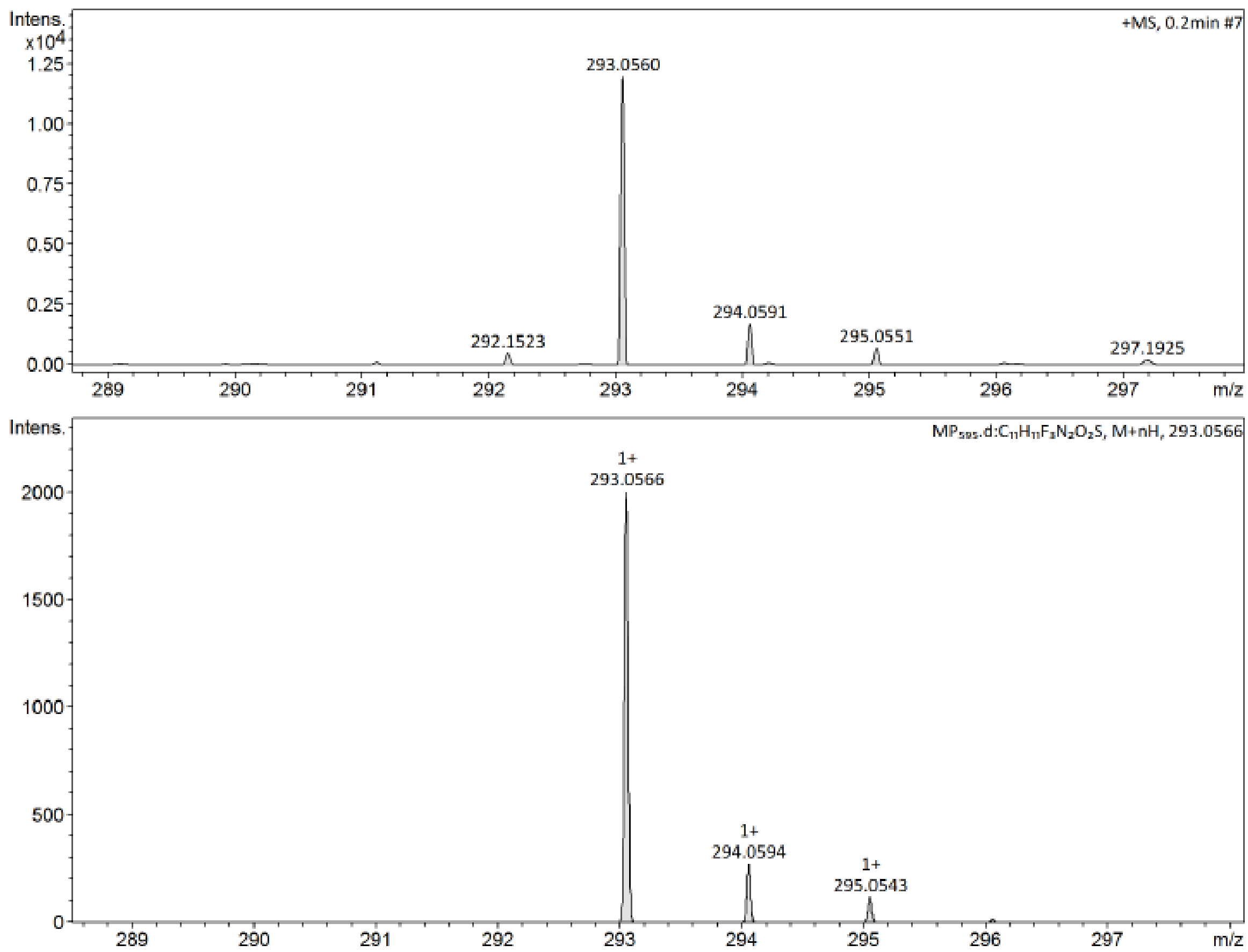

HRMS of final compound **12** (MeOH).

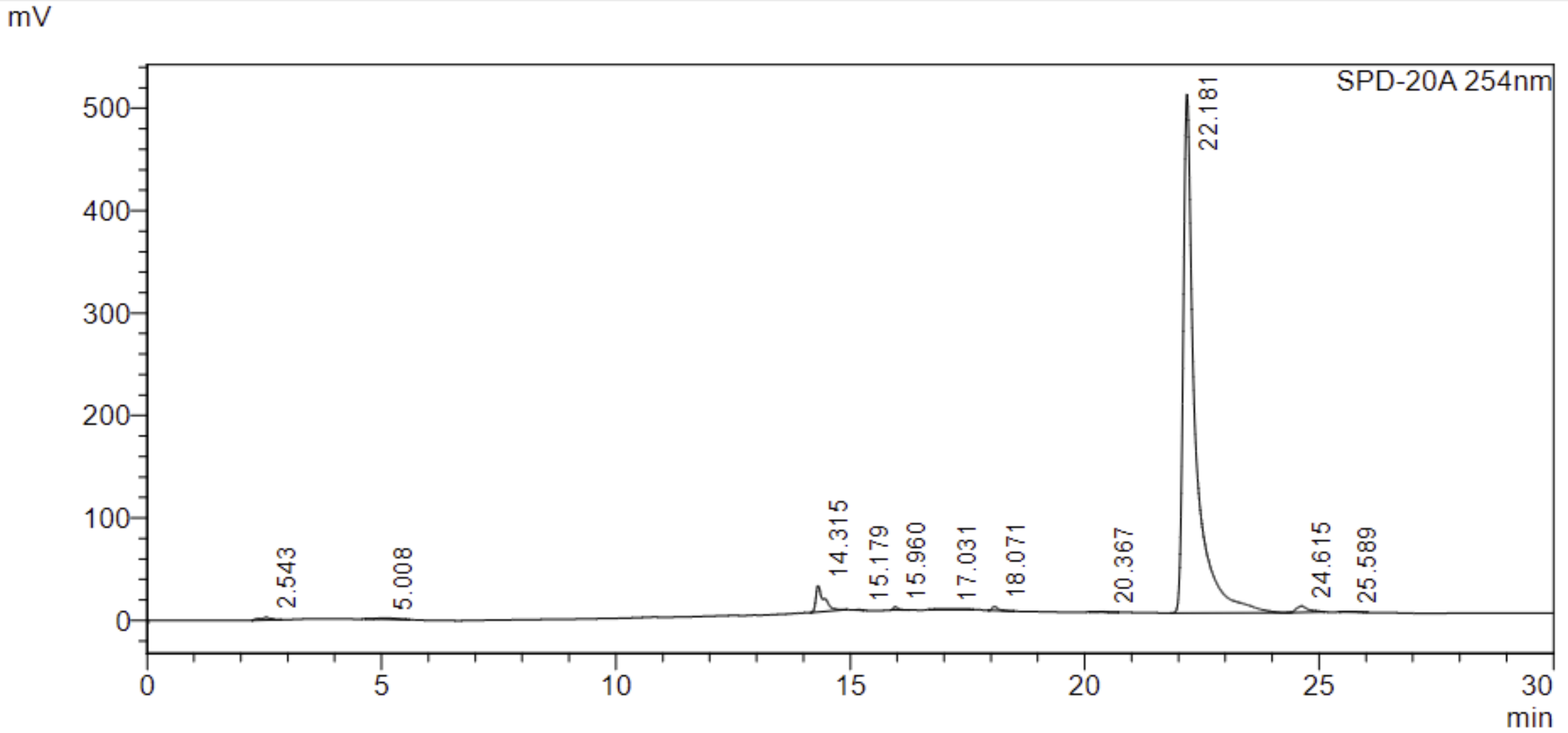

<Peak Table>

SPD-20A 254nm

Peak#	Ret. Time	Area	Height	Conc.	Unit	Mark	Name
1	2.543	43495	2876	0.449		M	
2	5.008	33759	1125	0.349		M	
3	14.315	314363	26091	3.248		M	
4	15.179	2433	432	0.025		M	
5	15.960	22048	3175	0.228		M	
6	17.031	21004	585	0.217		M	
7	18.071	37814	4233	0.391		M	
8	20.367	6348	683	0.066		M	
9	22.181	9077009	506430	93.791		M	
10	24.615	105987	6166	1.095		M	
11	25.589	13654	484	0.141		M	
Total		9677915	552280				

HPLC of final compound 1**2** (flow: 1 ml/min, eleunt gradient: from water-methanol 55:45 to 15:85 in 30 min; detector: 254 nm).

O-pentyl-N-(6-(trifluoromethoxy)benzo[d]thiazol-2-yl)hydroxylamine (**13**)

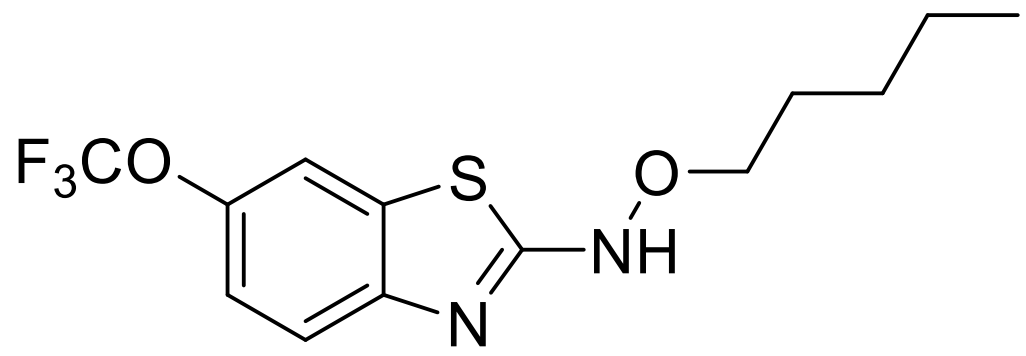

Chemical Formula: $C_{13}H_{15}F_3N_2O_2S$
Exact Mass: 320.08
Molecular Weight: 320.33

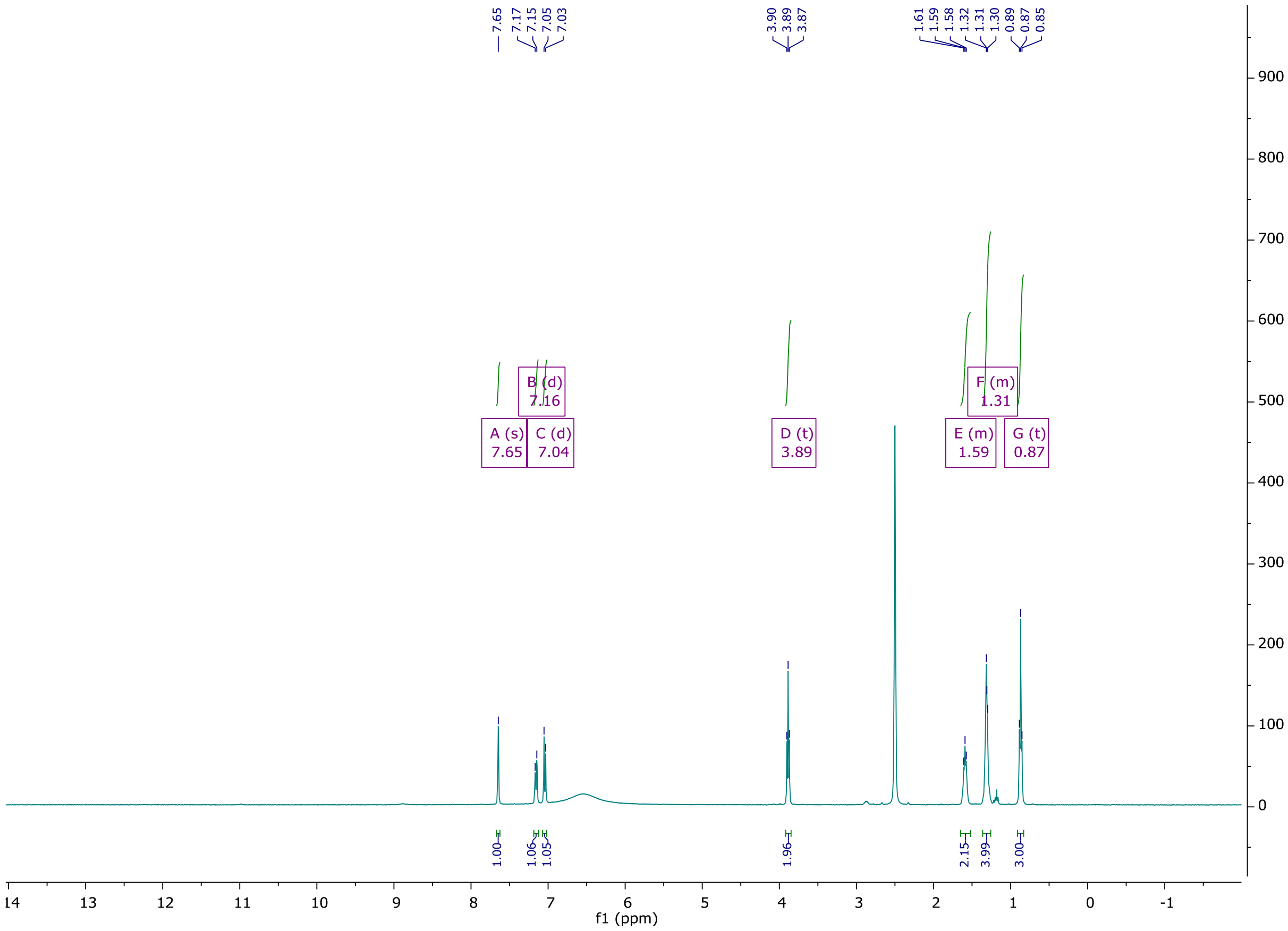

^{1}H-NMR of final compound **13** (DMSO-d_6).

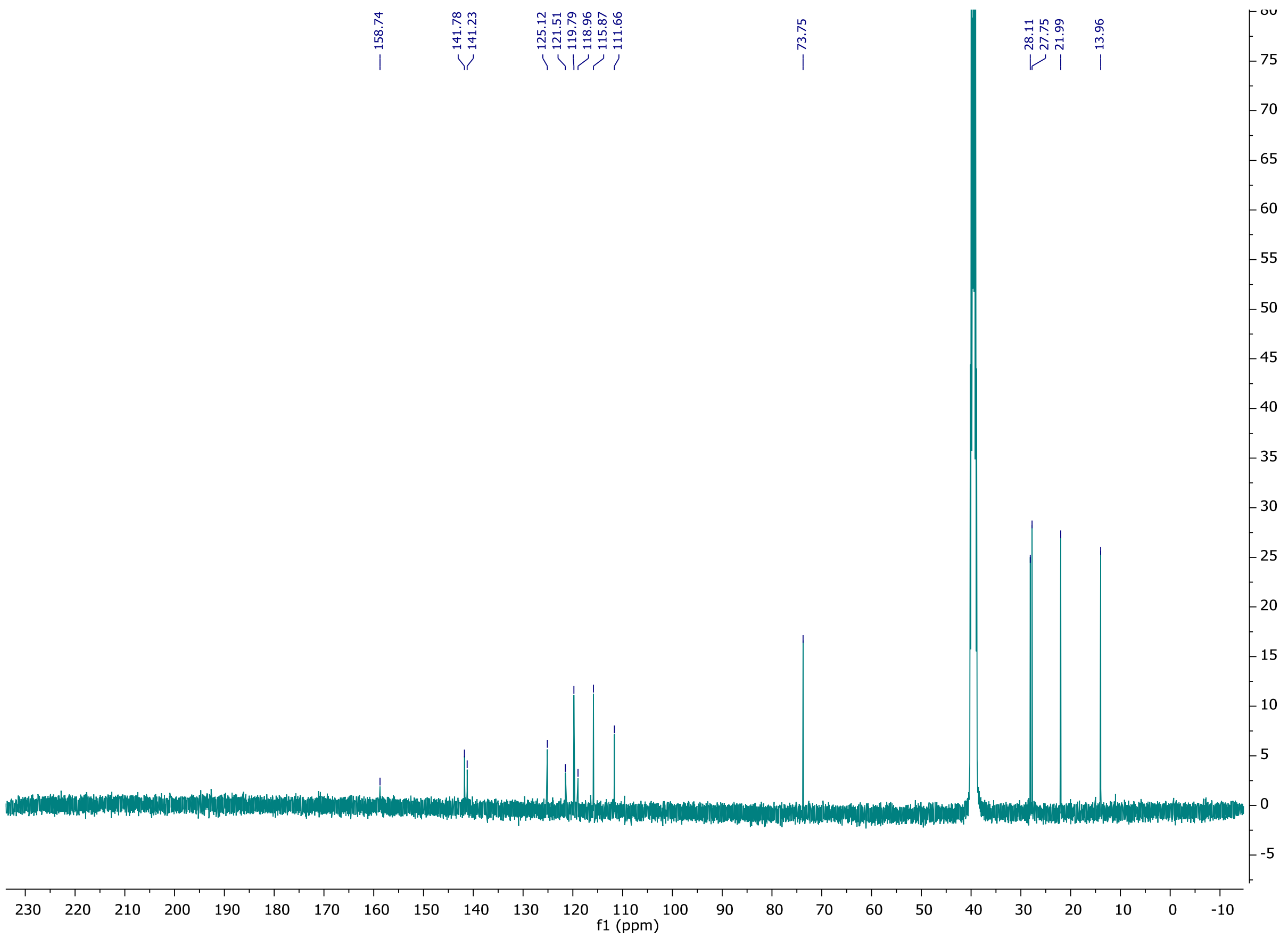

^{13}C-NMR of final compound **13** (DMSO-d_6).

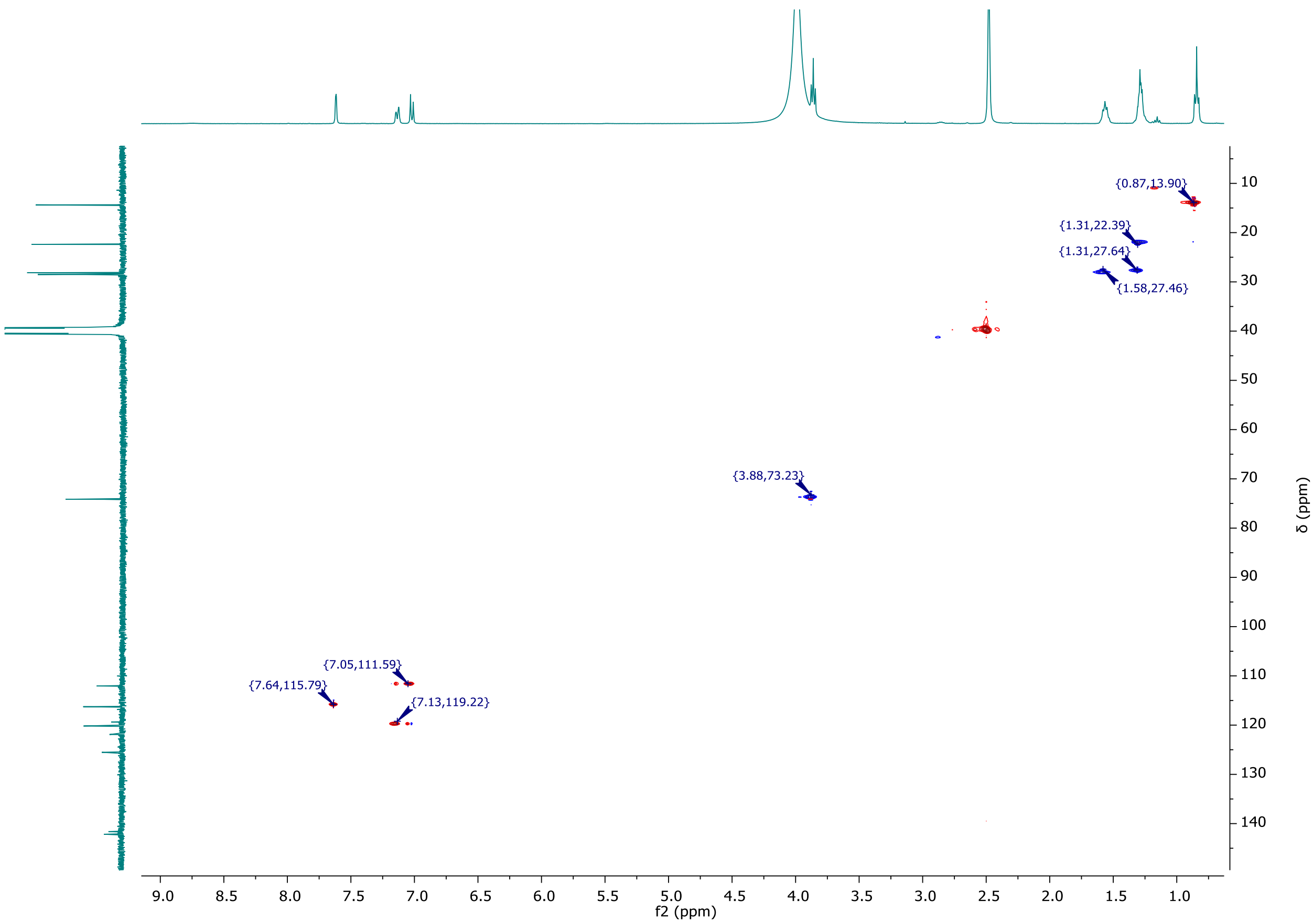

gHSQCAD of final compound **13** (DMSO-d_6).

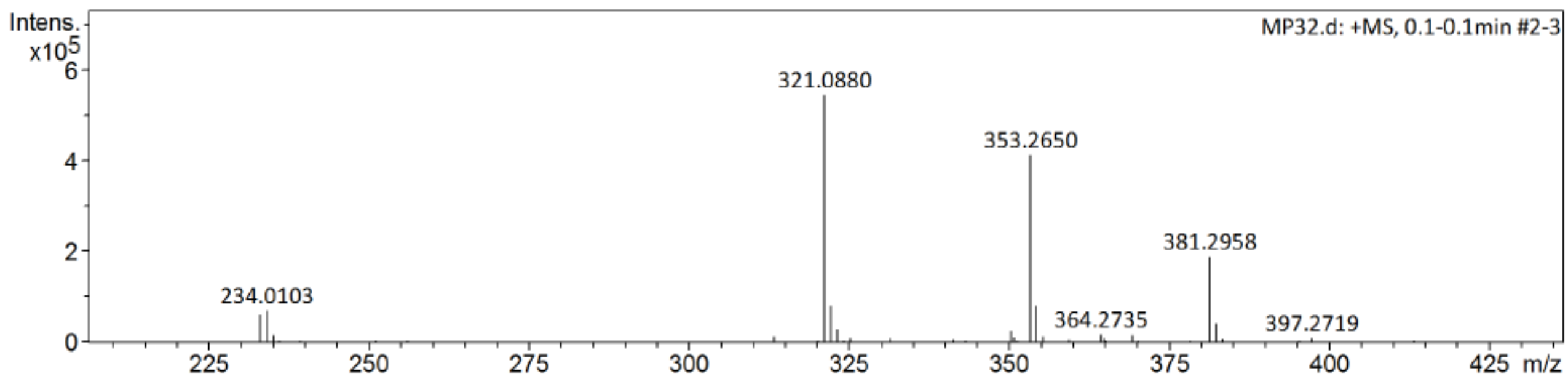

ES-MS of final compound **13** (MeOH).

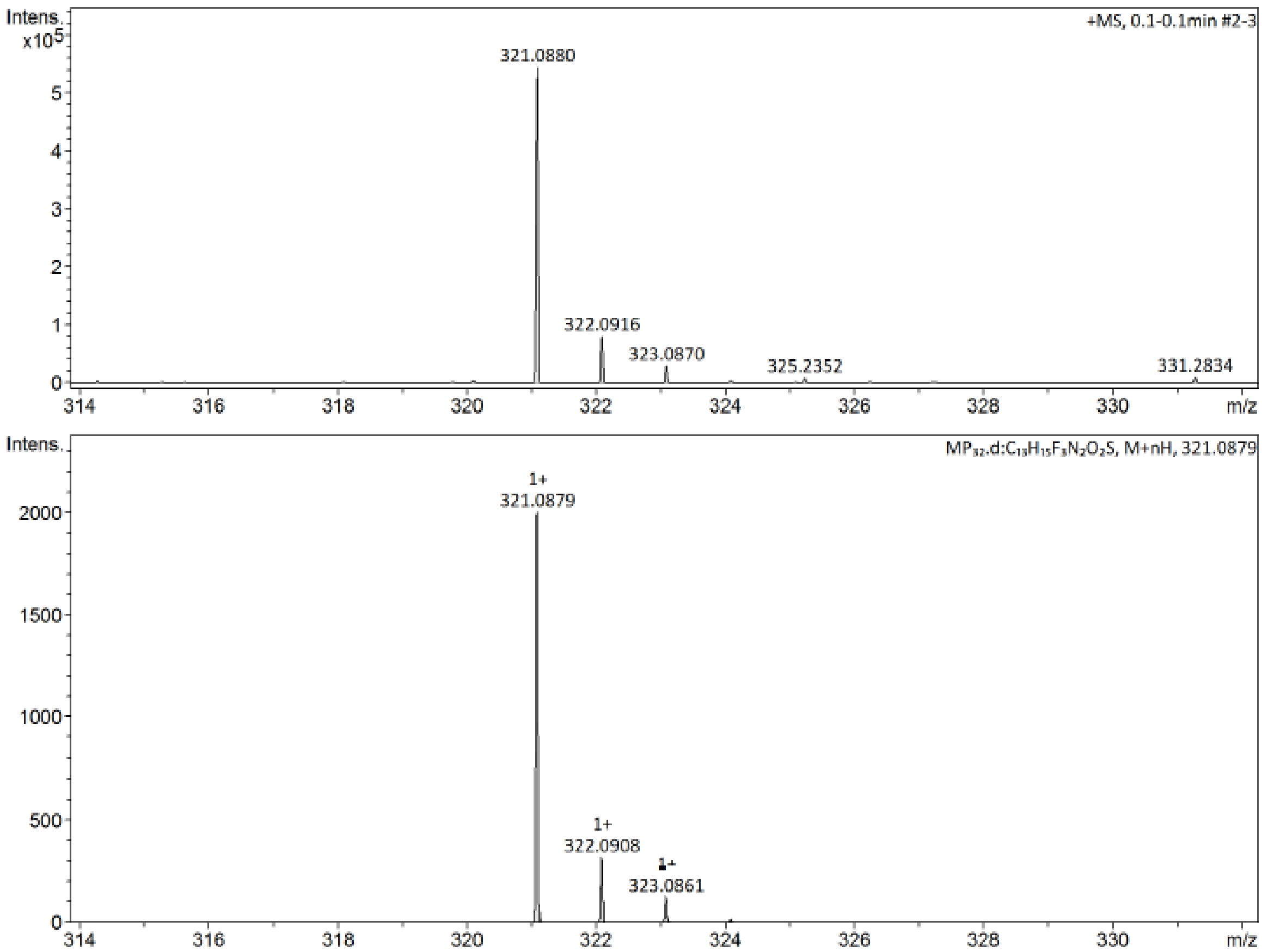

HRMS of final compound **13** (MeOH).

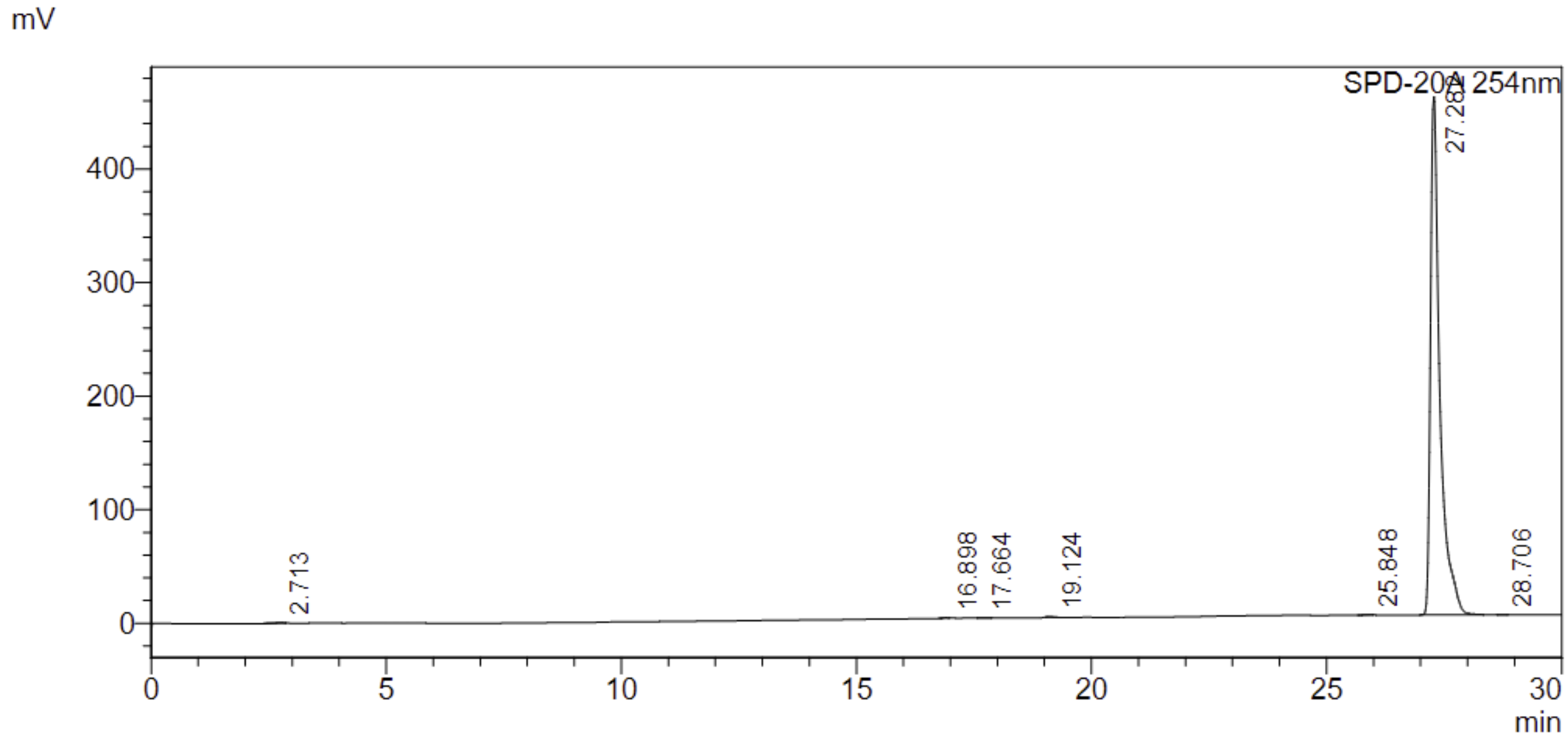

<Peak Table>

SPD-20A 254nm

Peak#	Ret. Time	Area	Height	Conc.	Unit	Mark	Name
1	2.713	10236	651	0.165		M	
2	16.898	2981	401	0.048		M	
3	17.664	582	64	0.009		M	
4	19.124	4952	483	0.080		M	
5	25.848	3298	383	0.053		M	
6	27.282	6179431	456308	99.632		M	
7	28.706	772	106	0.012		M	
Total		6202252	458395				

HPLC of final compound **13** (flow: 1 ml/min, eleunt gradient: from water-methanol 55:45 to 15:85 in 30 min; detector: 254 nm).

O-isopentyl-N-(6-(trifluoromethoxy)benzo[d]thiazol-2-yl)hydroxylamine (**14**)

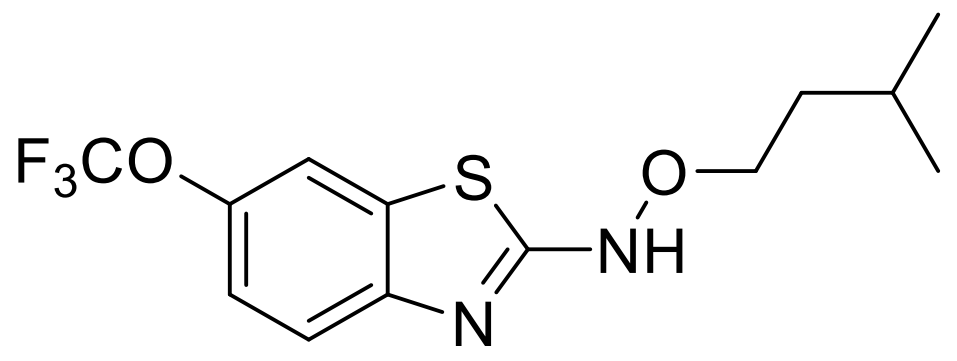

Chemical Formula: $C_{13}H_{15}F_3N_2O_2S$
Exact Mass: 320.08
Molecular Weight: 320.33

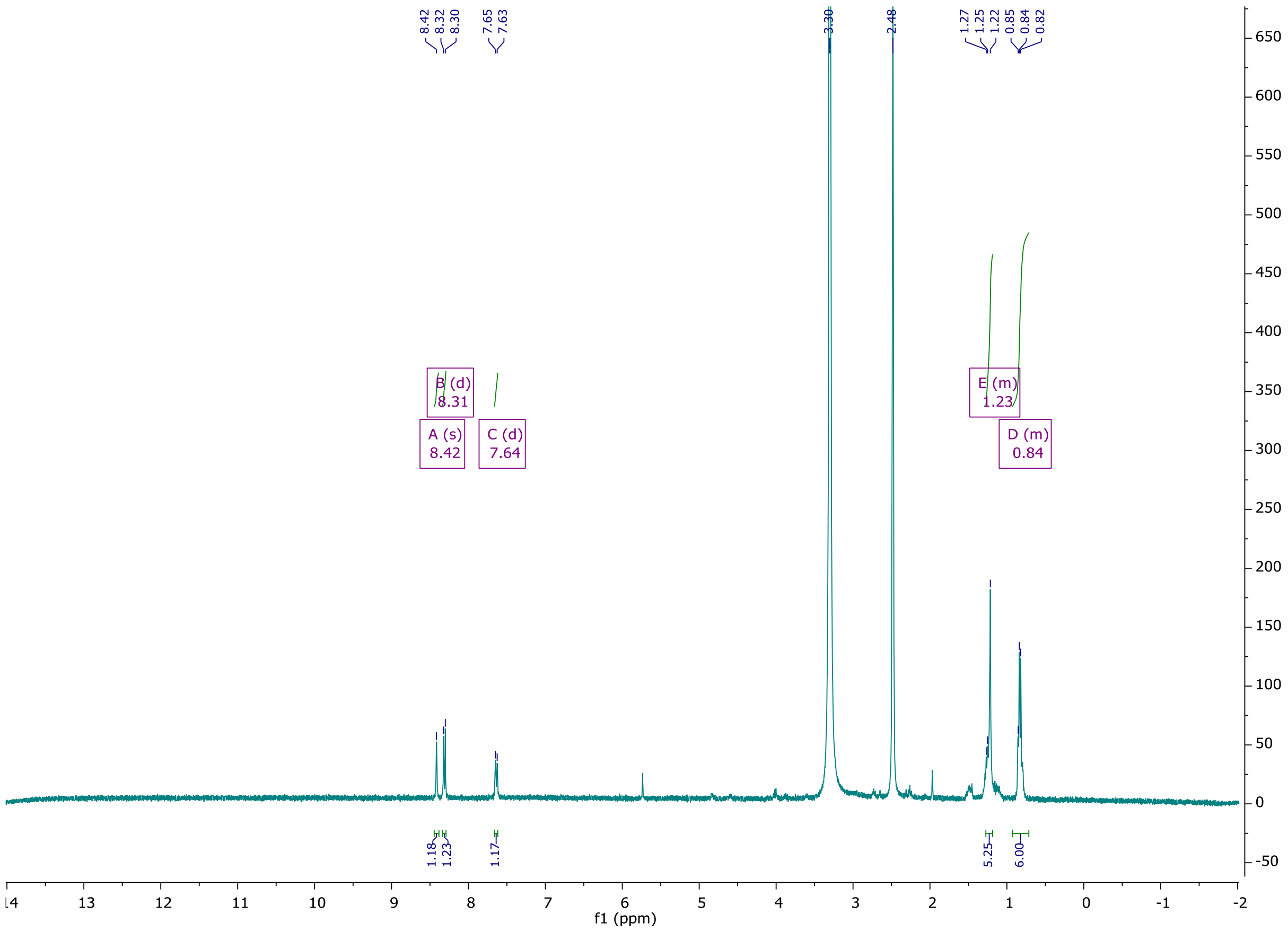

^{1}H-NMR of final compound **14** (DMSO-d_6).

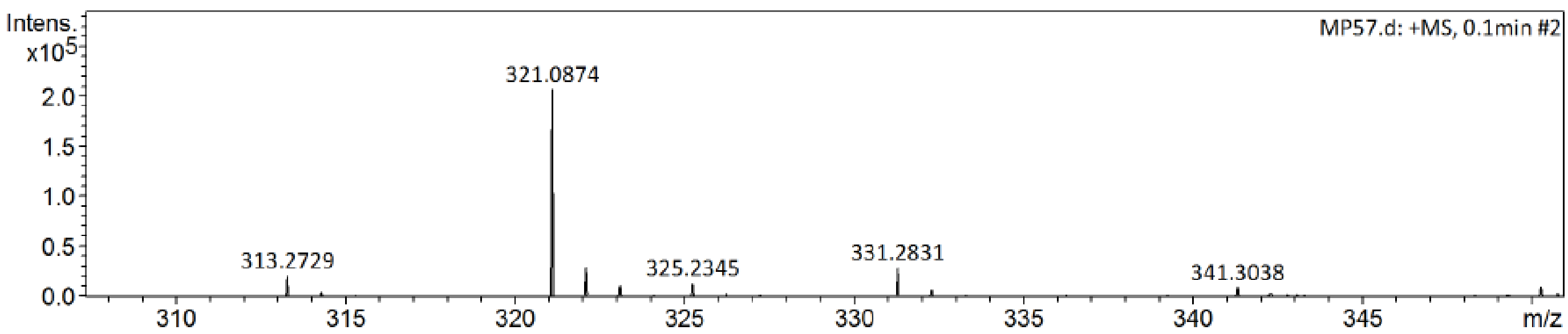

ES-MS of final compound **14** (MeOH).

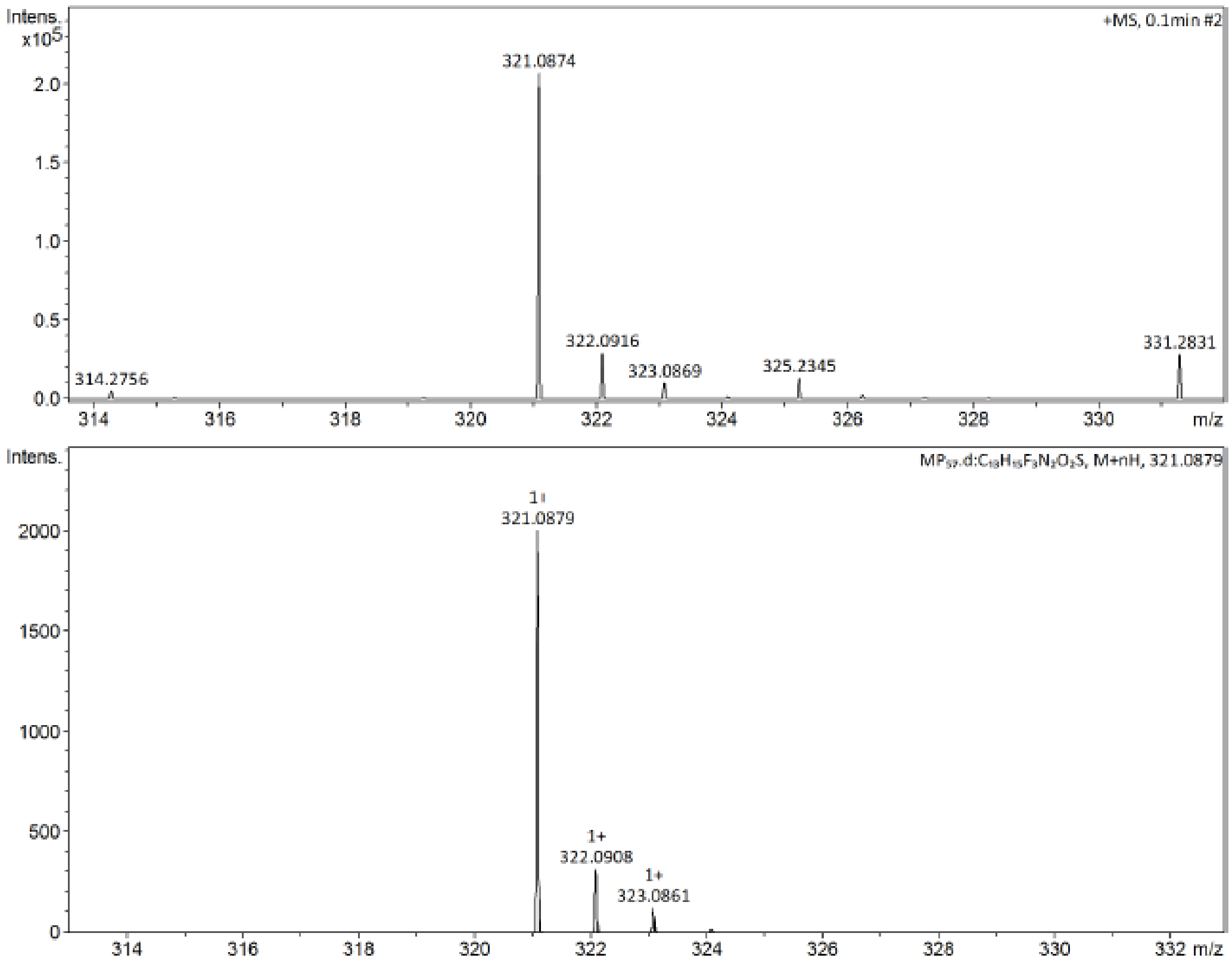

HRMS of final compound **14** (MeOH).

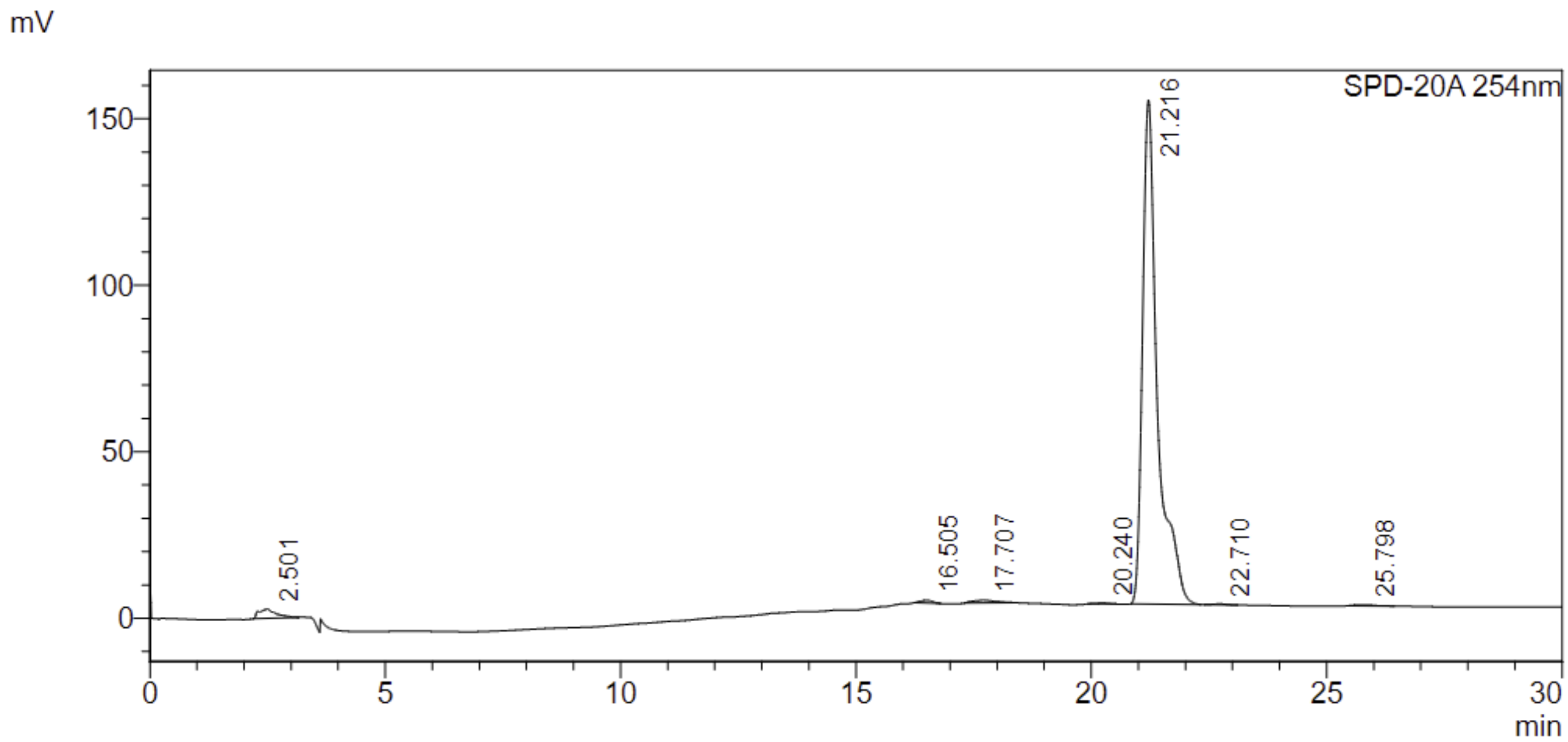

<Peak Table>

SPD-20A 254nm

Peak#	Ret. Time	Area	Height	Conc.	Unit	Mark	Name
1	2.501	70525	2783	2.022		M	
2	16.505	16039	912	0.460		M	
3	17.707	29773	859	0.854		M	
4	20.240	4672	222	0.134		M	
5	21.216	3352786	151325	96.120		M	
6	22.710	5318	283	0.152			
7	25.798	9000	285	0.258		M	
Total		3488114	156670				

HPLC of final compound **14** (flow: 1 ml/min, eleunt gradient: from water-methanol 55:45 to 15:85 in 30 min; detector: 254 nm).

O-benzyl-N-(6-(trifluoromethoxy)benzo[d]thiazol-2-yl)hydroxylamine (**15**)

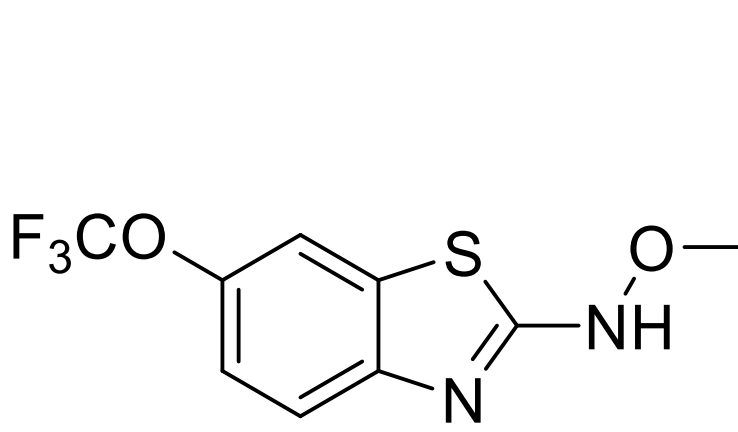

Chemical Formula: $C_{15}H_{11}F_3N_2O_2S$
Exact Mass: 340.05
Molecular Weight: 340.32

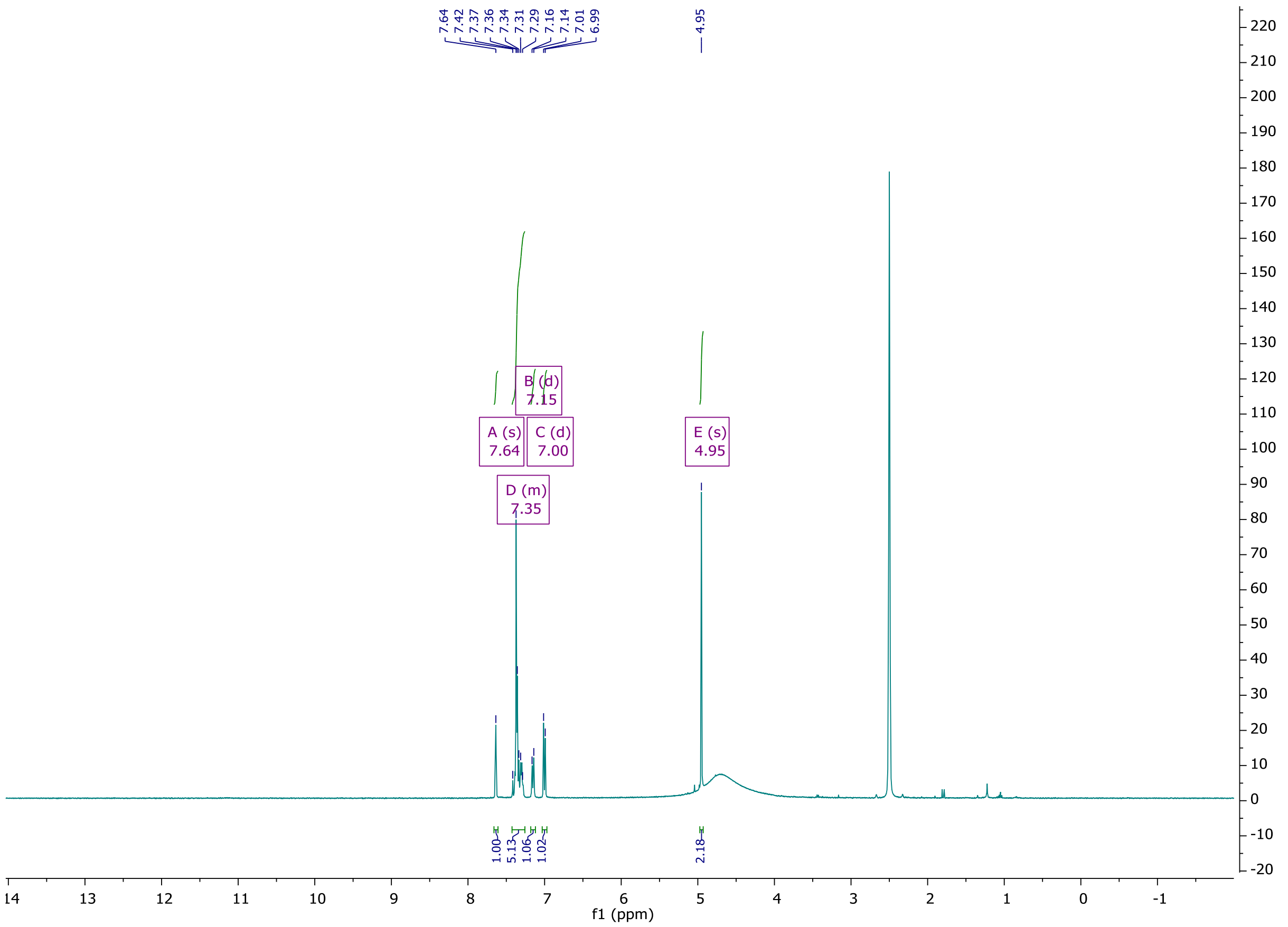

^{1}H-NMR of final compound **15** (DMSO-d_6).

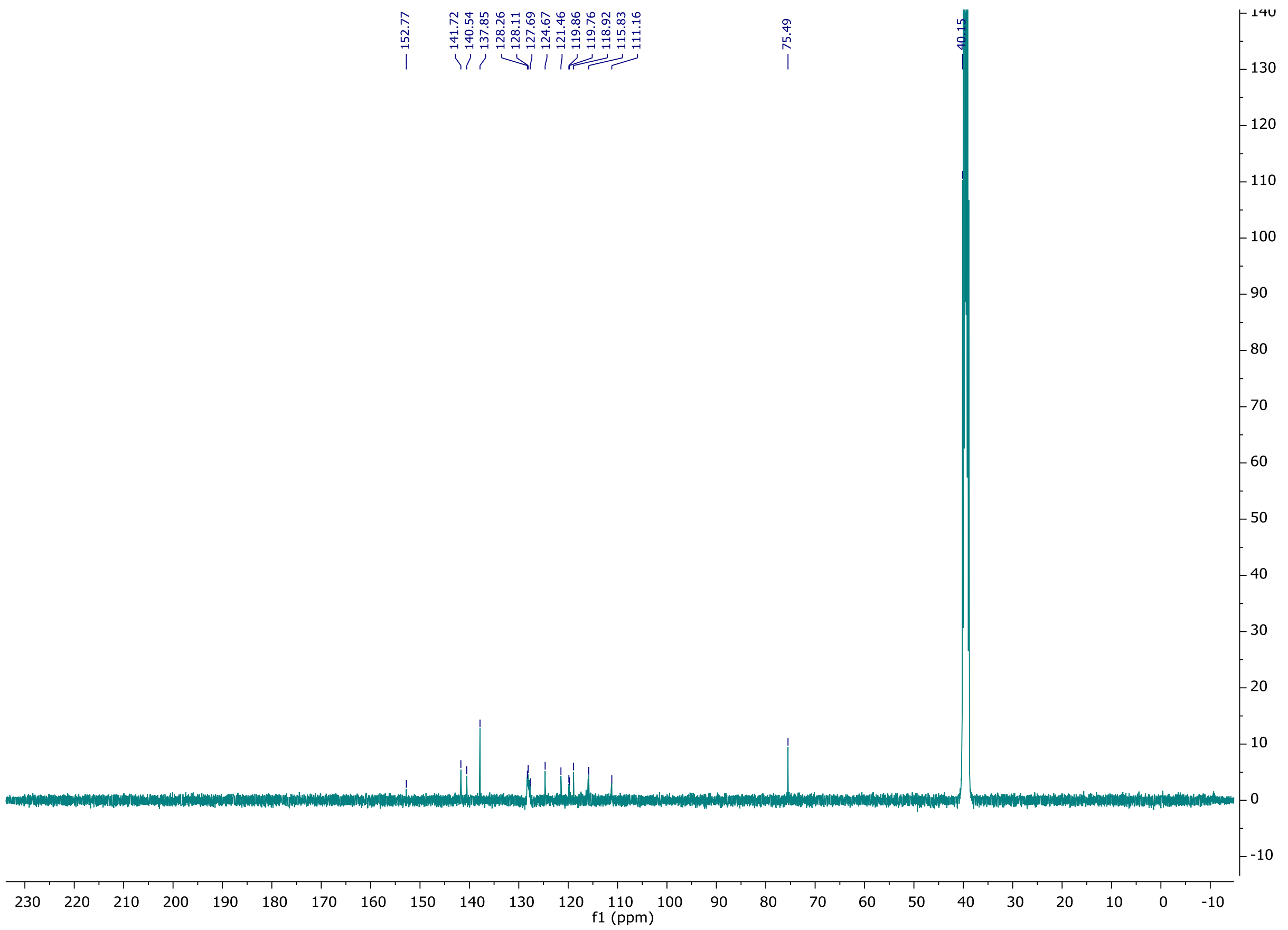

^{13}C-NMR of final compound **15** (DMSO-*d*$_6$).

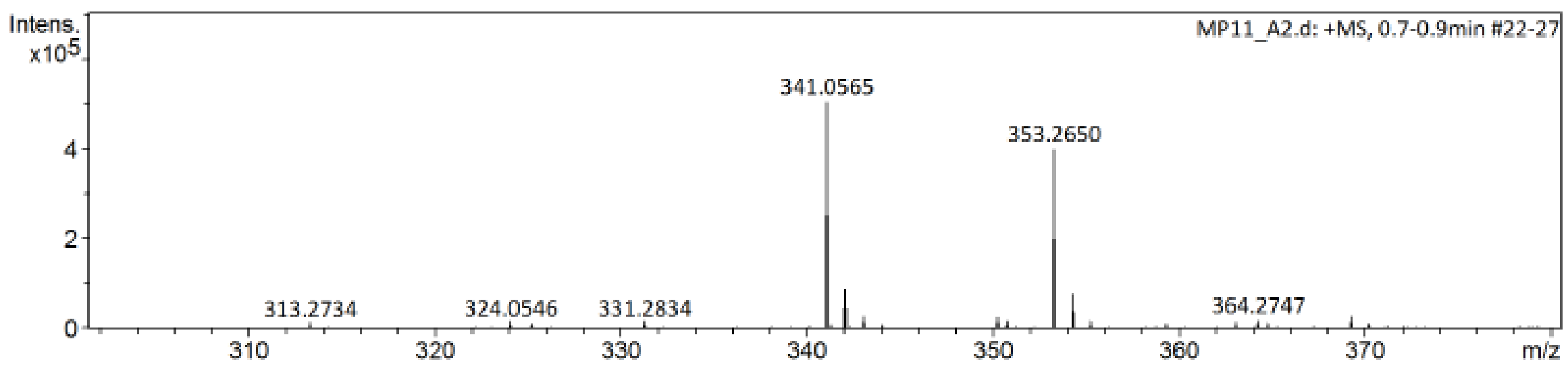

ES-MS of final compound **15** (MeOH).

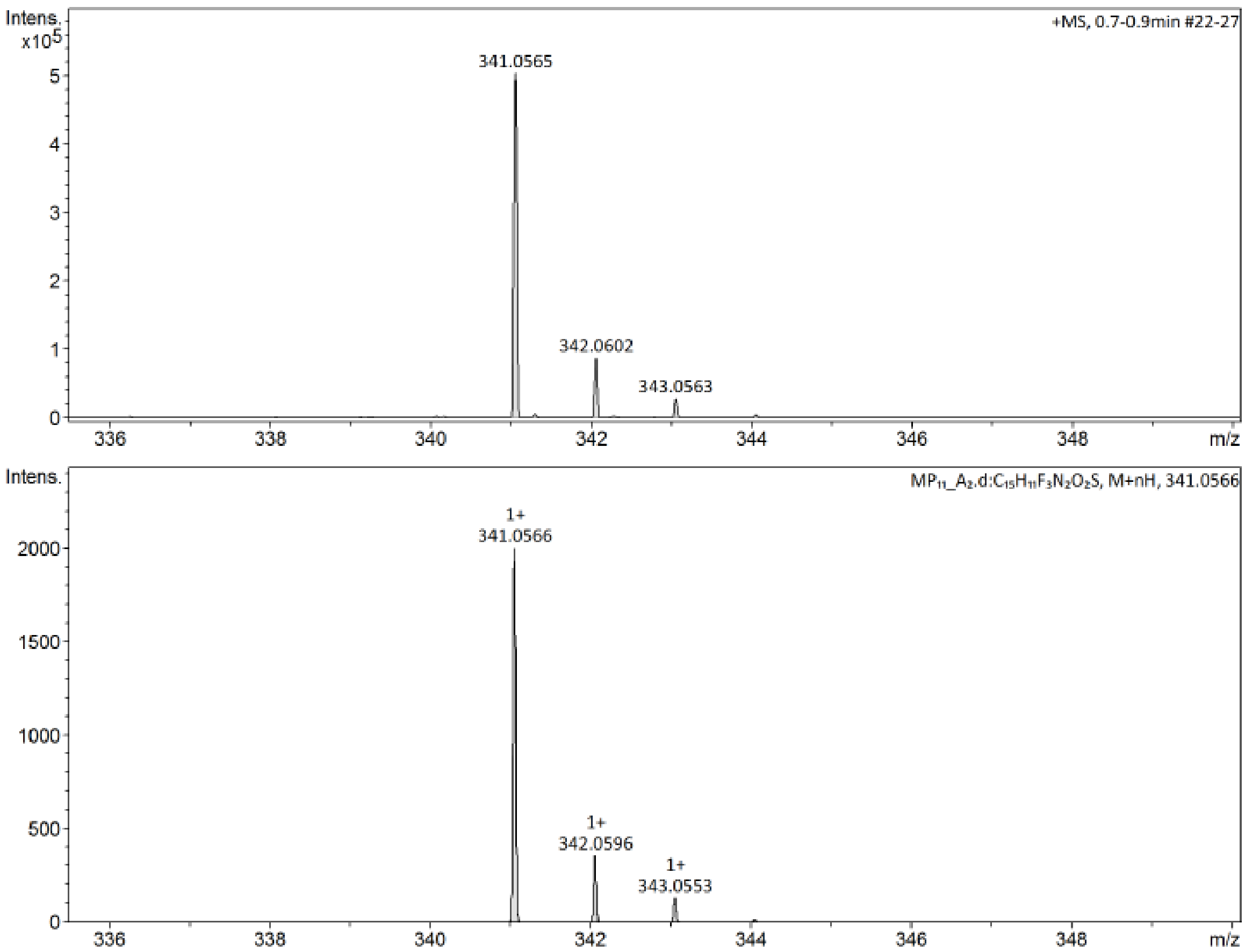

HRMS of final compound **15** (MeOH).

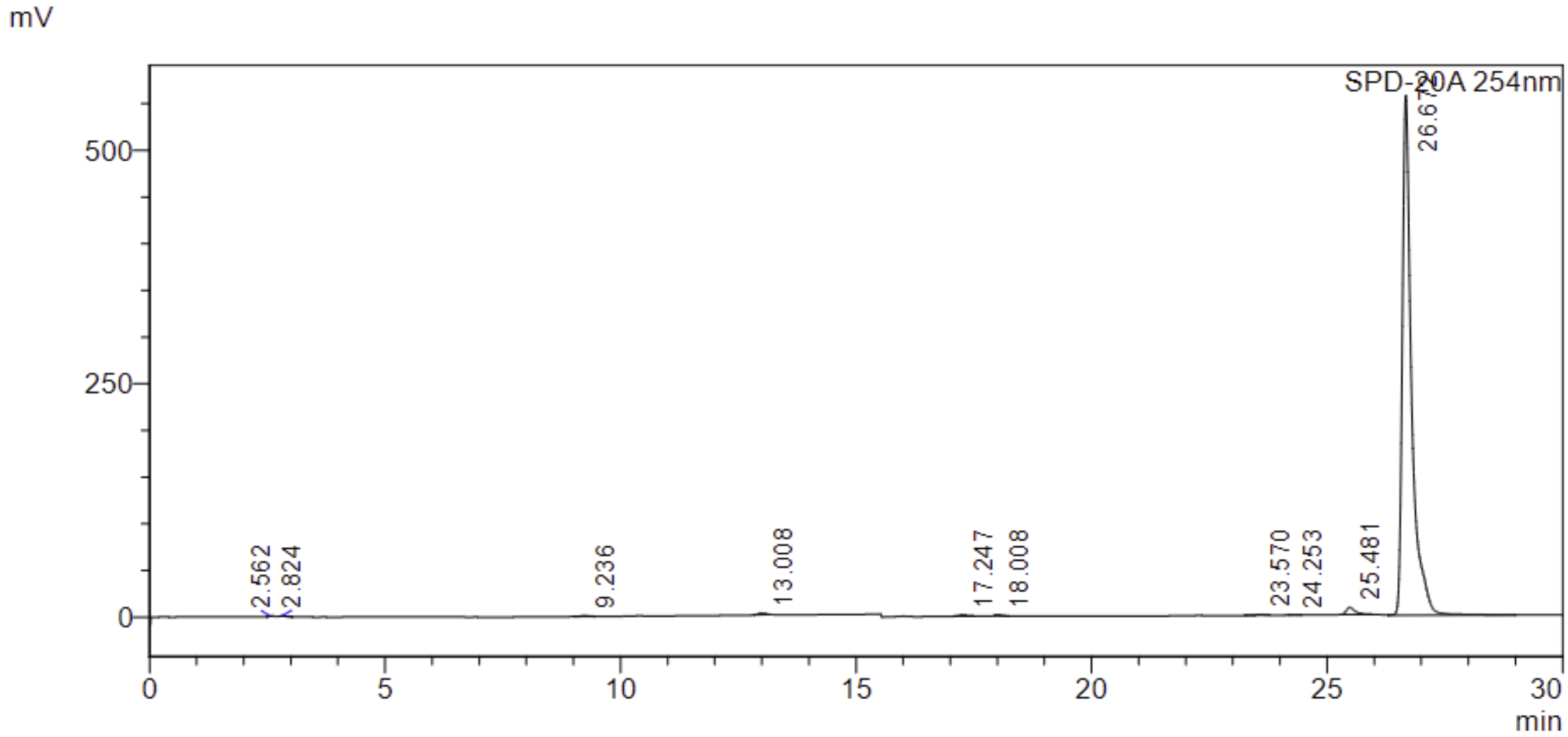

<Peak Table>

SPD-20A 254nm

Peak#	Ret. Time	Area	Height	Conc.	Unit	Mark	Name
1	2.562	2404	467	0.031		M	
2	2.824	4286	576	0.055		M	
3	9.236	9323	755	0.119		M	
4	13.008	12840	1230	0.164		M	
5	17.247	13742	1355	0.175		M	
6	18.008	7395	992	0.094		M	
7	23.570	9566	868	0.122		M	
8	24.253	463	44	0.006		M	
9	25.481	99778	7790	1.274		M	
10	26.672	7674217	557063	97.960		S	
Total		7834014	571140				

HPLC of final compound **15** (flow: 1 ml/min, eleunt gradient: from water-methanol 55:45 to 15:85 in 30 min; detector: 254 nm).

O-(2-ethylhexyl)-N-(6-(trifluoromethoxy)benzo[d]thiazol-2-yl)hydroxylamine (**16**)

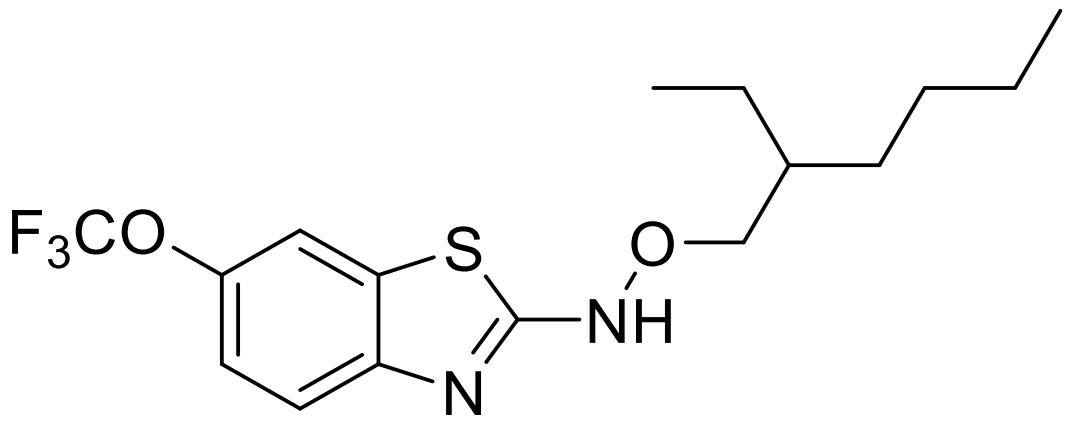

Chemical Formula: $C_{16}H_{21}F_3N_2O_2S$
Exact Mass: 362.13
Molecular Weight: 362.41

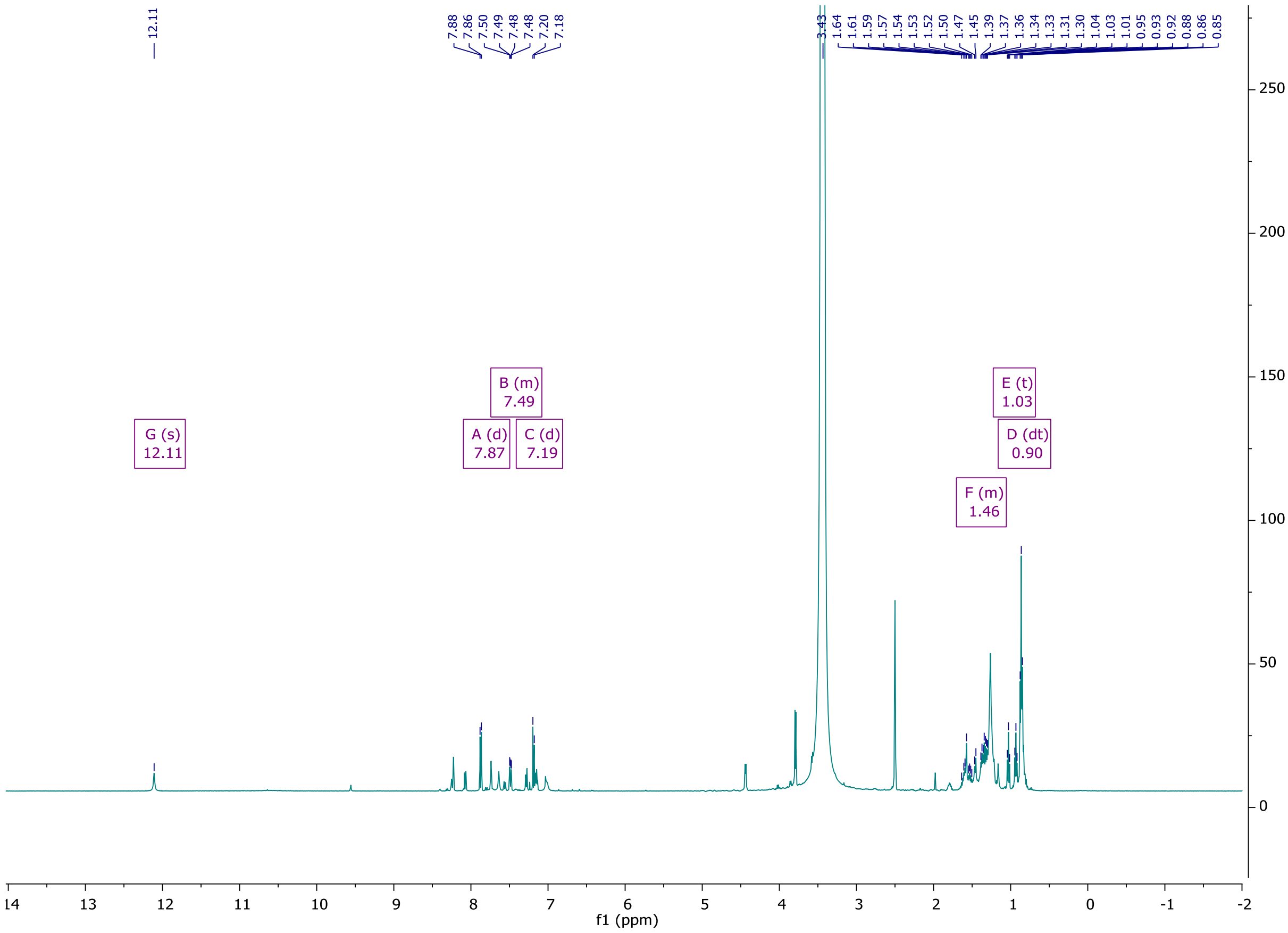

^{1}H-NMR of final compound **16** (DMSO-d_6).

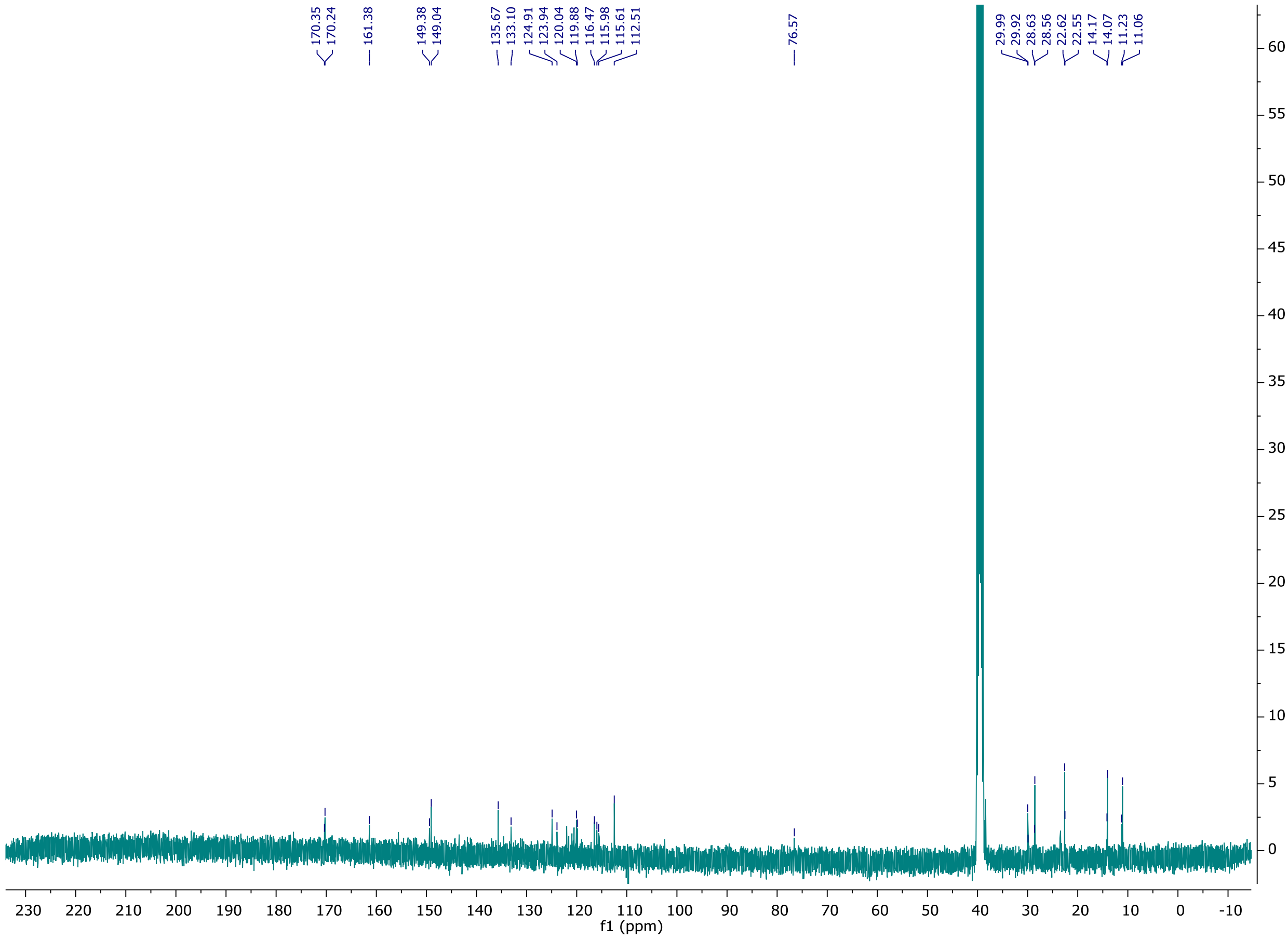

^{13}C-NMR of final compound **16** (DMSO-d_6).

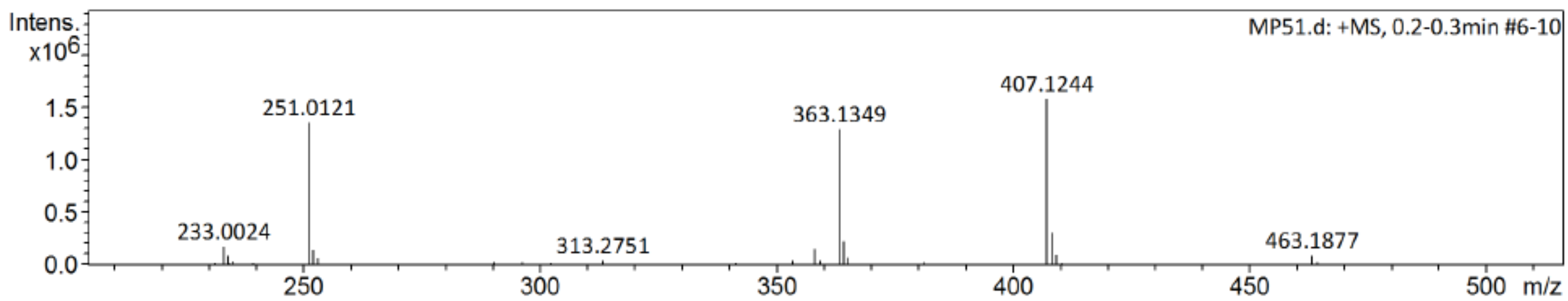

ES-MS of final compound **16** (MeOH).

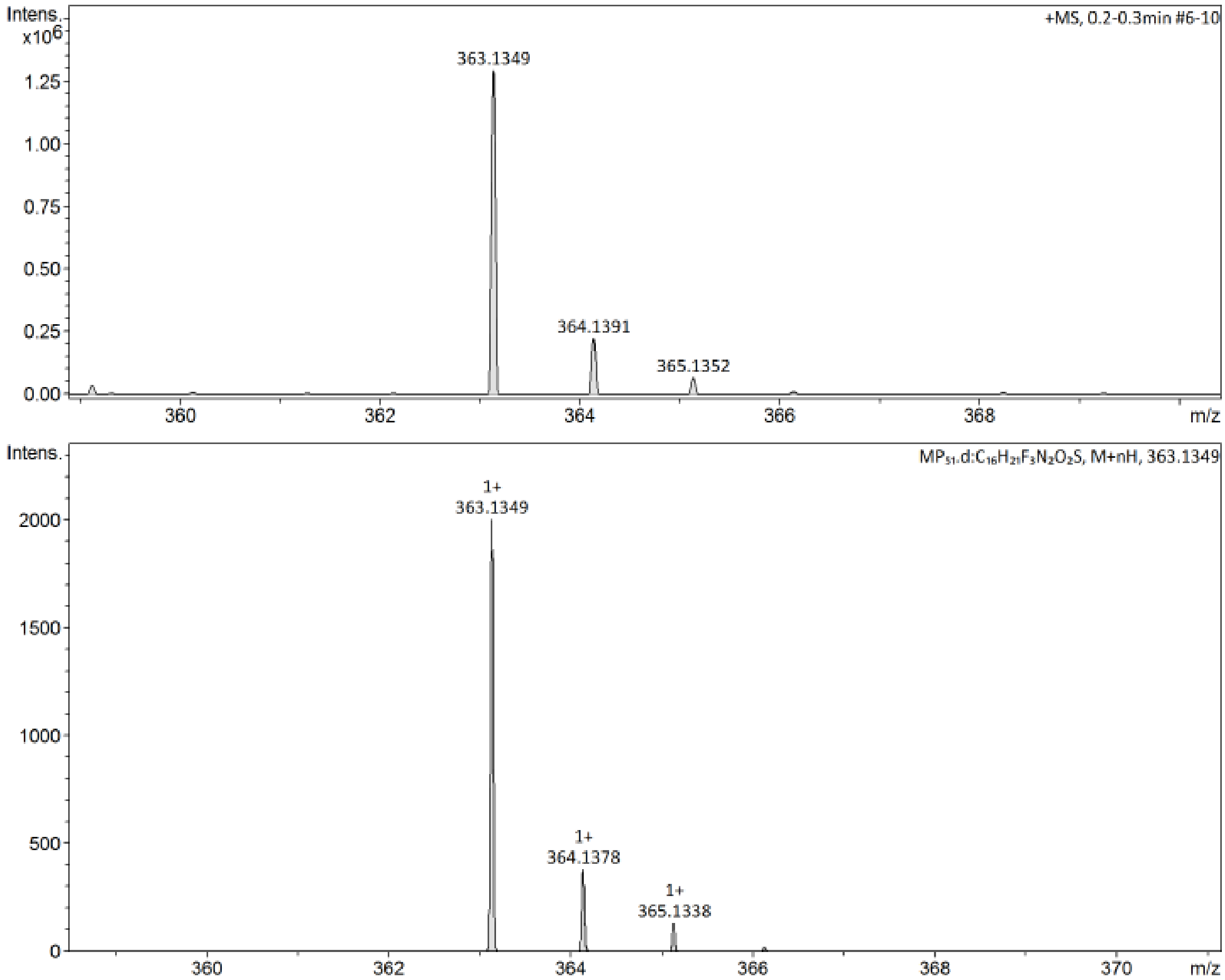

HRMS of final compound **16** (MeOH).

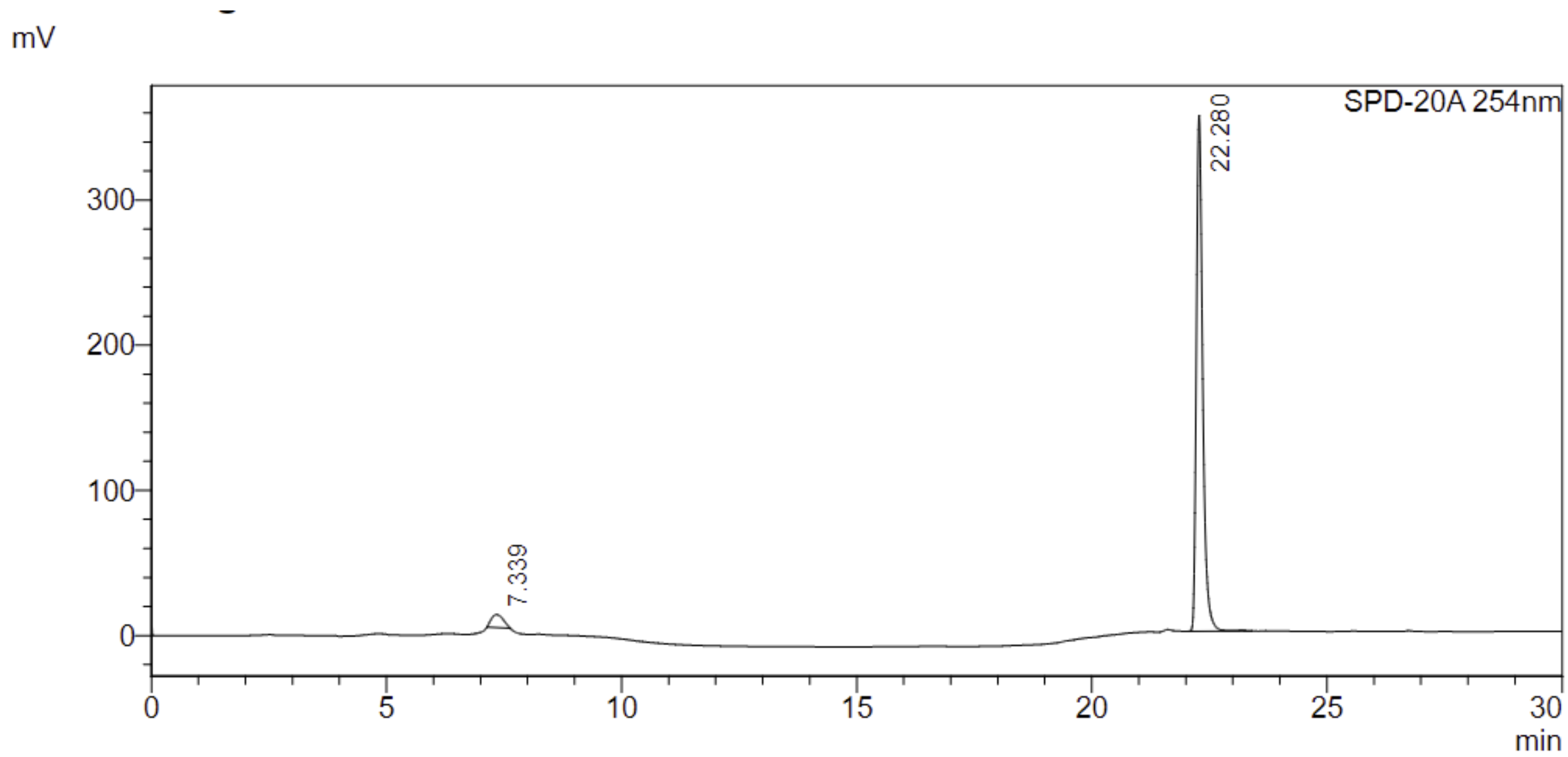

<Peak Table>

SPD-20A 254nm

Peak#	Ret. Time	Area	Height	Conc.	Unit	Mark	Name
1	7.339	162273	9020	4.599			
2	22.280	3365957	355337	95.401			
Total		3528230	364357				

HPLC of final compound **16** (flow: 1 ml/min, eleunt gradient: from water-methanol 55:45 to 15:85 in 30 min; detector: 254 nm).

2-(2-methylhydrazineyl)-6-(trifluoromethoxy)benzo[*d*]thiazole hydrochloride (**17**)

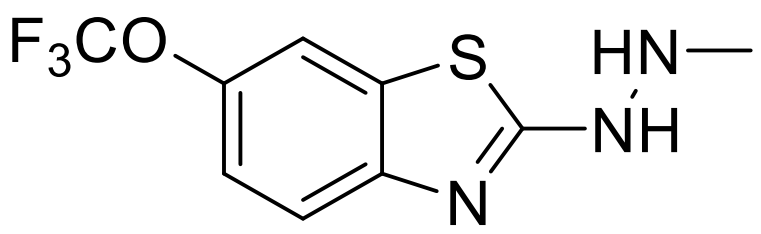

Chemical Formula: $C_9H_8F_3N_3OS$
Exact Mass: 263.03
Molecular Weight: 263.24

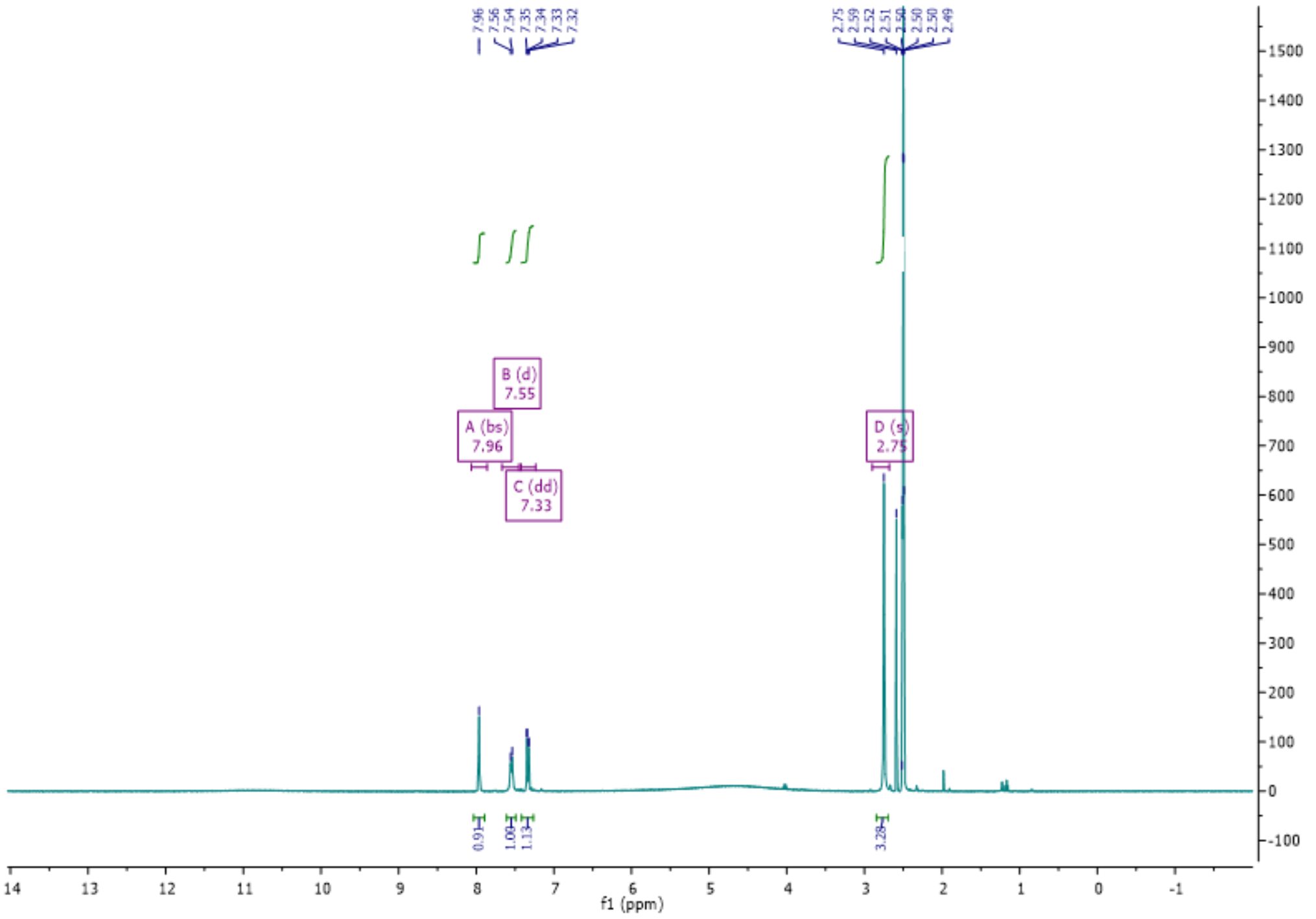

^{1}H-NMR of final compound **17** (DMSO-d_6).

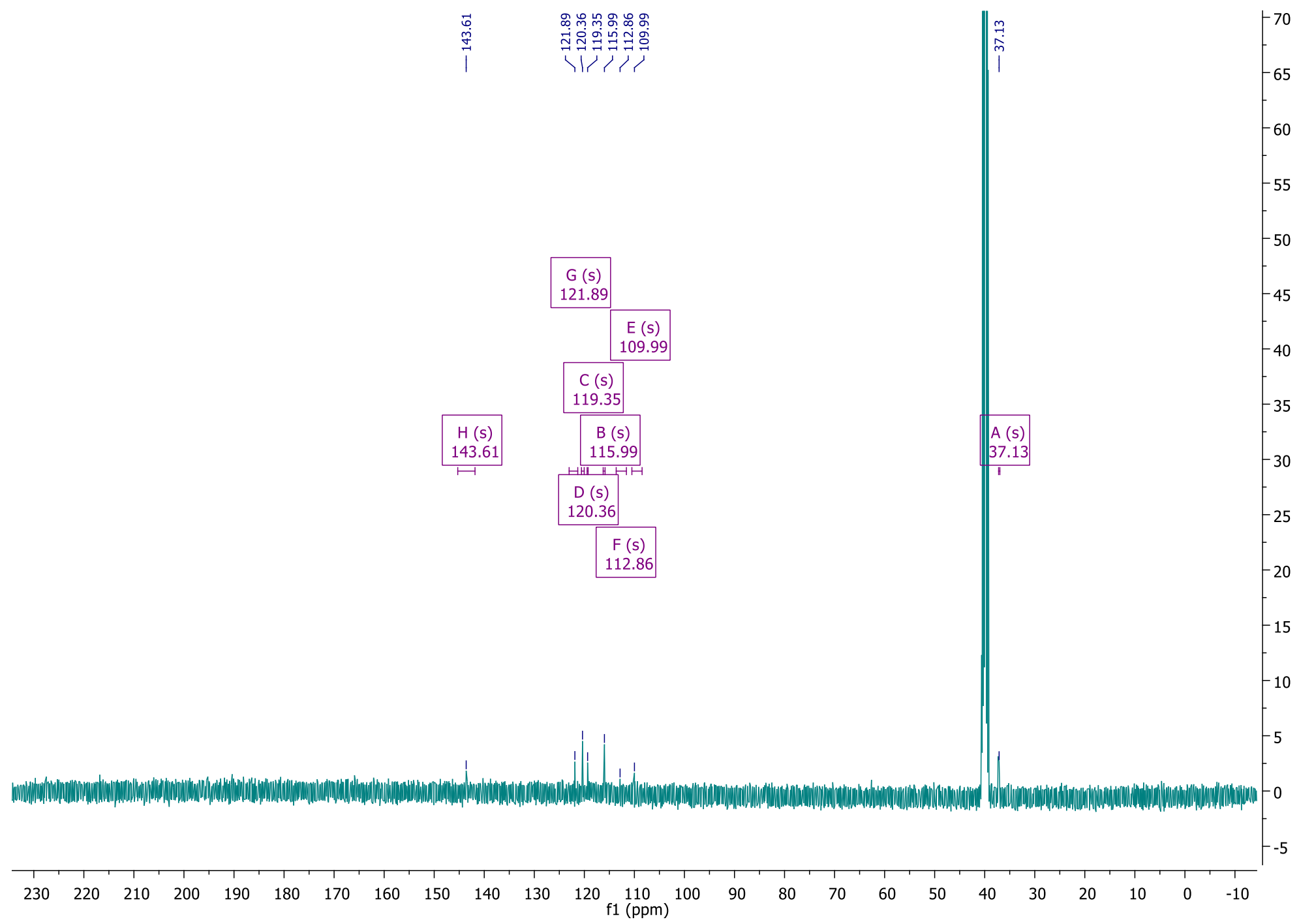

^{13}C-NMR of final compound **17** (DMSO-d_6).

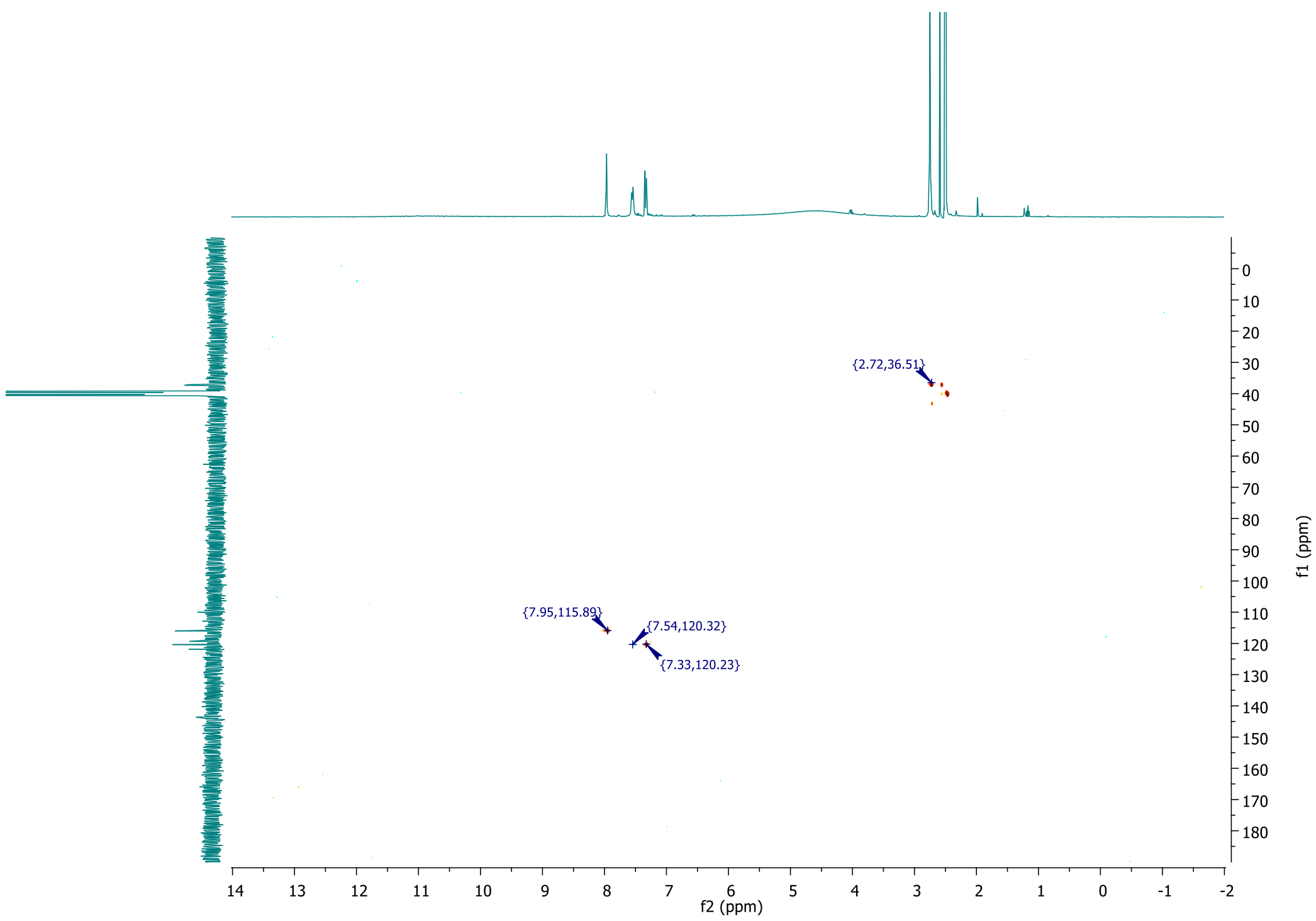

gHSQCAD of final compound **17** (DMSO-d_6).

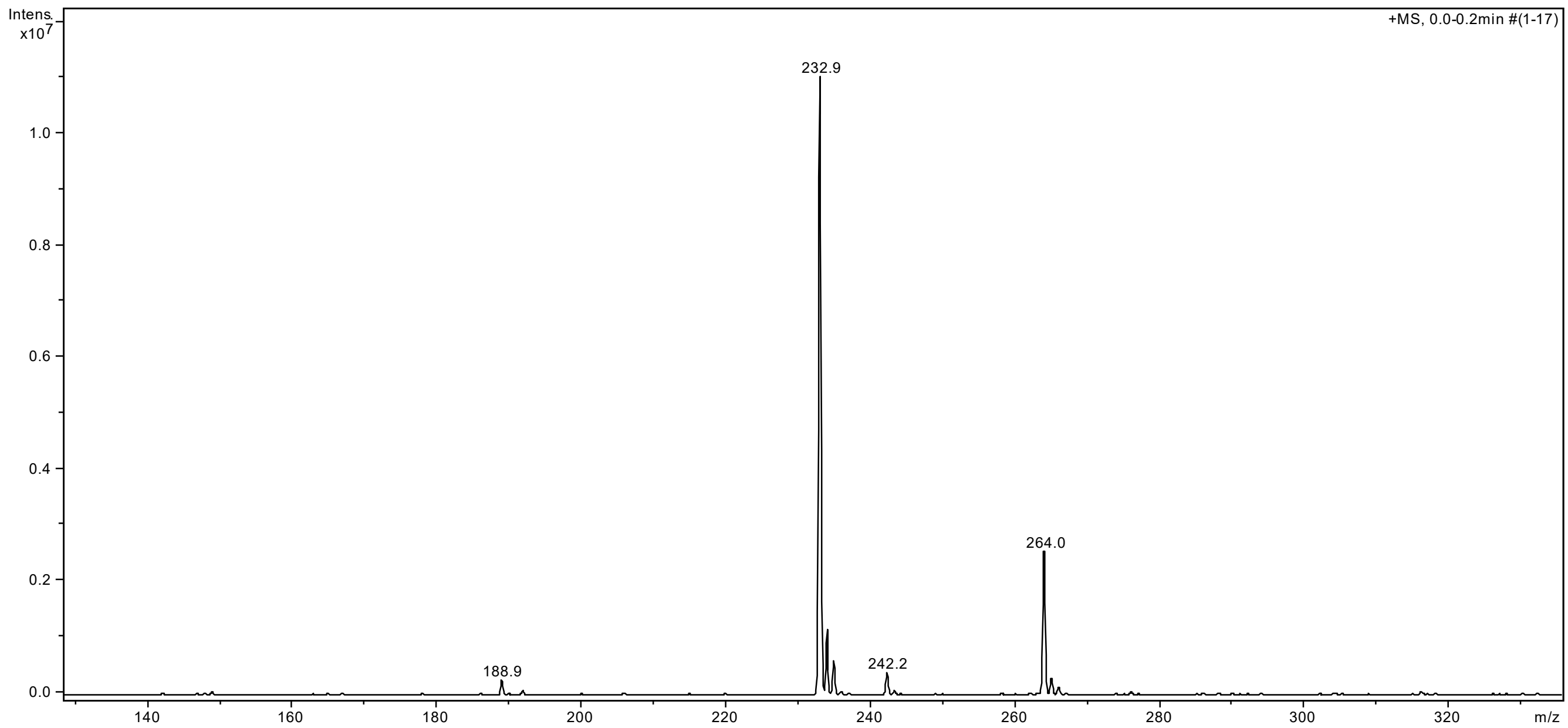

ES-MS of final compound **17** (MeOH).

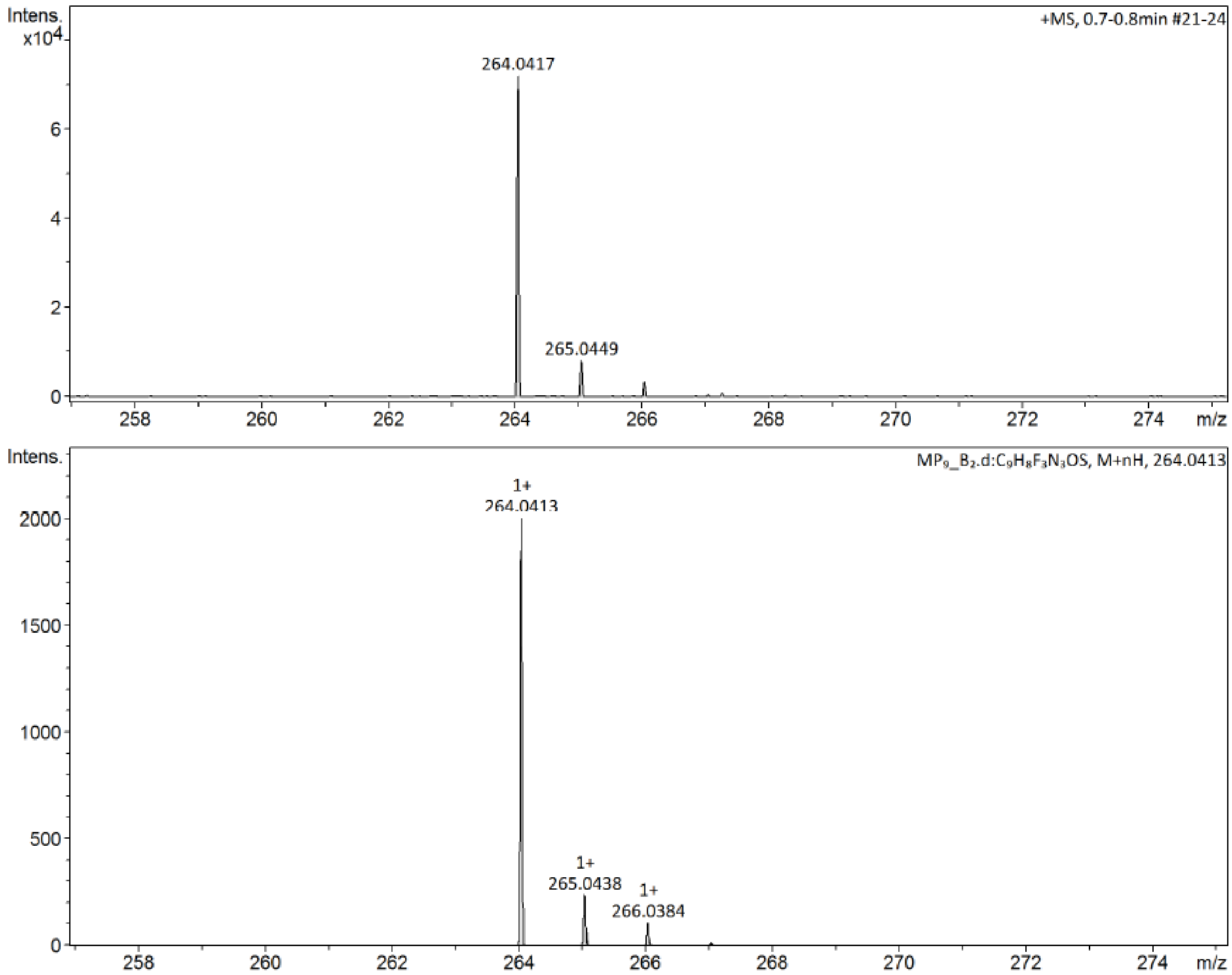

HRMS of final compound **17** (MeOH).

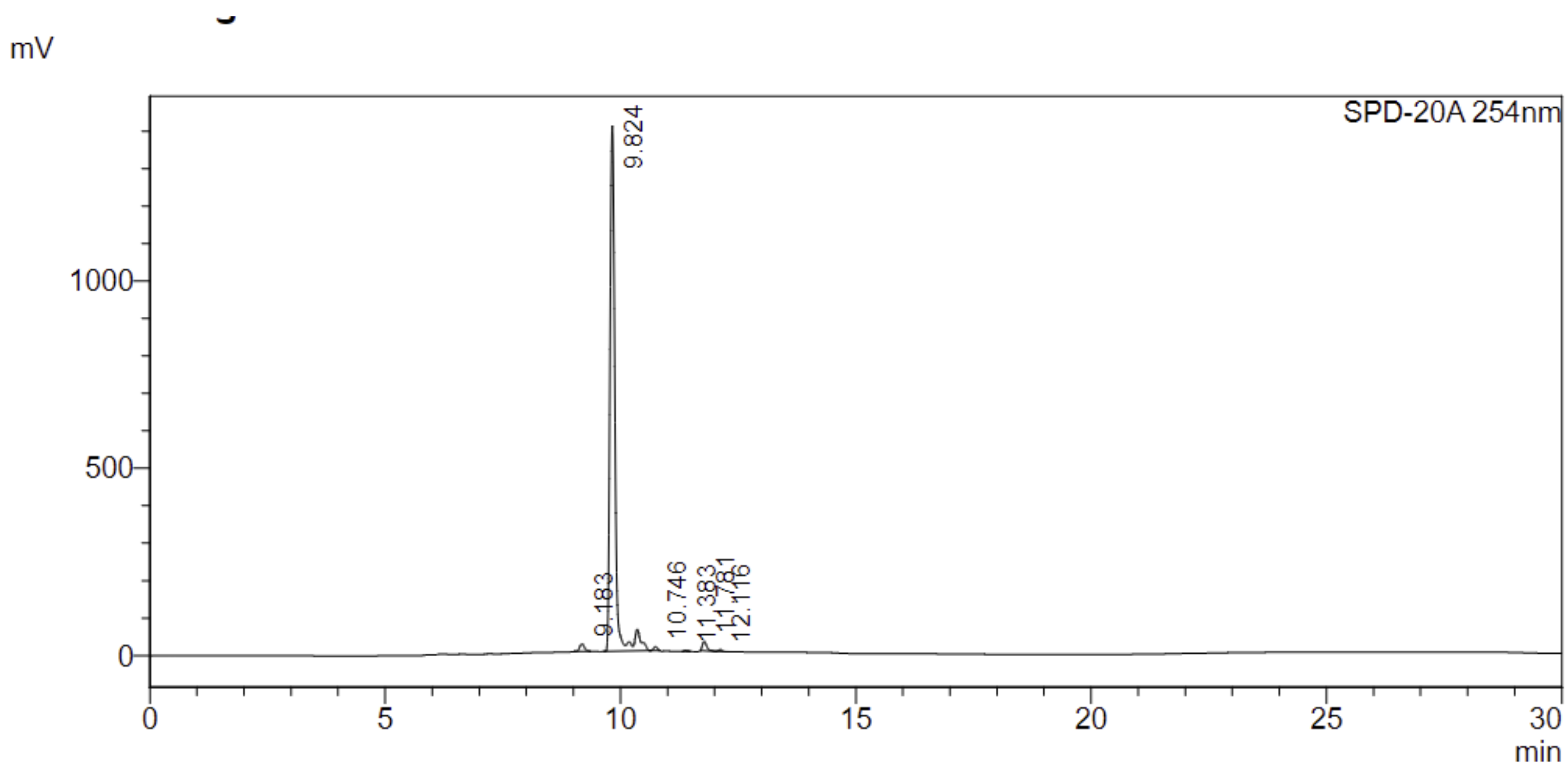

<Peak Table>

SPD-20A 254nm

Peak#	Ret. Time	Area	Height	Conc.	Unit	Mark	Name
1	9.183	140345	20282	1.282		M	
2	9.824	10547784	1402406	96.346		M	
3	10.746	61050	10834	0.558		M	
4	11.383	7630	1604	0.070		M	
5	11.781	162184	24872	1.481		M	
6	12.116	28835	5029	0.263		M	
Total		10947827	1465027				

HPLC of final compound **17** (flow: 1 ml/min, eleunt gradient: from water-methanol 55:45 to 15:85 in 30 min; detector: 254 nm).

2-(2-propylhydrazineyl)-6-(trifluoromethoxy)benzo[*d*]thiazole hydrochloride (**18**)

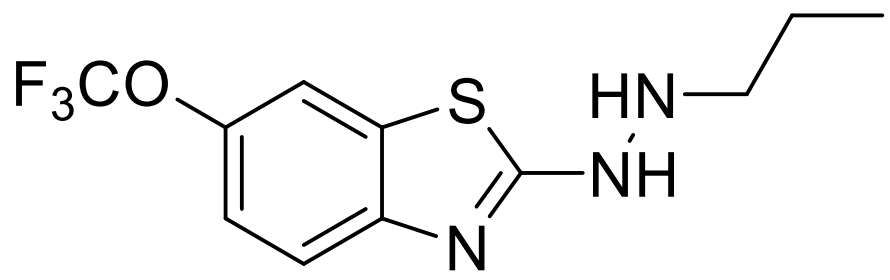

Chemical Formula: $C_{11}H_{12}F_3N_3OS$
Exact Mass: 291.07
Molecular Weight: 291.29

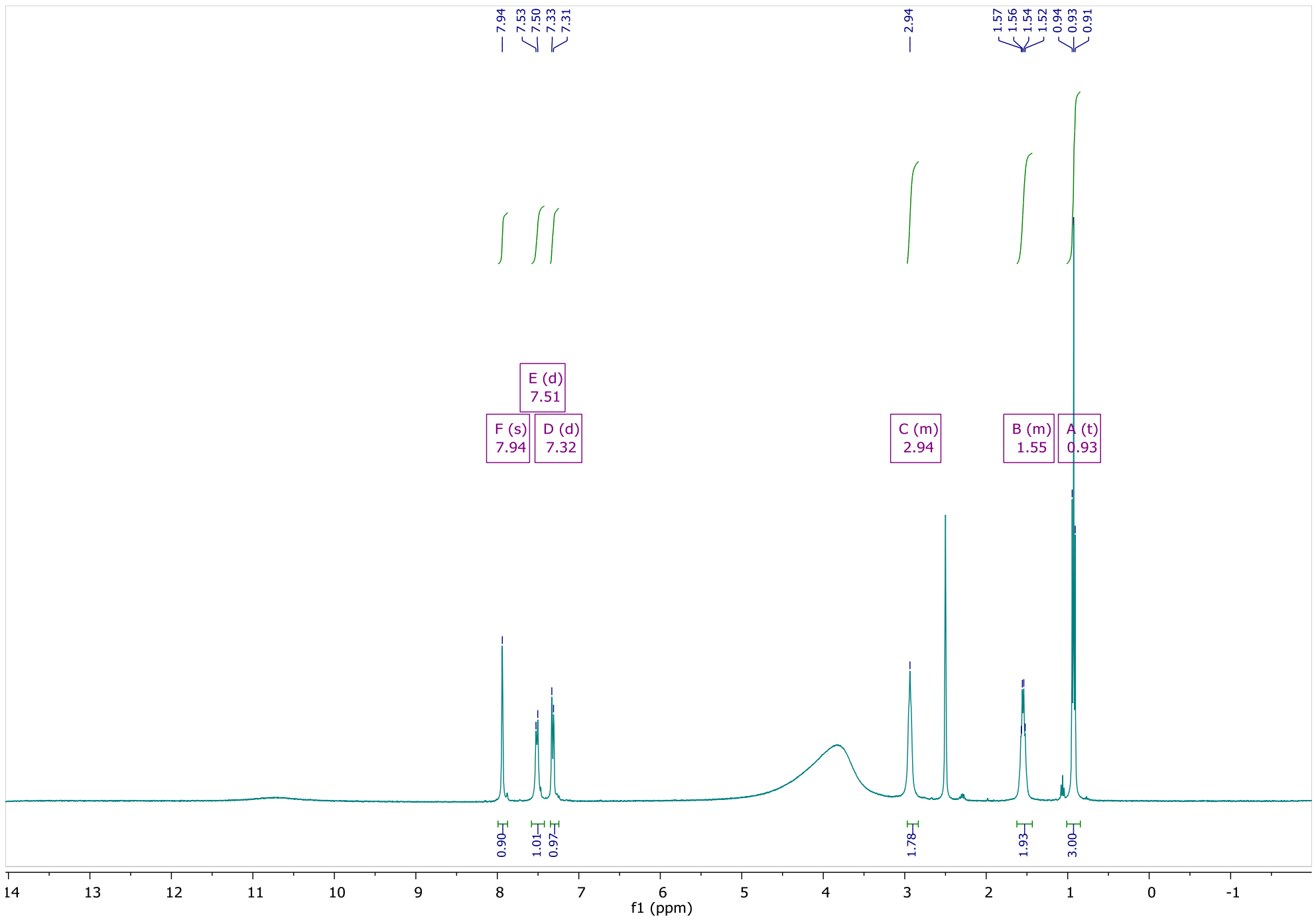

^{1}H-NMR of final compound **18** (DMSO-d_6).

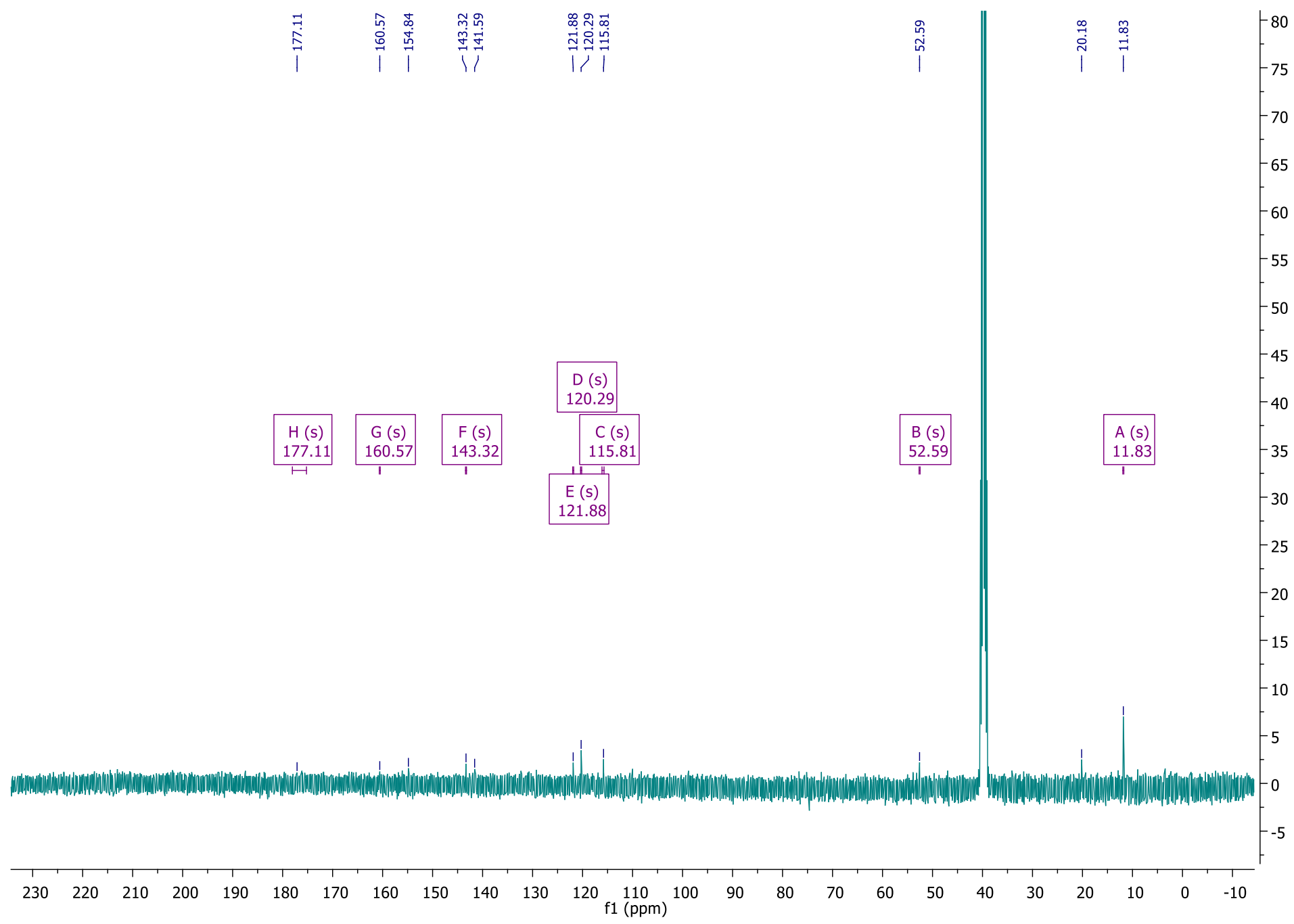

^{13}C-NMR of final compound **18** (DMSO-d_6).

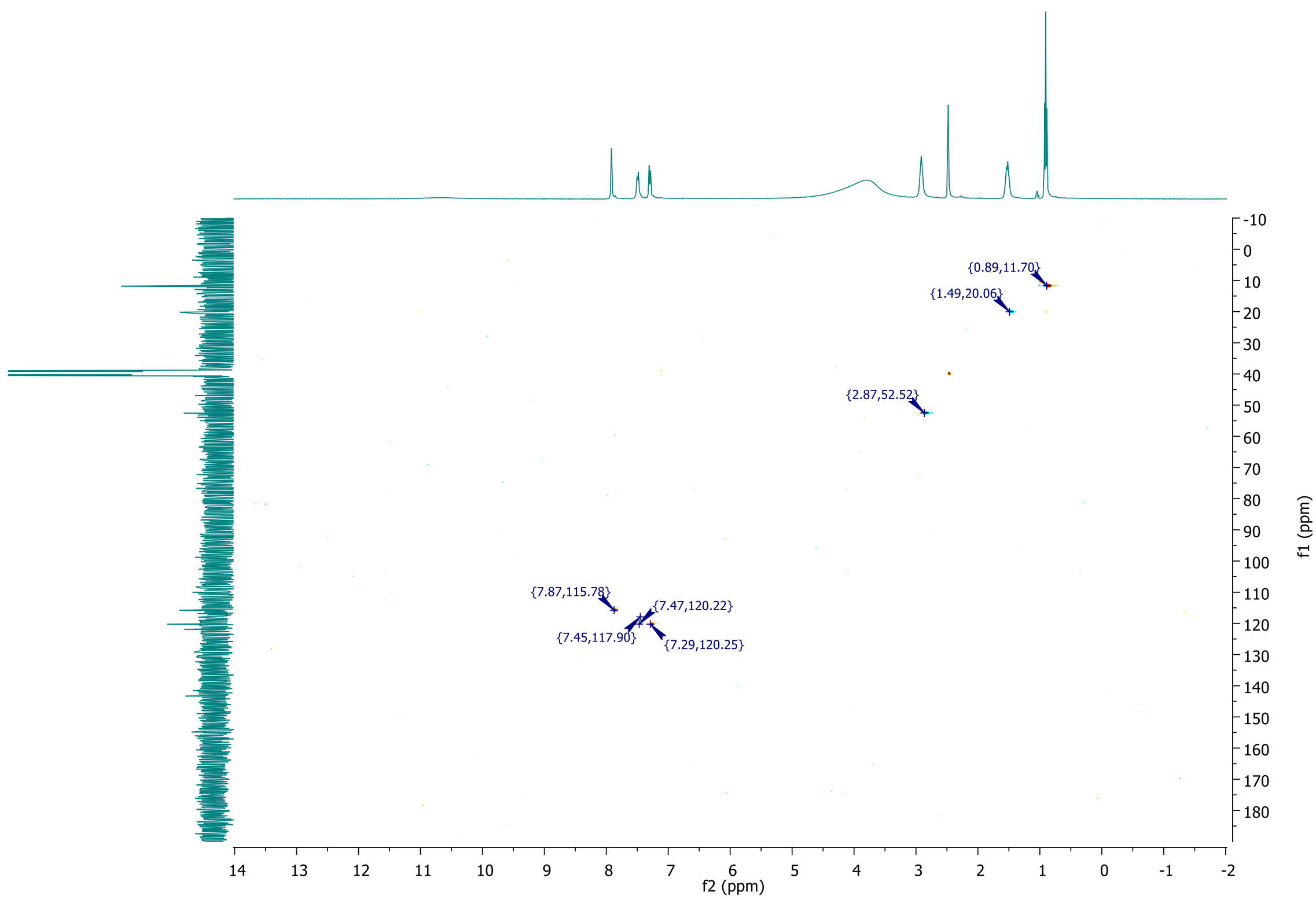

gHSQCAD of final compound **18** (DMSO-d_6).

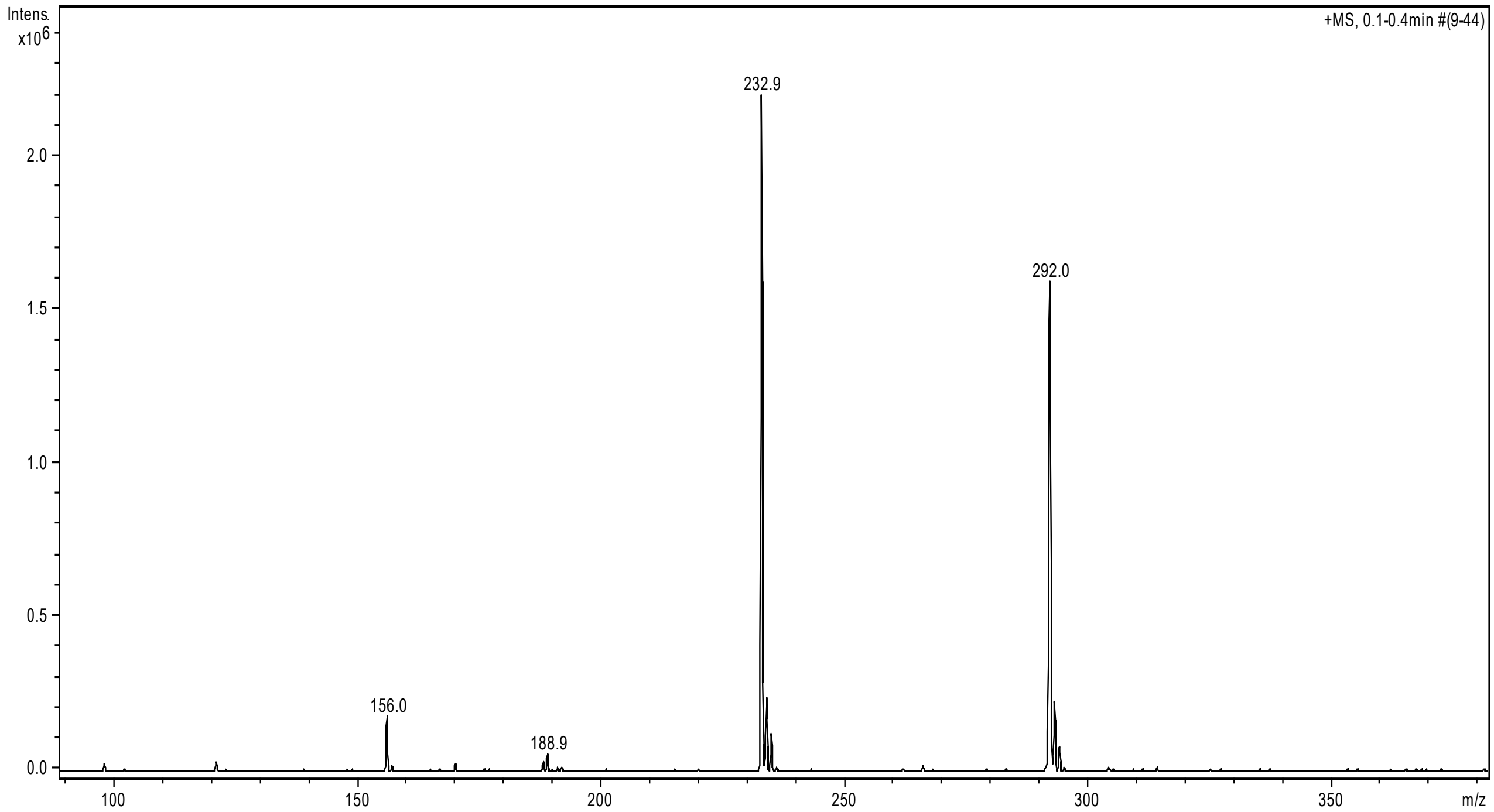

ES-MS of final compound **18** (MeOH).

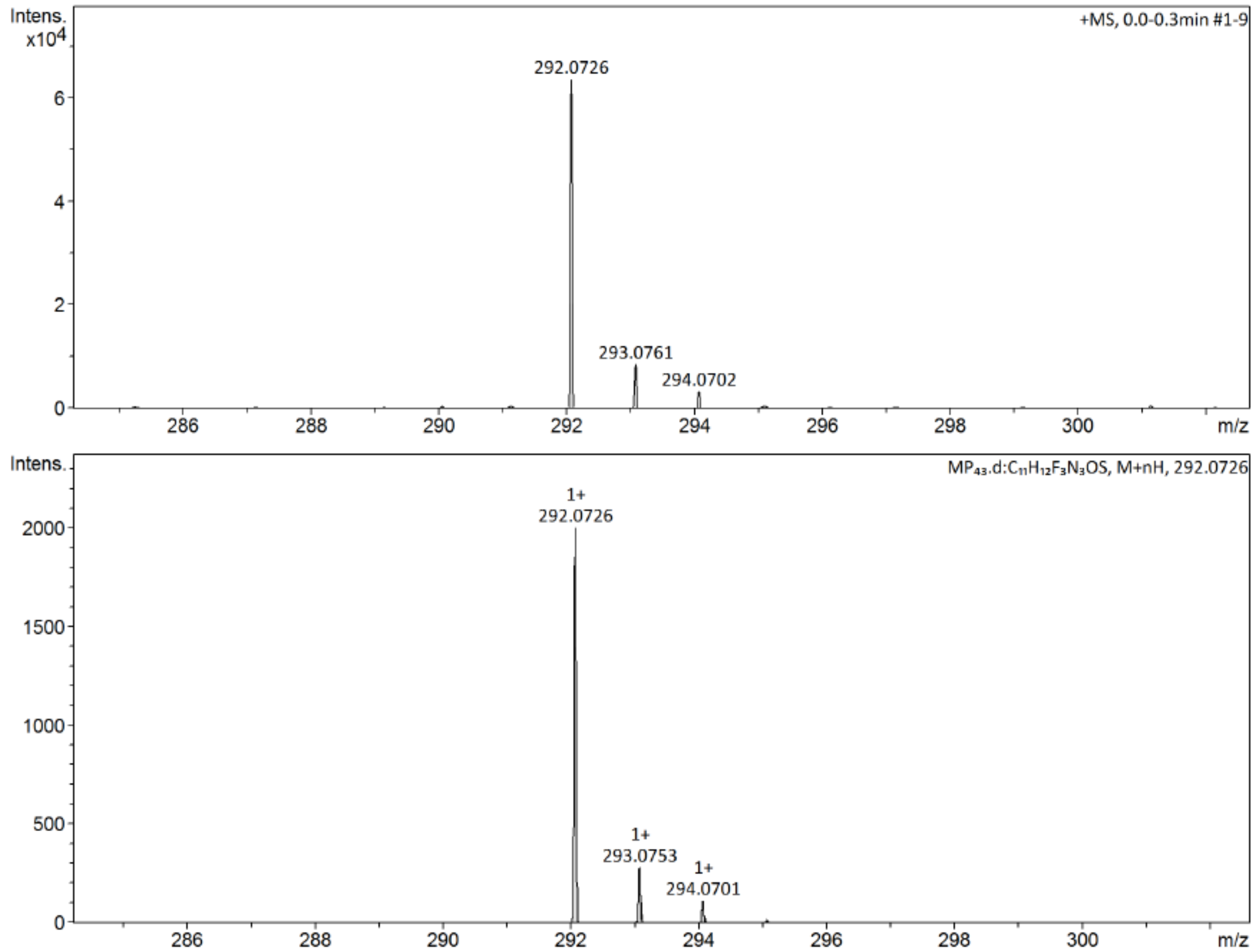

HRMS of final compound **18** (MeOH).

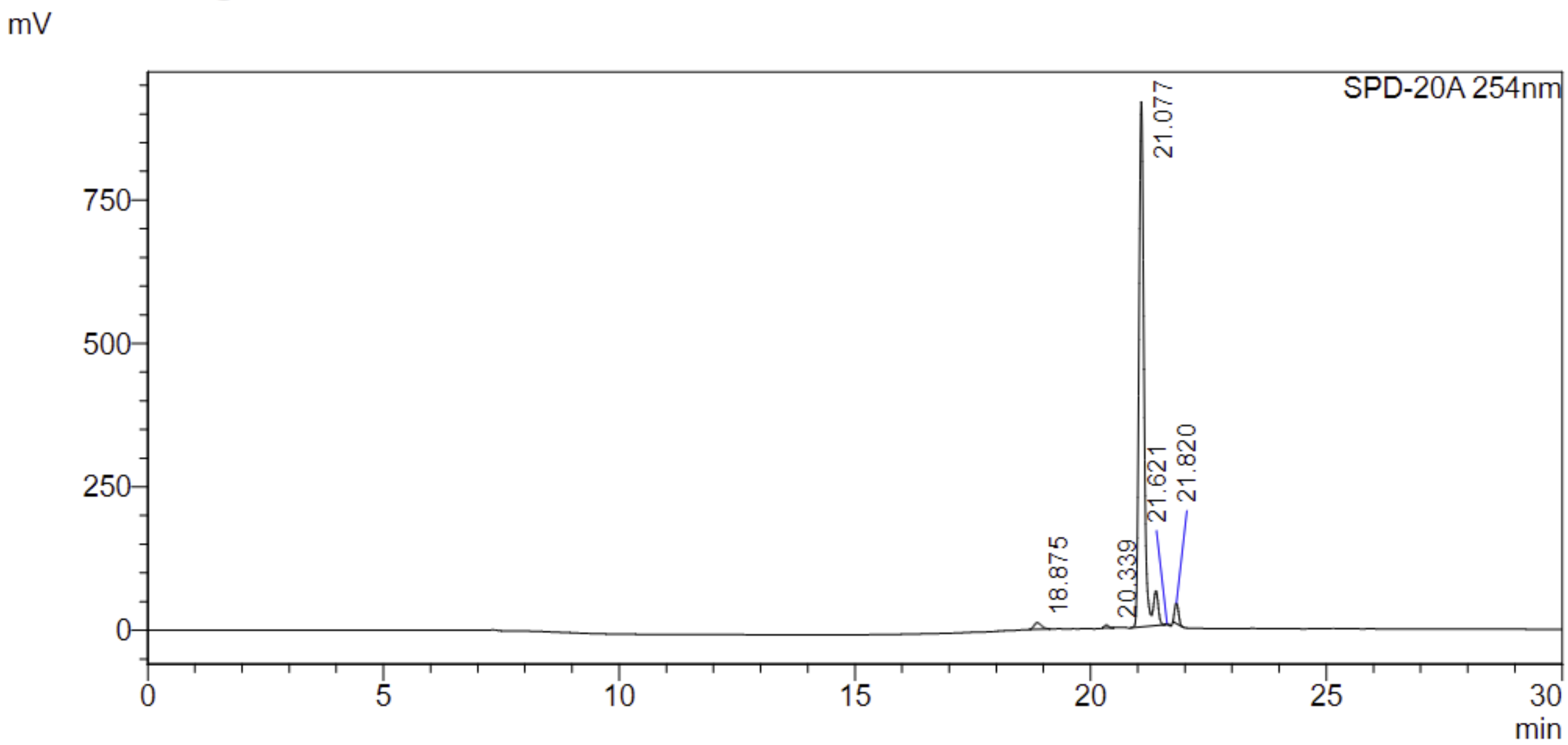

<Peak Table>

SPD-20A 254nm

Peak#	Ret. Time	Area	Height	Conc.	Unit	Mark	Name
1	18.875	124866	11478	1.674		M	
2	20.339	28541	4861	0.383		M	
3	21.077	7112762	915362	95.375		M	
4	21.621	5778	2261	0.077		M	
5	21.820	185706	35103	2.490		M	
Total		7457653	969064				

HPLC of final compound **18** (flow: 1 ml/min, eluent gradient: from water-methanol 55:45 to 15:85 in 30 min; detector: 254 nm).

2-(2-pentylhydrazineyl)-6-(trifluoromethoxy)benzo[d]thiazole (**19**)

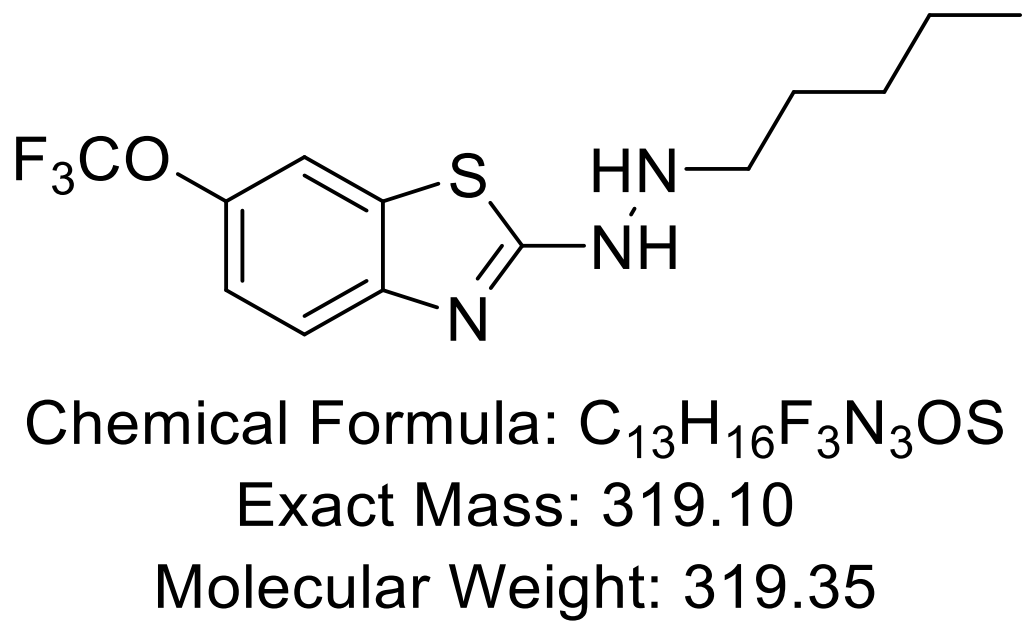

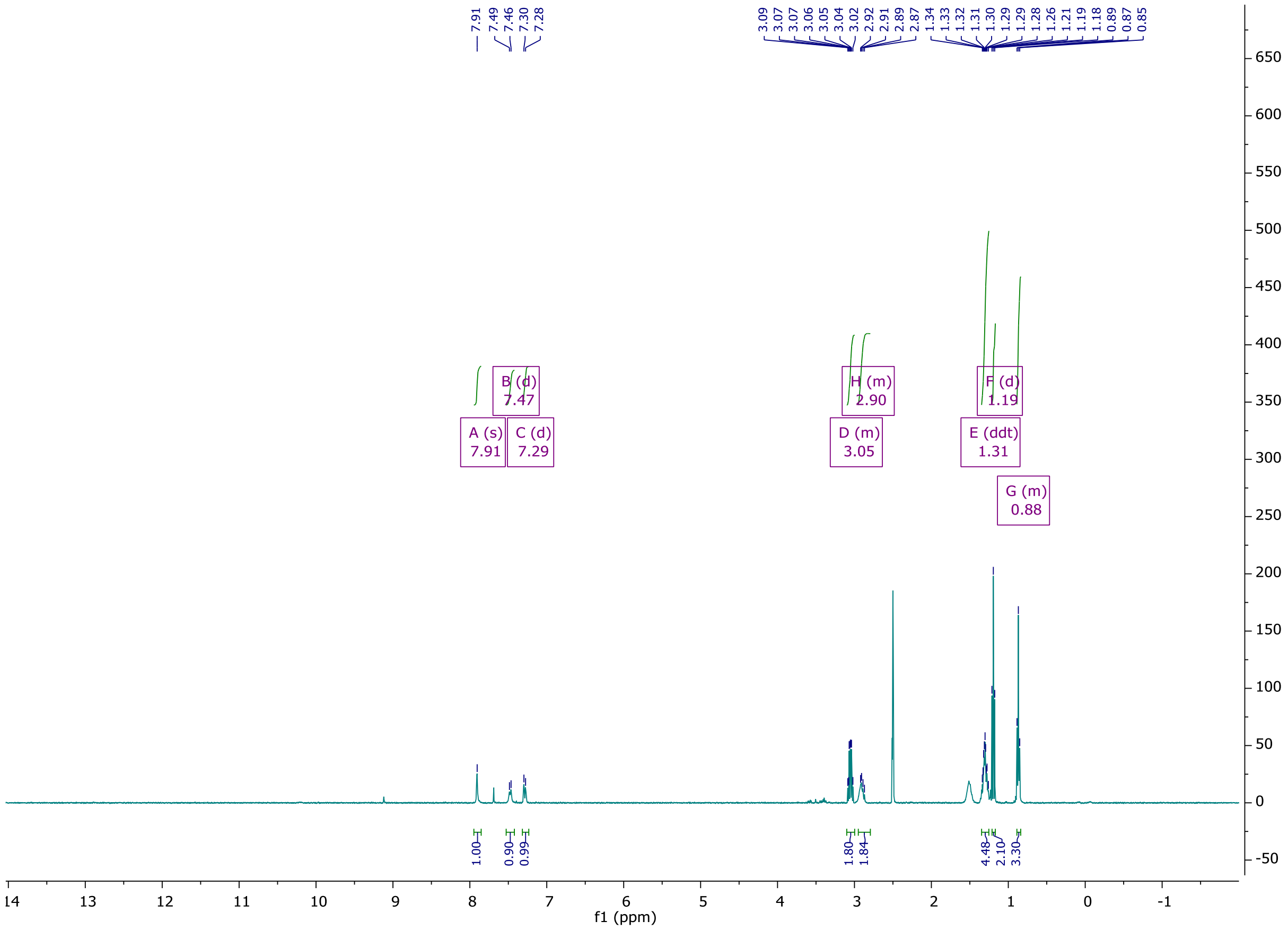

^{1}H-NMR of final compound **19** (DMSO-d_6).

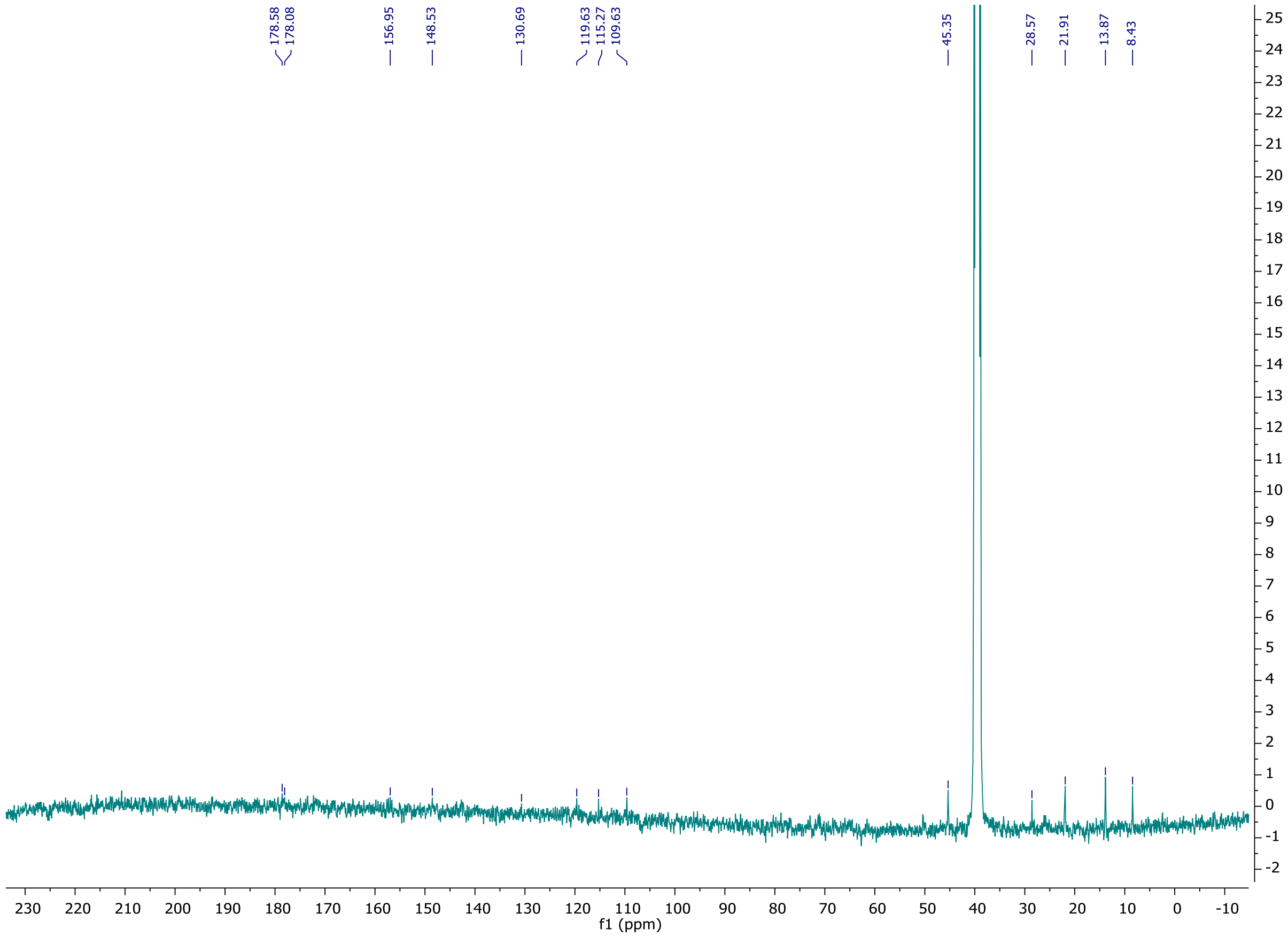

^{13}C-NMR of final compound **19** (DMSO-d_6).

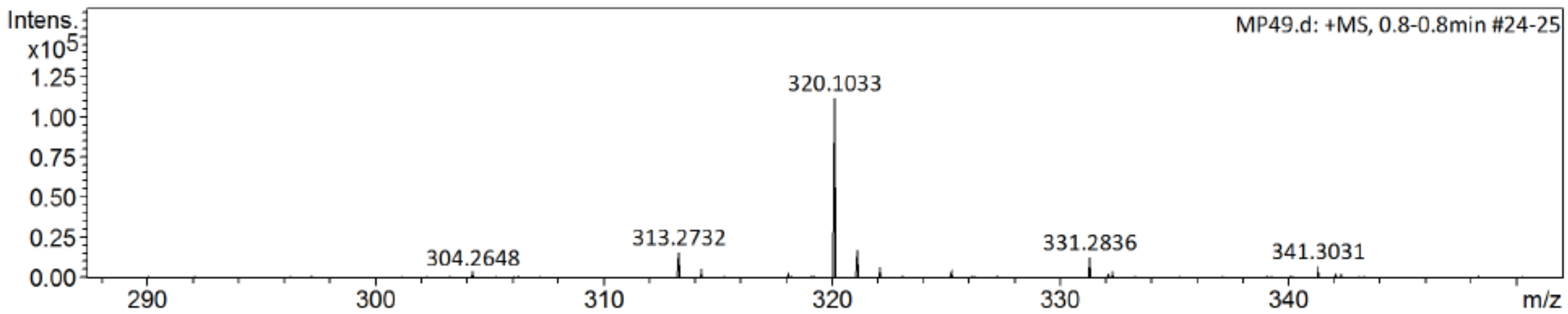

ES-MS of final compound **19** (MeOH).

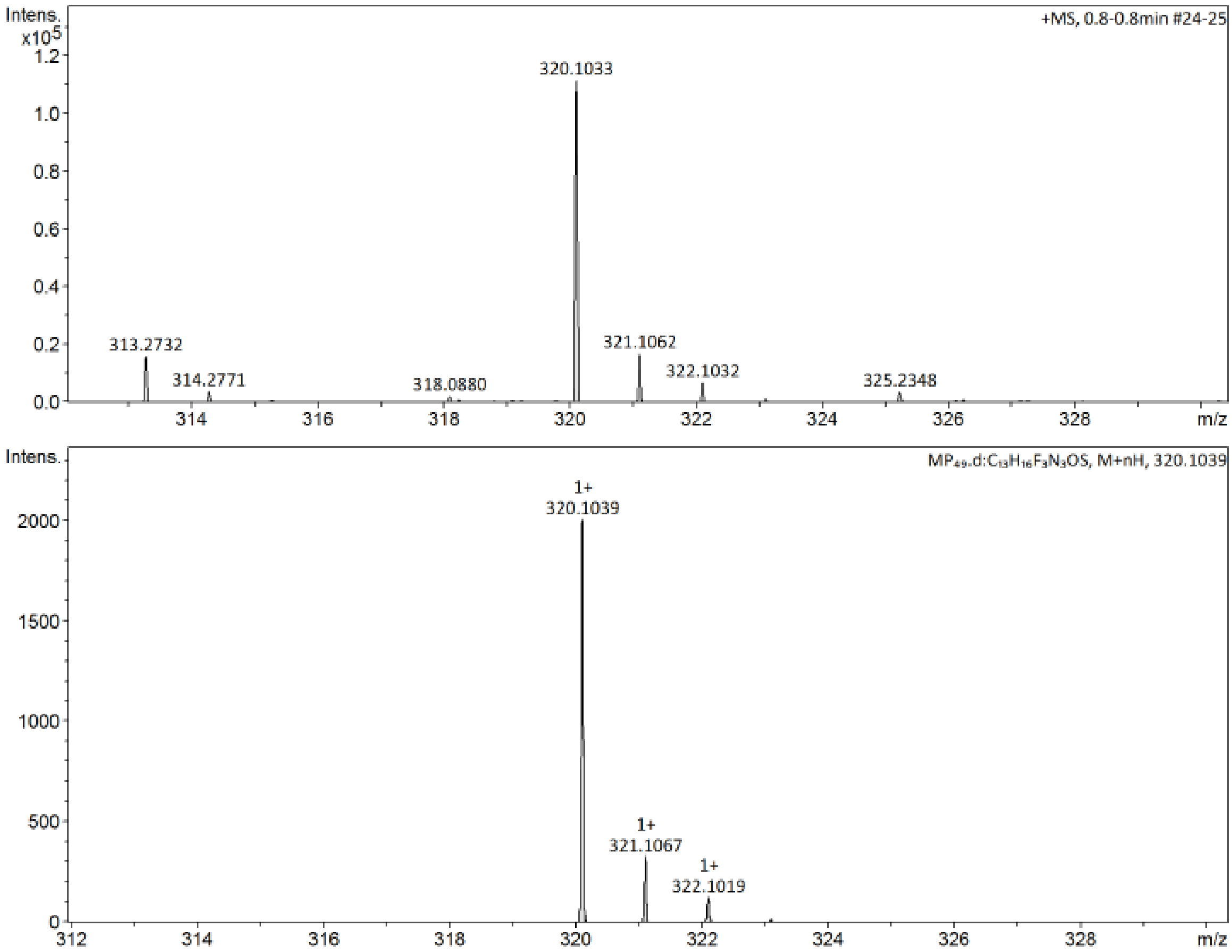

HRMS of final compound **19** (MeOH).

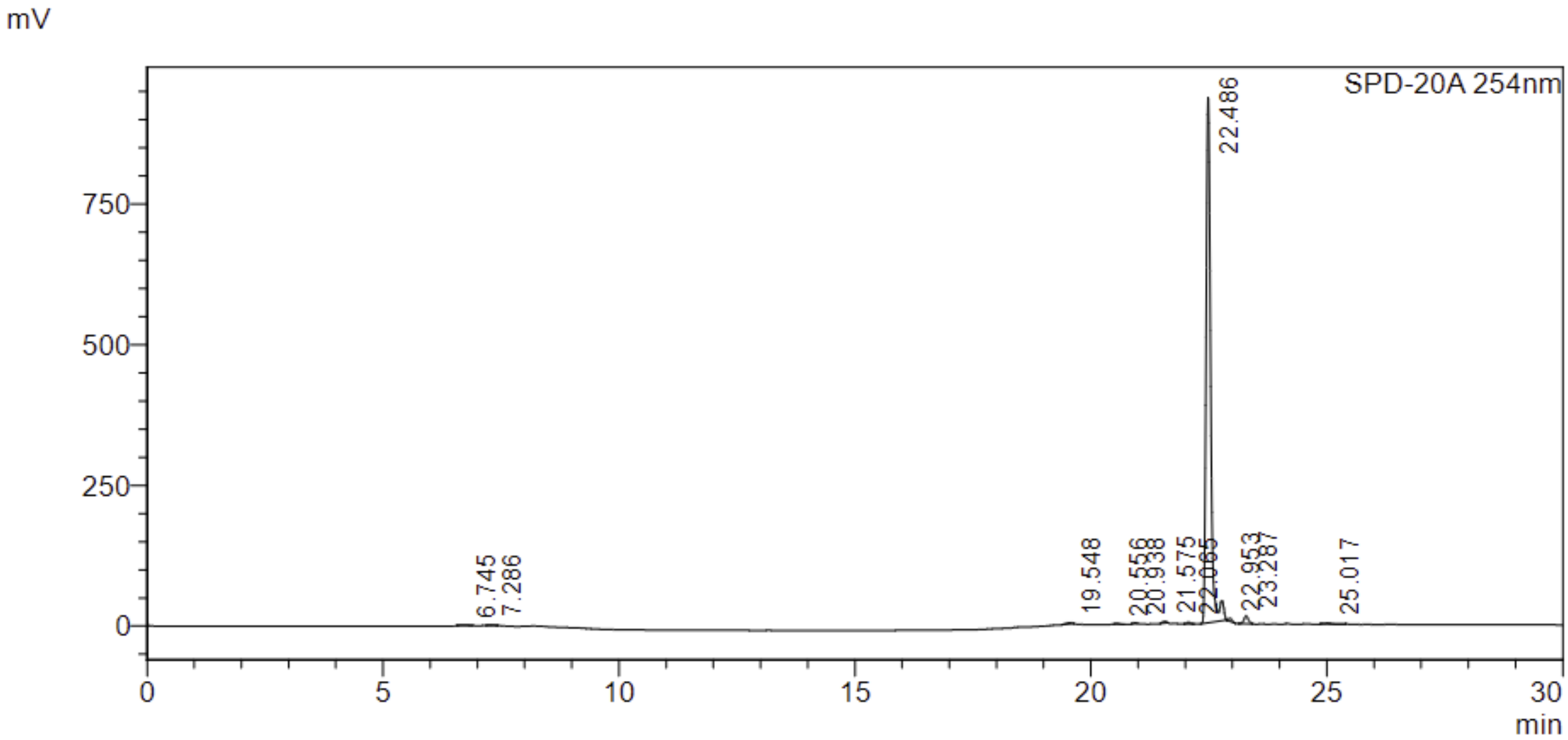

<Peak Table>

SPD-20A 254nm

Peak#	Ret. Time	Area	Height	Conc.	Unit	Mark	Name
1	6.745	9564	676	0.147		M	
2	7.286	7307	856	0.112		M	
3	19.548	24377	2428	0.375		M	
4	20.556	6826	1063	0.105		M	
5	20.938	7699	1473	0.118		M	
6	21.575	21507	3778	0.331		M	
7	22.065	18145	3138	0.279		M	
8	22.486	6279476	934059	96.645		M	
9	22.953	26513	6017	0.408		M	
10	23.287	87991	13884	1.354		M	
11	25.017	8034	1602	0.124		M	
Total		6497439	968976				

HPLC of final compound **19** (flow: 1 ml/min, eleunt gradient: from water-methanol 55:45 to 15:85 in 30 min; detector: 254 nm).

2-(2-isopentylhydrazineyl)-6-(trifluoromethoxy)benzo[*d*]thiazole hydrochloride (**20**)

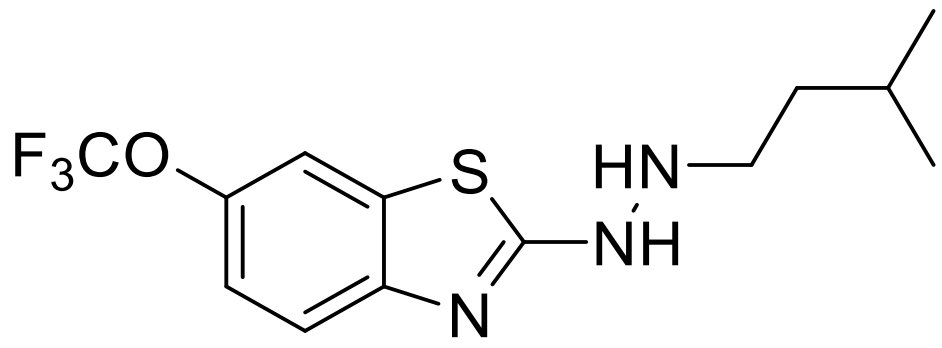

Chemical Formula: $C_{13}H_{16}F_3N_3OS$
Exact Mass: 319.10
Molecular Weight: 319.35

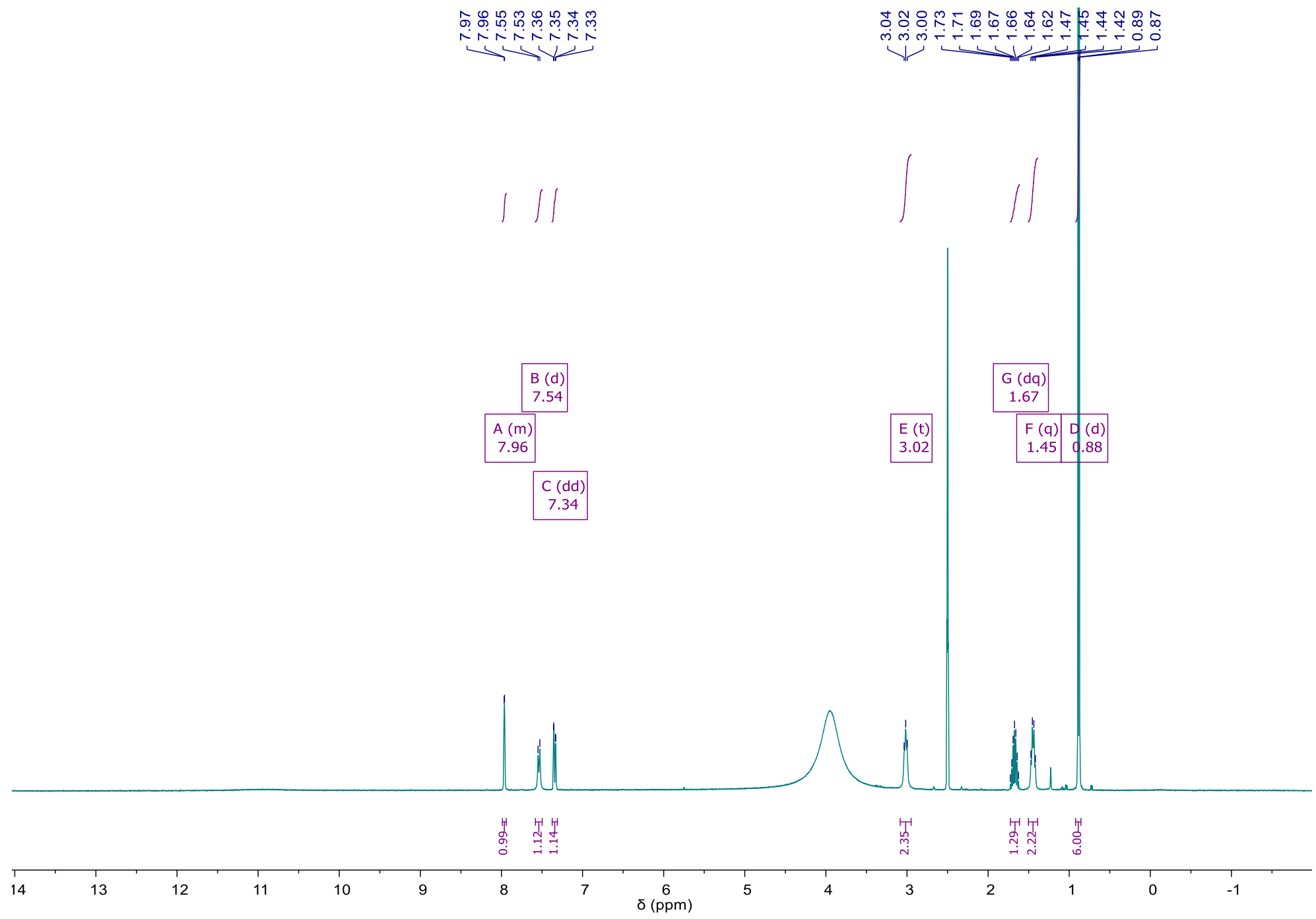

^{1}H-NMR of final compound **20** (DMSO-*d*$_6$).

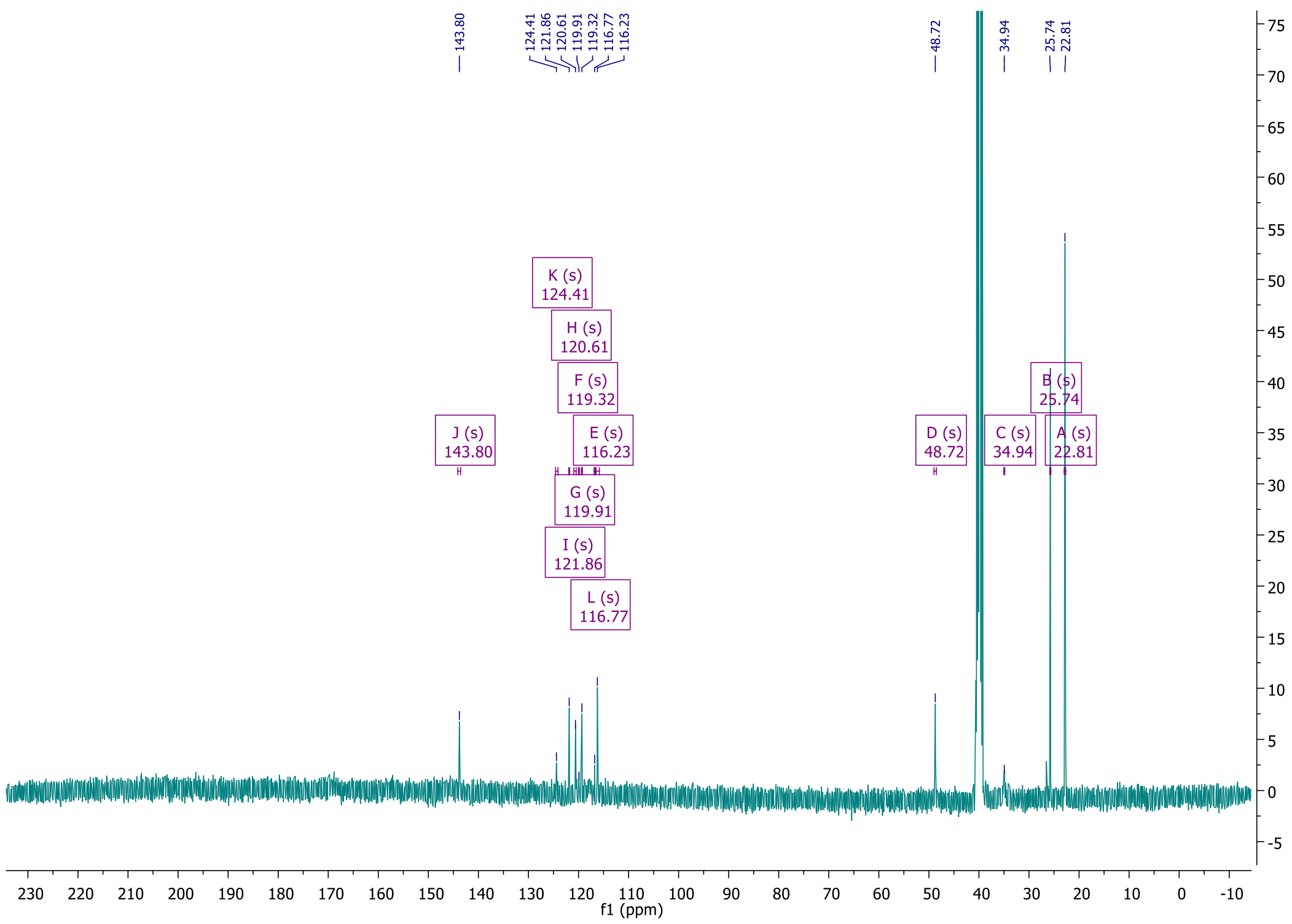

^{13}C-NMR of final compound **20** (DMSO-d_6).

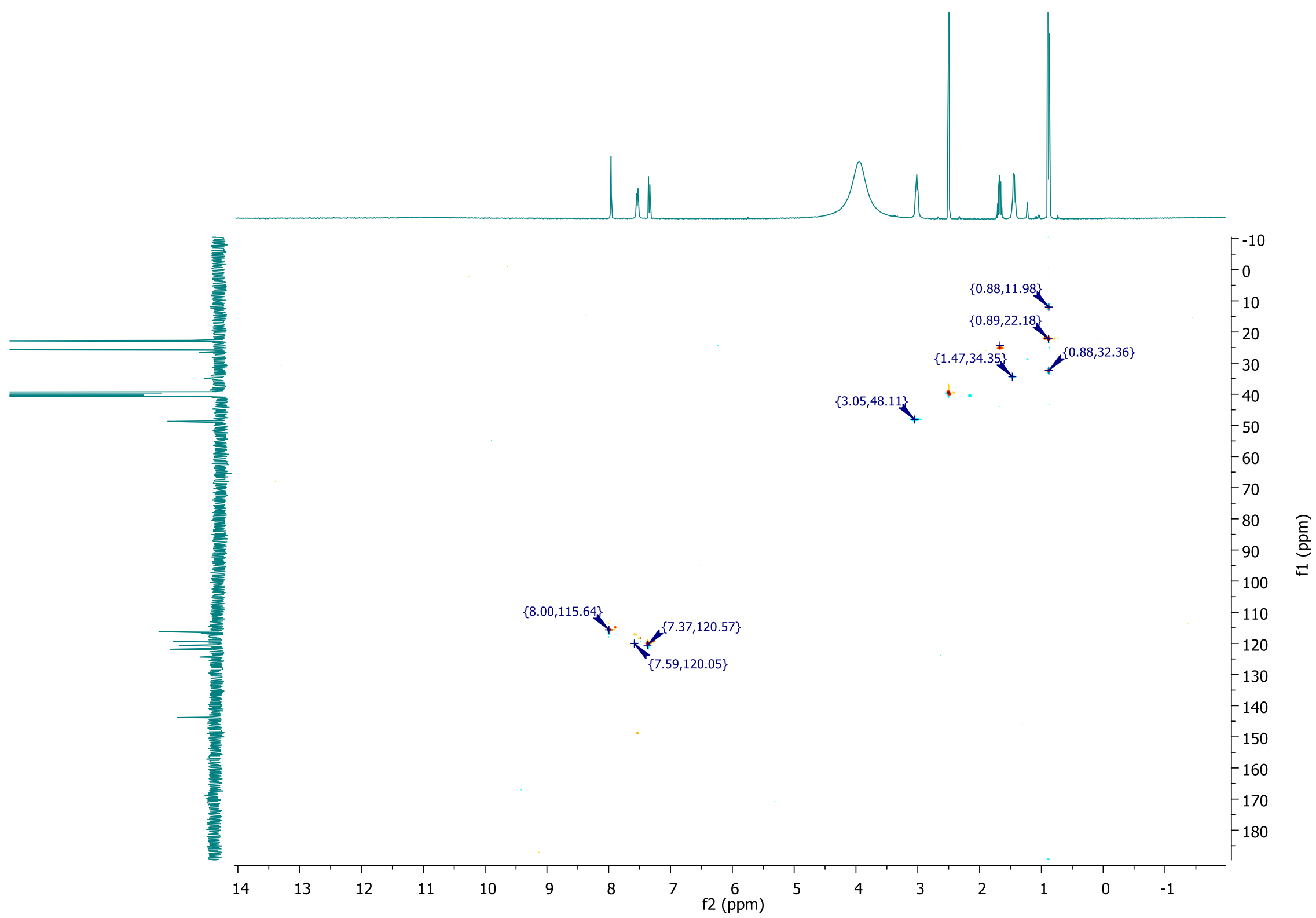

gHSQCAD of final compound **20** (DMSO-d_6).

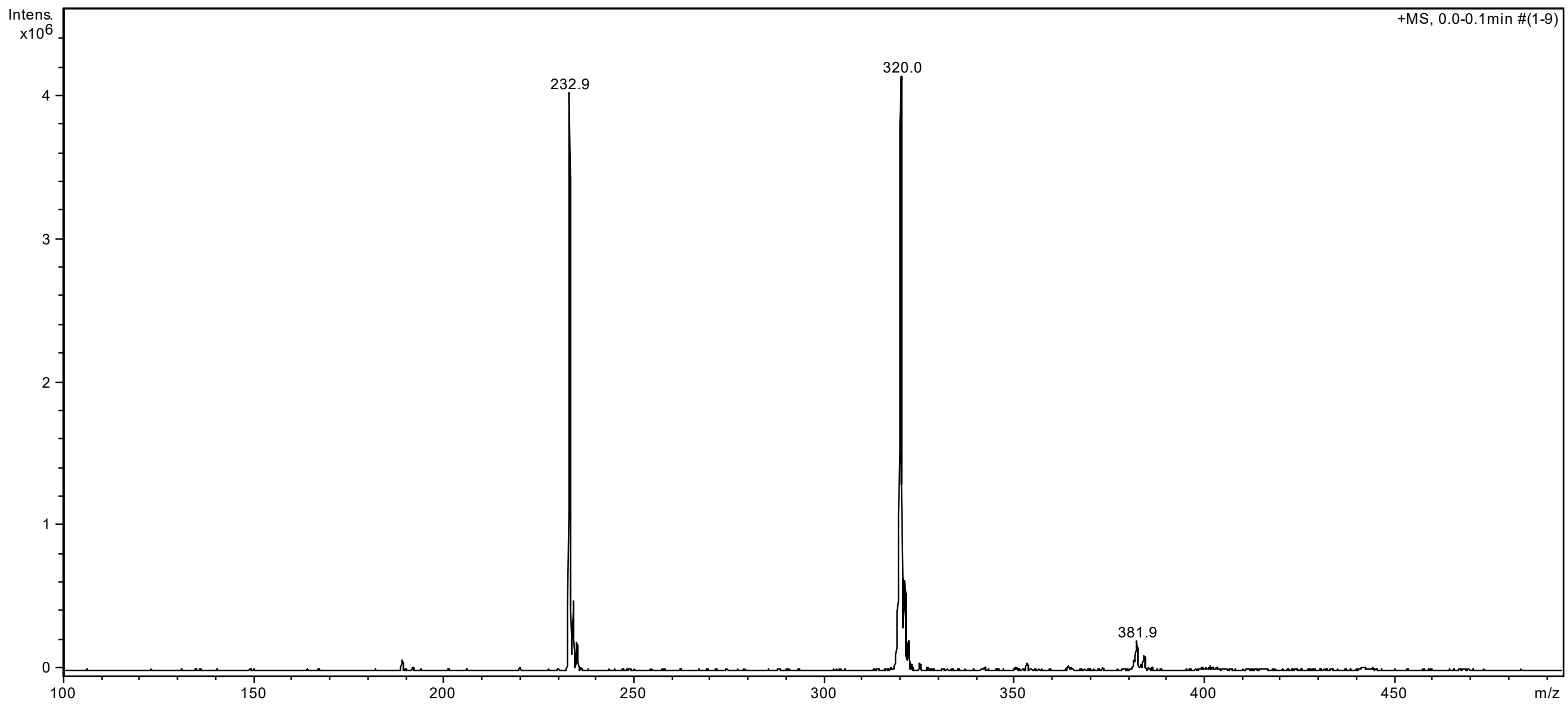

ES-MS of final compound **20** (MeOH).

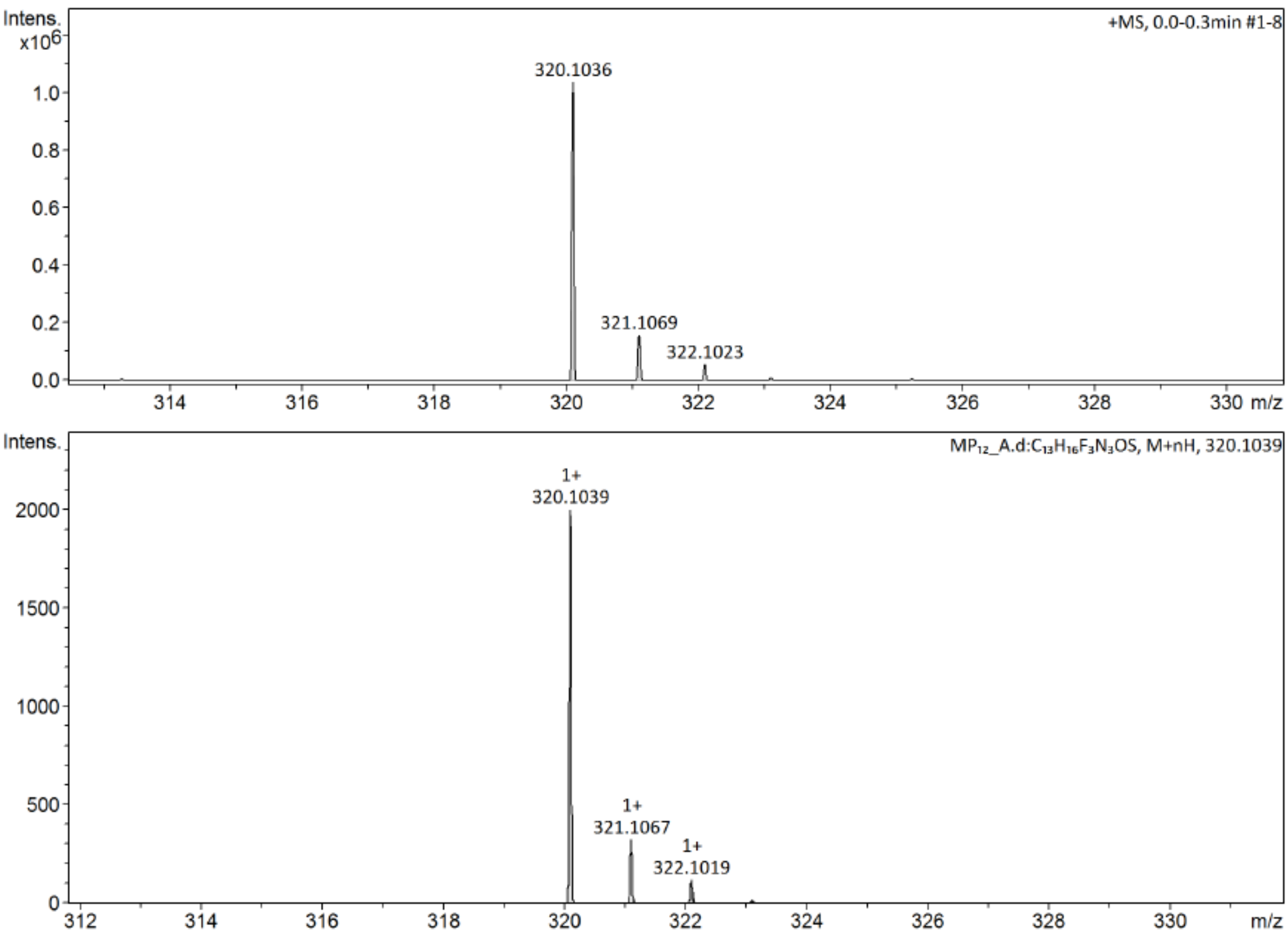

HRMS of final compound **20** (MeOH).

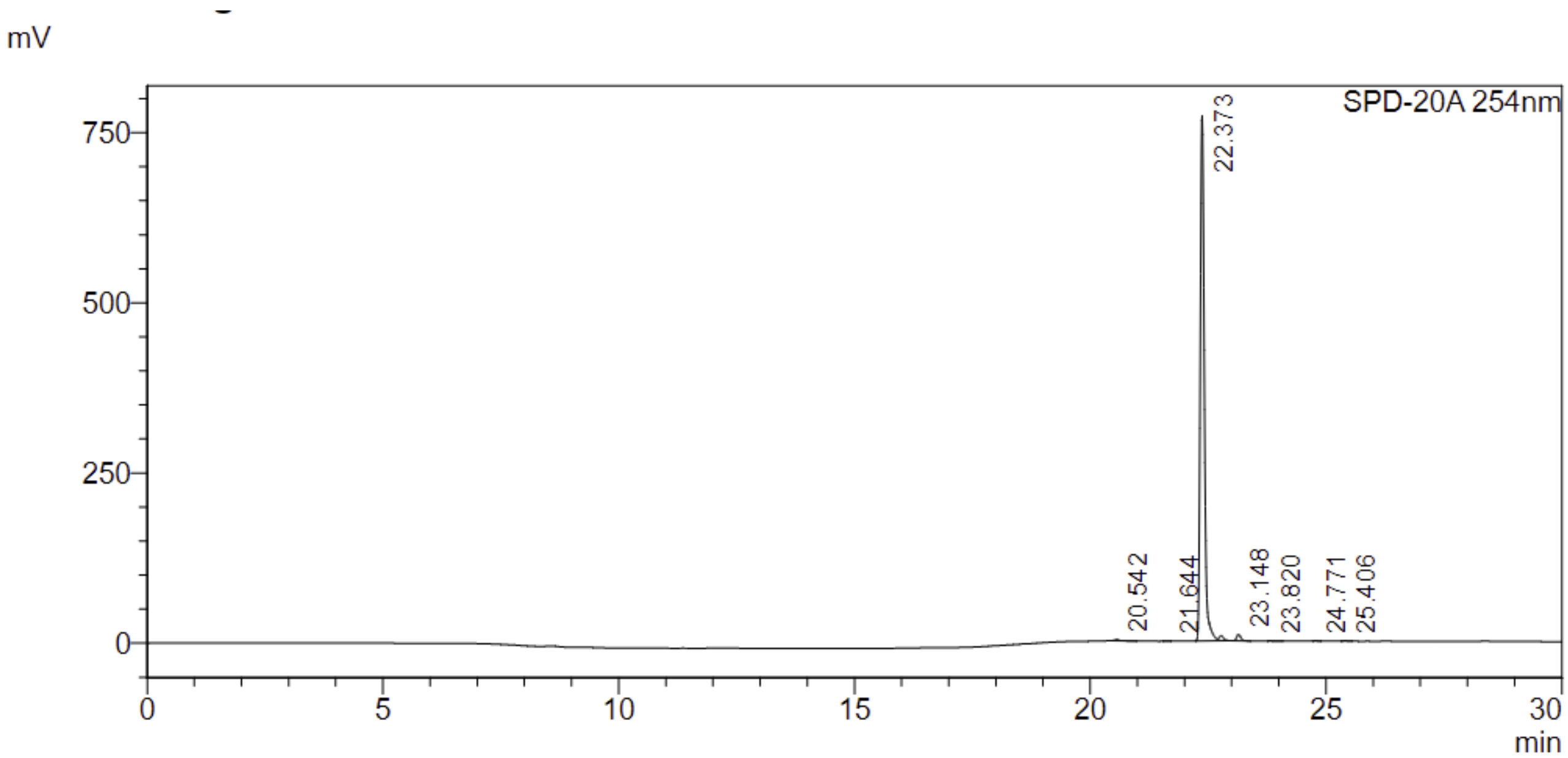

<Peak Table>

SPD-20A 254nm

Peak#	Ret. Time	Area	Height	Conc.	Unit	Mark	Name
1	20.542	13134	1638	0.276		M	
2	21.644	1119	181	0.024		M	
3	22.373	4680552	771733	98.430		M	
4	23.148	53268	9272	1.120		M	
5	23.820	1920	301	0.040		M	
6	24.771	2044	381	0.043		M	
7	25.406	3164	437	0.067		M	
Total		4755200	783944				

HPLC of final compound **20** (flow: 1 ml/min, eleunt gradient: from water-methanol 55:45 to 15:85 in 30 min; detector: 254 nm).

2-(2-(2-ethylhexyl)hydrazineyl)-6-(trifluoromethoxy)benzo[d]thiazole (**21**)

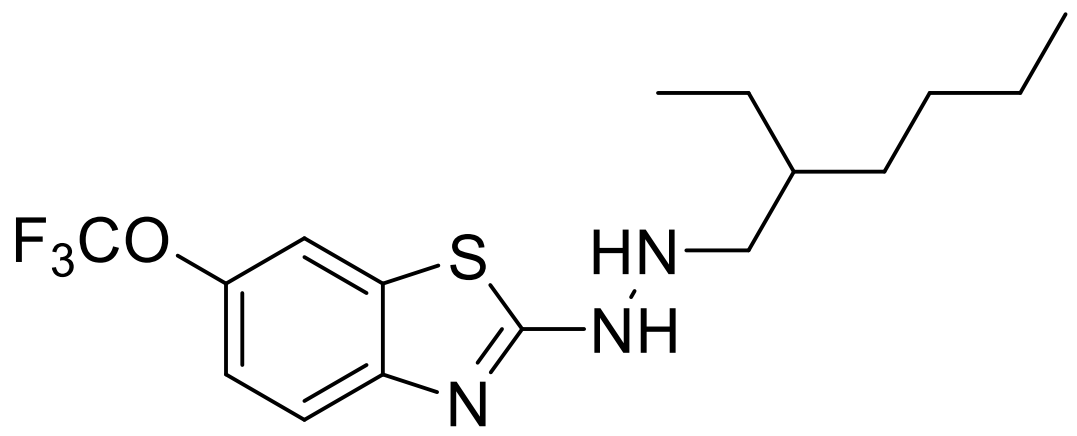

Chemical Formula: $C_{16}H_{22}F_3N_3OS$
Exact Mass: 361.14
Molecular Weight: 361.43

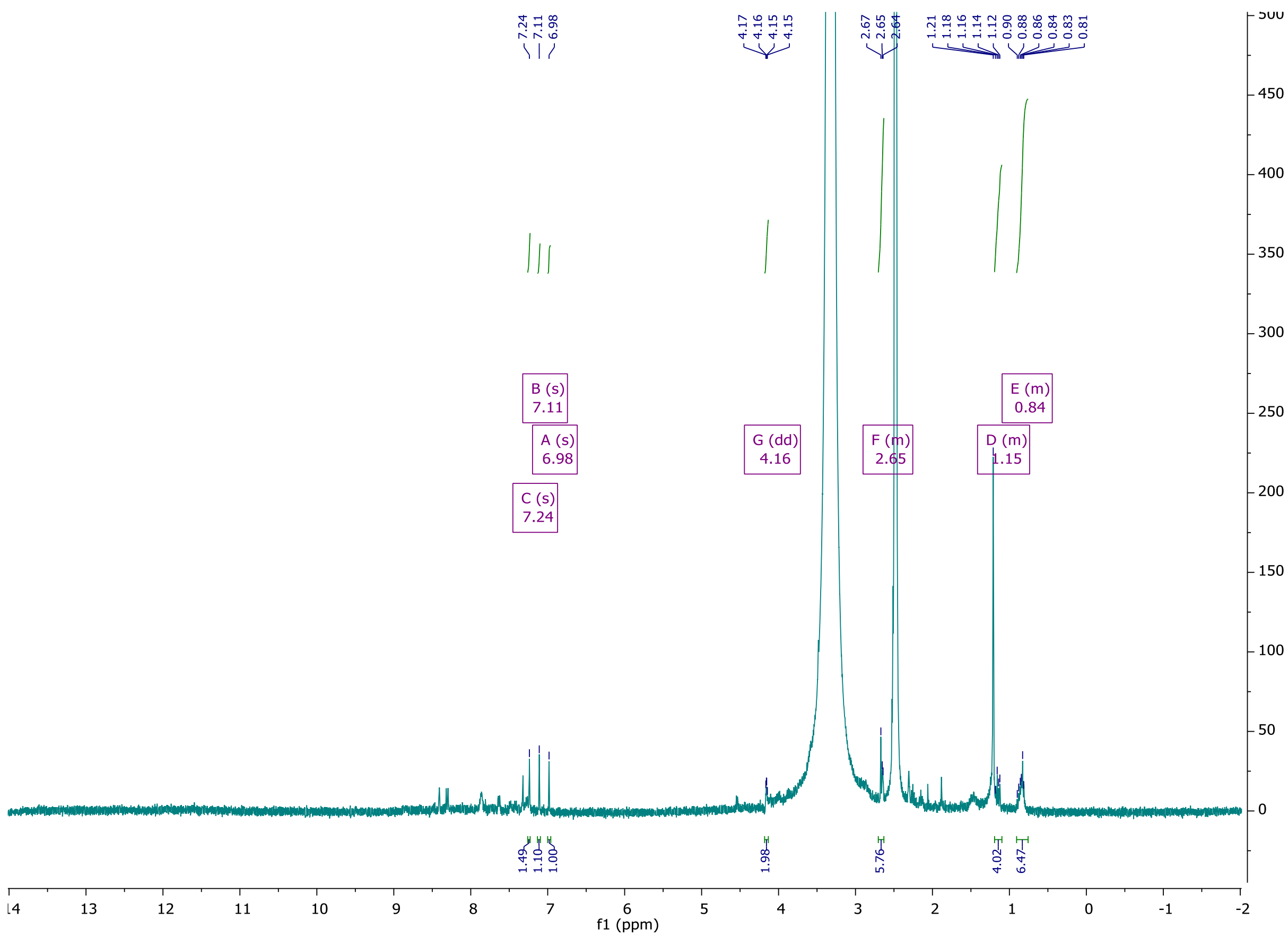

^{1}H-NMR of final compound **21** (DMSO-d_6).

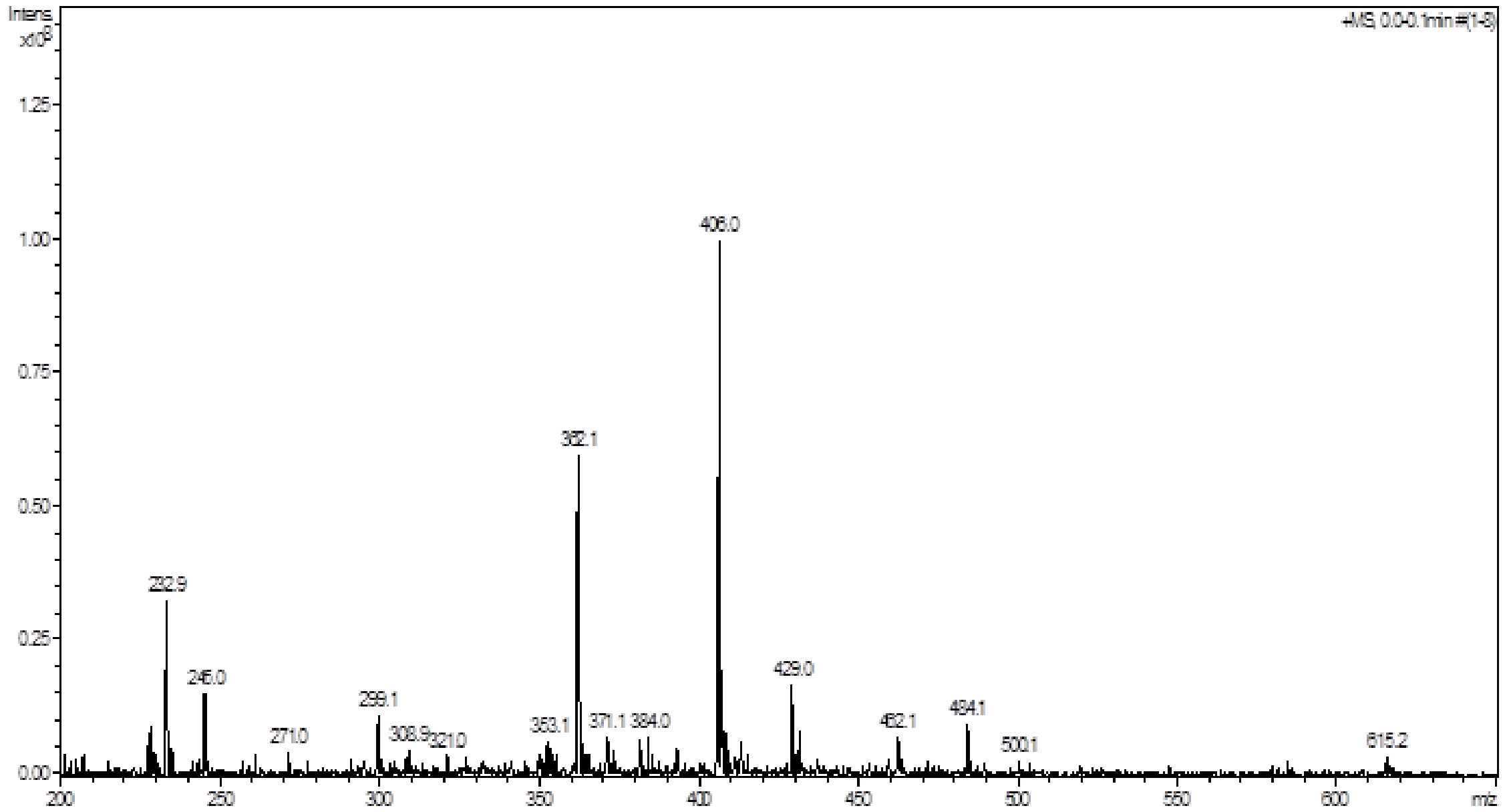

ES-MS of final compound **21** (MeOH).

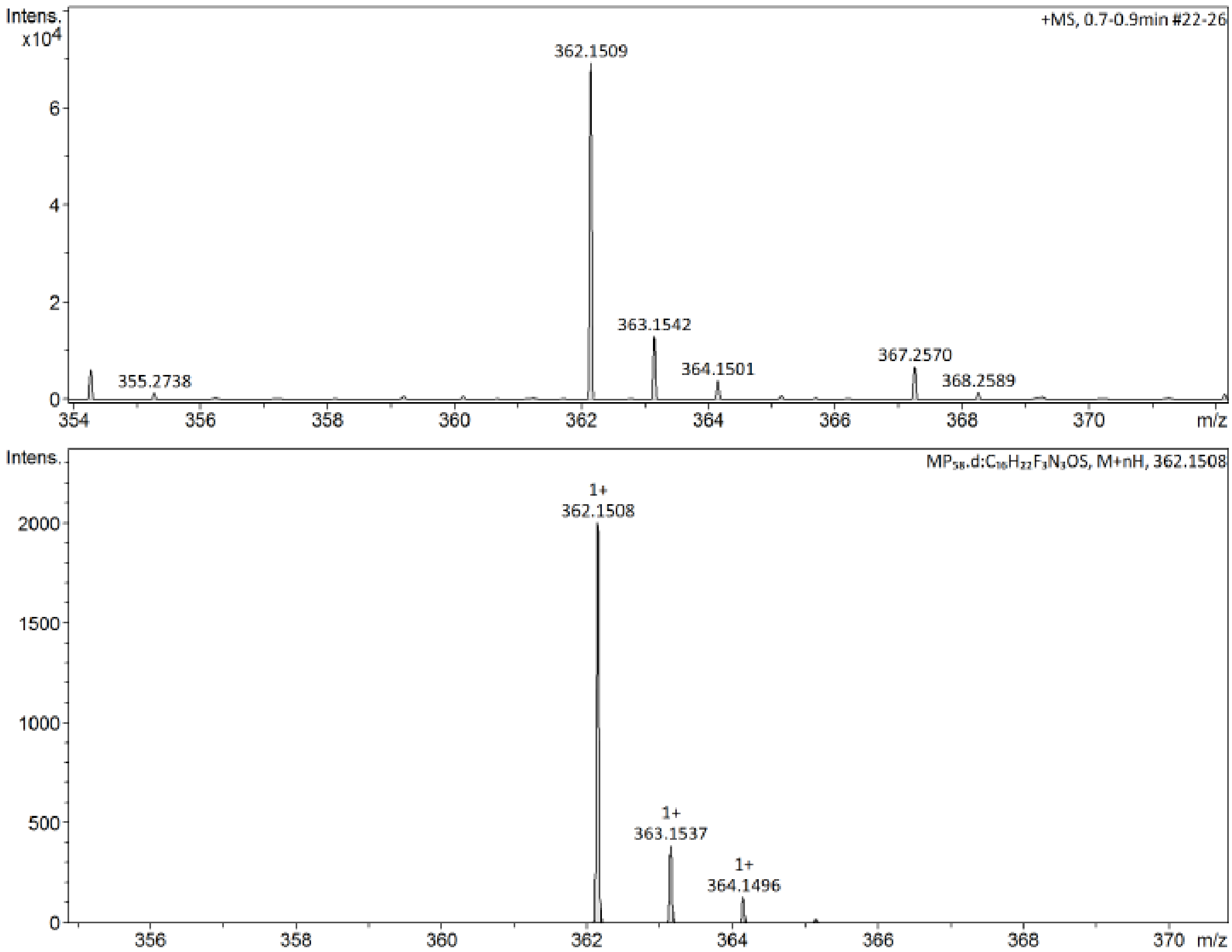

HRMS of final compound **21** (MeOH).

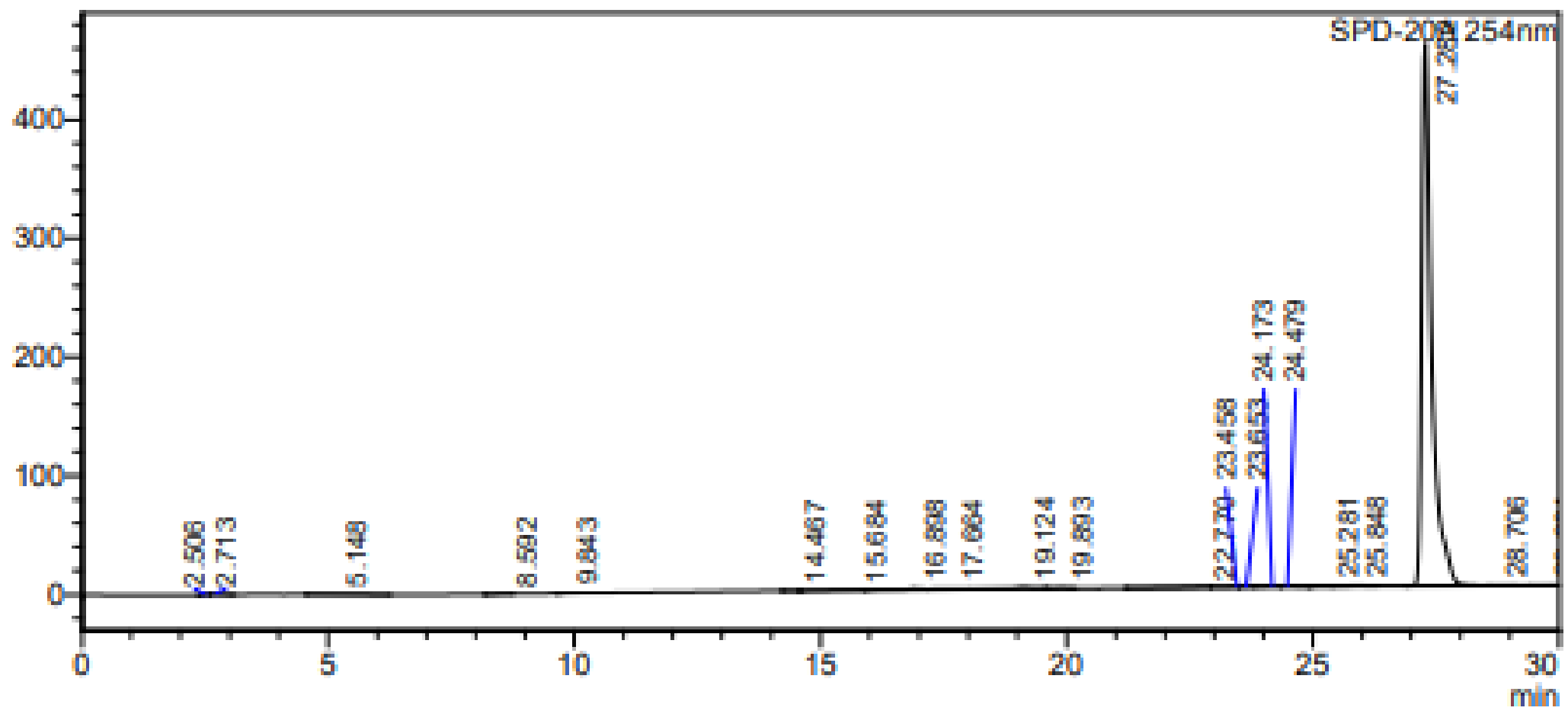

<Peak Table>

SPD-20A 254nm

Peak#	Ret. Time	Area	Height	Conc.	Unit	Mark	Name
1	2.506	5232	550	0.000			
2	2.713	9326	810	0.000		V	
3	5.148	10210	217	0.000			
4	8.592	1040	40	0.000			
5	9.843	2751	225	0.000			
6	14.467	1114	82	0.000			
7	15.684	16023	361	0.000		V	
8	16.898	28851	1029	0.000		V	
9	17.664	23298	539	0.000		V	
10	19.124	27526	915	0.000		V	
11	19.893	3898	281	0.000		V	
12	22.770	15572	412	0.000			
13	23.458	15518	558	0.000		V	
14	23.653	10909	663	0.000		V	
15	24.173	17845	642	0.000		V	
16	24.479	11252	501	0.000		V	
17	25.281	5219	198	0.000		V	
18	25.848	10065	558	0.000		V	
19	27.282	6201745	456475	0.000		S	
20	28.706	2540	201	0.000		T	
21	29.684	1224	57	0.000		TV	
Total		6421161	465314				

HPLC of final compound **21** (flow: 1 ml/min, eleunt gradient: from water-methanol 55:45 to 15:85 in 30 min; detector: 254 nm).

2-(2-phenylhydrazineyl)-6-(trifluoromethoxy)benzo[d]thiazole (**22**)

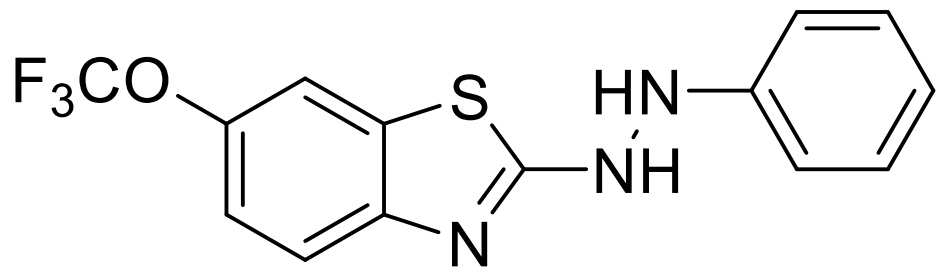

Chemical Formula: $C_{14}H_{10}F_3N_3OS$
Exact Mass: 325.05
Molecular Weight: 325.31

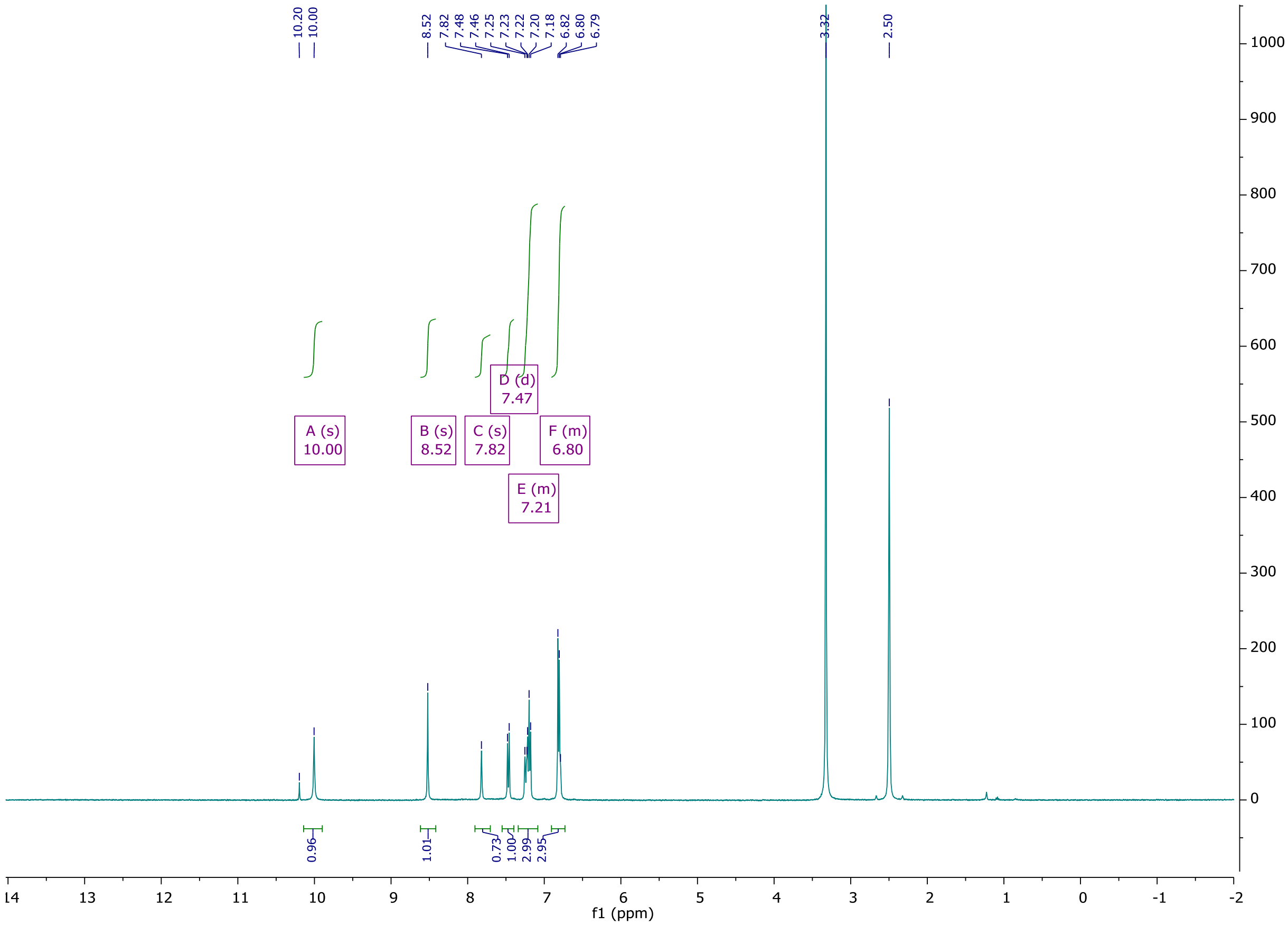

^{1}H-NMR of final compound **22** (DMSO-d_6).

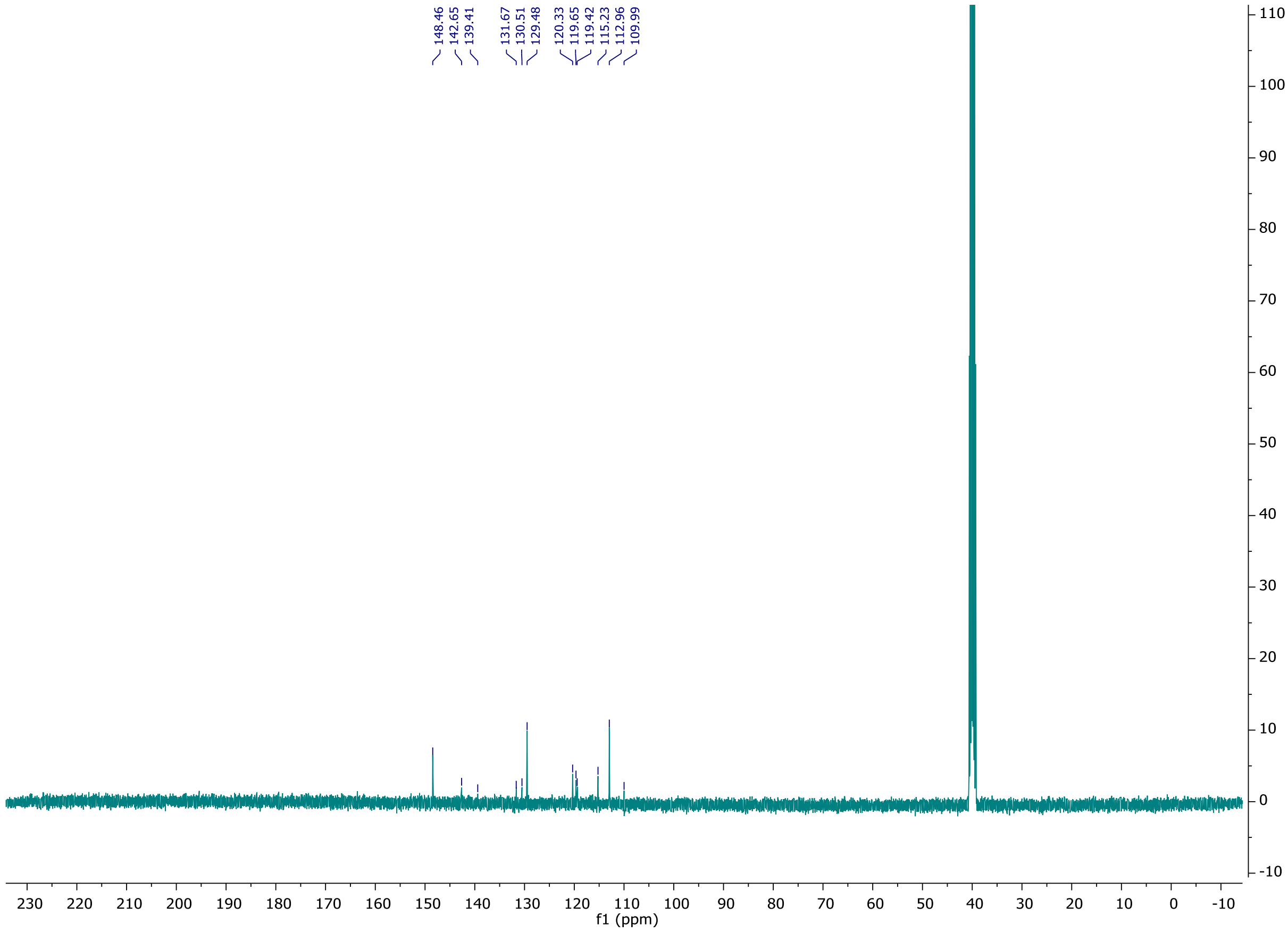

^{13}C-NMR of final compound **22** (DMSO-d_6).

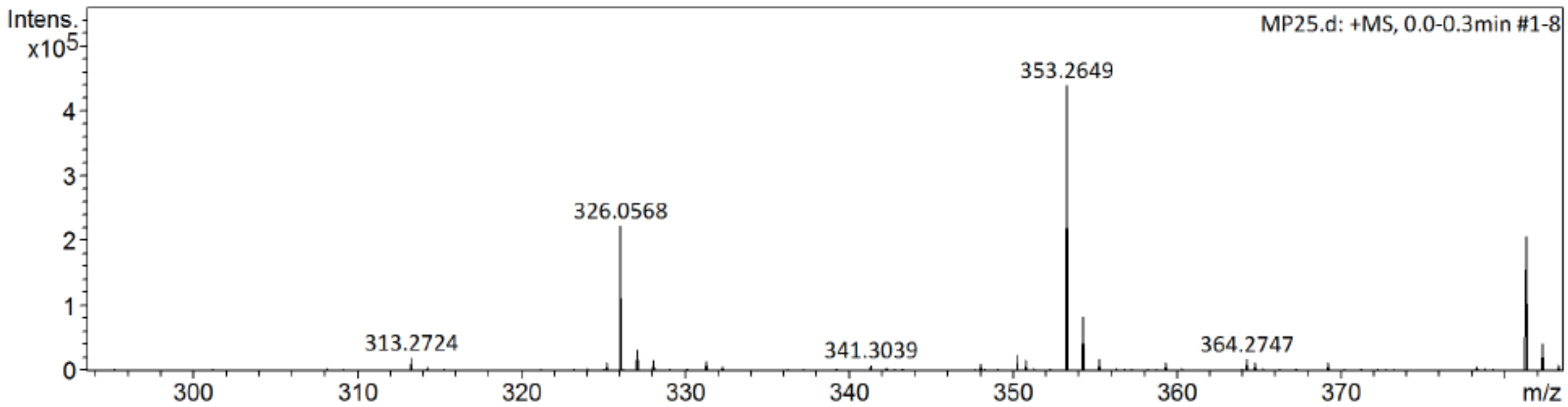

ES-MS of final compound **22** (MeOH).

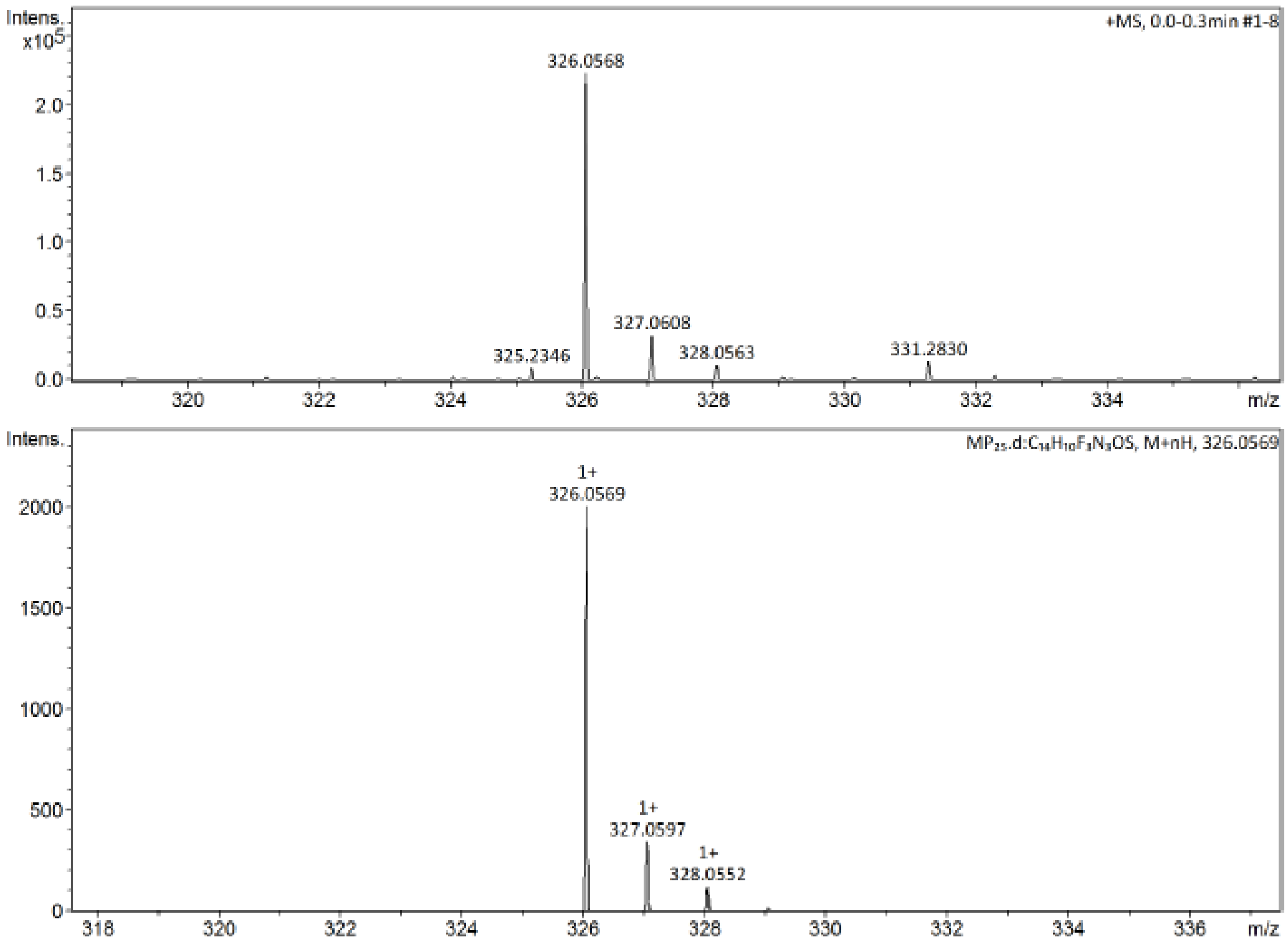

HRMS of final compound **22** (MeOH).

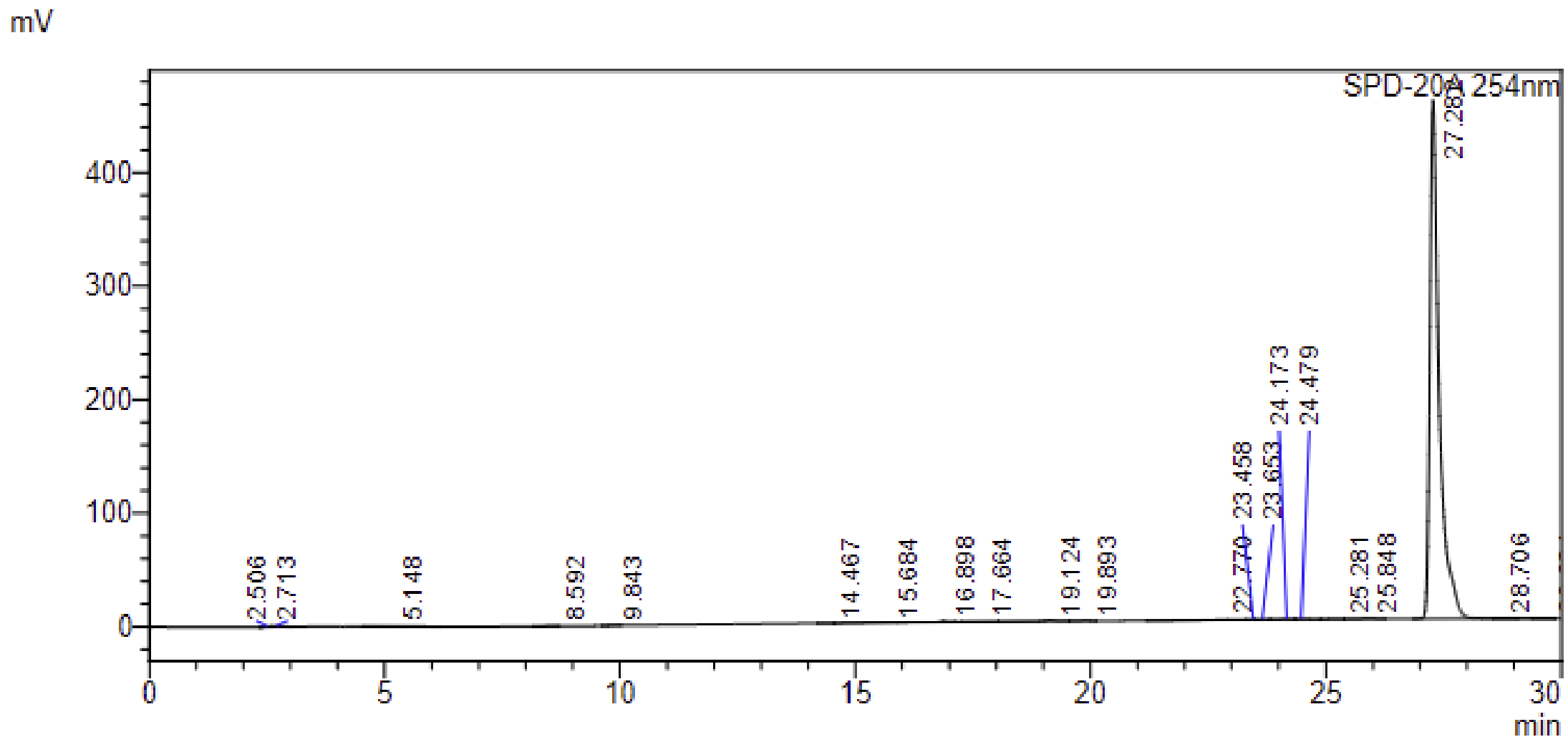

<Peak Table>

SPD-20A 254nm

Peak#	Ret. Time	Area	Height	Conc.	Unit	Mark	Name
1	2.506	5232	550	0.000			
2	2.713	9326	810	0.000		V	
3	5.148	10210	217	0.000			
4	8.592	1040	40	0.000			
5	9.843	2751	225	0.000			
6	14.467	1114	82	0.000			
7	15.684	16023	361	0.000		V	
8	16.898	28851	1029	0.000		V	
9	17.664	23298	539	0.000		V	
10	19.124	27526	915	0.000		V	
11	19.893	3898	281	0.000		V	
12	22.770	15572	412	0.000			
13	23.458	15518	558	0.000		V	
14	23.653	10909	663	0.000		V	
15	24.173	17845	642	0.000		V	
16	24.479	11252	501	0.000		V	
17	25.281	5219	198	0.000		V	
18	25.848	10065	558	0.000		V	
19	27.282	6201745	456475	0.000		S	
20	28.706	2540	201	0.000		T	
21	29.684	1224	57	0.000		TV	
Total		6421161	465314				

HPLC of final compound **22** (flow: 1 ml/min, eleunt gradient: from water-methanol 55:45 to 15:85 in 30 min; detector: 254 nm).

2-(2-benzylhydrazineyl)-6-(trifluoromethoxy)benzo[*d*]thiazole hydrochloride (**23**)

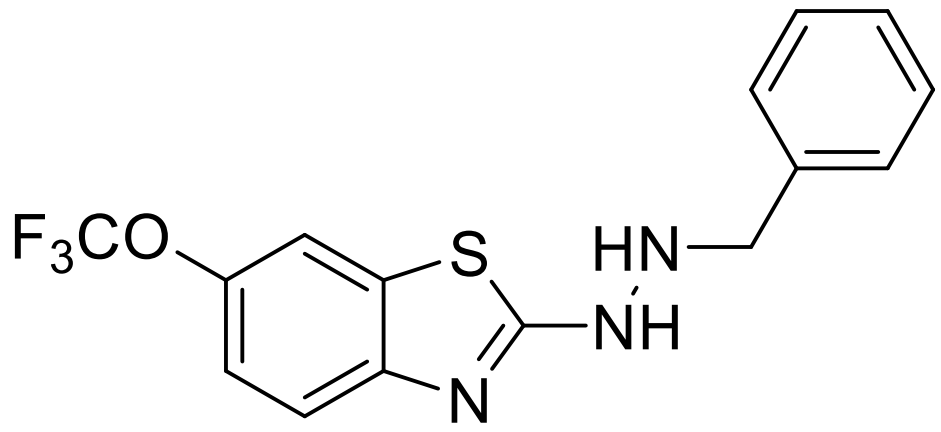

Chemical Formula: $C_{15}H_{12}F_3N_3OS$
Exact Mass: 339.07
Molecular Weight: 339.34

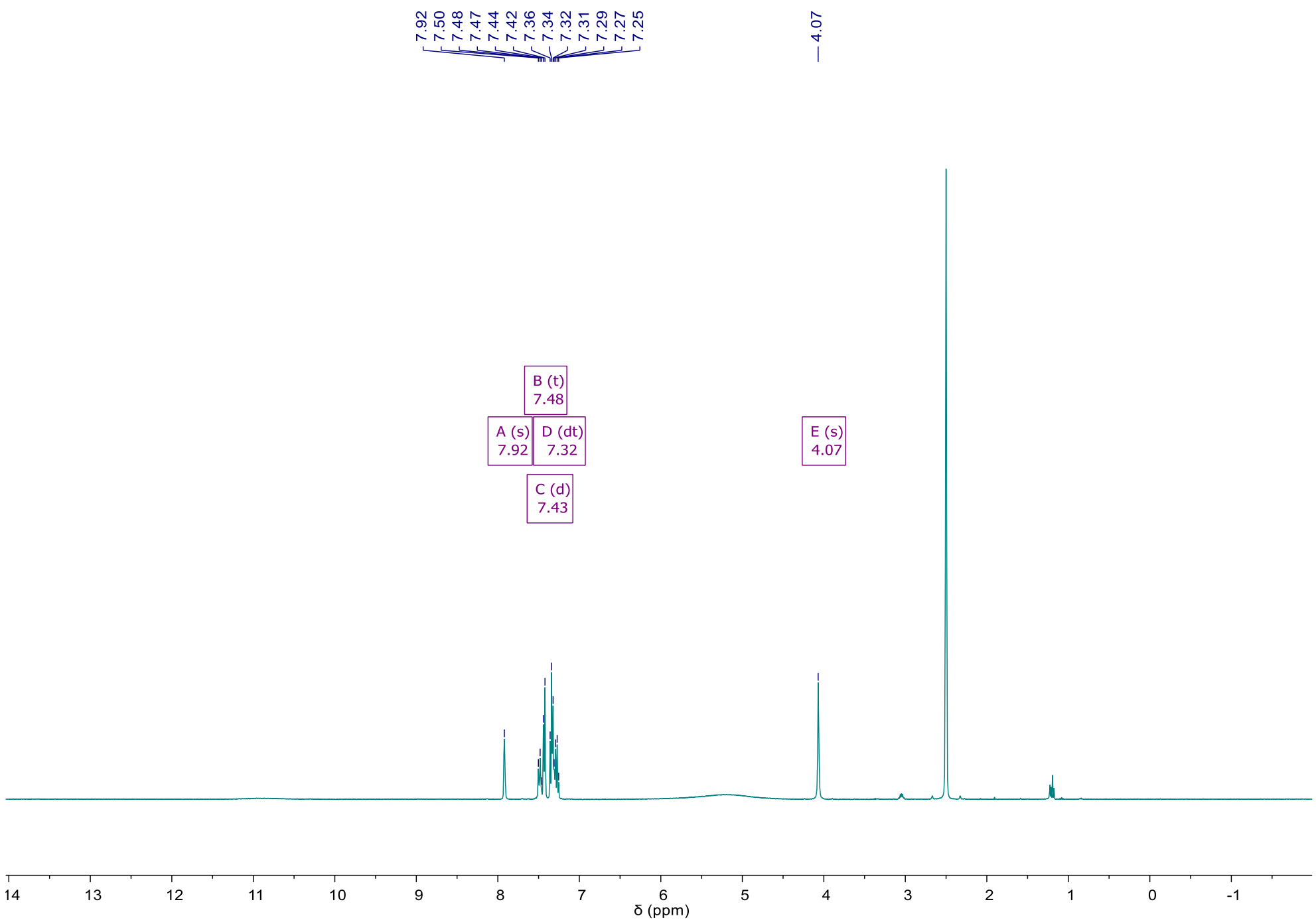

^{1}H-NMR of final compound **23** (DMSO-d_6).

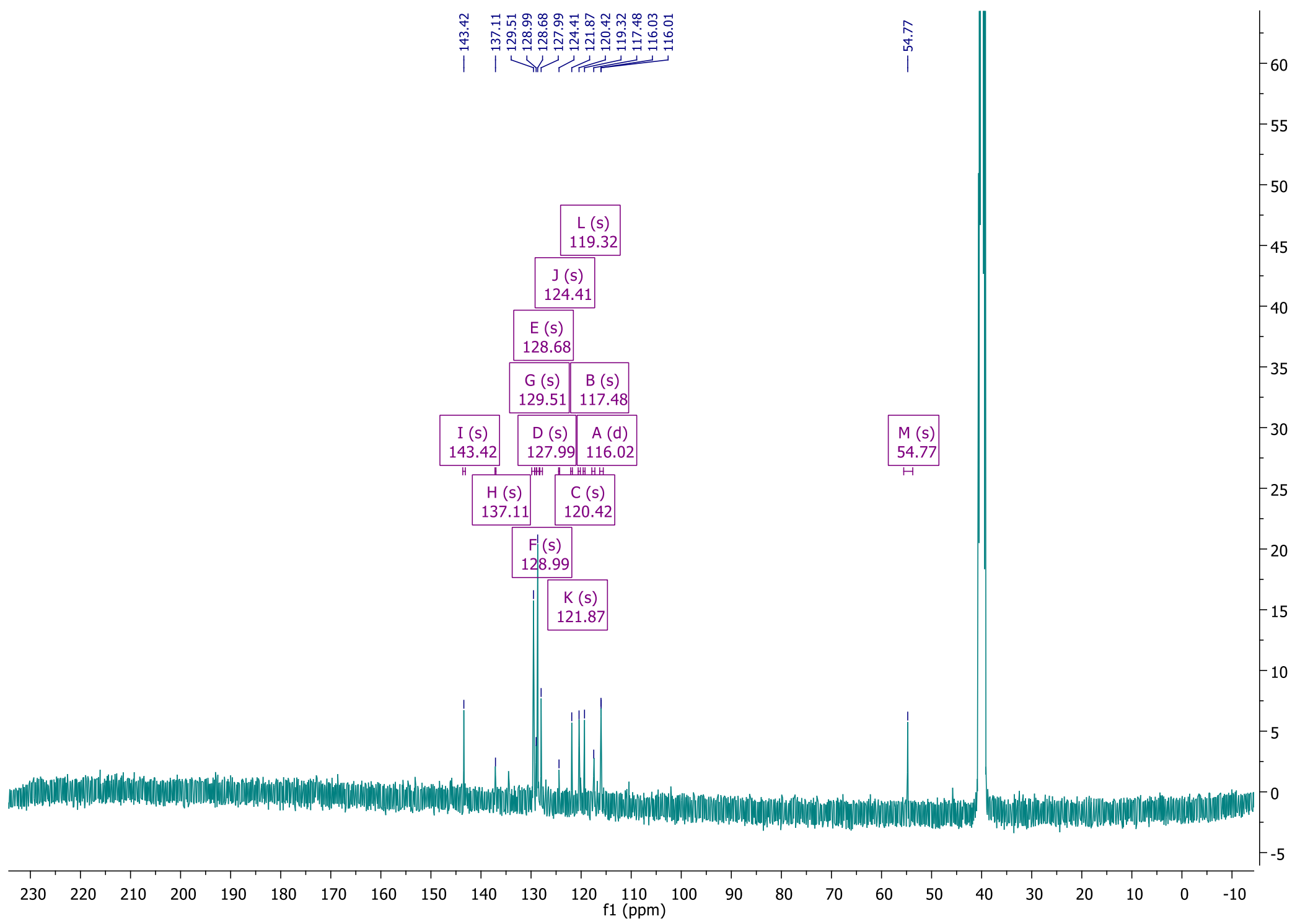

^{13}C-NMR of final compound **23** (DMSO-d_6).

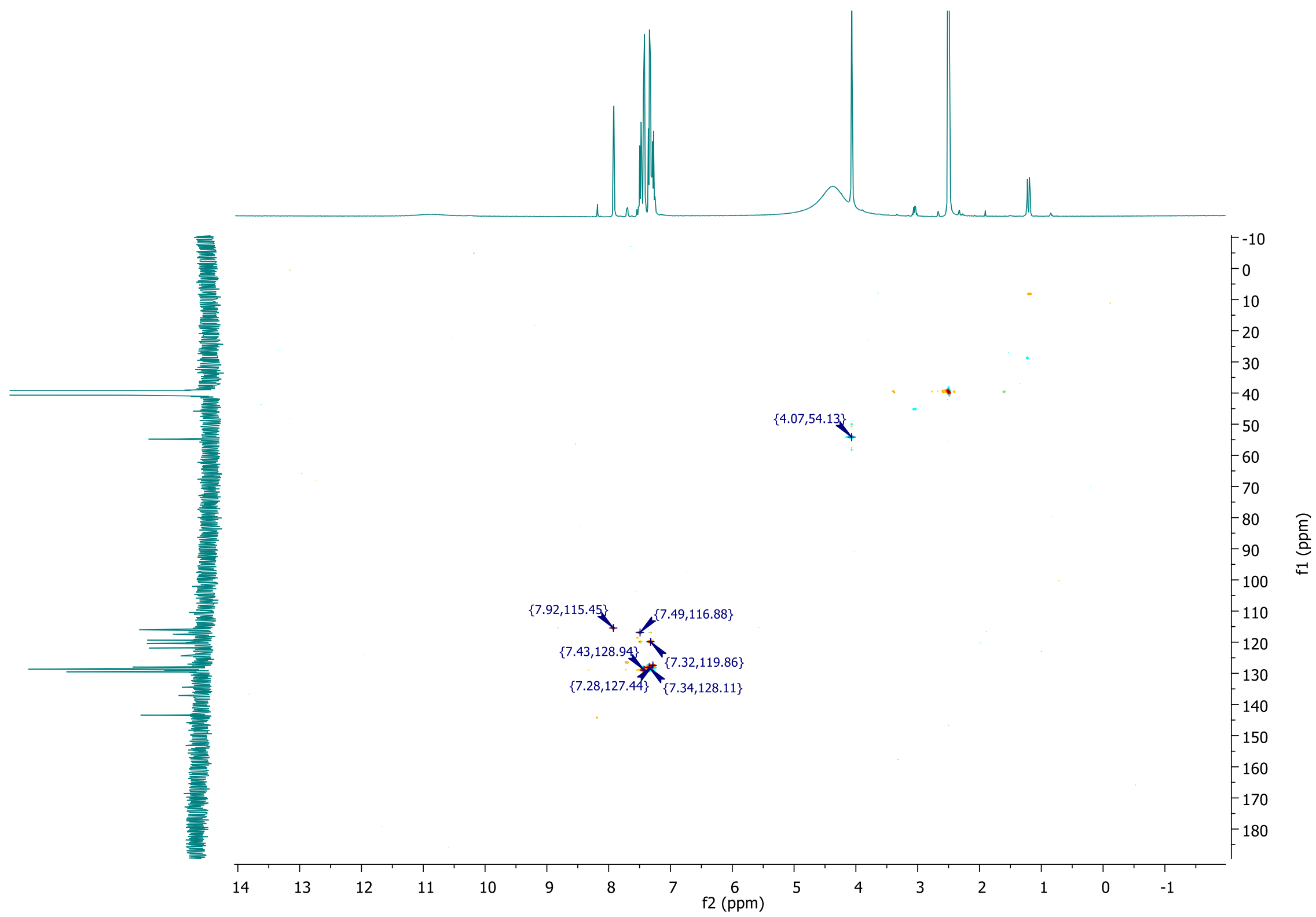

gHSQCAD of final compound **23** (DMSO-d_6).

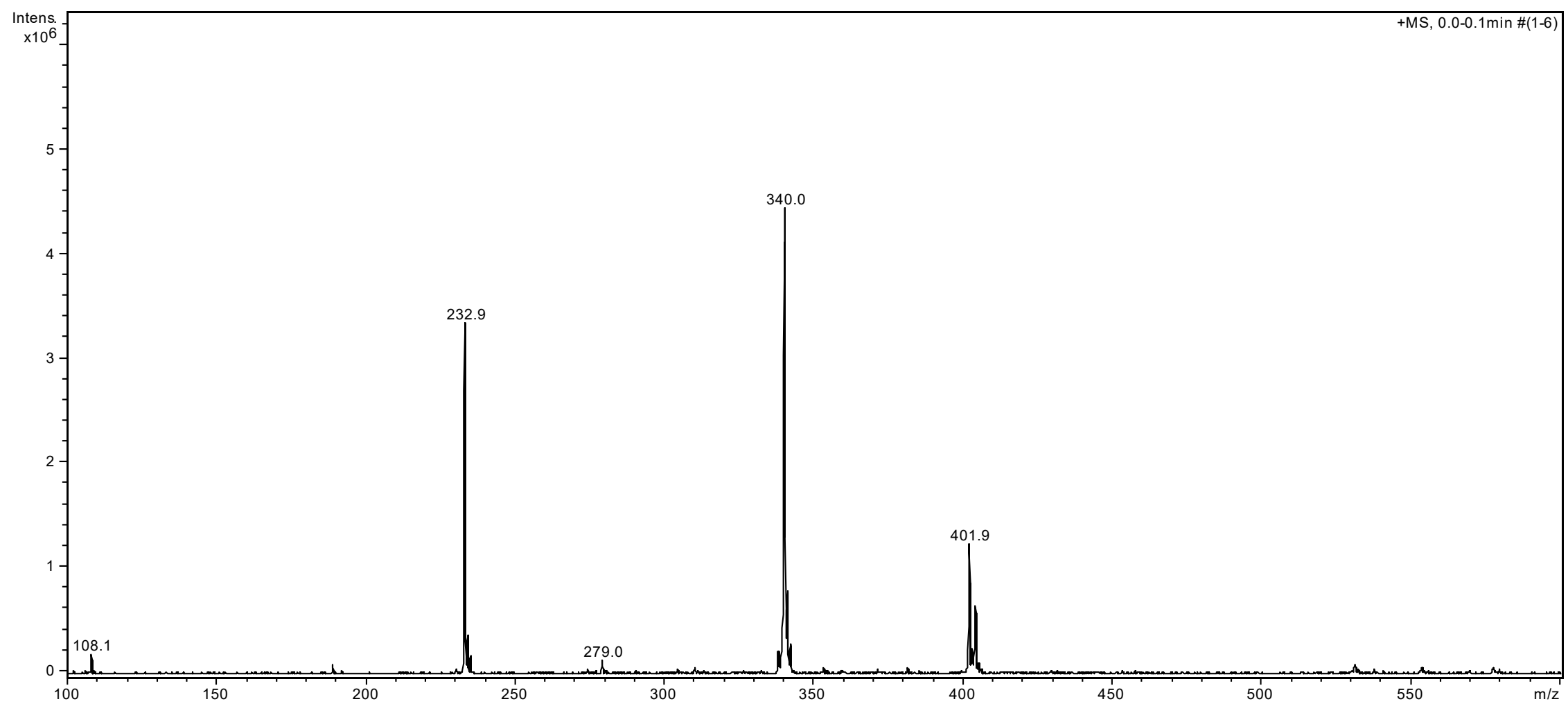

ES-MS of final compound **23** (MeOH)

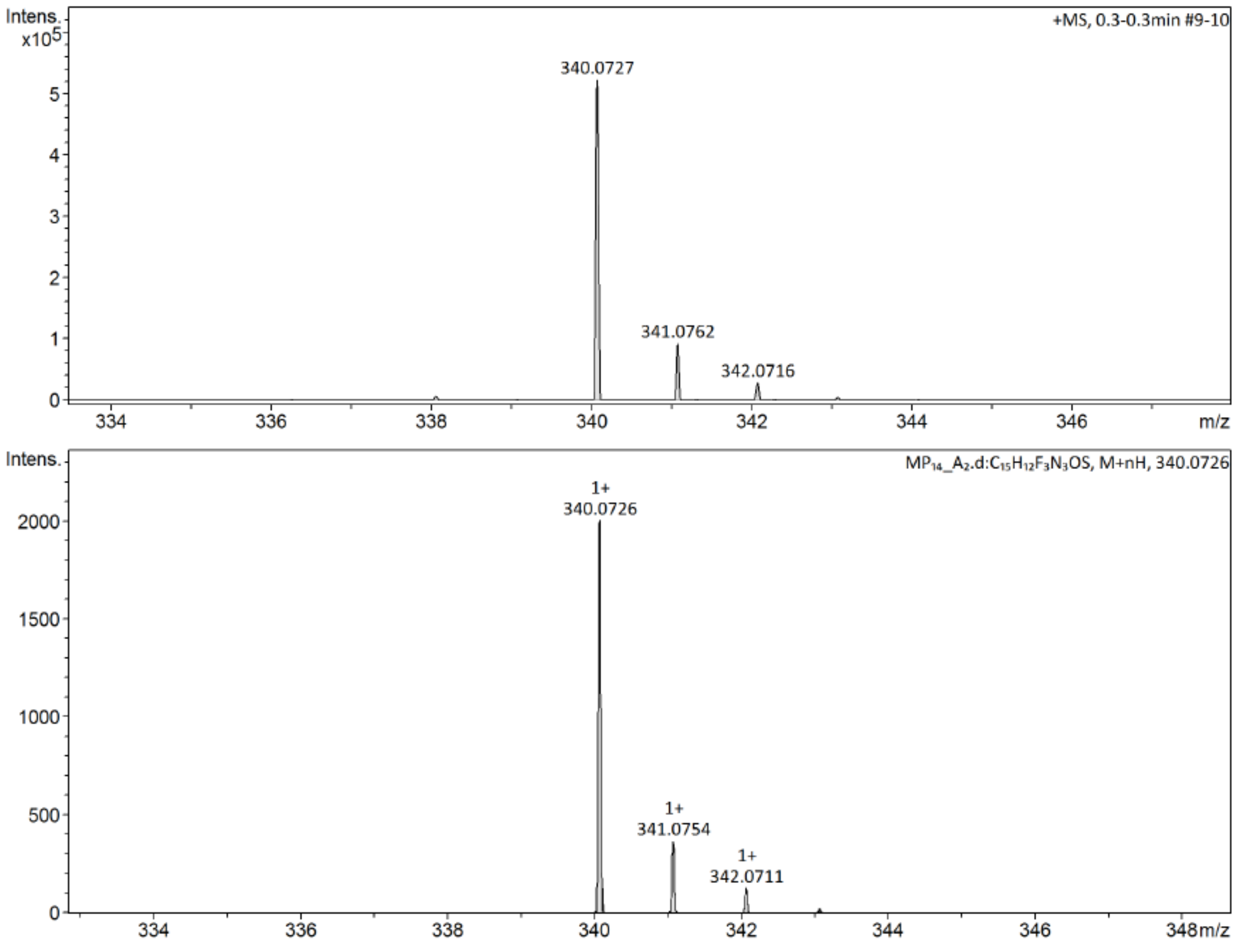

HRMS of final compound **23** (MeOH).

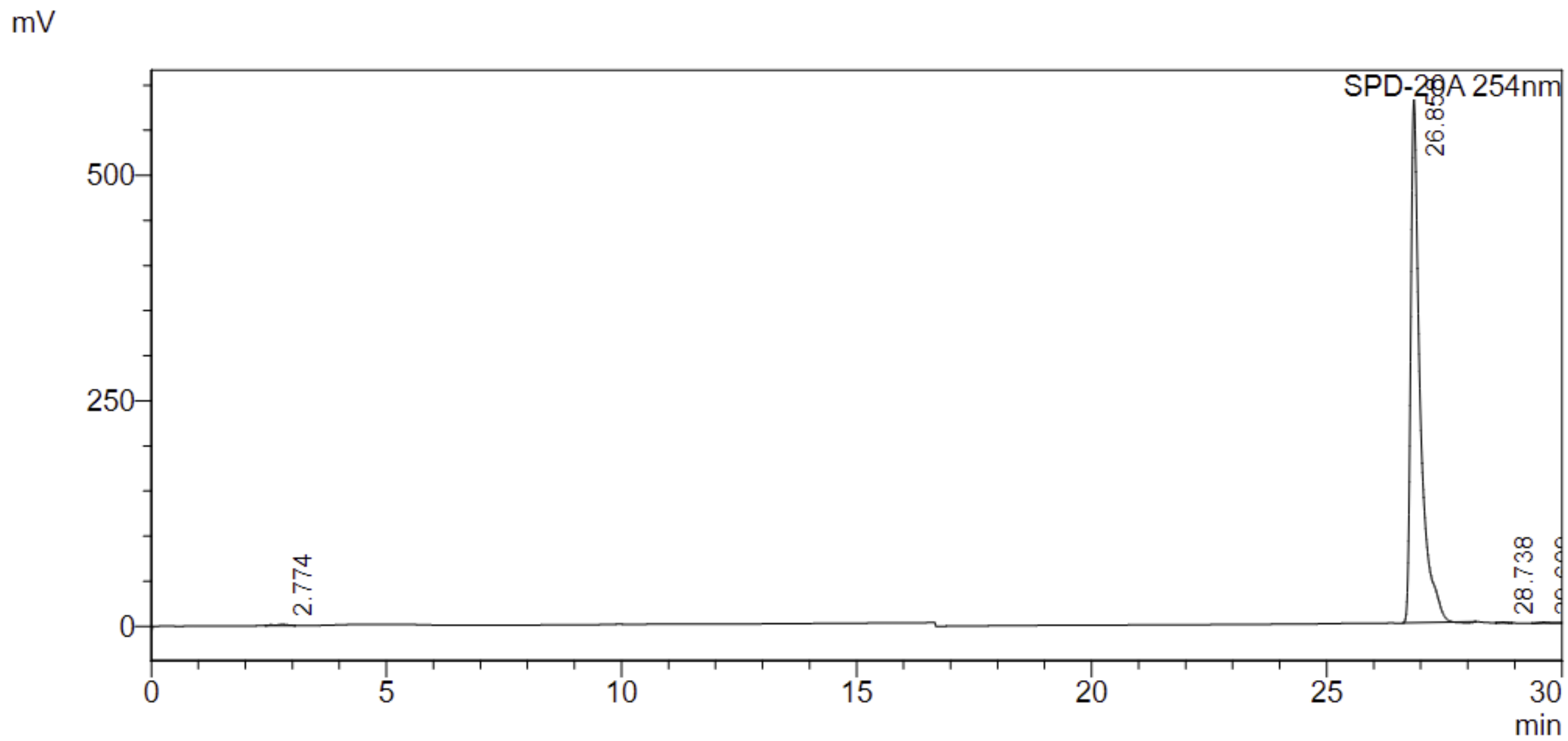

<Peak Table>

SPD-20A 254nm

Peak#	Ret. Time	Area	Height	Conc.	Unit	Mark	Name
1	2.774	21871	1211	0.266		M	
2	26.858	8189177	579734	99.469		M	
3	28.738	7636	718	0.093		M	
4	29.609	14239	1027	0.173		M	
Total		8232923	582690				

HPLC of final compound **23** (flow: 1 ml/min, eleunt gradient: from water-methanol 55:45 to 15:85 in 30 min; detector: 254 nm).

2-(2-phenethylhydrazineyl)-6-(trifluoromethoxy)benzo[d]thiazole (**24**)

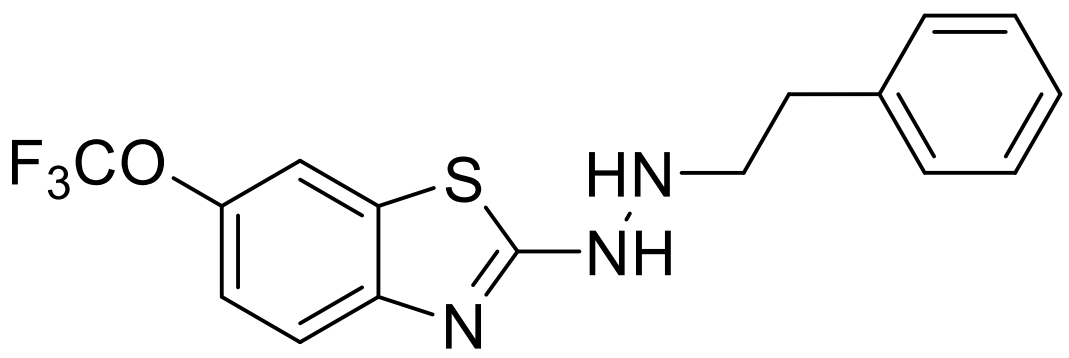

Chemical Formula: $C_{16}H_{14}F_3N_3OS$
Exact Mass: 353.08
Molecular Weight: 353.36

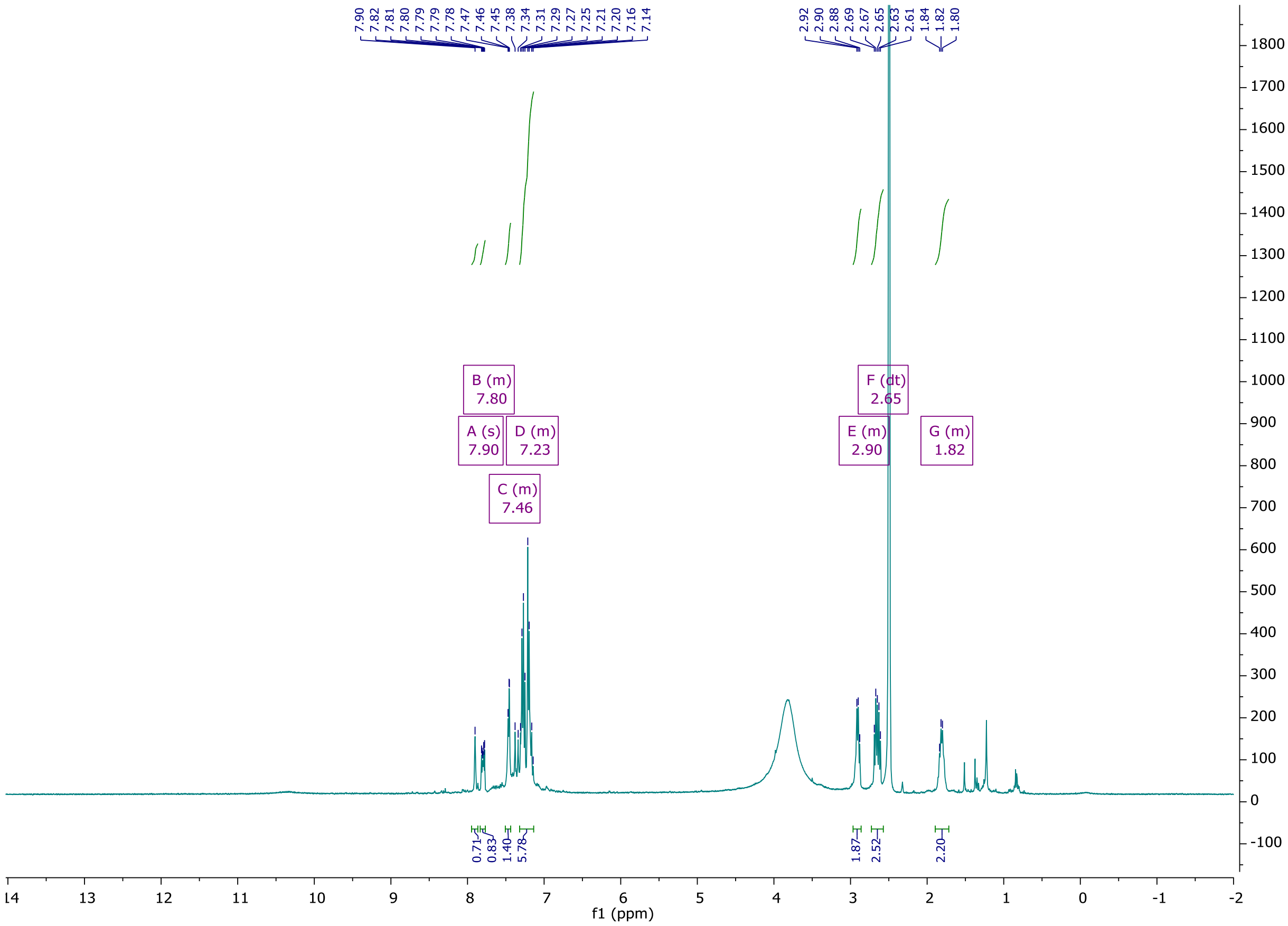

^{1}H-NMR of final compound **24** (DMSO-d_6).

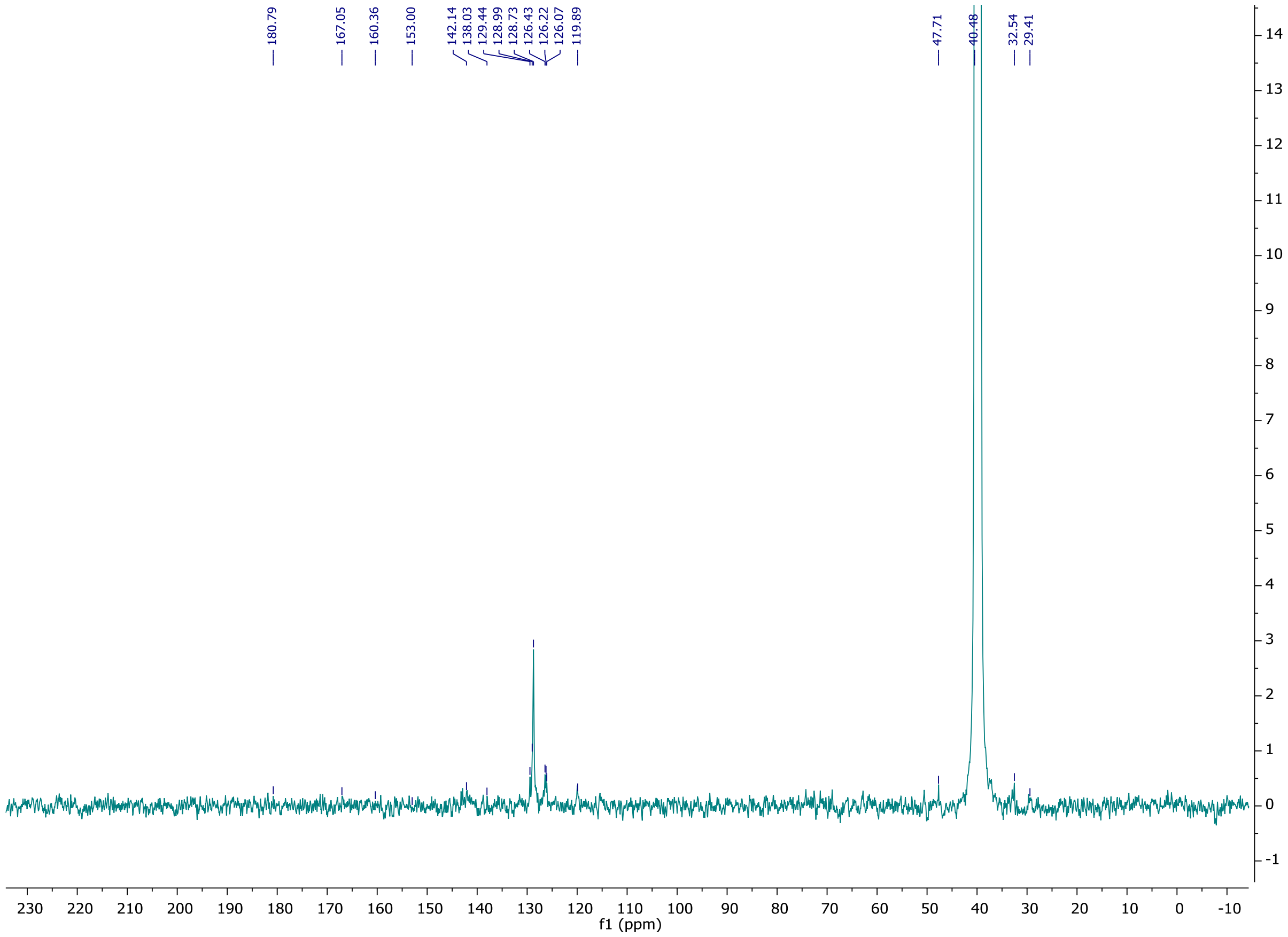

^{13}C-NMR of final compound **24** (DMSO-d_6).

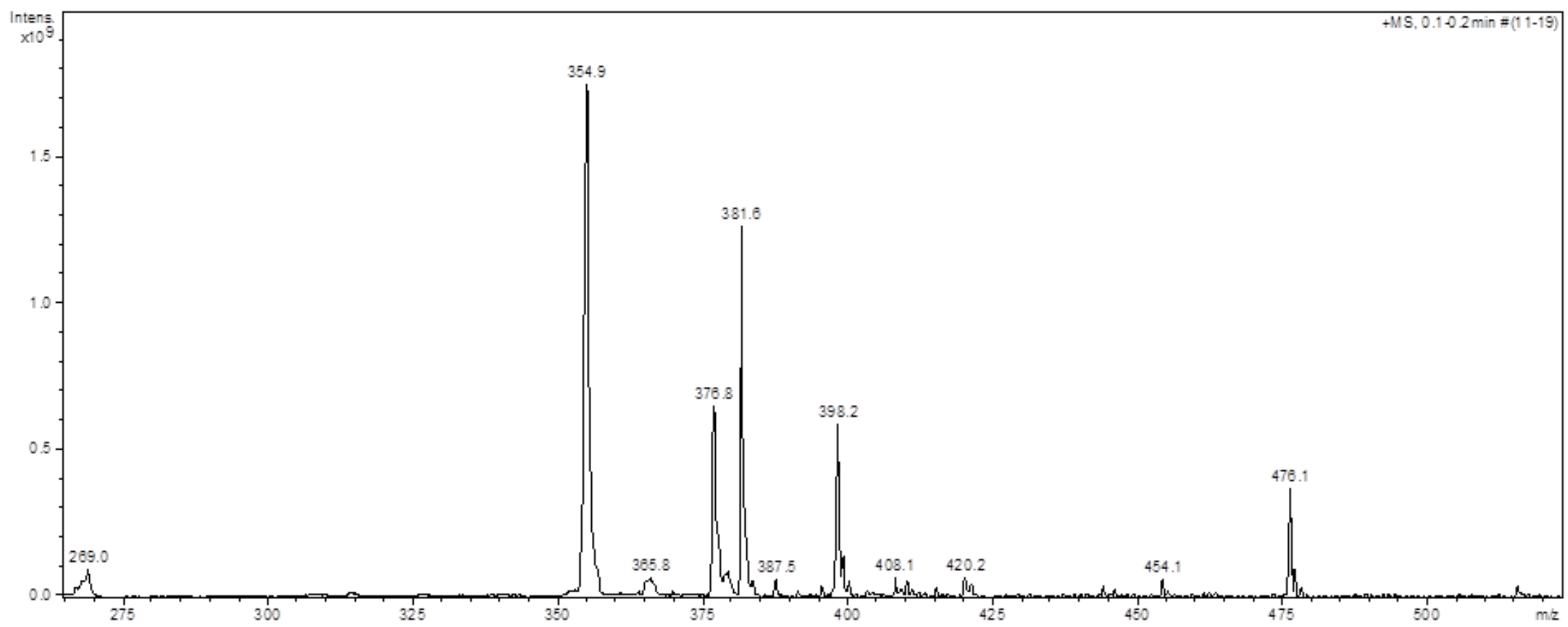

ES-MS of final compound **24** (MeOH).

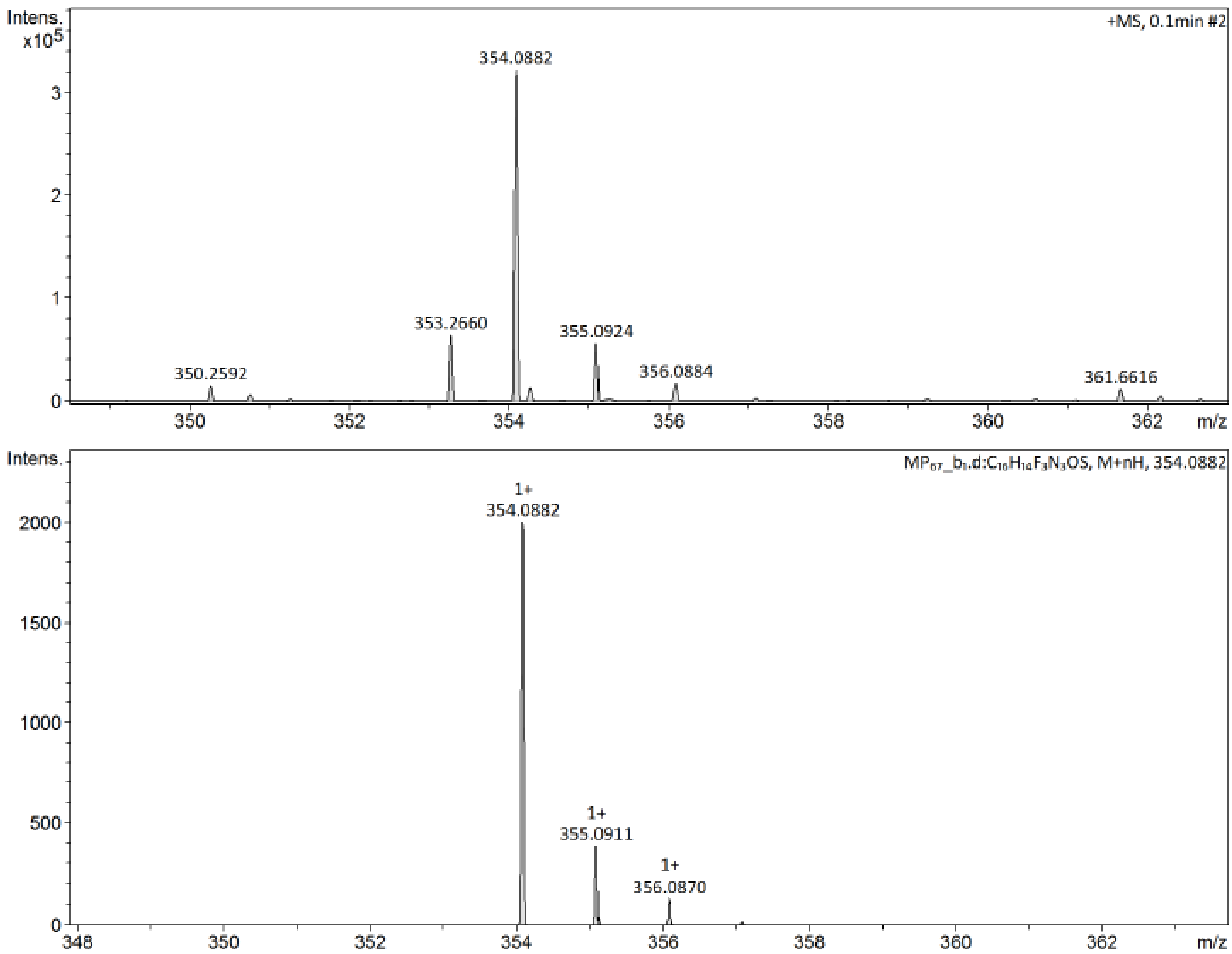

HRMS of final compound **24** (MeOH).

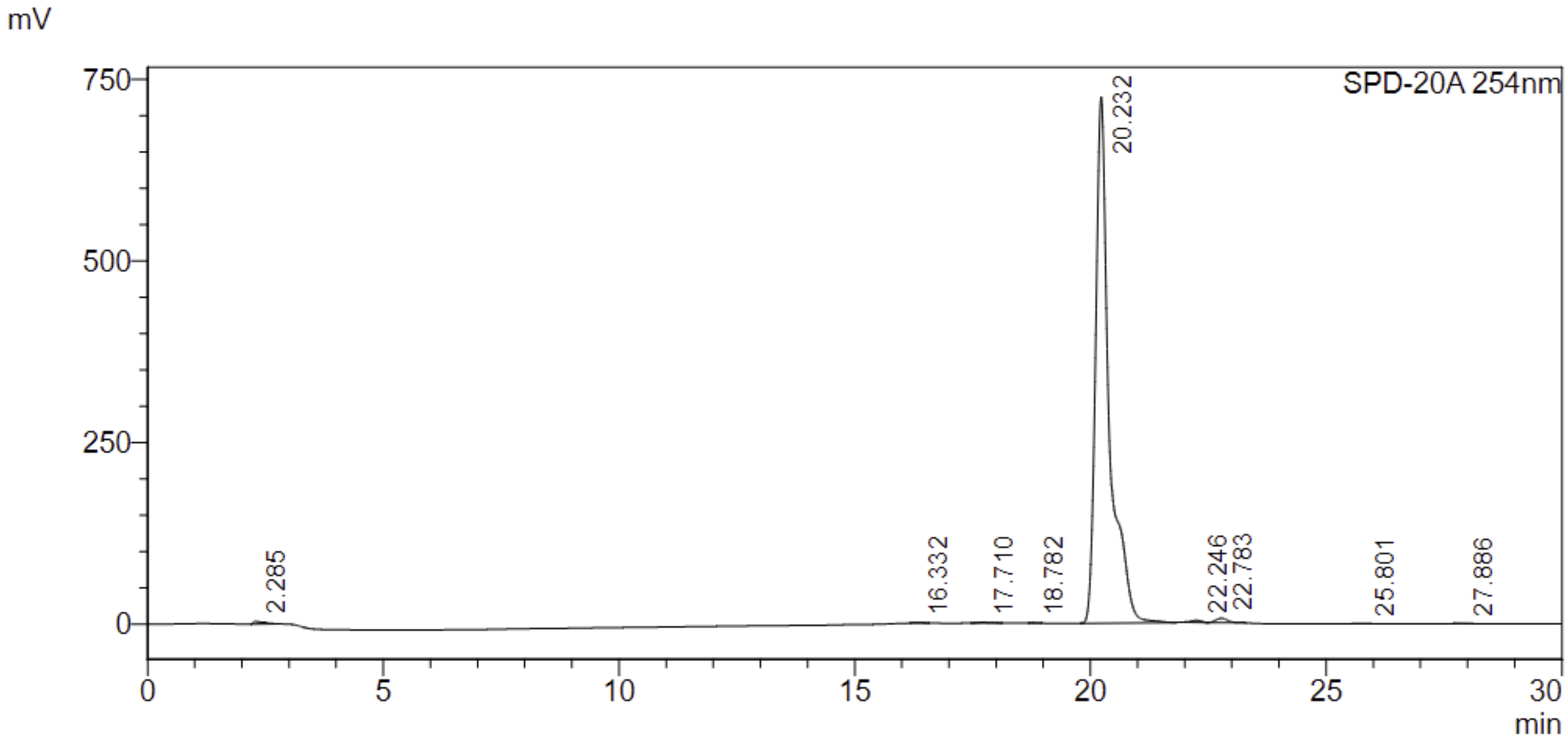

<Peak Table>

SPD-20A 254nm

Peak#	Ret. Time	Area	Height	Conc.	Unit	Mark	Name
1	2.285	61055	3871	0.409		M	
2	16.332	4946	372	0.033		M	
3	17.710	16181	871	0.108		M	
4	18.782	2420	278	0.016		M	
5	20.232	14697934	724382	98.415		M	
6	22.246	38210	2880	0.256		M	
7	22.783	102725	5975	0.688		M	
8	25.801	2824	128	0.019		M	
9	27.886	8347	529	0.056		M	
Total		14934642	739286				

HPLC of final compound **24** (flow: 1 ml/min, eleunt gradient: from water-methanol 55:45 to 15:85 in 30 min; detector: 254 nm).

2-(2-(3-phenylpropyl)hydrazineyl)-6-(trifluoromethoxy)benzo[d]thiazole (**25**)

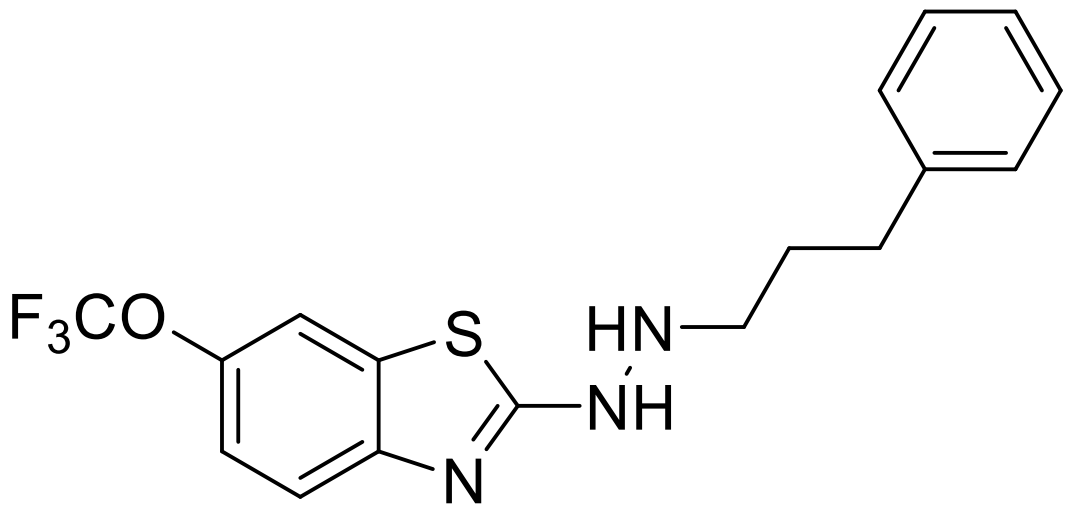

Chemical Formula: $C_{17}H_{16}F_3N_3OS$
Exact Mass: 367.10
Molecular Weight: 367.39

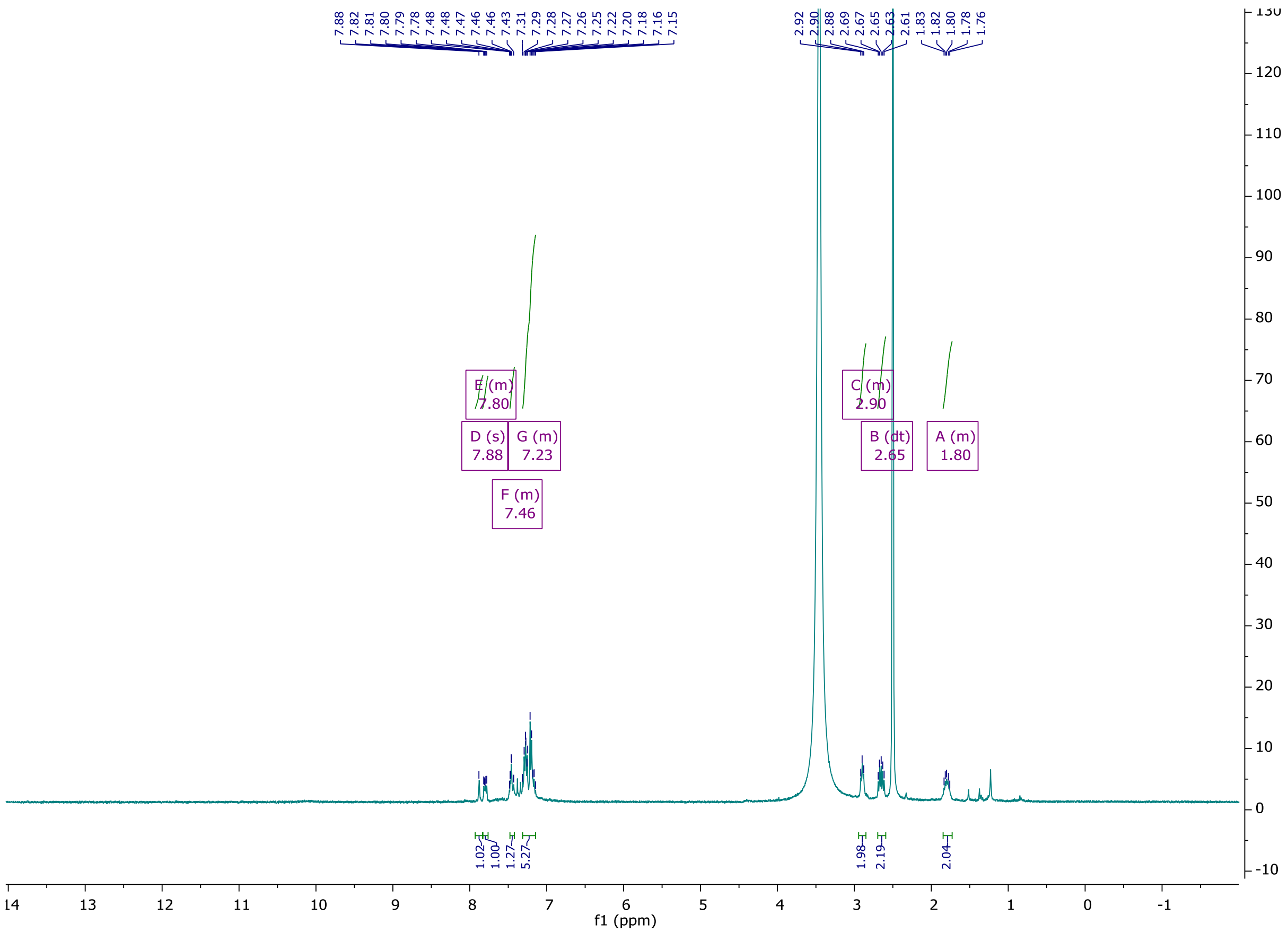

^{1}H-NMR of final compound **25** (DMSO-d_6).

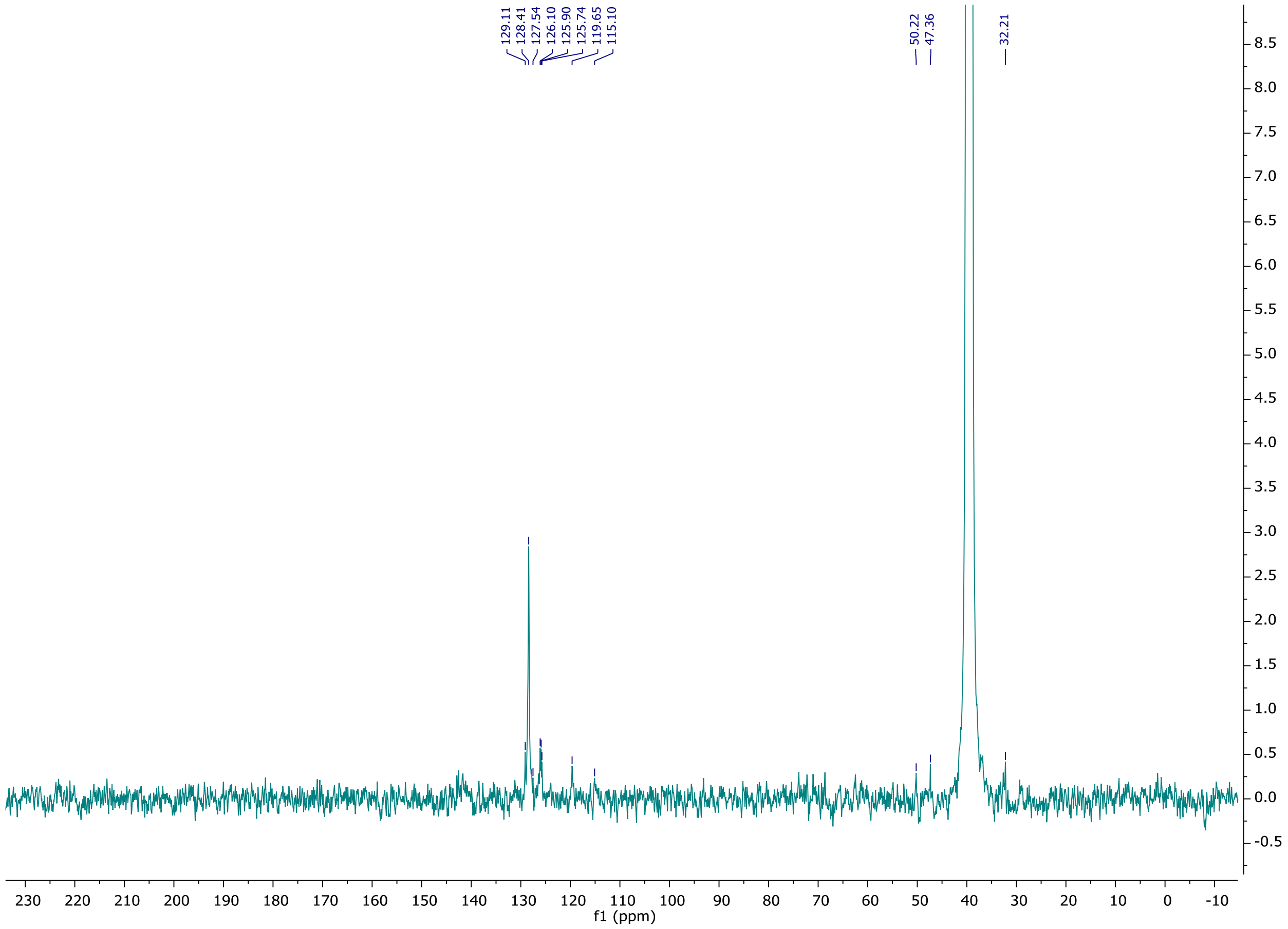

^{13}C-NMR of final compound **25** (DMSO-d_6).

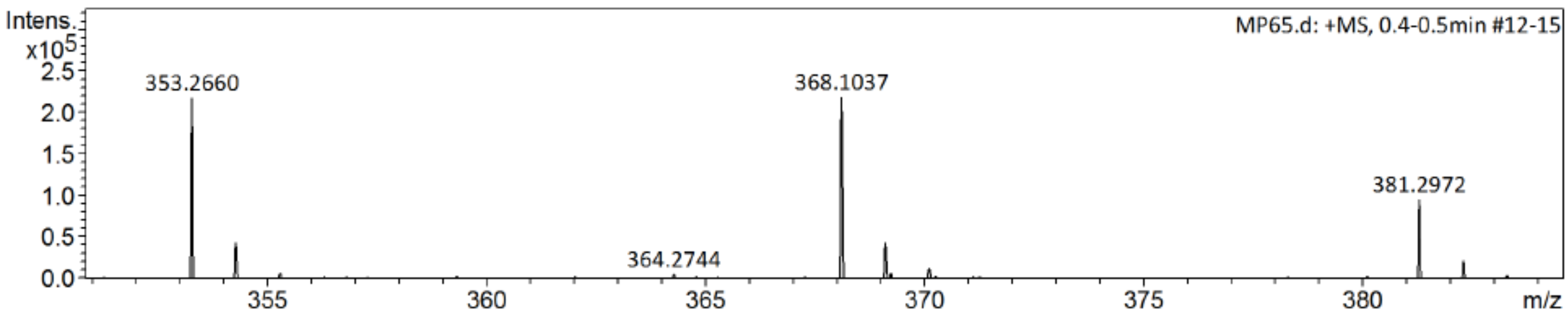

ES-MS of final compound **25** (MeOH).

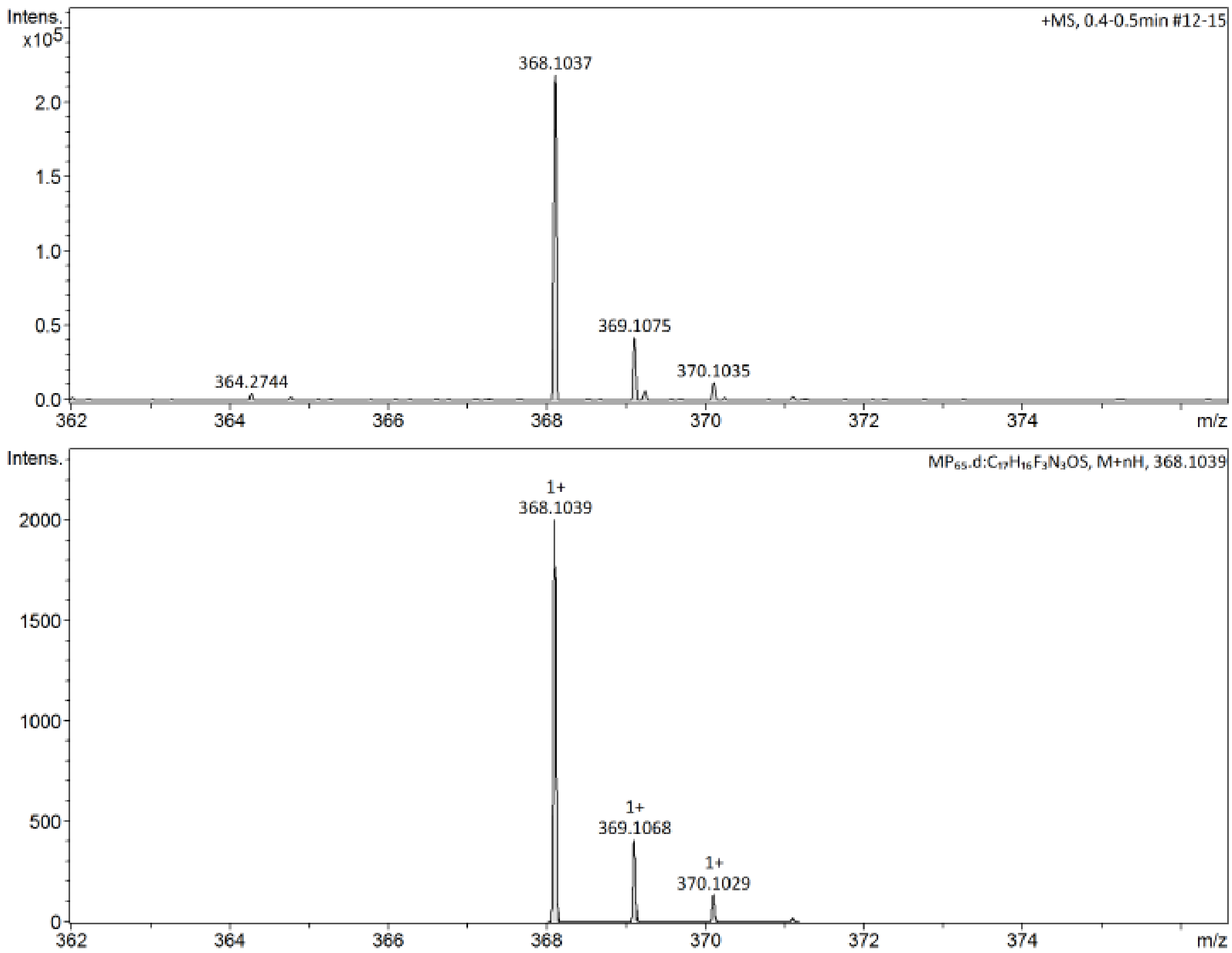

HRMS of final compound **25** (MeOH).

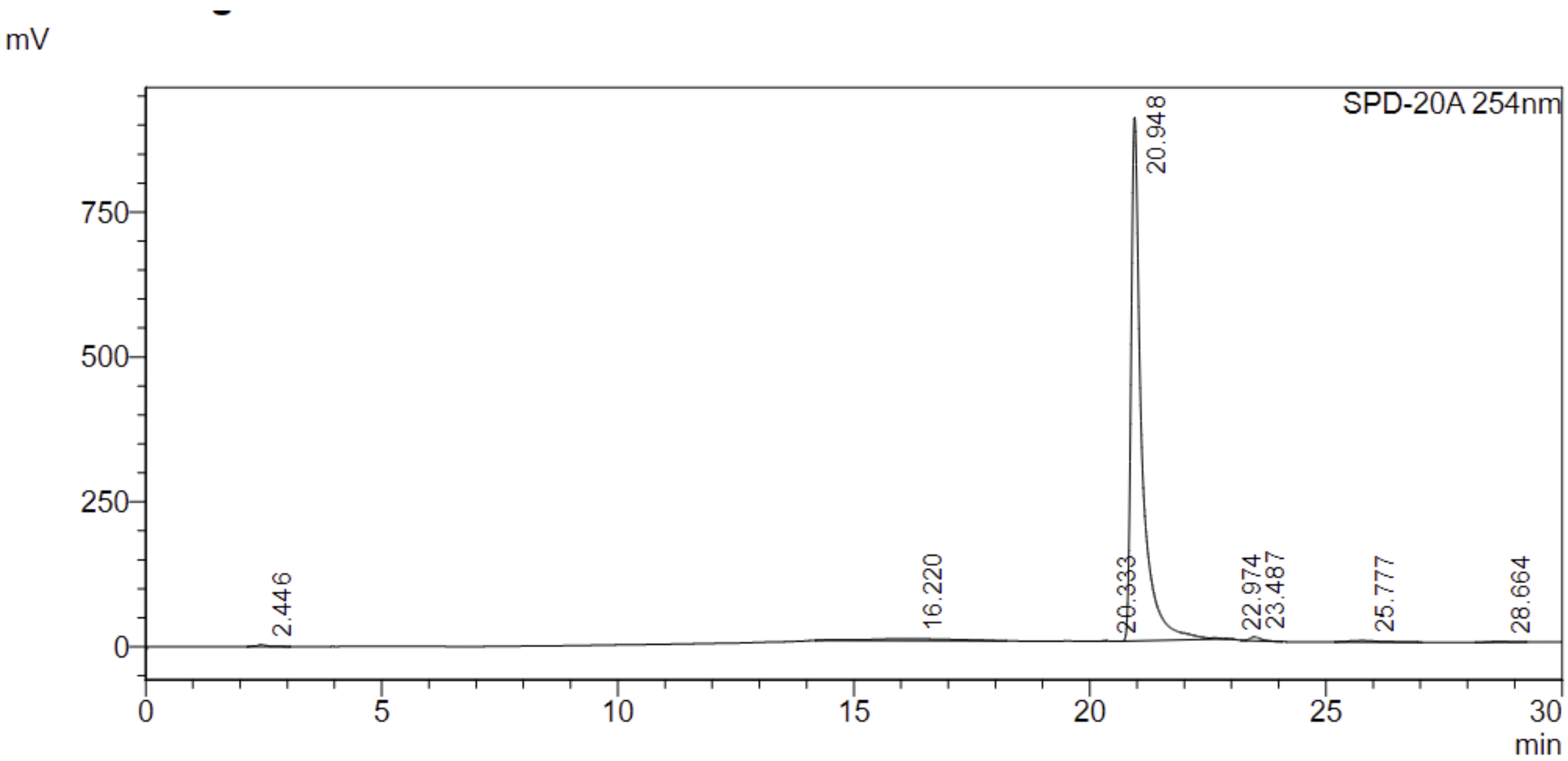

<Peak Table>

SPD-20A 254nm

Peak#	Ret. Time	Area	Height	Conc.	Unit	Mark	Name
1	2.446	55482	3658	0.369		M	
2	16.220	423938	3087	2.819		M	
3	20.333	568	67	0.004		M	
4	20.948	14316059	903679	95.207		M	
5	22.974	5272	715	0.035		M	
6	23.487	106145	6774	0.706		M	
7	25.777	107004	2383	0.712		M	
8	28.664	22292	947	0.148		M	
Total		15036758	921309				

HPLC of final compound **25** (flow: 1 ml/min, eluent gradient: from water-methanol 55:45 to 15:85 in 30 min; detector: 254 nm).

tert-butyl (benzyloxy)(6-(trifluoromethoxy)benzo[d]thiazol-2-yl)carbamate (**48**)

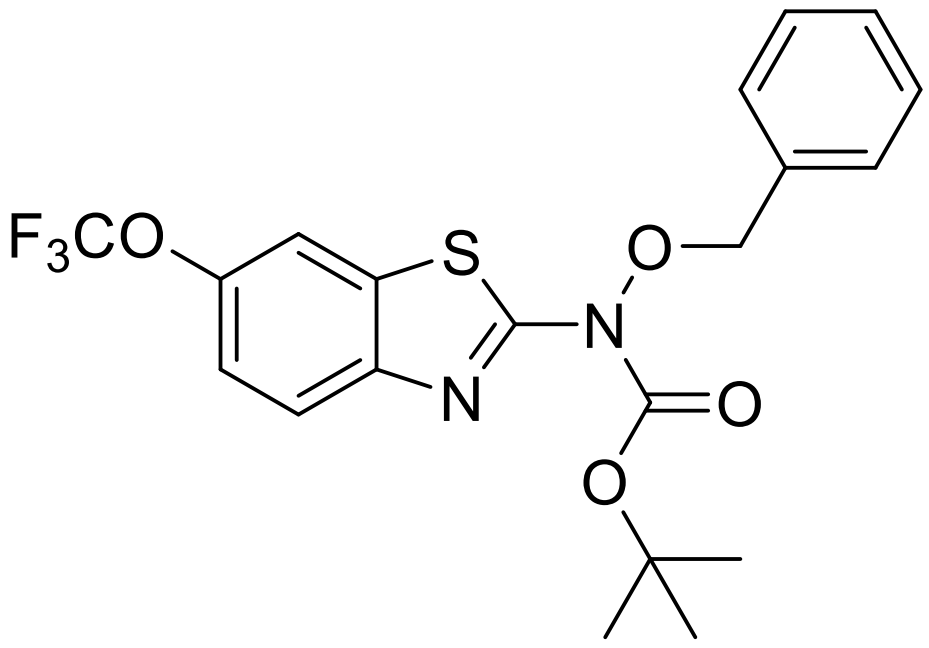

Chemical Formula: $C_{20}H_{19}F_3N_2O_4S$
Exact Mass: 440.10
Molecular Weight: 440.44

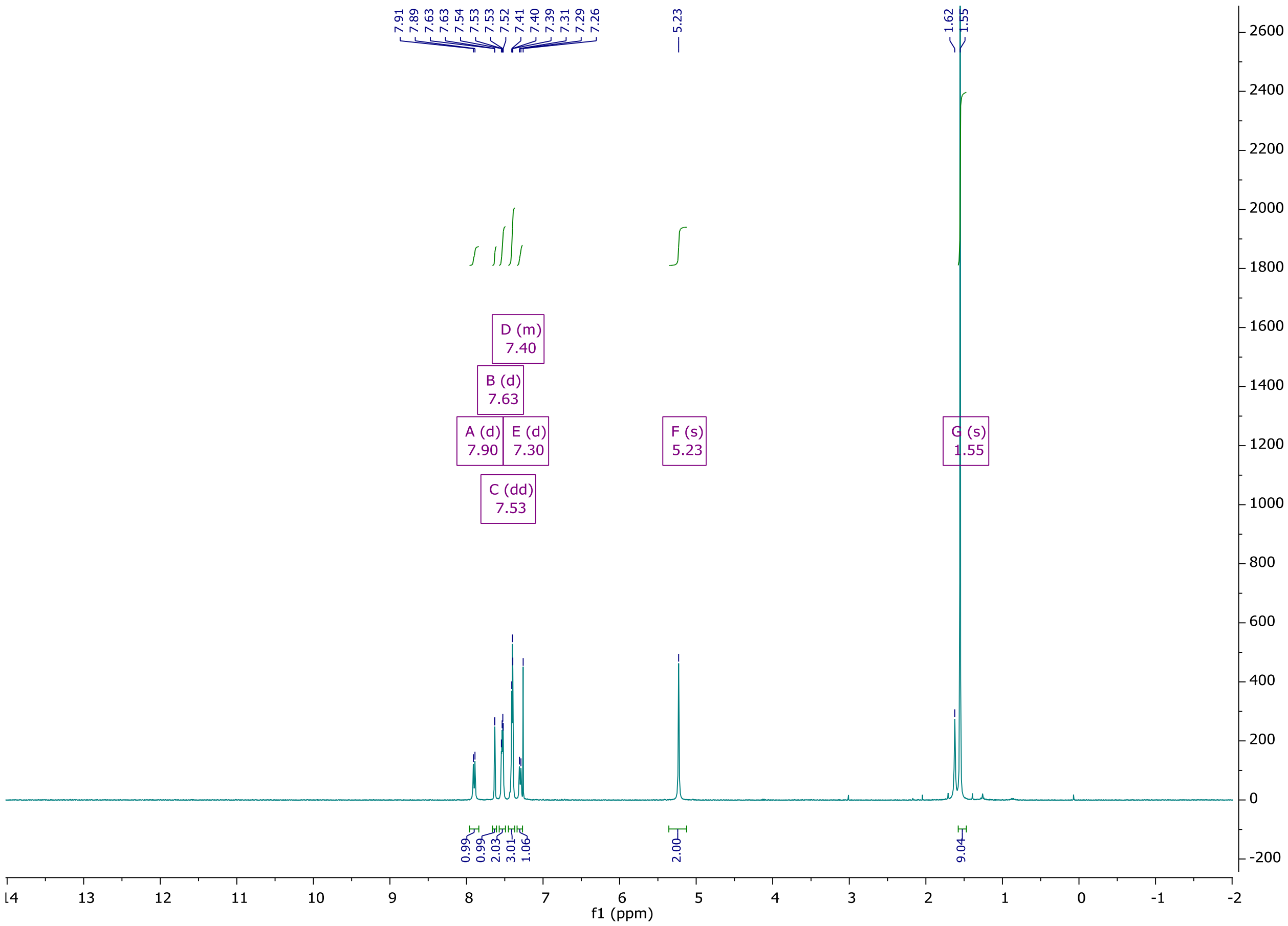

^{1}H-NMR of final compound **48** ($CDCl_3$).

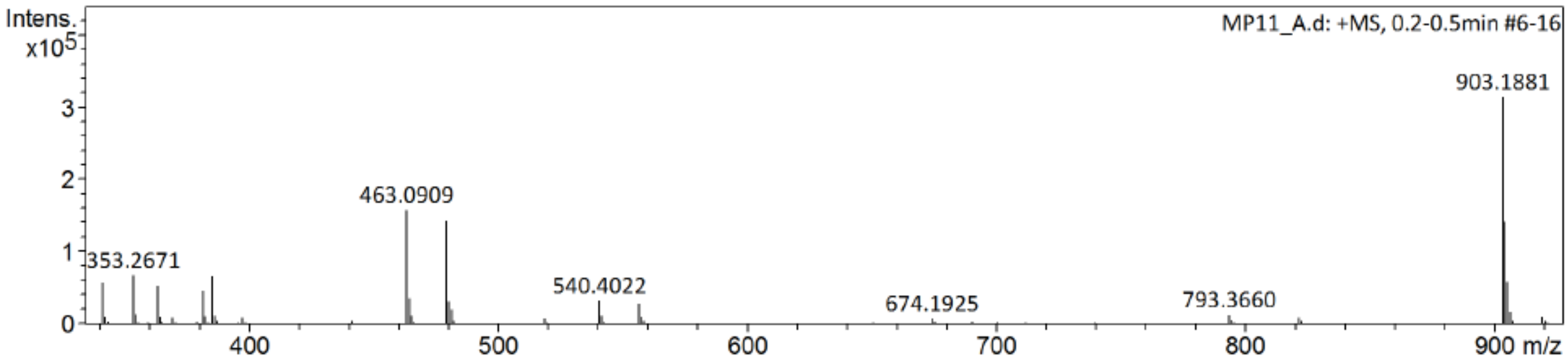

ES-MS of final compound **48** (MeOH).

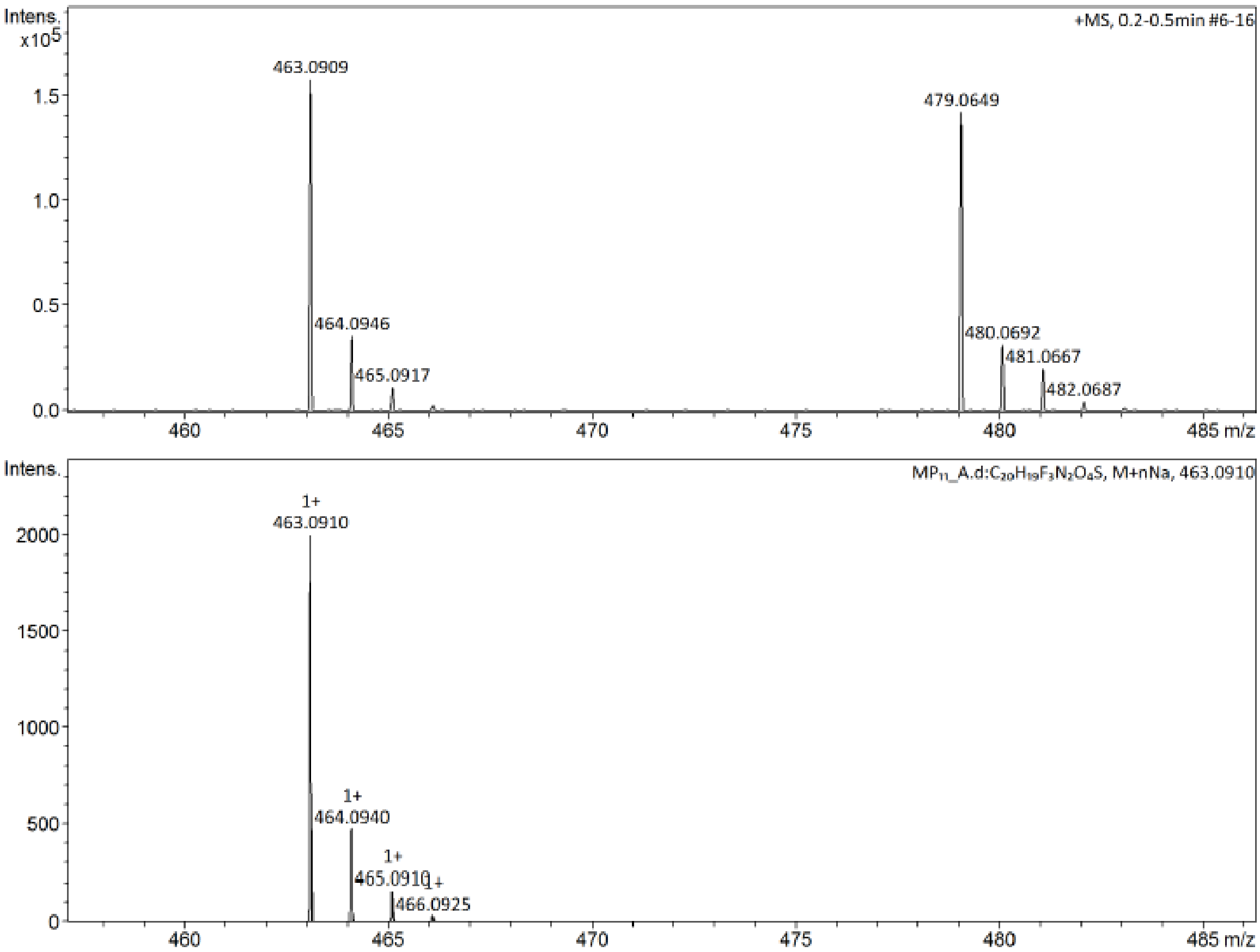

HRMS of final compound **48** (MeOH).

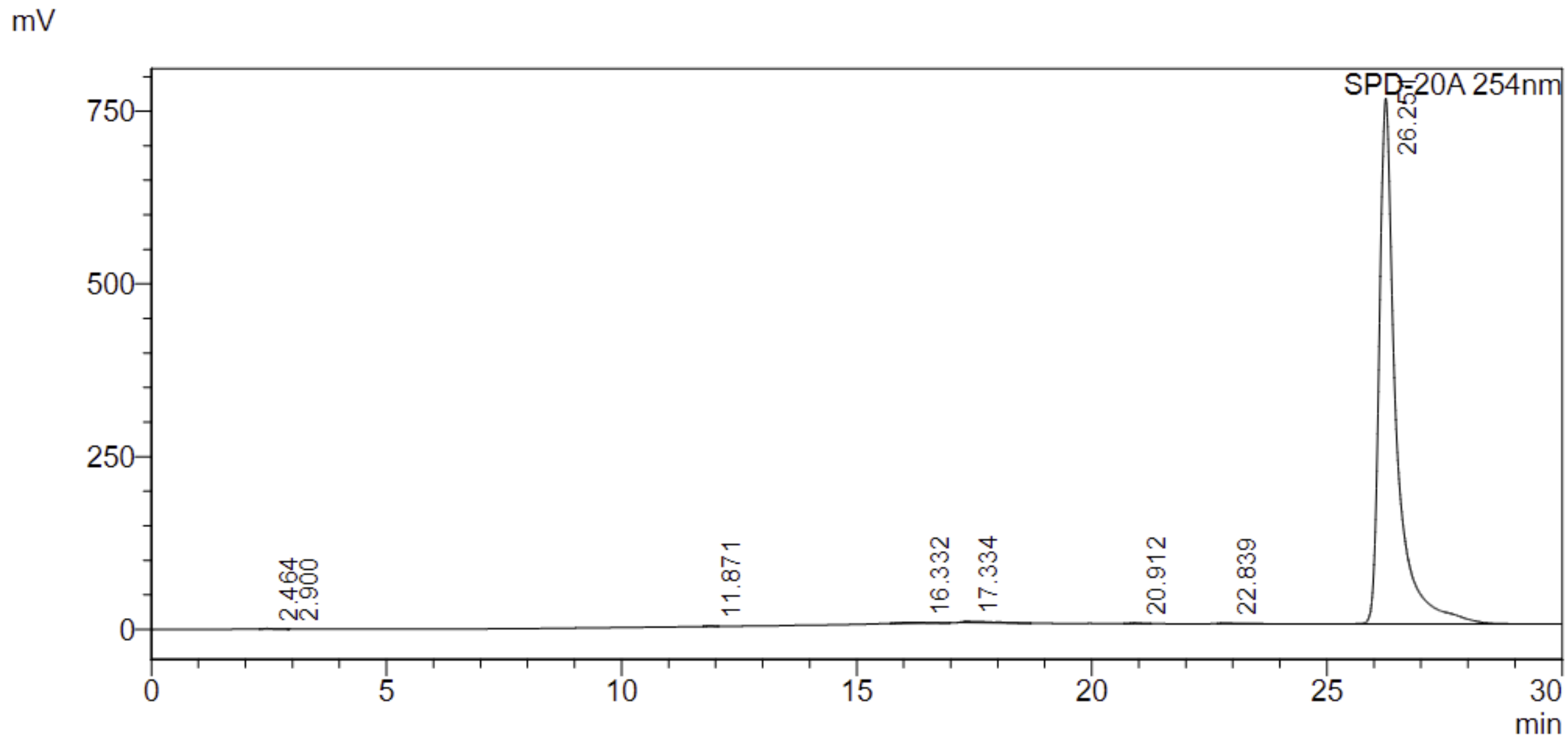

<Peak Table>

SPD-20A 254nm

Peak#	Ret. Time	Area	Height	Conc.	Unit	Mark	Name
1	2.464	12793	1011	0.067		S M	
2	2.900	-0	2	-0.000		T M	
3	11.871	6434	684	0.034		M	
4	16.332	54871	1198	0.286		M	
5	17.334	84226	2199	0.439		M	
6	20.912	11271	968	0.059		M	
7	22.839	17661	595	0.092		M	
8	26.251	19004790	760337	99.024		M	
Total		19192046	766993				

HPLC of final compound **48** (flow: 1 ml/min, eleunt gradient: from water-methanol 55:45 to 15:85 in 30 min; detector: 254 nm).

tert-butyl ((2-ethylhexyl)oxy)(6-(trifluoromethoxy)benzo[d]thiazol-2-yl)carbamate (**49**)

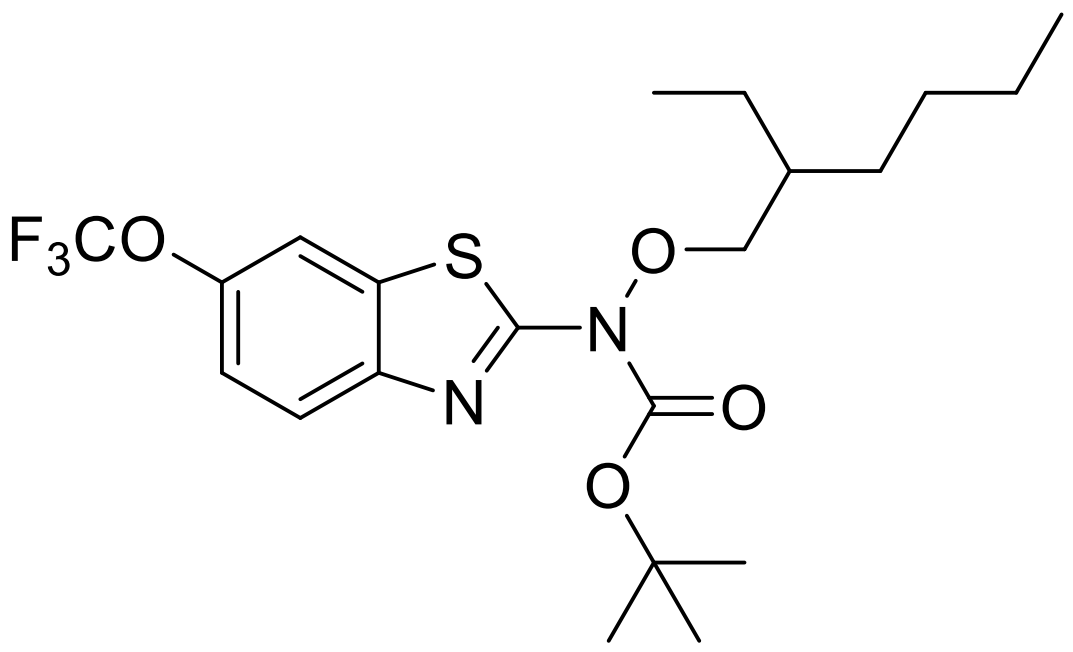

Chemical Formula: $C_{21}H_{29}F_3N_2O_4S$
Exact Mass: 462.18
Molecular Weight: 462.53

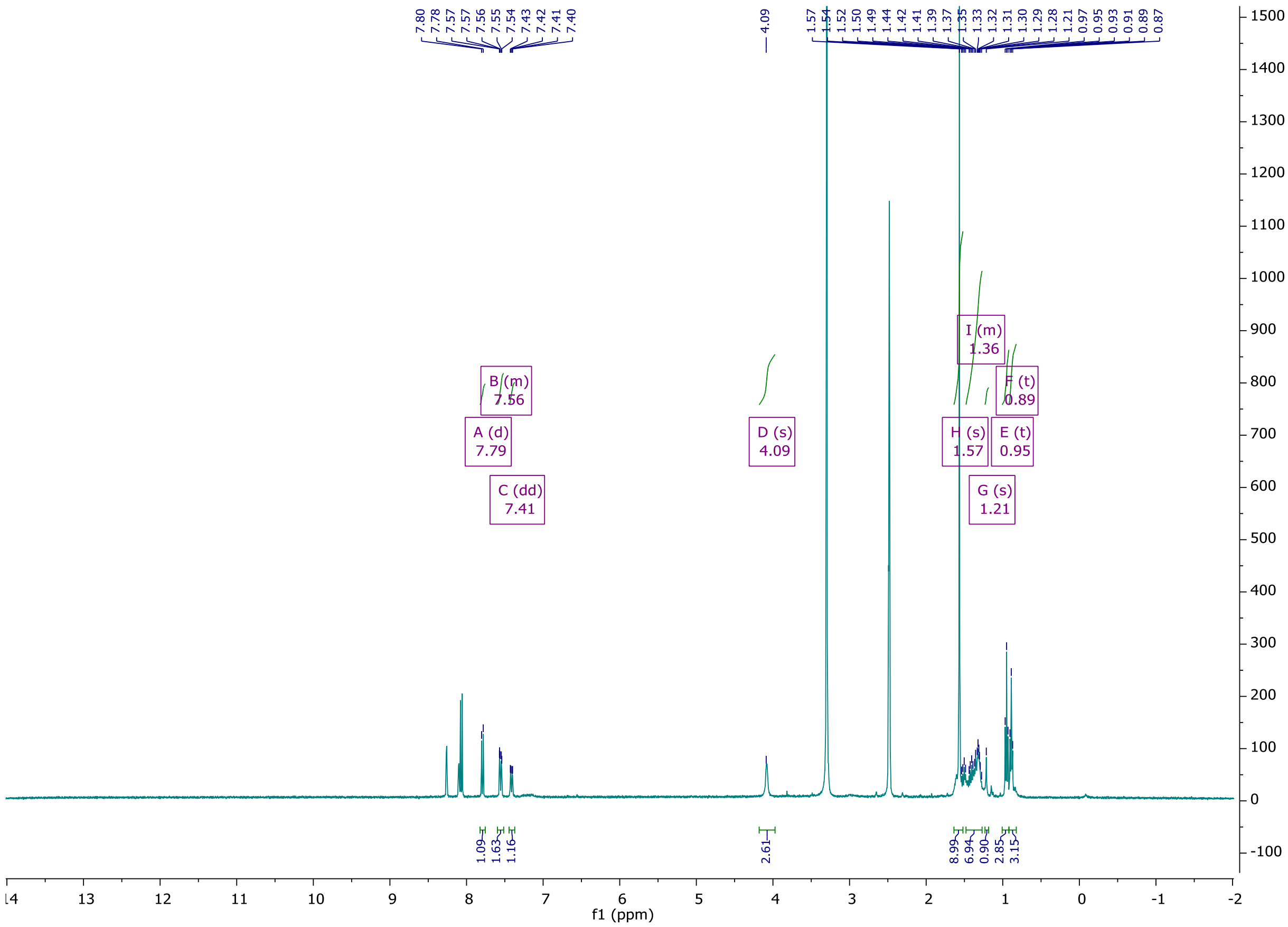

^{1}H-NMR of final compound **49** (DMSO-d_6).

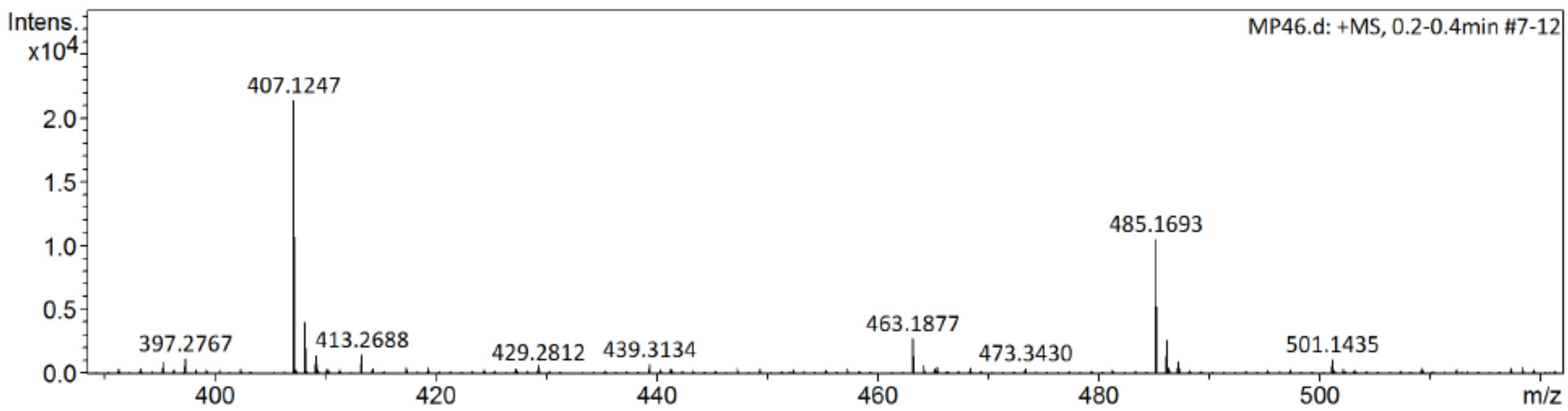

ES-MS of final compound **49** (MeOH).

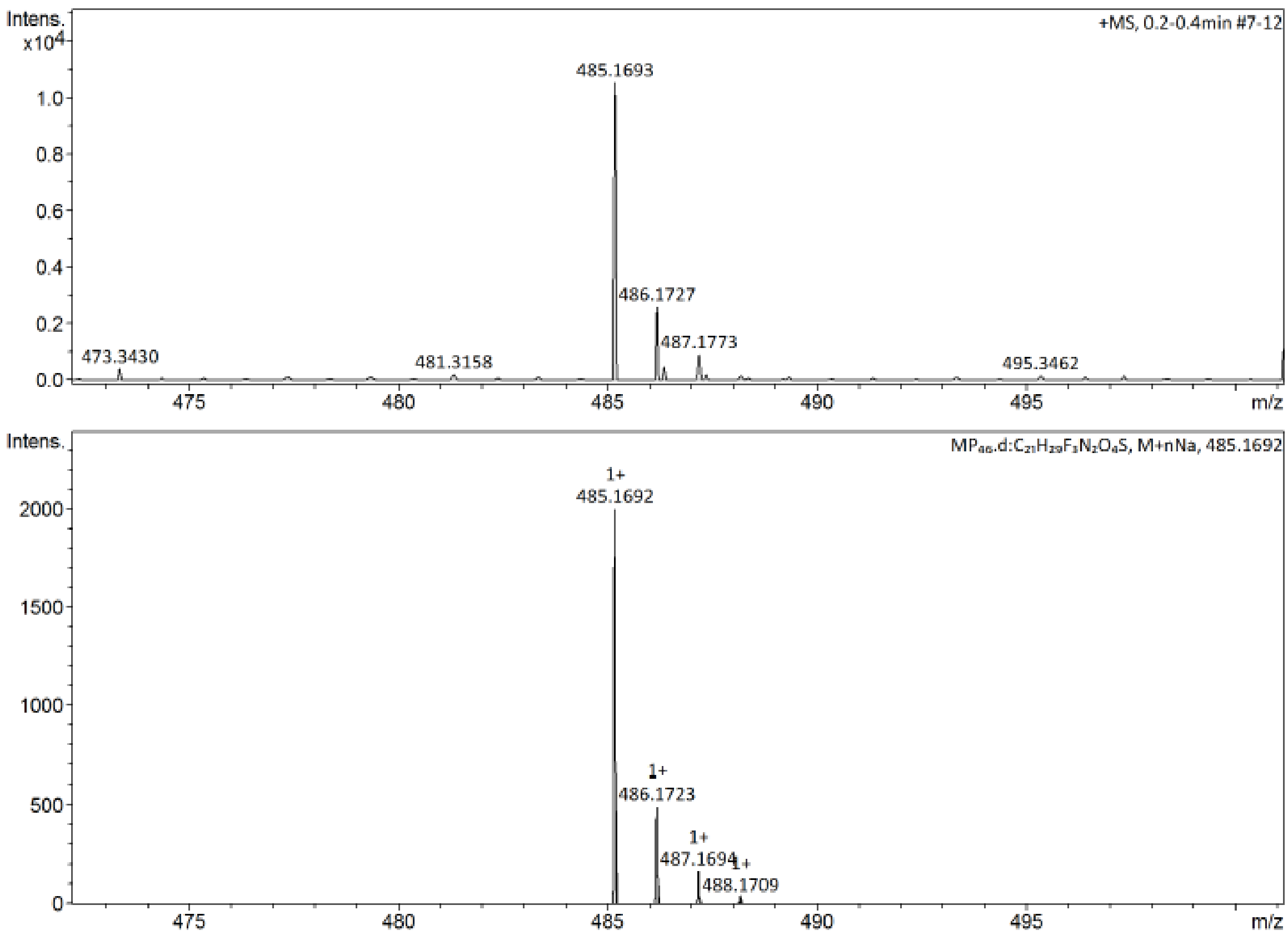

HRMS of final compound **49** (MeOH).

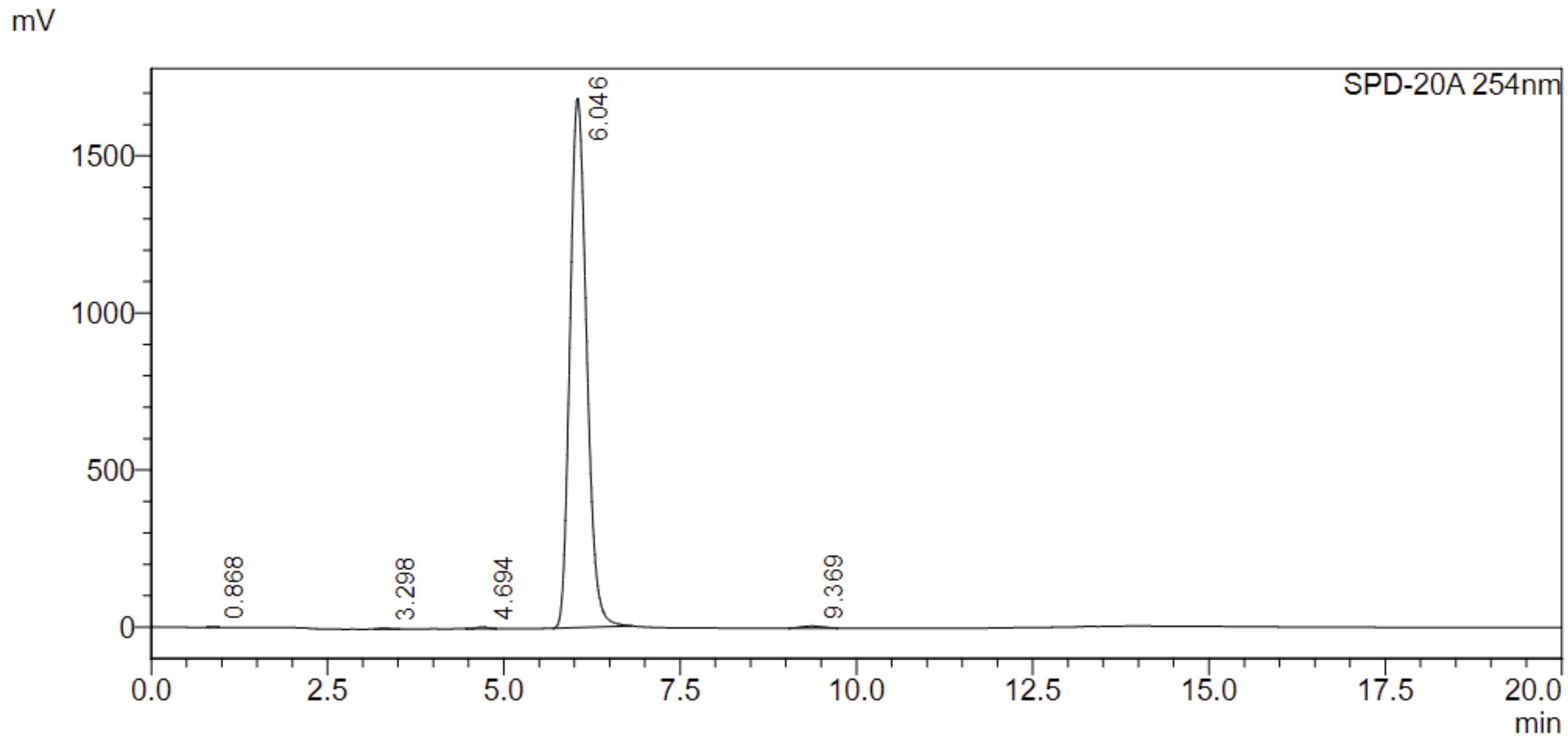

<Peak Table>

SPD-20A 254nm

Peak#	Ret. Time	Area	Height	Conc.	Unit	Mark	Name
1	0.868	1859	335	0.007		M	
2	3.298	27839	3559	0.099		M	
3	4.694	57146	5295	0.204		M	
4	6.046	27787039	1684735	99.193		M	
5	9.369	139264	6611	0.497		M	
Total		28013148	1700535				

HPLC of final compound **49** (flow: 1 ml/min, eleunt gradient: from water-methanol 55:45 to 15:85 in 30 min; detector: 254 nm).

di-*tert*-butyl 1-pentyl-2-(6-(trifluoromethoxy)benzo[*d*]thiazol-2-yl)hydrazine-1,2-dicarboxylate (**62**)

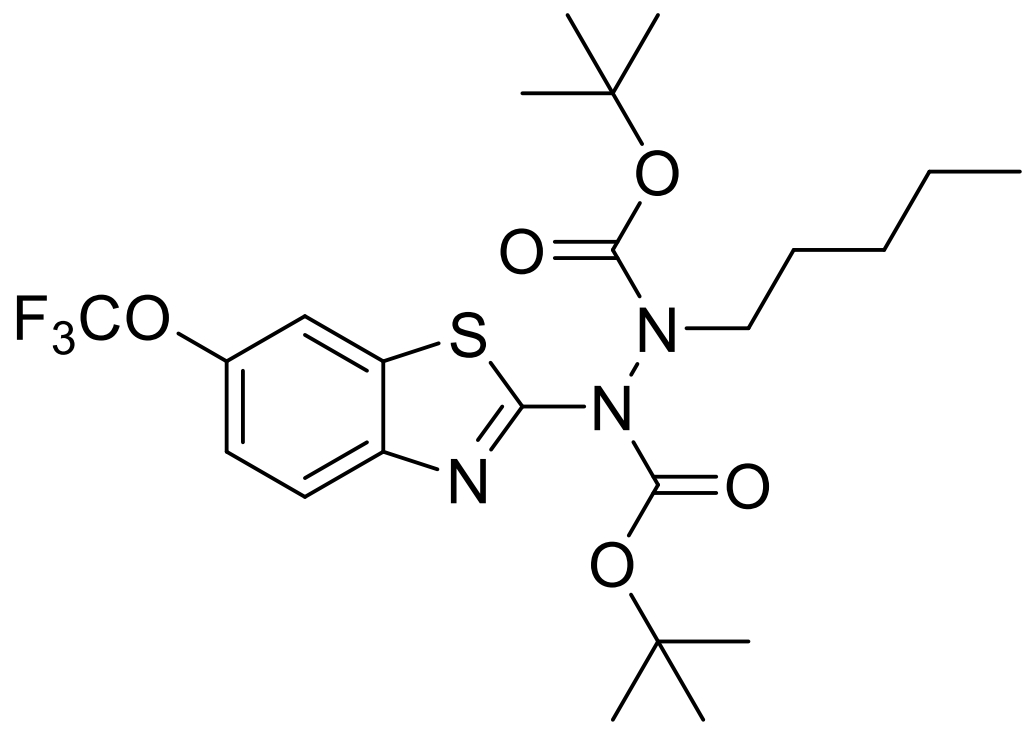

Chemical Formula: $C_{23}H_{32}F_3N_3O_5S$
Exact Mass: 519.20
Molecular Weight: 519.58

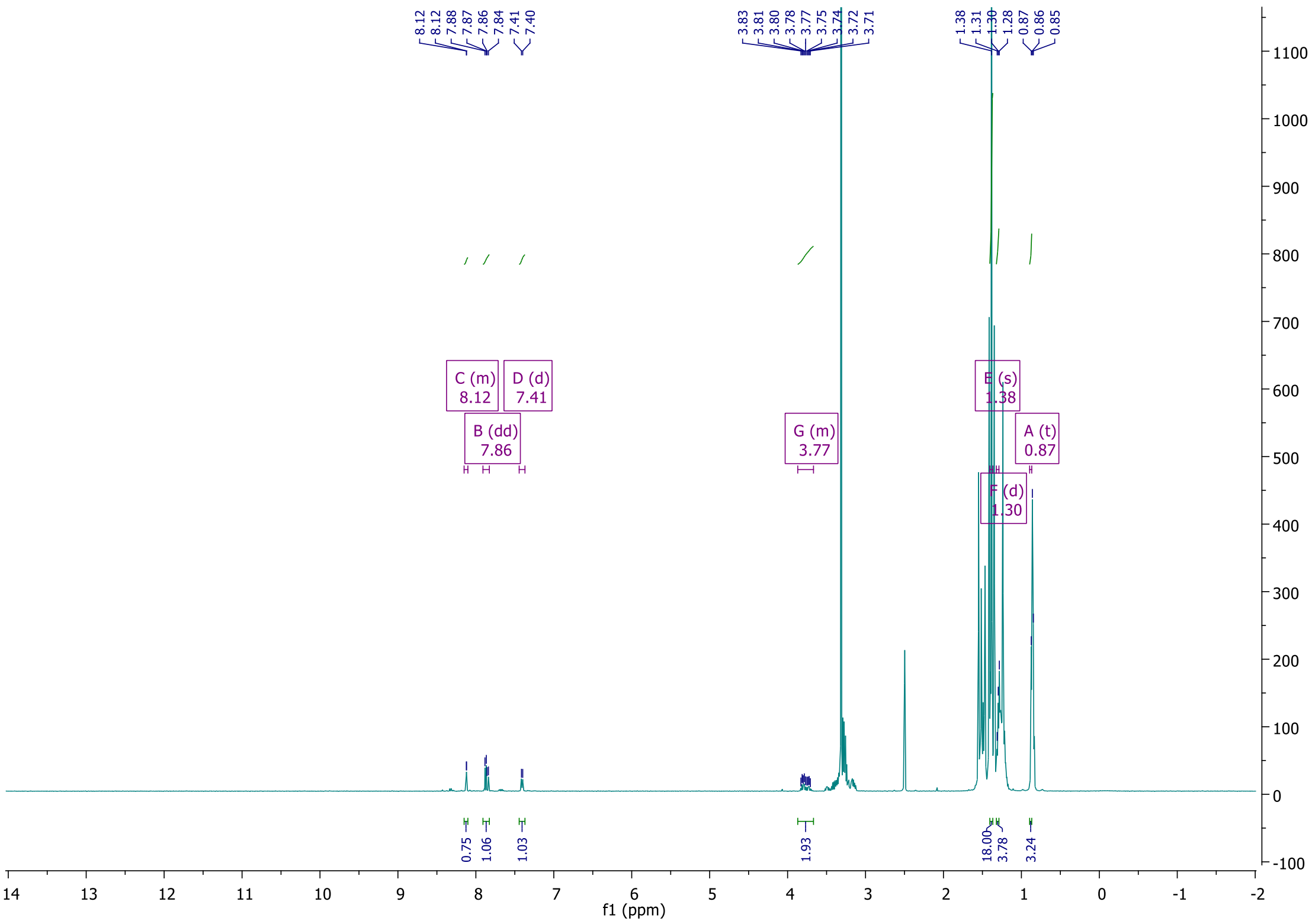

^{1}H-NMR of final compound **62** (DMSO-*d*$_6$).

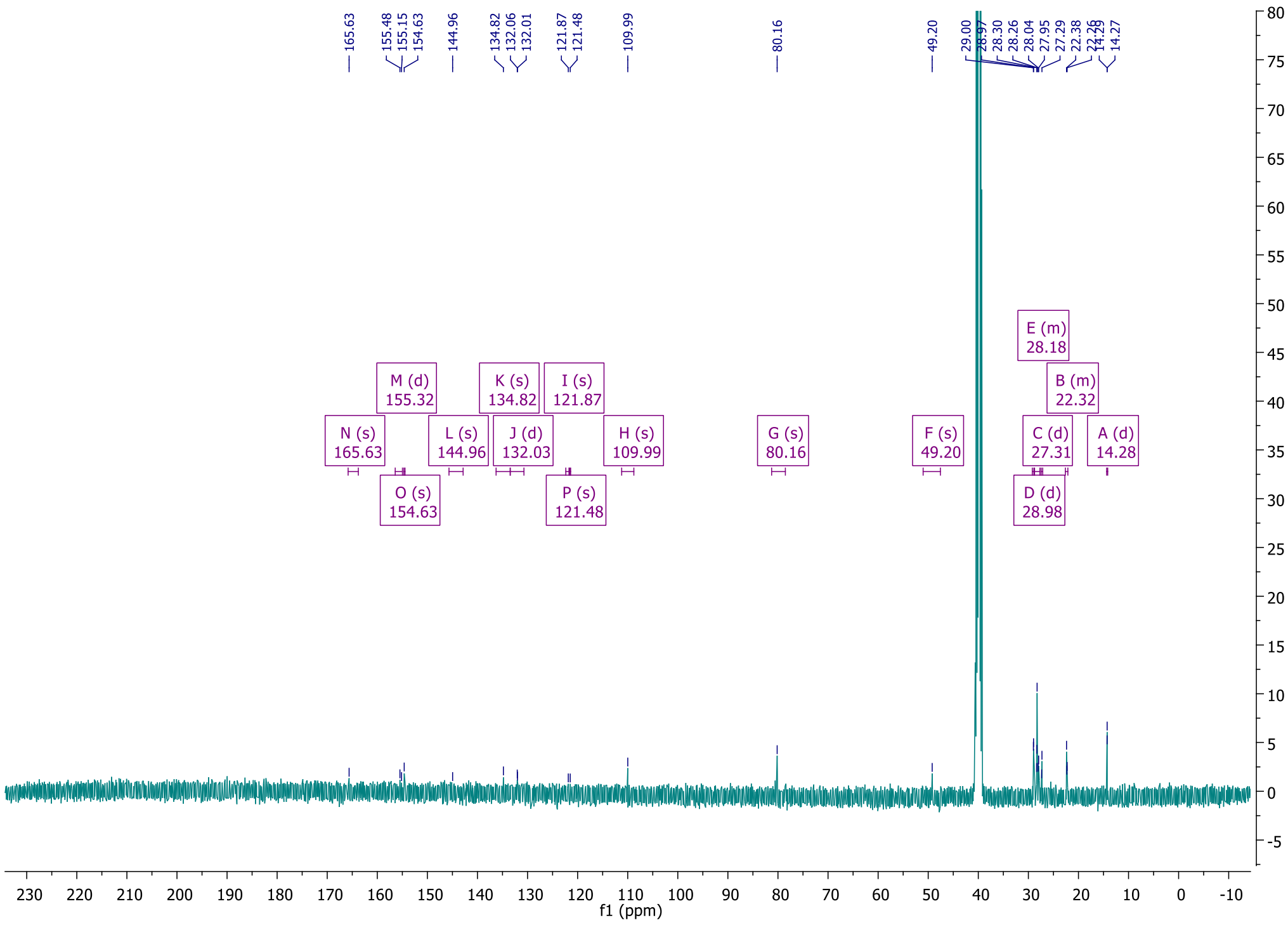

^{13}C-NMR of final compound **62** (DMSO-d_6).

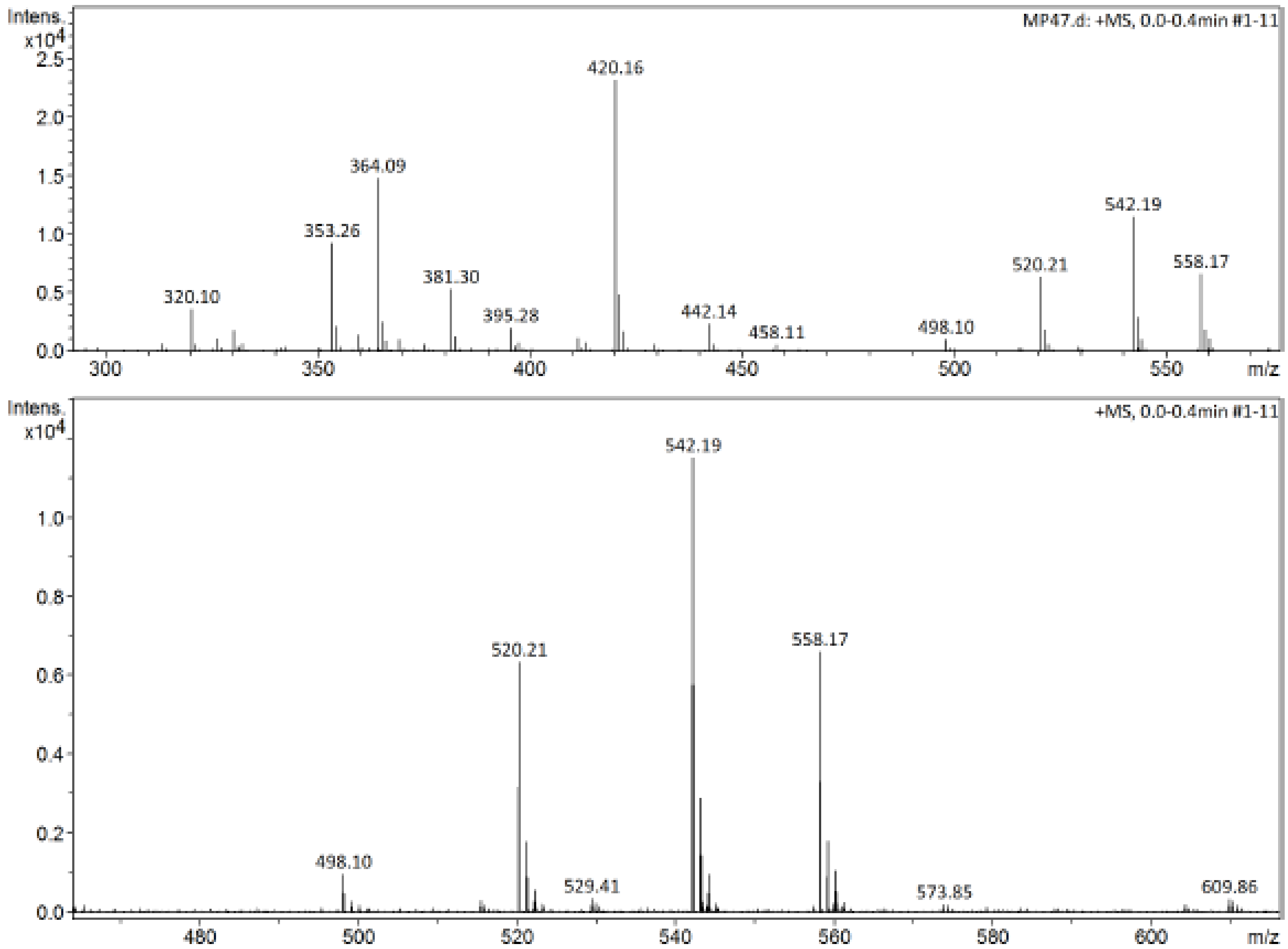

ES-MS of final compound **62** (MeOH).

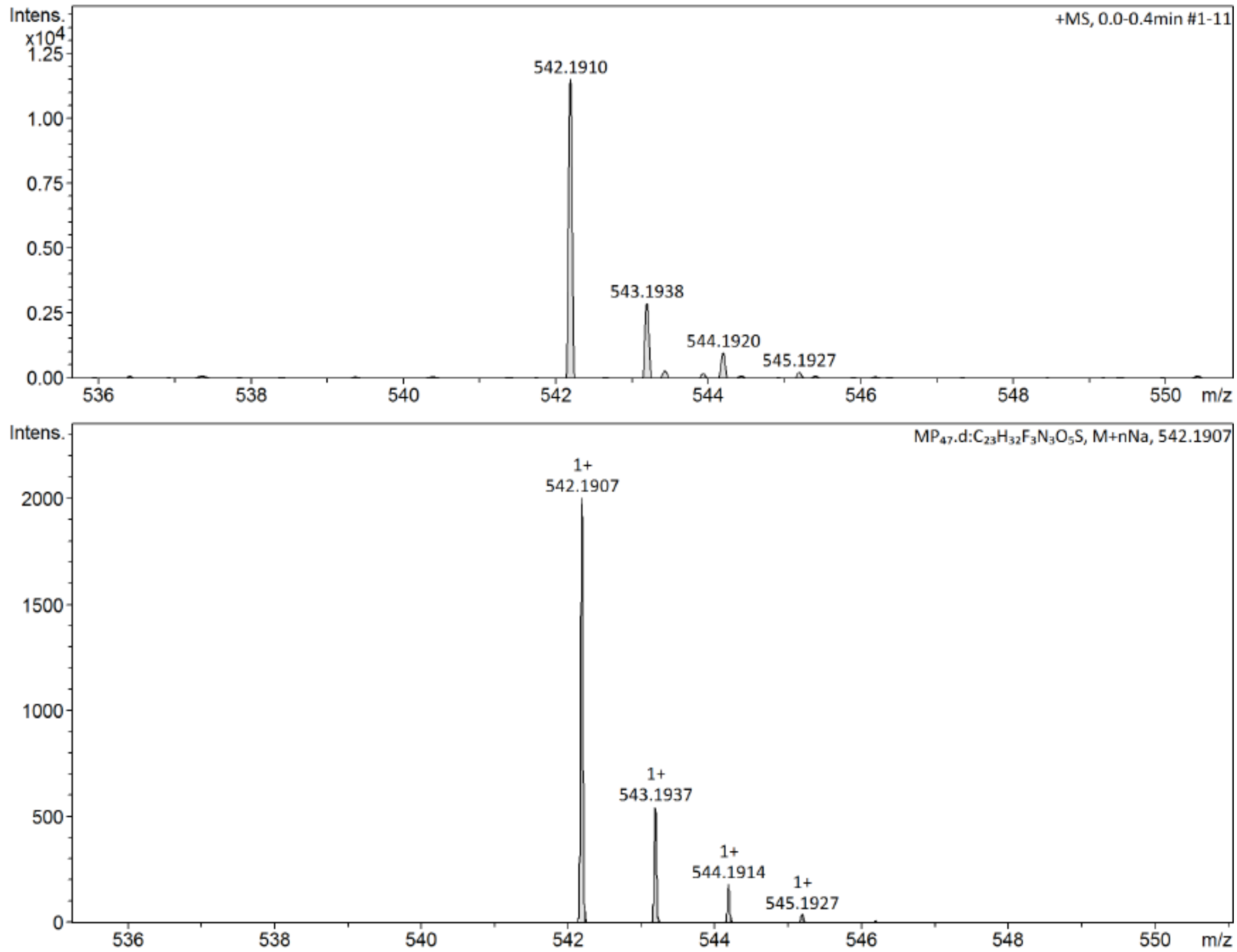

HRMS of final compound **62** (MeOH).

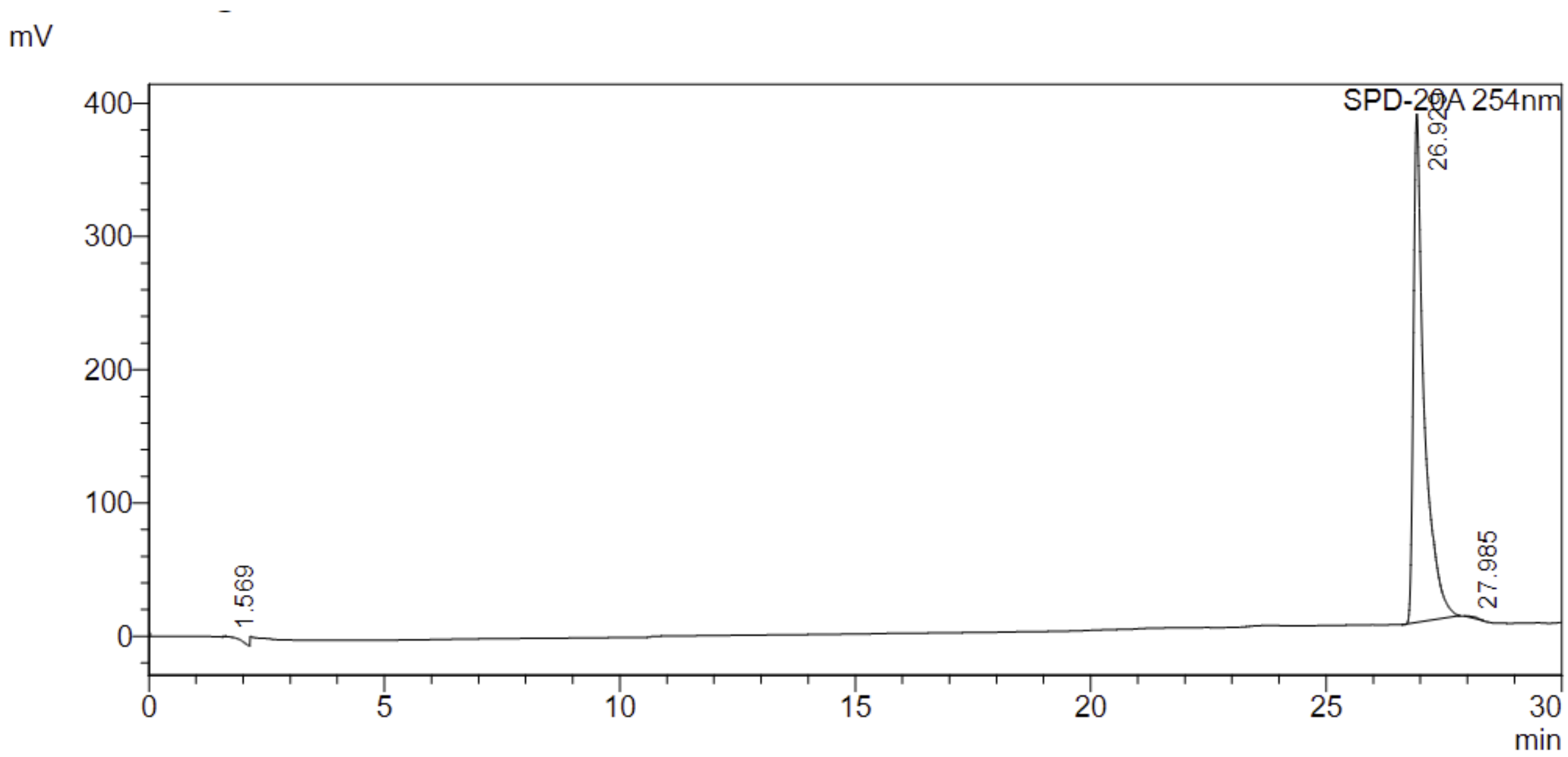

<Peak Table>

SPD-20A 254nm

Peak#	Ret. Time	Area	Height	Conc.	Unit	Mark	Name
1	1.569	1384	427	0.023		M	
2	26.929	6097336	381521	99.724		M	
3	27.985	15475	336	0.253		M	
Total		6114195	382284				

HPLC of final compound **62** (flow: 1 ml/min, eleunt gradient: from water-methanol 55:45 to 15:85 in 30 min; detector: 254 nm).

di-*tert*-butyl 1-benzyl-2-(6-(trifluoromethoxy)benzo[*d*]thiazol-2-yl)hydrazine-1,2-dicarboxylate (**65**)

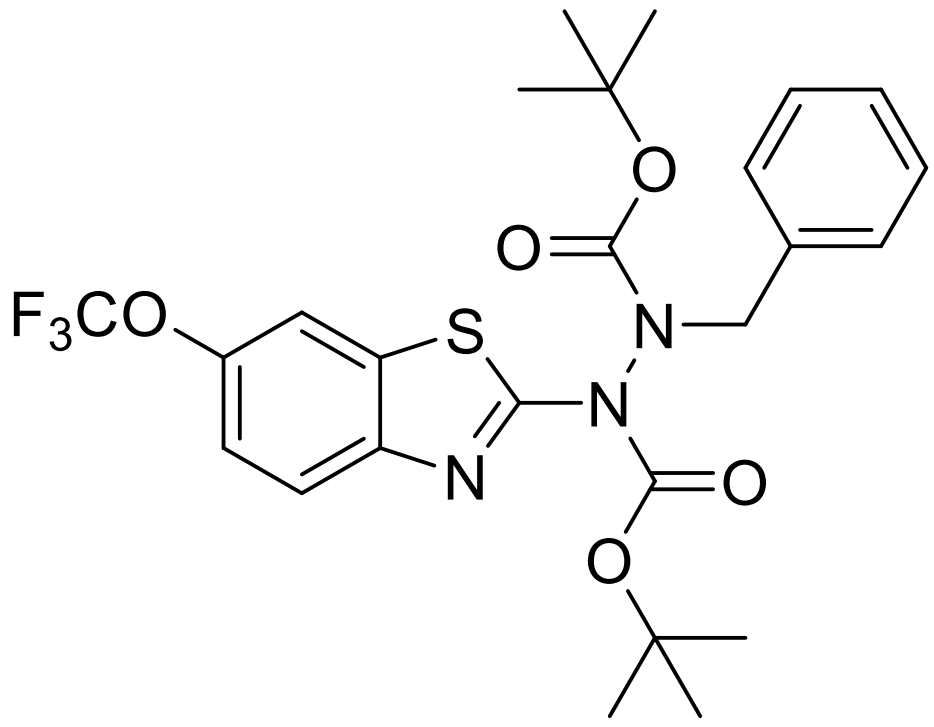

Chemical Formula: $C_{25}H_{28}F_3N_3O_5S$
Exact Mass: 539.17
Molecular Weight: 539.57

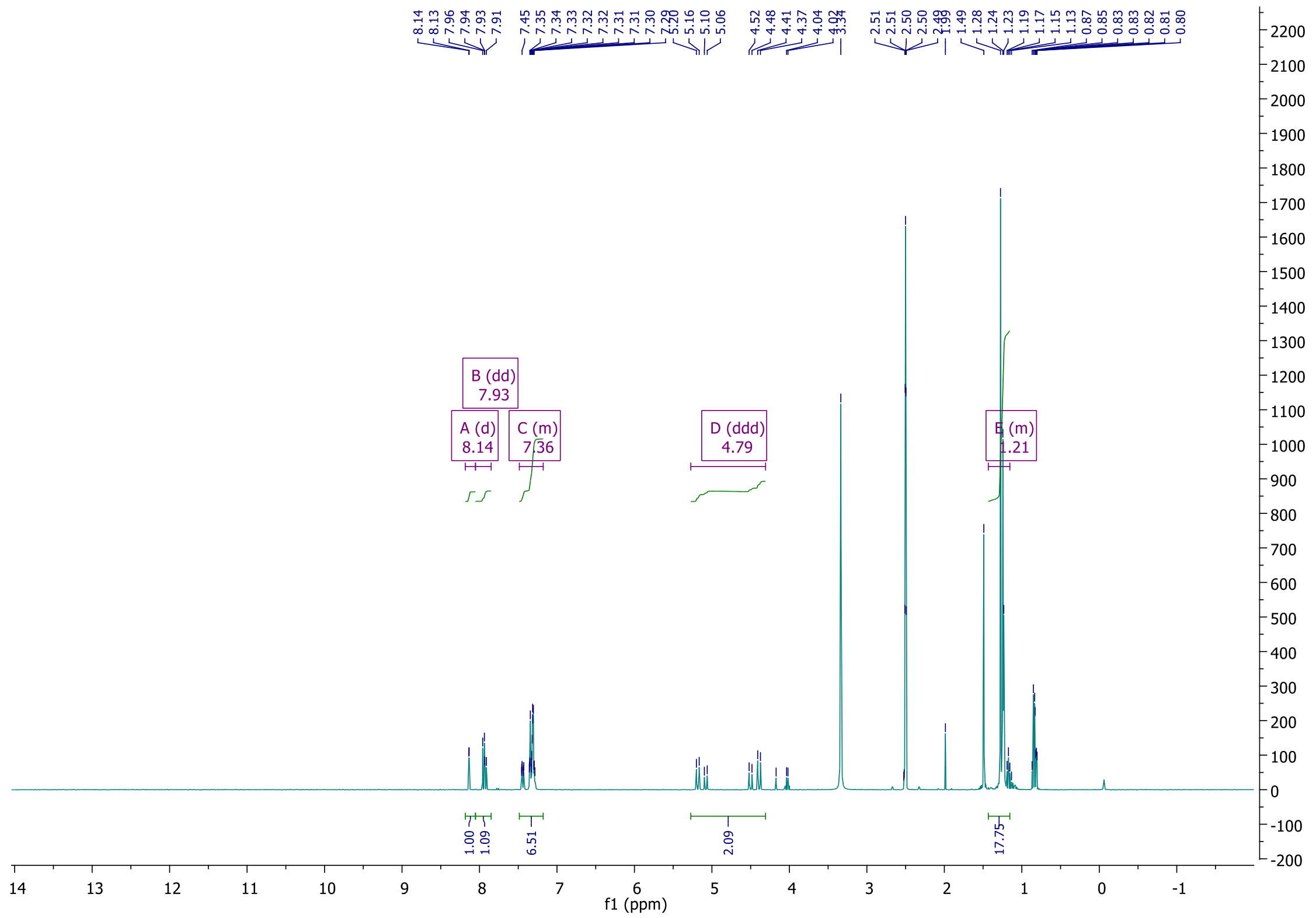

^{1}H-NMR of compound **65** (DMSO-d_6).

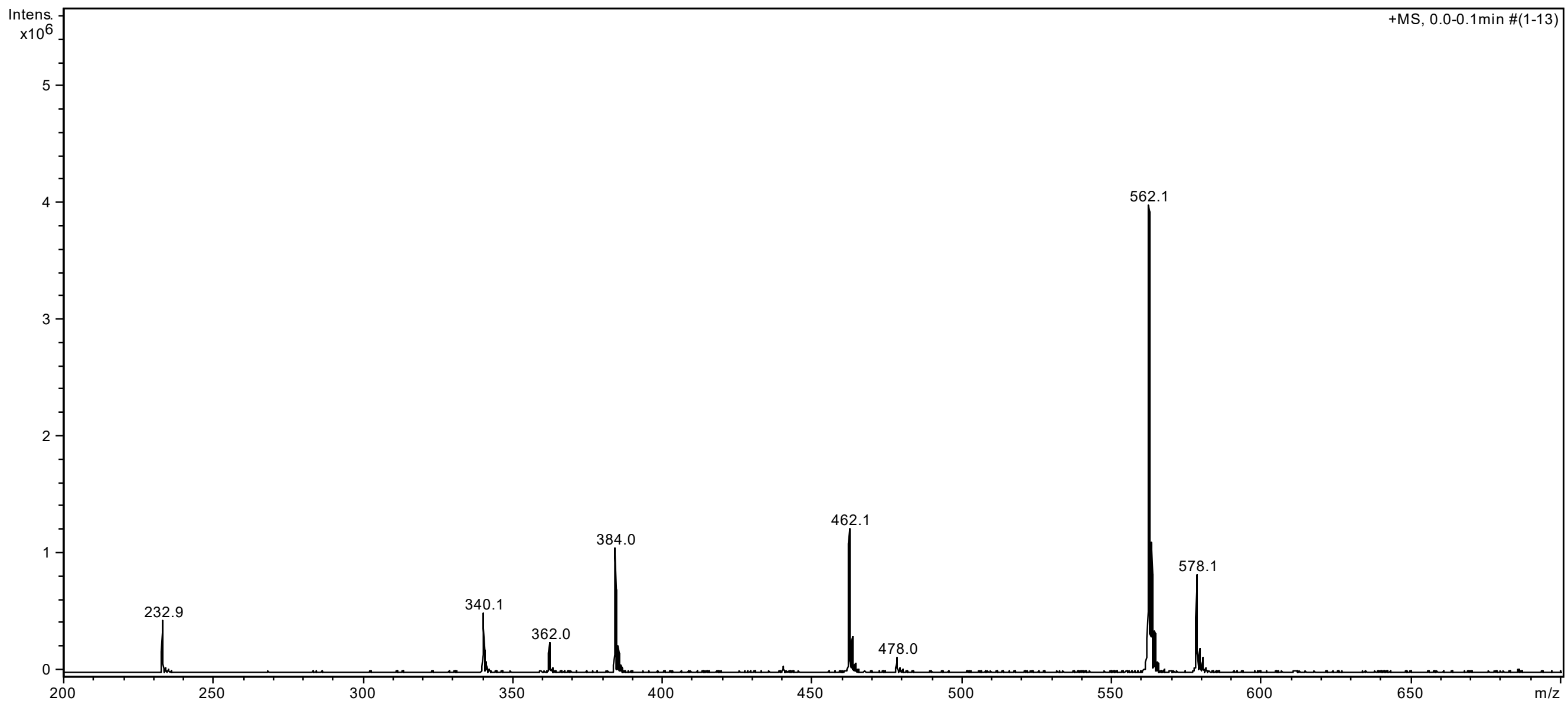

ES-MS of compound **65** (MeOH).

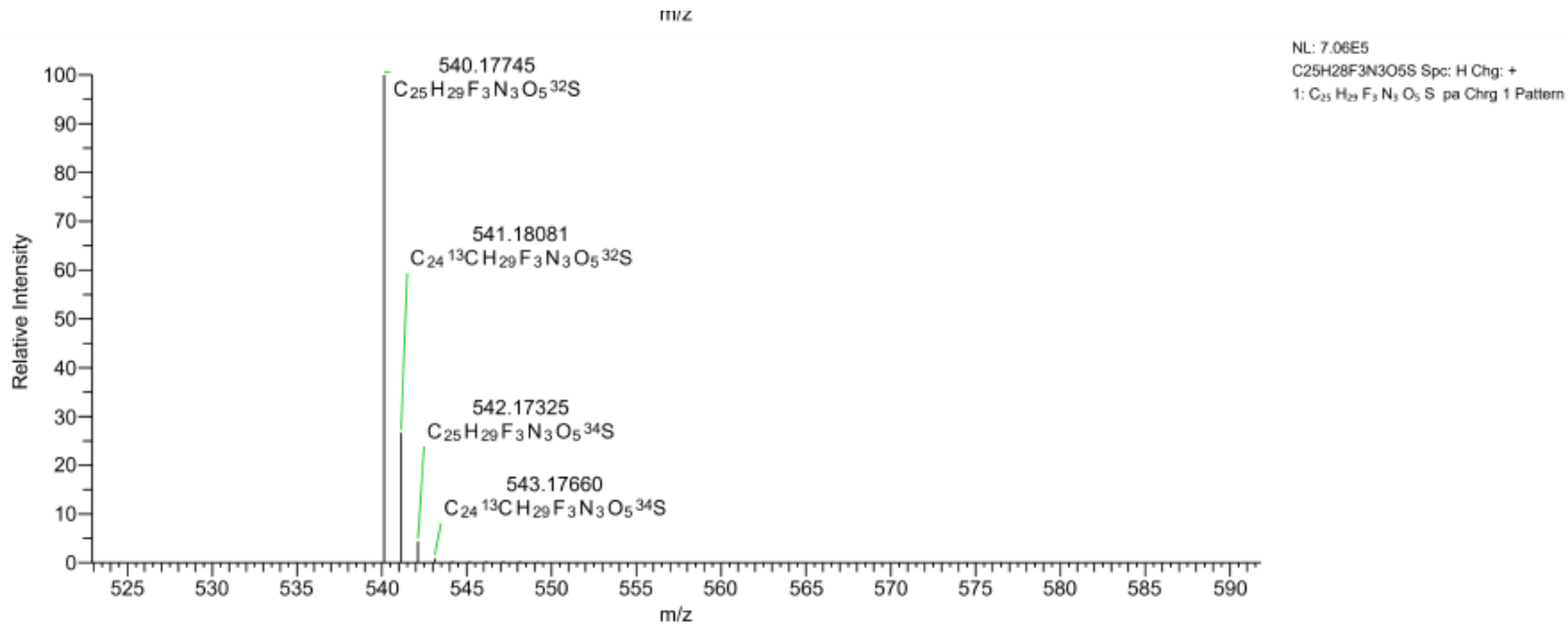

HRMS of final compound **65** (MeOH).

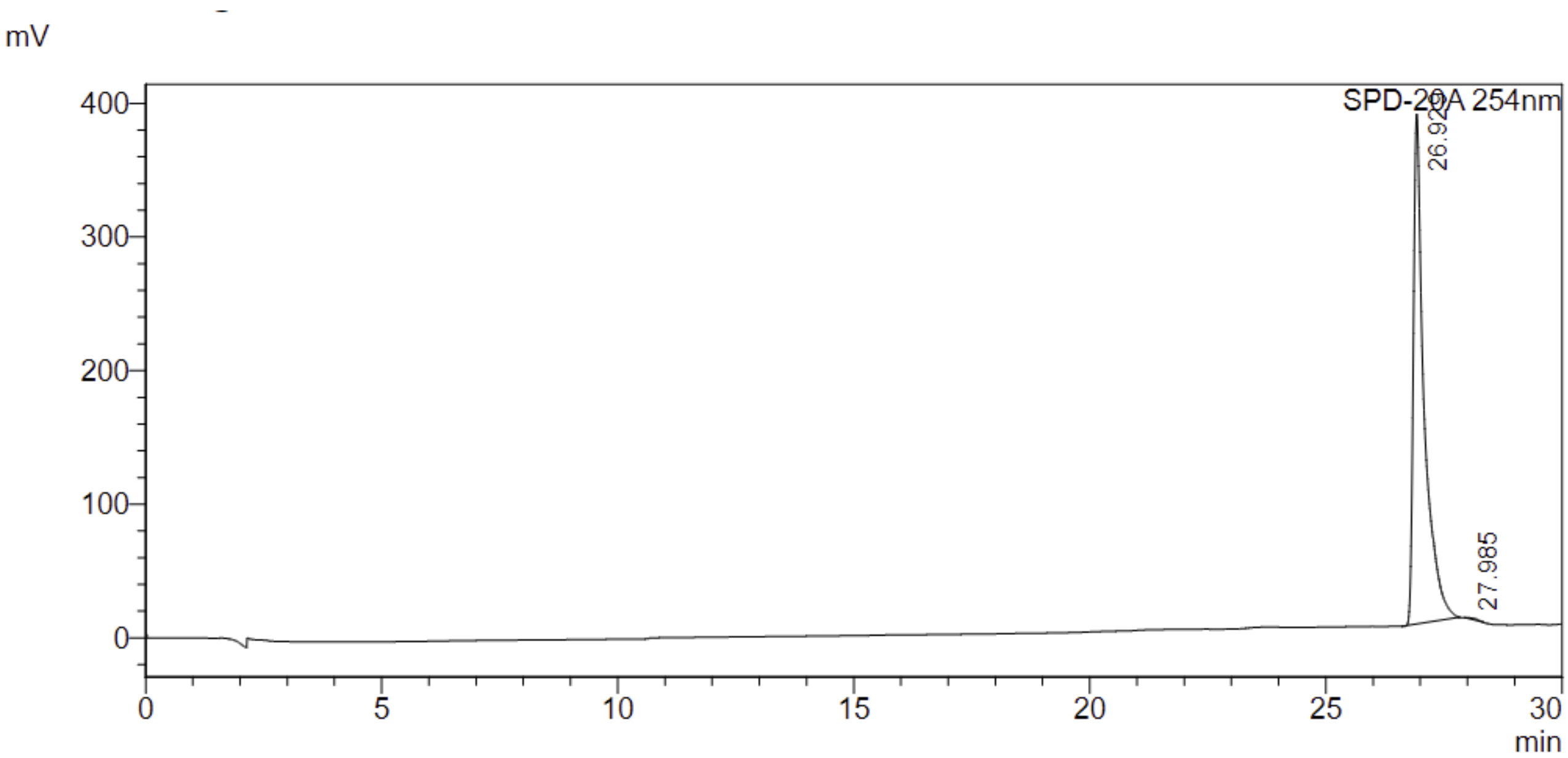

<Peak Table>

SPD-20A 254nm

Peak#	Ret. Time	Area	Height	Conc.	Unit	Mark	Name
1	26.929	6097336	381521	99.747		M	
2	27.985	15475	336	0.253		M	
Total		6112811	381857				

HPLC of final compound **65** (flow: 1 ml/min, eleunt gradient: from water-methanol 55:45 to 15:85 in 30 min; detector: 254 nm).

2. Concentration-inhibition curves for CK1δ

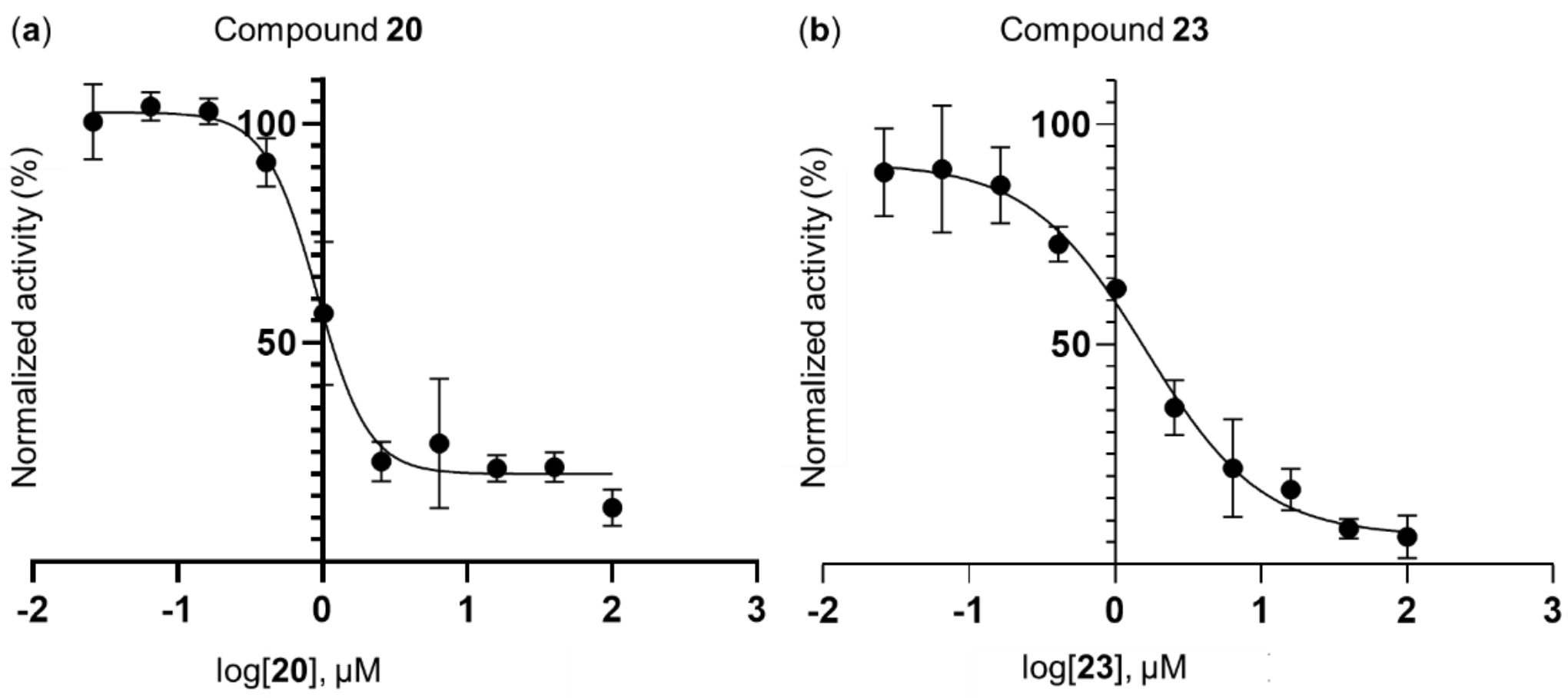

Figure S1: *IC_{50} concentration-inhibition curves of (a) compounds **20** and (b) **23** on Ck1δ, respectively.*

3. Stability studies

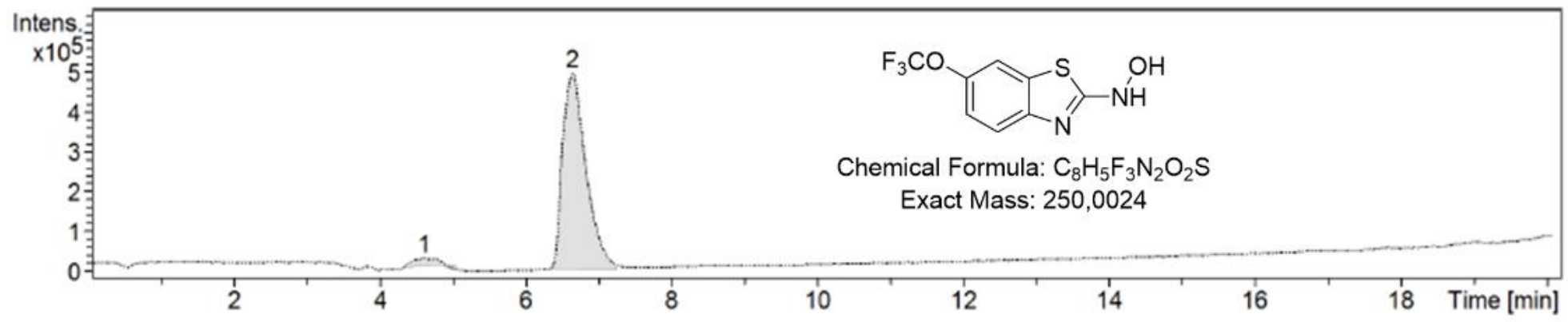

#	RT [min]	Area	Int. Type	I	S/N	Chromatogram	Max. m/z	FWHM [min]	Area Frac. %
1	4.6	490965	Manual	33908	15.4	BPC +All MS	223.9454	0.4	4.30
2	6.6	10918574	Manual	495899	386.5	BPC +All MS	251.0105	0.3	95.70

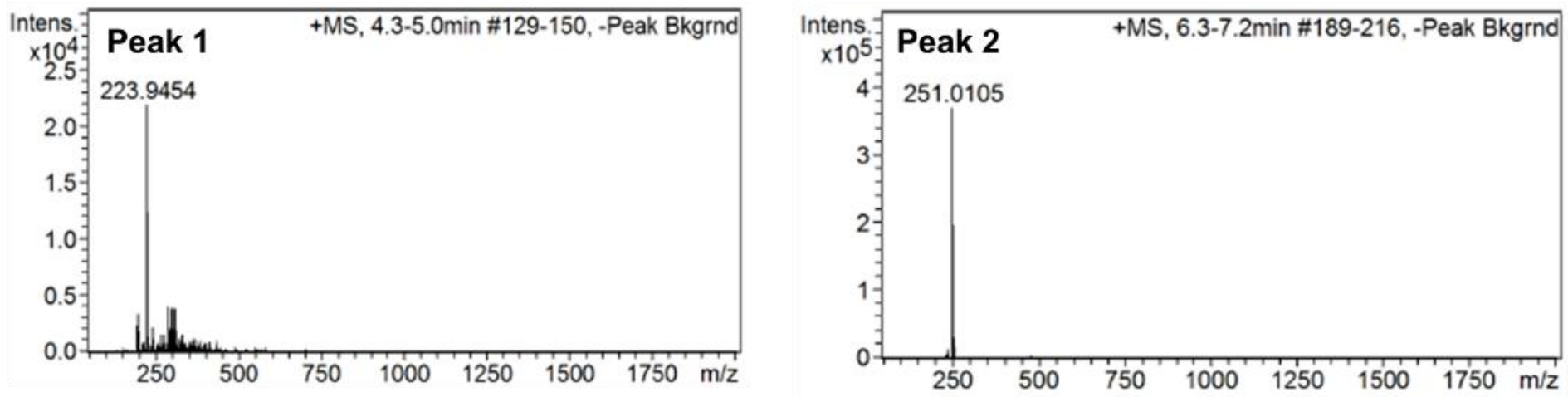

Figure S2: HPLC-MS chromatogram of compound **2** at t = 0. Purity reported of 95.70%. Below the chromatograms, masses of the two peaks observed are given. The mass error of compound **20** is +3.19 ppm.

(a)

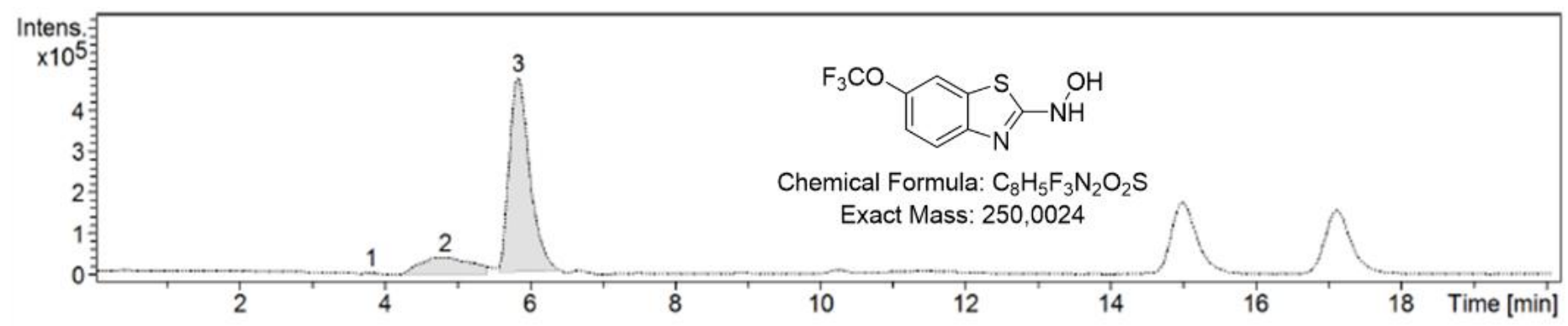

#	RT [min]	Area	Int. Type	I	S/N	Chromatogram	Max. m/z	FWHM [min]	Area Frac. %
1	3.8	60582	Manual	8482	11.9	BPC +All MS	226.9527	0.2	0.53
2	4.8	2036164	Manual	44296	80.6	BPC +All MS	223.9449	0.9	17.80
3	5.8	9343107	Manual	478239	908.2	BPC +All MS	251.0101	0.3	81.67

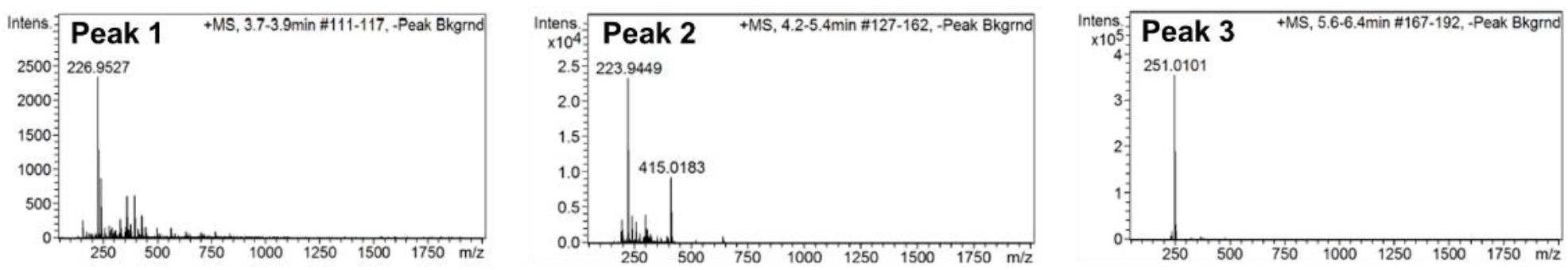

(b)

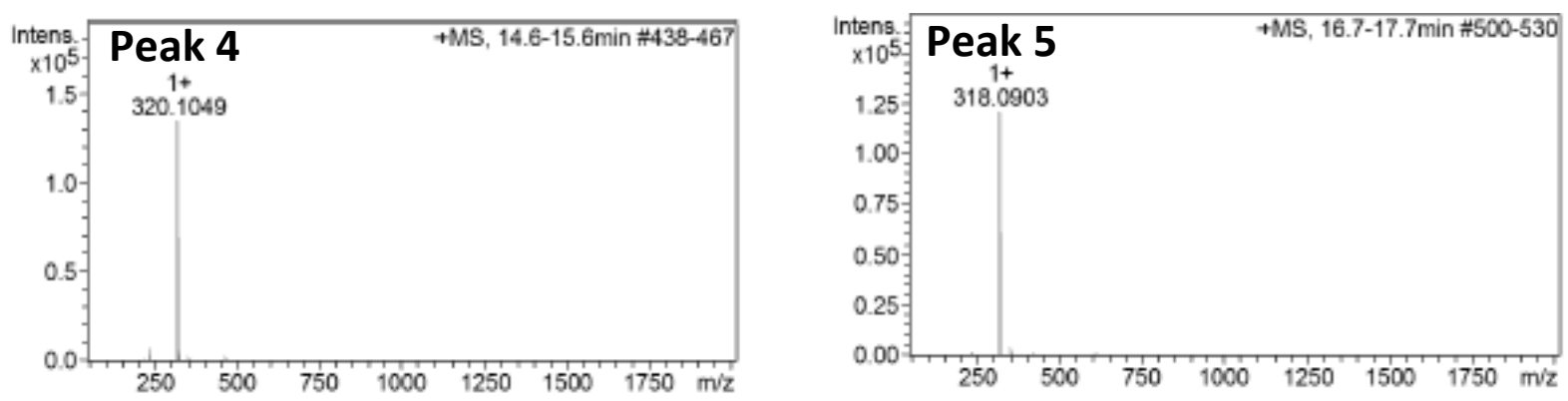

Figure S3: (a) HPLC-MS chromatogram of compound **2** at t = 28 days. Purity reported of 81.67%. Below the chromatogram, masses of the three peaks observed are given. The mass error of compound **2** is +1.59 ppm. **(b)** The peaks observed at approximately 15 and 17 min were not included in the purity calculation. Inspection of the corresponding mass spectra indicated that these signals originated from chromatographic carryover of compound 20 analysed in preceding injections and are therefore unrelated to compound 2 or its degradation products. The mass spectra associated with these peaks are reported.

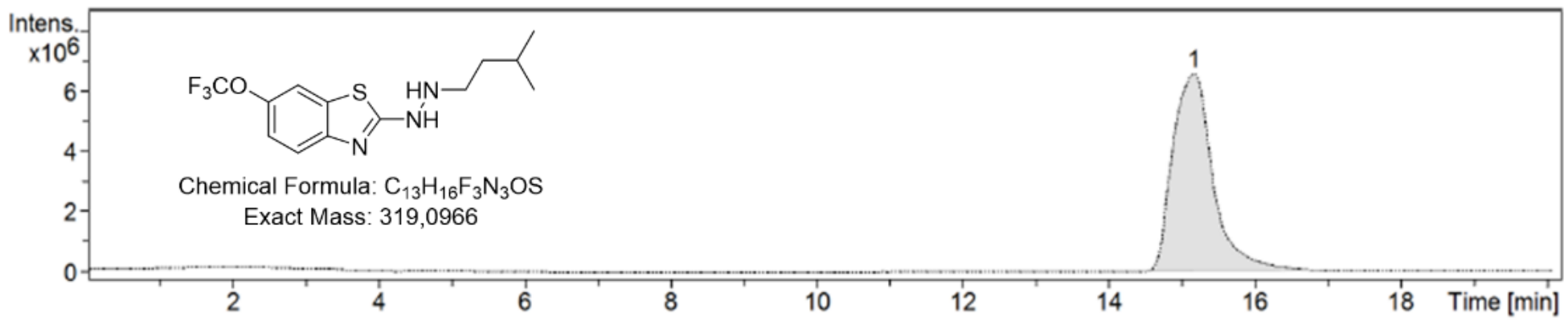

#	RT [min]	Area	Int. Type	I	S/N	Chromatogram	Max. m/z	FWHM [min]	Area Frac. %
1	15.2	247533104	Manual	6554707	3485.4	BPC +All MS	320.1033	0.6	100.00

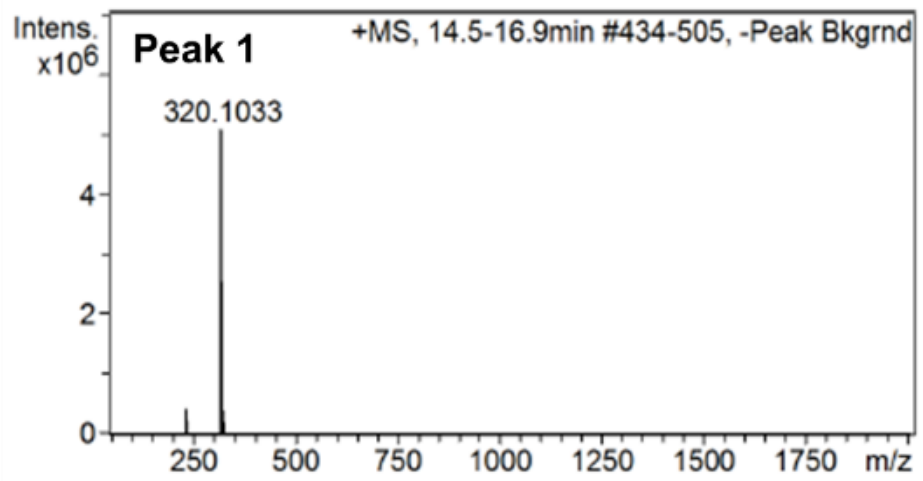

Figure S4: HPLC-MS chromatogram of compound **20** at t = 0. Purity reported of 100.00%. Below the chromatogram, the mass of peak observed is given. The mass error of compound **20** is −1.87 ppm.

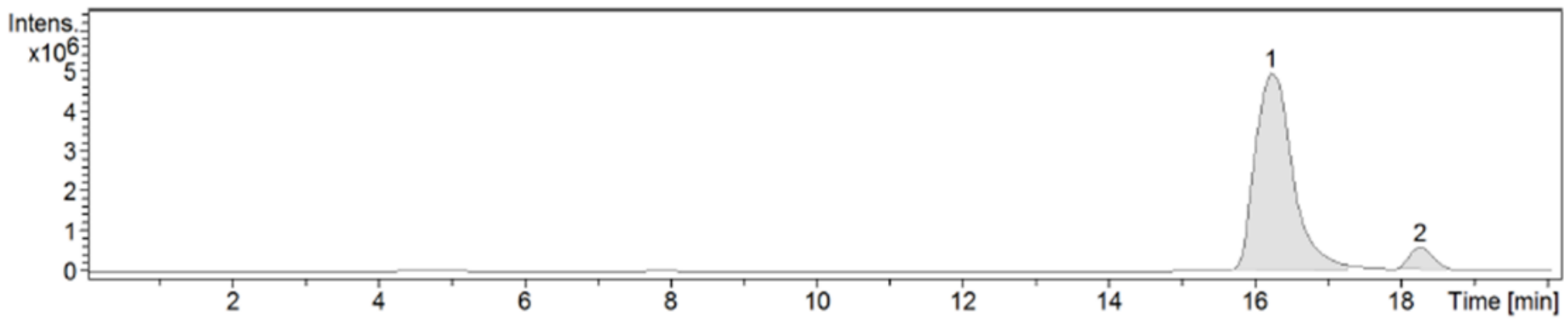

#	RT [min]	Area	Int. Type	I	S/N	Chromatogram	Max. m/z	FWHM [min]	Area Frac. %
1	16.2	174217824	Manual	4915071	16675.4	BPC +All MS	320.1030	0.5	93.77
2	18.3	11571374	Manual	595669	1807.1	BPC +All MS	318.0881	0.4	6.23

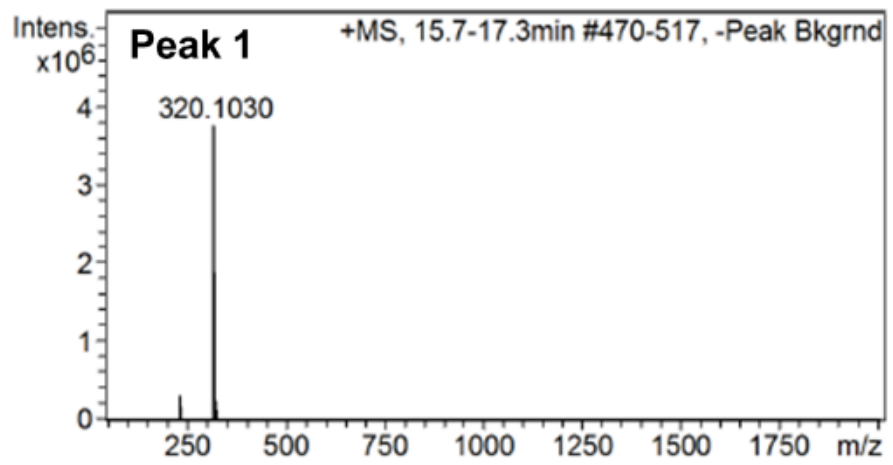

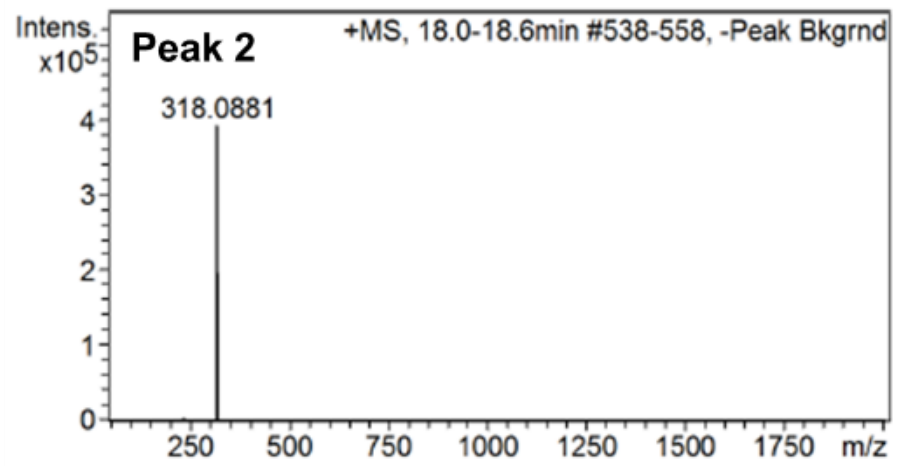

Figure S5: HPLC-MS chromatogram of compound **20** at t = 28 days. Purity reported of 93.77%. Below the chromatogram, masses of the two peaks observed are given. The mass error of compound **20** is −2.81 ppm.

The mass errors have been calculated by the following formula:

$$mass\ error\ (ppm) = \frac{\frac{m}{z_{exp}} - \frac{m}{z_{calc}}}{\frac{m}{z_{exp}}} \times 10^6$$

4. LipE profile

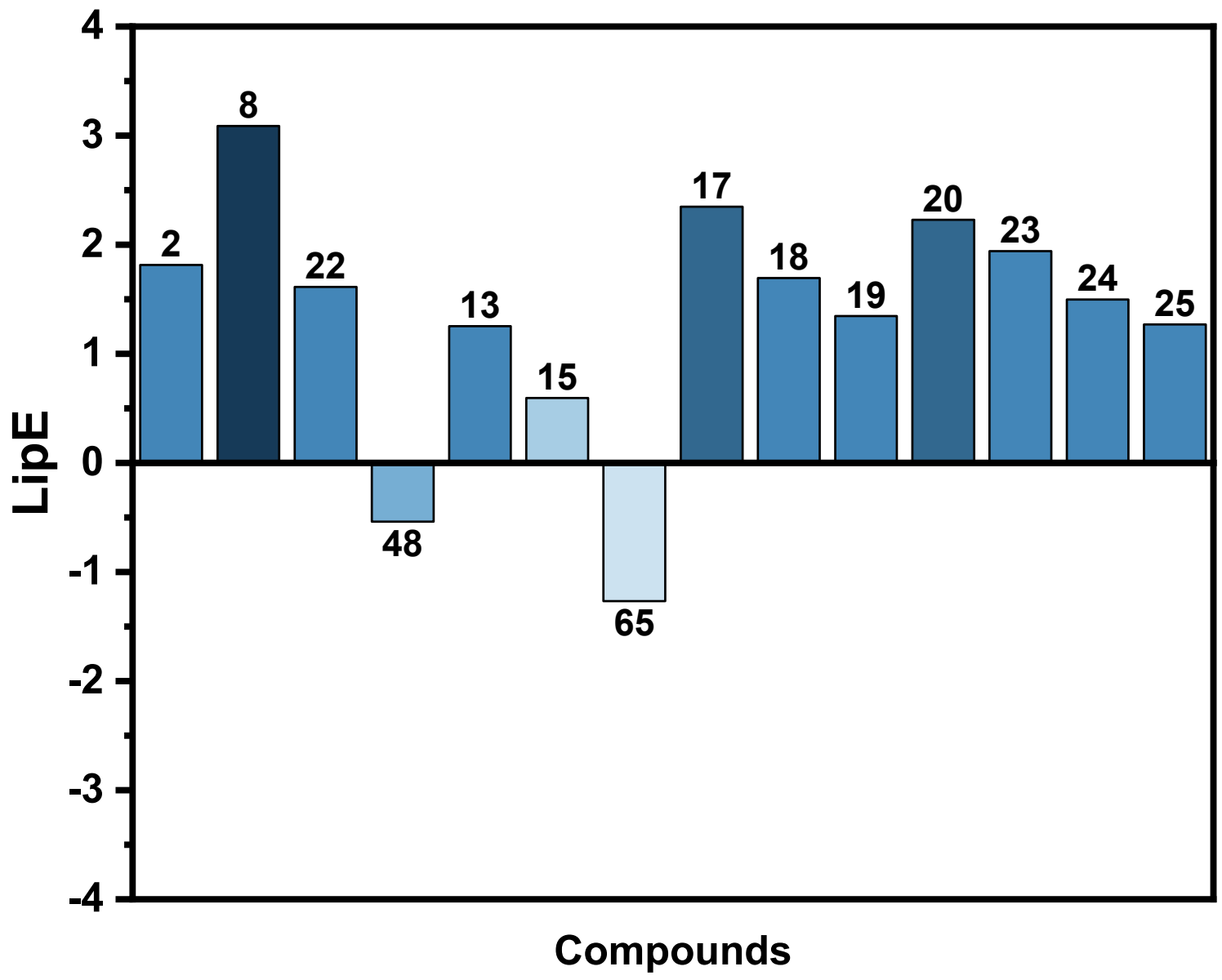

Figure S6: Lipophilicity efficiency analysis of the active benzo[d]thiazole derivatives. LipE values calculated for the active compounds as pIC_{50} minus ClogP, showing the efficiency profile of the hydroxylamine and hydrazine analogues. Positive values indicate compounds in which CK1δ potency is better balanced against lipophilicity, whereas lower or negative values reflect a greater lipophilic cost for the observed activity.

5. *In vitro* evaluation of BBB-permeability

Obtained P_{app} have been filtered according to the literature: the BBB permeability in humans is predicted as positive if it is higher than $4 \cdot 10^{-6}$ cm/s and negative if it is lower than $2 \cdot 10^{-6}$ cm/s. Therefore, applying these limits to the equation of calibration line (Figure S7) it is possible to establish cut-off values: in this case compounds with P_{app} higher than $1.1 \cdot 10^{-6}$ cm/s and $2.1 \cdot 10^{-6}$ cm/s, experiment 1 (Figure S7a and S8a) and 2 (Figure S7b and S8b) respectively, are classified as CNS+ while P_{app} permeations below $0.6 \cdot 10^{-6}$ cm/s and $1.4 \cdot 10^{-6}$ cm/s represent CNS- derivatives.

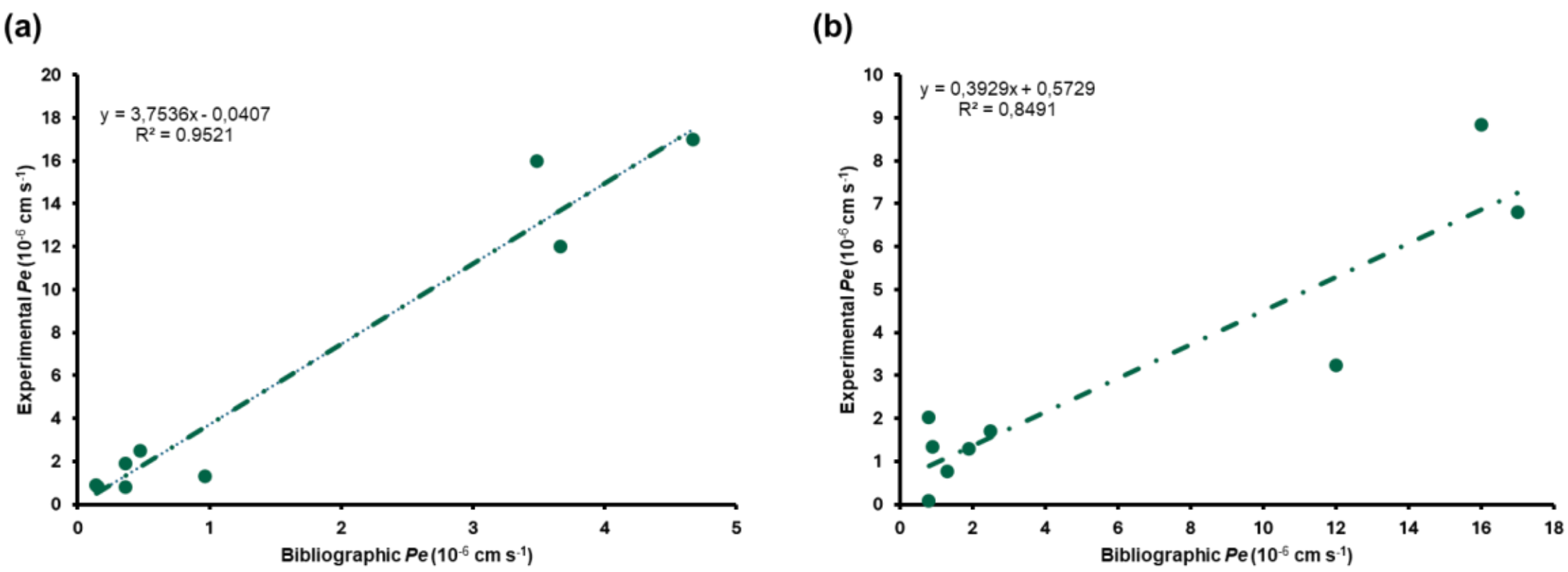

Figure S7: BBB-PAMPA calibration plots. Correlation between experimentally determined and literature-reported effective permeability values, Pe, for ten reference drugs used to calibrate the BBB-PAMPA assay and define the CNS permeability cut-off values. Panels (a) and (b) show the calibration lines obtained for batches A and B, respectively.

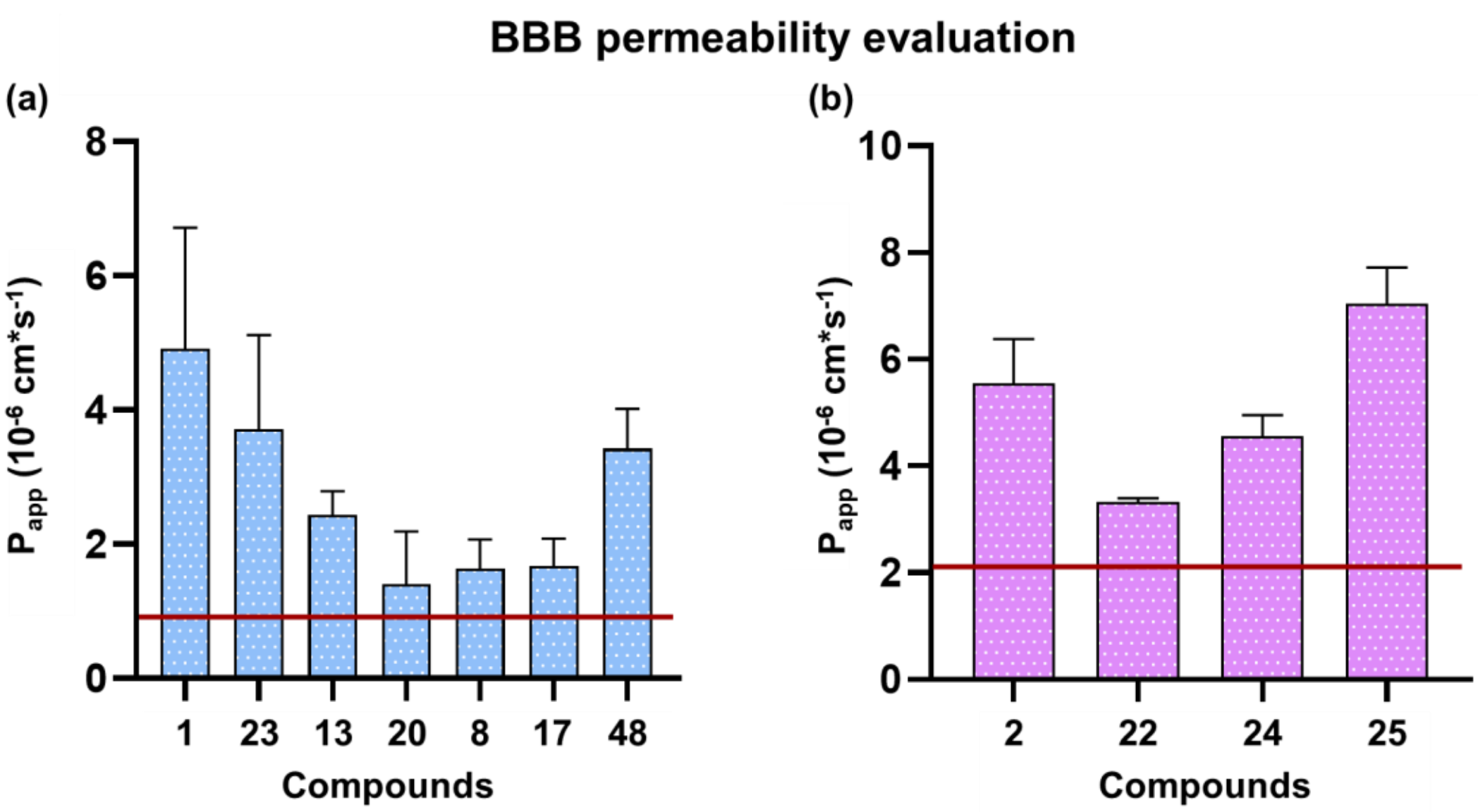

Figure S8: BBB-PAMPA permeability evaluation of riluzole and selected benzo[d]thiazole derivatives. Histograms reporting the apparent permeability values, P_{app}, obtained for the tested compounds in the BBB-PAMPA assay. Panels (a) and (b) show the two independent experimental batches, respectively. The red horizontal line indicates the CNS+ permeability cut-off defined from the calibration with reference drugs.

Table S1: *Passive BBB permeation for selected compounds.*

Compounds	P_{app} (10^{-6} cm s^{-1})[a]	BBB perm.[b]
**1**	4.9 ± 2.2	CNS+
**2**	5.6 ± 0.8	CNS+
**8**	1.6 ± 0.4	CNS+
**22**	3.3 ± 0.1	CNS+
**48**	3.4 ± 0.6	CNS+
**13**	2.4 ± 0.4	CNS+
**17**	1.7 ± 0.4	CNS+
**18**	7.0 ± 0.7	CNS+
**20**	1.4 ± 0.9	CNS+
**23**	4.3 ± 1.7	CNS+
**24**	4.6 ± 0.4	CNS+
**25**	7.0 ± 0.7	CNS+

[a] Apparent permeability (P_{app}) determined using the BBB-PAMPA assay. [b] BBB behavior was classified based on BBB-PAMPA P_{app} thresholds, categorizing compounds as permeable (CNS+), non-permeable (CNS−), or intermediate permeability (CNS+/−).

6. Thermal Shift Assay (TSA)

Table S2: *Table of shift values for TSA performed for compound **20**. The "condition" reports concentrations of compound (multiples of protein concentration), the "average" column indicates the mean of temperature of technical triplicate accompanied by the standard deviation ("SD") while the "ΔT°C" (difference of temperature) column reports the temperature shift.*

**Conditions**	**T_m**	**ΔT (°C)**	**SD triplicates**
**CK1δ**	51.0		0
**DMSO** 2.5%	51.0	(0)	0
**20** 0.5x	51.0	0	0
**20** 1x	51.0	0	0
**20** 3x	51.7	+0.7	0.6
**20** 5x	52.0	+1	0
**20** 10x	52.7	+1.7	0.6
**20** 15x	53.0	+2	0

7. Kinase selectivity studies

Table S3: *IC_{50} of compounds **2**, **20**, **23**, and activity percentage at* GSK3β.

Compounds	GSK3β<br>IC_{50} μM[a] (% activity at 40 μM)[b]
**2**	23.1 ± 1.0 (45.7% ± 2.6)
**20**	n. d. (100% ± 0)
**23**	16.4 ± 1.1 (29.4% ± 5.8)

[a] Data represent the mean ± SD of three independent experiments performed in technical duplicate. [b] Data represent the % of activity at 40 μM concentration expressed as a mean ± SD of two independent experiments performed in technical duplicate; n.d.: not determined.

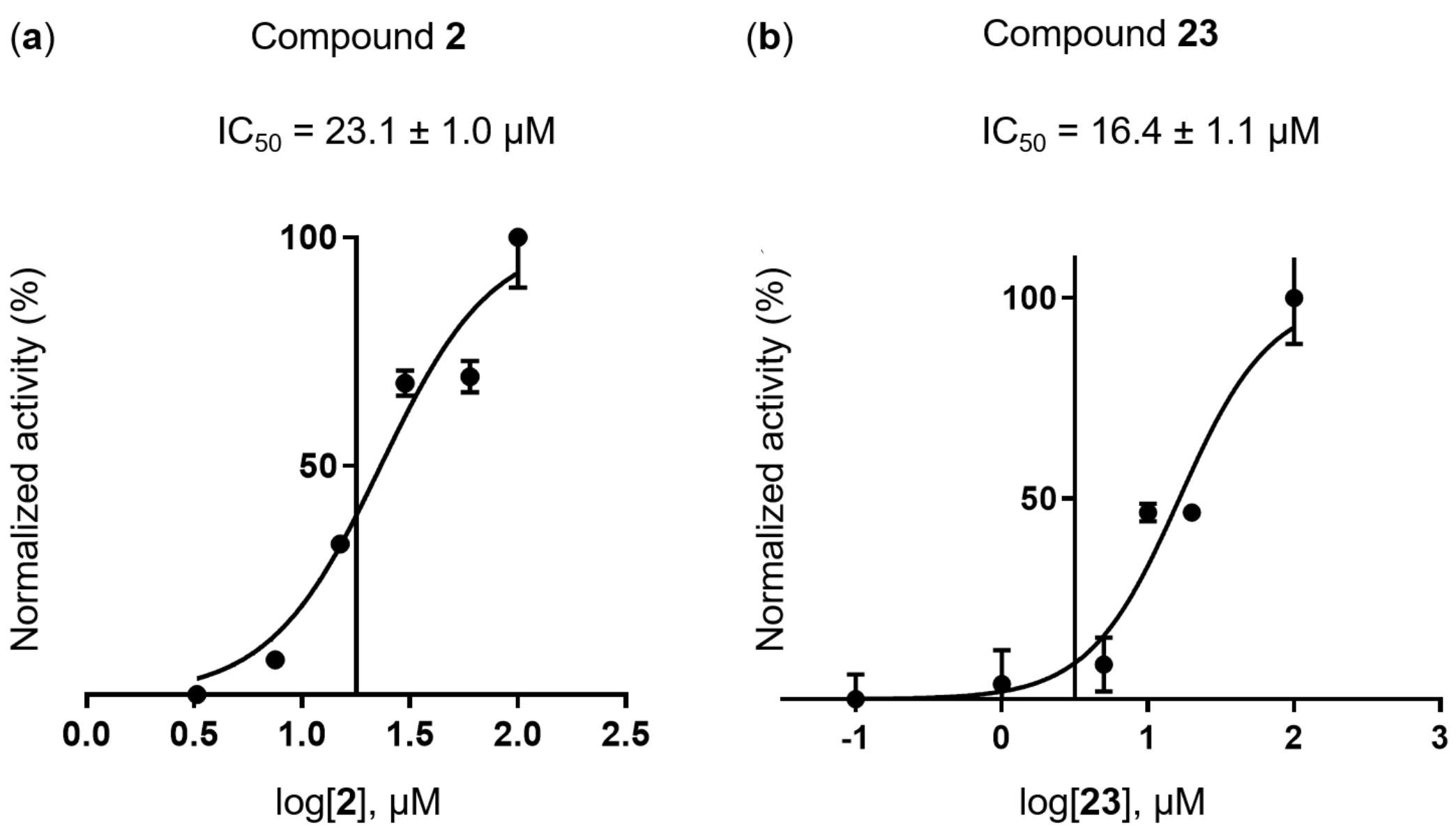

Figure S9: IC_{50} concentration-inhibition curves of (a) compounds **2** and (b) **23** on GSK3β, respectively.

***Table S4:** IC$_{50}$s of compounds **20**, **23**, and activity percentage towards CK1ε and p38α, respectively.*

Compounds	CK1ε<br>IC$_{50}$ μM[a]	p38α<br>% activity at 50 μM[b]
**20**	6.20 ± 2.01	82.6% ± 0.59
**23**	8.53 ± 2.47	87.8% ± 2.9

[a] Data represent the mean ± SD of three independent experiments performed in technical duplicate. [b] Data represent the % of activity at 50 μM concentration expressed as a mean ± SD of two independent experiments performed in technical duplicate.

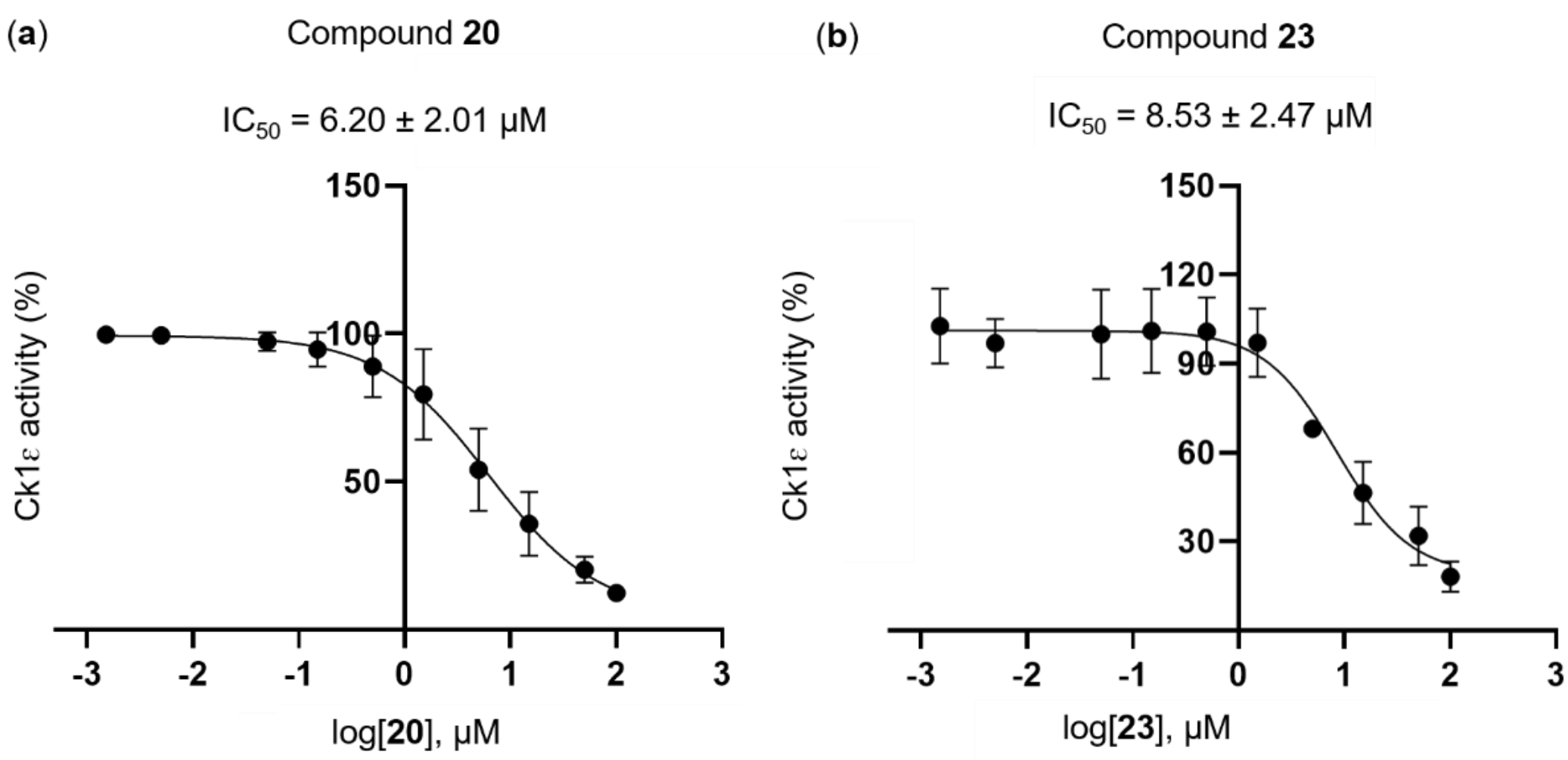

Figure S10: IC$_{50}$ concentration-inhibition curves of (a) compounds **2** and (b) **23** on CK1ε, respectively.

8. MTT assay

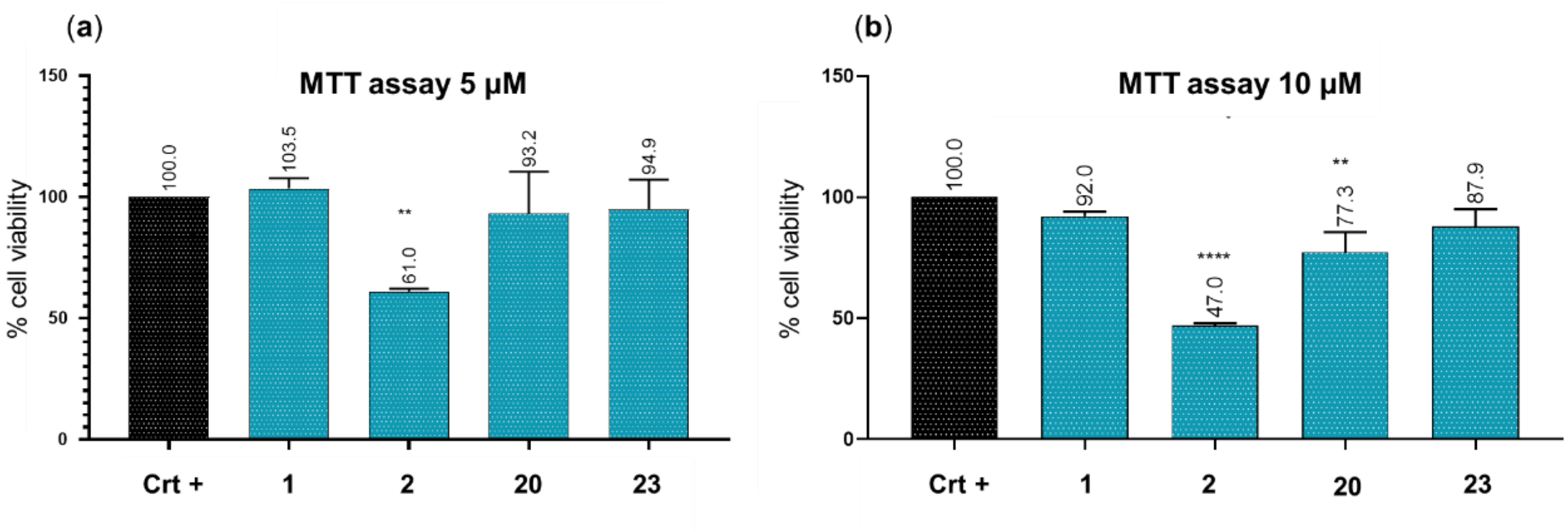

Figure S11: Cytotoxicity evaluation of riluzole and selected benzo[d]thiazole derivatives in SH-SY5Y cells. MTT assay performed after treatment with riluzole, metabolite 2, and hydrazine derivatives **20** and **23** at 5 µM (a) and 10 µM (b). Cell viability is reported as percentage relative to untreated control cells. Data are expressed as mean ± SEM from three independent experiments, each calculated from the mean of six wells per compound. versus control cells: *p < 0.1, **p < 0.01, ***p < 0.001, ****p < 0.0001.
